# Induced ubiquitination of the partially disordered estrogen receptor alpha via a 14-3-3 directed molecular glue-PROTAC

**DOI:** 10.1038/s41467-026-75333-w

**Published:** 2026-07-15

**Authors:** Carlo J. A. Verhoef, Charlotte Crowe, Mark A. Nakasone, Aitana DeLaCuadra-Basté, Tessa Harzing, Naomi A. S. Span, Gajanan Sathe, Laura C. Demmers, Kentaro Iso, Christian Ottmann, Luc Brunsveld, Alessio Ciulli, Peter J. Cossar

**Affiliations:** 1 Laboratory of Chemical Biology, Department of Biomedical Engineering and Institute for Complex Molecular Systems, Eindhoven University of Technology, Eindhoven, Netherlands; 2 Centre for Targeted Protein Degradation, School of Life Sciences, University of Dundee, Dundee, UK; 3 Ambagon Therapeutics, Catalyst Business Center, Eindhoven, Netherlands; 4 Present Address: Birkbeck University of London, ISMB EM Facility, London, UK

**Keywords:** Post-translational modifications, Structure-based drug design, Electron microscopy, Molecular biophysics

## Abstract

Proteins lacking defined ligandable pockets remain challenging drug targets. Here, we develop a molecular glue-based PROTAC (^MG^PROTAC) approach that chemically conjugates a molecular glue stabilizer to a VHL-recruiting ligand to capture and ubiquitinate the 14-3-3/Estrogen receptor α (ERα) complex. Our designed ^MG^PROTACs engage a composite interface between 14-3-3 and the disordered F-domain of ERα, promoting cooperative complex formation and targeted ubiquitination. Biophysical characterization revealed distinct linker-dependent cooperativities across the ^MG^PROTAC series, which influenced both cellular permeability and ubiquitination efficiency. Cryo-EM of the most cooperative ^MG^PROTAC uncovered de novo VHL–14-3-3ζ contacts, while molecular dynamics simulations rationalize the stabilizing interactions underlying cooperativity. Strikingly, fine-tuning linker design enables selective ubiquitination of distinct complex subunits. These findings establish a structural and mechanistic framework for integrating molecular glue and PROTAC principles, expanding the scope of drug discovery to previously intractable protein complexes.

## Abstract (web summary)

Proteins lacking defined ligandable pockets are challenging drug targets. Here the authors combine molecular glue-stabilizer and PROTAC strategies to enable ubiquitination of the 14-3-3/estrogen receptor α complex, revealing cooperative linker-dependent activity and a structural basis for targeted E3 ligase recruitment.

## Introduction

**Fig. 1:**
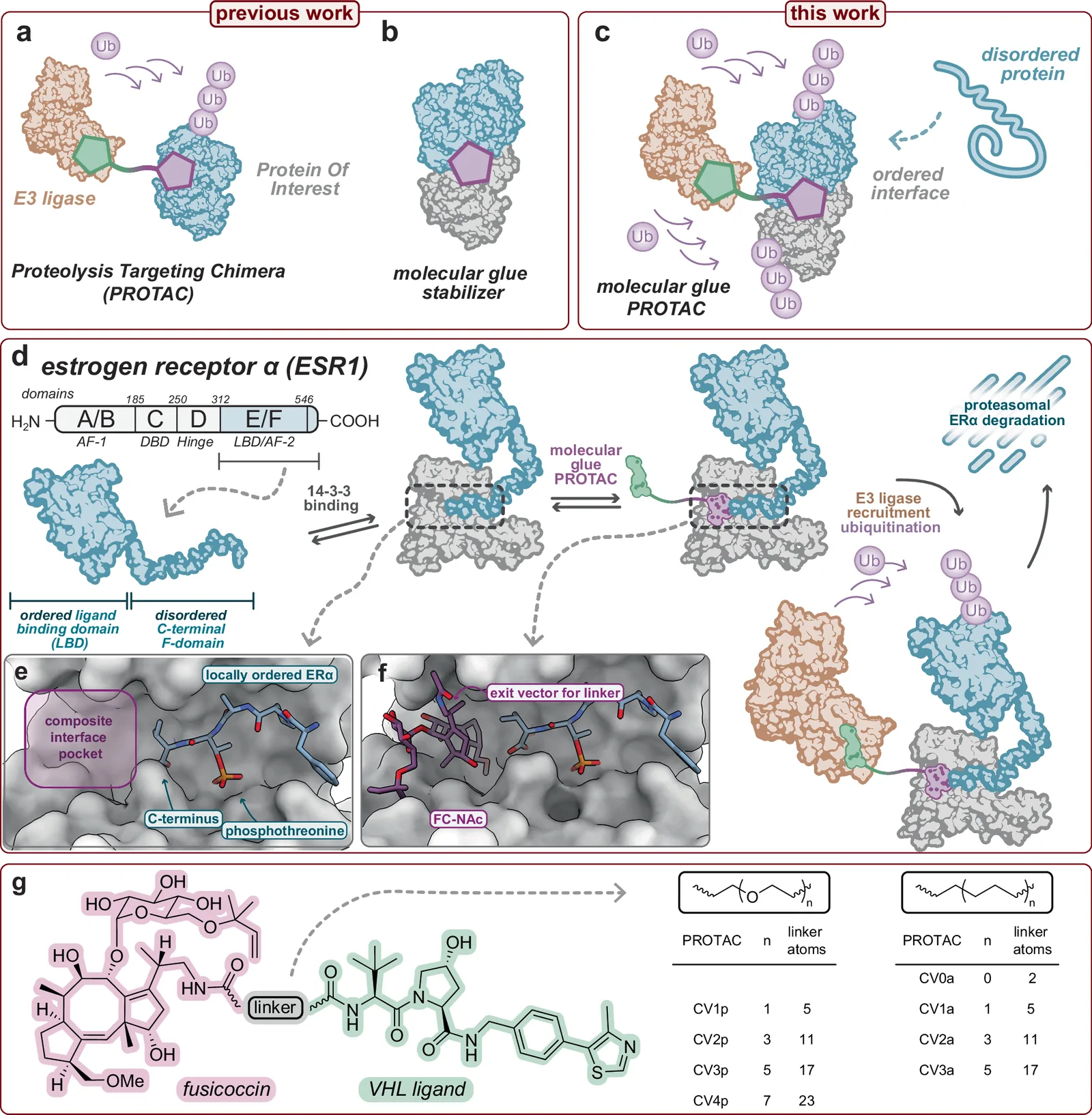
Concept and design of ^MG^PROTACs. **a**, **b** Schematic illustrates the difference between heterobifunctional PROTACs and monovalent molecular glue-induced proximity. **a** Depicts PROTAC (green/purple) induced labelling of a protein of interest (POI, blue) by a representative E3 ligase (light tan) with ubiquitin (Ub, light purple). **b** Depicts molecular glue (purple) stabilization of two representative proteins (blue and grey) that endogenously form a protein complex. **c** Illustration of our approach, which replaces a protein of interest ligand with a molecular glue stabilizer within a PROTAC to produce a molecular glue PROTAC (^MG^PROTAC) that can ubiquitinate a protein complex. Color pallet follows (**a** and **b**). **d** Schematic illustration of the ^MG^PROTAC (green/purple) directed VHL E3 ligase (light tan) labelling of the 14-3-3/ERα complex (shown in grey and blue, respectively) with ubiquitin (light purple). **e** Crystal structure of the binary 14-3-3/ERα complex reveals the composite interface and a pocket for small molecule stabilization (PDB: 7NFW). 14-3-3 and ERα F-domain peptide depicted in grey and blue, respectively. **f** Crystal structure of the ternary 14-3-3/ERα/FC-NAc complex, showing the exit vector for ^MG^PROTAC development (PDB: 8BZH). The 14-3-3/ERα complex depicted as in (**f**) with FC-NAc in purple. **g** Overview of the ^MG^PROTAC panel, featuring PEG and alkyl linkers.

Induced proximity has enabled precise chemical control over protein–protein interactions (PPIs) to elicit defined biochemical outcomes. Rather than simply inhibiting protein activity, small molecules that bring proteins together modulate function through processes such as PPI stabilization^1–3^, or signaling modulation^4^. Conventionally, this is achieved using either bifunctional molecules, e.g., proteolysis-targeting chimeras (PROTACs) that are composed of two separate protein-binding ligands, or monovalent small molecules termed “molecular glues” that typically bind to either of the two proteins to enhance their complex formation (Fig. 1a)^5^. The most prominently explored modality illustrating this concept is targeted protein degradation (TPD), where the ubiquitin-proteasome system is harnessed to chemically induce ubiquitination and subsequent proteolysis of a protein of interest^6^. Over 30 degrader molecules are currently in clinical evaluation^7–9^, with several degraders demonstrating efficacy against previously intractable targets such as KRAS^10^, STAT5^11^, and LRRK2^12^. Together, these advances highlight the broad potential of protein degradation as a powerful paradigm in drug discovery.

Beyond degradation, induced proximity strategies have expanded to include diverse heterobifunctional approaches that recruit alternative enzymatic machineries, such as kinases, phosphatases, deubiquitinases, and lysosomal components^1,13–17^. Non-enzymatic heterobifunctional strategies have also emerged, including regulated induced proximity targeting chimeras and transcriptional chemical inducers of proximity^1,17^. Together, these approaches modulate protein fate to rewire cellular networks^3,18,19^. The potential of molecular glue stabilization has also been demonstrated by Revolution Medicines’ zoldonrasib, which glues KRAS^G12D^ (ON) to CYPA^3^, and 14-3-3 glues that selectively target specific 14-3-3 complexes^2,18,20–24^. Collectively, these strategies vastly expand the chemical space for modulating protein behavior.

The efficacy of induced proximity often arises from the cooperative assembly of higher-order complexes^25^, in which activity depends on the formation and stability of multicomponent assemblies rather than on high-affinity ligand binding. Such cooperativity enhances potency and dictates selectivity through the emergent network of PPI contacts within the induced complex, as observed structurally and biophysically for both bifunctional^26–30^ and monovalent glue molecules^2,31–35^. However, most current strategies remain confined to protein targets bearing ligandable surfaces, while many clinically relevant proteins, such as RNA-binding proteins, transcription factors, and scaffolding proteins lack ligandable pockets or surfaces.

Here, we establish a framework for chemical controlled ubiquitination of a protein complex by integrating a molecular glue stabilizer as the target-recruiting ligand within a PROTAC design to develop a molecular glue PROTAC (^MG^PROTAC; Fig. 1c). In our system, the molecular glue functions as the ligand for a protein complex, stabilizing an interface between a partially disordered target and a scaffold protein, enabling the conditional recruitment of the complex to an E3 ligase. We demonstrate this concept by recruiting the von Hippel–Lindau (VHL) E3 ligase to a complex between the partially disordered Estrogen receptor α (ERα) and the hub protein 14-3-3. We found that the degree of cooperative VHL recruitment is strictly influenced by linker composition and length. Structural analysis using cryogenic electron microscopy (cryo-EM) reveals a de novo VHL/14-3-3 interface formed through induced hydrogen bonds and hydrophobic contacts. Recruitment requires both 14-3-3 and ERα, and promotes compound-dependent ubiquitination whose pattern and extent were modulated by tuning linker properties.

## Results and discussion

### Design and synthesis of 14-3-3/ERα complex ^MG^PROTACs

To explore small-molecule–induced degradation of a protein complex, we conjugated the 14-3-3/ERα molecular glue 3′-deacetylated fusicoccin N-acetyl (FC-NAc)^36^ to the VHL ligand VH032^37^ through a series of chemical linkers (Fig. 1d, e). ERα comprises both structured and intrinsically disordered regions. The N-terminal AF-1 domain, hinge region, and C-terminal F-domain existing as unstructured, whereas the DNA-binding domain and ligand-binding domain (LBD) adopt well-defined tertiary structures (Fig. 1d). FC-NAc enhances 14-3-3 binding to the disordered C-terminal F-domain of ERα, which undergoes a disorder-to-order transition upon 14-3-3 engagement with phosphorylated T594 (pT594), generating a composite interface stabilized by FC-NAc (Fig. 1e, f)^36,38^. ERα remains a clinically relevant target in ERα–positive breast cancer, where existing small molecules targeting the LBD are susceptible to resistance conferred by recurrent LBD mutations (e.g., Y537S, Y537N, D538G)^39^. Further, we selected VHL as the target E3 ligase given its extensive use as an E3 ligase ligand and tractability for biophysics and structural biology of ternary complexes, though recent advances in chemical warheads and structural enablement warrant future expansion to CRBN as well^40^. Taken together, this presented an interesting biomolecular case study to explore VHL recruitment and ubiquitination of a partially disordered 14-3-3 protein complex.

^MG^PROTAC design began with analysis of the crystal structure of the 14-3-3/ERα/FC-NAc complex (Fig. 1f) (PDB: 8BZH)^36^, which identified the amide modification site of FC-NAc as a suitable exit vector for PROTAC extension. As the acetyl group projects into the solvent, we hypothesized that this position would tolerate linker attachment without disrupting 14-3-3/ERα binding. Consistent with this rationale, FC-NAc binds the 14-3-3σ/ERα complex with high affinity (EC_50_ = 15 nM), substantially stronger than FC-A (EC_50_ = 421 nM)^36^. Furthermore, FC-NAc is chemically tractable, and previous studies demonstrate its tolerance to bulky substitutions at this position^36,41,42^. To optimize cooperative E3 ligase recruitment to the 14-3-3/ERα complex, an eight-member panel of FC-NAc–VH032 chimeras incorporating linker lengths ranging from 2 to 23 heavy atoms and two distinct chemical compositions—polyethylene glycol (PEG) or alkyl was synthesized (Fig. 1g). The ^MG^PROTAC panel was prepared via a six-step convergent synthesis (SI Scheme 1), starting from the fungal extract 3′-deacetylated fusicoccin-A. Notably, the final products showed a partial cleavage (5–43%) of the 2-methylbut-3-en-2-yl ether moiety on the fusicoccane ligand. Investigation of this gem-dimethyl ether cleavage revealed that the moiety is labile under the acidic conditions used during HPLC purification, with the highest extent of cleavage (43%) observed for CV0a. Importantly, this modification had a negligible impact on 14-3-3/ERα complex stabilization (Supplementary Fig. 1).

### ^MG^PROTACs stabilize the 14-3-3/ERα PPI and engage VHL in vitro and *in cellulo*

**Fig. 2:**
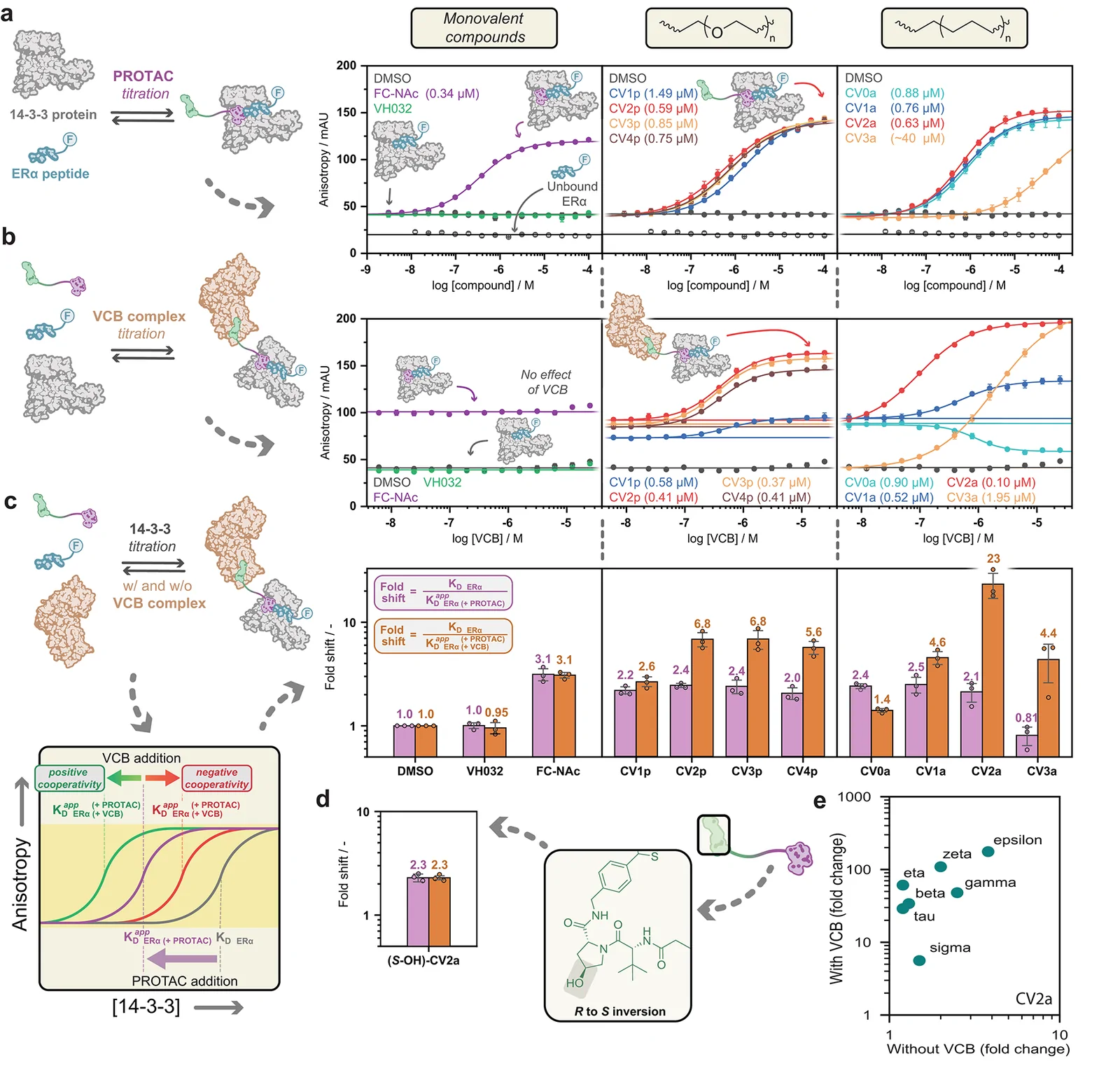
Stabilization of the 14-3-3/ERα protein complex and cooperative binding of VCB. **a** Titration of^MG^PROTAC (green/purple; or monovalent compound (purple)) to 10 nM fluorescently labeled ERα 8-mer phosphopeptide (blue) and 1 µM 14-3-3σ (grey) using a fluorescence anisotropy assay, with DMSO serving as negative control. EC_50_ values of binding curves are indicated. **b** Titration of the VCB protein complex (tan) to 10 nM fluorescently labeled ERα peptide (blue), 500 nM 14-3-3 (grey), and 1 µM PROTAC (green/purple; or monovalent compound (purple). EC_50_ values of binding curves are indicated. **c** Conceptual overview and  fold-stabilization of 14-3-3 protein titration to 10 nM fluorescently labeled ERα peptide, 1 µM PROTAC, and in the presence (5 µM, orange bars) or absence (purple bars) of VCB protein complex present in a bar chart. **d** Fold-stabilization of negative control compound (S-OH)-CV2 in the presence (5 µM, orange bars) or absence (purple bars) of VCB protein complex presented in a bar chart. **e** Scatter plot comparing CV2a stabilization of 14-3-3 isoforms beta, gamma, epsilon, zeta, eta, sigma, tau in the in the presence (5 µM) or absence of the VCB protein complex. Data presented in (**a**–**d**) are represented as mean values ± standard deviations of independent biological replicates (*n* = 3) and source data are provided in the Source Data file.

^MG^PROTAC stabilization of the binary 14-3-3/ERα complex (*K*_*D*_ = 2.32 µM) was then characterized by 14-3-3 titration in the presence of 1 µM ^MG^PROTAC (Supplementary Fig. 2). All compounds, except CV3a, enhanced ERα binding to 14-3-3, yielding lower apparent *K*_*D*_ (*K*_*D*_^app^) values of 0.96–1.14 µM (2.0–2.5-fold stabilization). Both linker series provided stabilization only marginally weaker than FC-NAc (*K*_*D*_^app^ = 0.75 µM, 3.1-fold stabilization), indicating that the ^MG^PROTAC retained molecular glue stabilization.

With the bifunctional panel in hand, we examined the biophysical properties of the ^MG^PROTACs, specifically the influence of linker length and hydrophobicity. ^MG^PROTAC binding and 14-3-3/ERα complex stabilization were assessed using a fluorescence anisotropy (FA) assay. To assess ^MG^PROTAC binding, each compound was titrated to a 14-3-3/ERα complex (1 µM 14-3-3σ and 10 nM ERα peptide; Fig. 2a). The monovalent molecular glue FC-NAc served as a positive control (EC₅₀ = 0.34 µM). Notably, in our hands, FC-NAc was less potent than previously reported. All ^MG^PROTACs, with the exception of CV3a, displayed dose-dependent binding (EC₅₀ = 0.59–1.49 µM; Fig. 2a), with CV2p exhibiting the highest apparent potency. In contrast, CV3a, which features a 17-atom aliphatic linker, showed markedly weaker binding (EC₅₀ ≈ 40 µM). Further, the VHL ligand VH032 did not engage the 14-3-3/ERα complex. These findings validated that the N-acetyl exit vector of FC-NAc was compatible with linker derivatization.

Next, we assessed ^MG^PROTAC engagement with the VHL/Elongin C/Elongin B (VCB) complex using two assays; an FA-based HIF1α displacement assay—the native VHL substrate—and an in cell VHL target engagement assay^43^. All ^MG^PROTACs displaced the HIF1α peptide in the FA assay (IC₅₀ ranged from 0.43 to 1.41 µM), except CV3a, which showed minimal displacement (IC₅₀ ≥ 100 µM) (Fig. S3a).

In cell VHL engagement was evaluated using a bioluminescence resonance energy transfer (BRET) assay in ERα-positive cells (MCF-7). Cells were transiently transfected with NanoLuc–VHL, treated with a fluorscent VHL probe and incubated with each compound (Promega, N2931). In permeabilized cells, the ^MG^PROTACs elicited an analogous trend to the FA-based HIF1α displacement assay (Supplementary Fig. 3b). All ^MG^PROTACs except CV3a displaced the probe with potencies comparable to VH032, exhibiting IC₅₀ values in the 0.3–1.3 µM range. In live MCF-7 cells, marked differences in cellular permeability were observed. ^MG^PROTACs bearing alkylic linkers showed improved cell penetration relative to their PEG-linked counterparts, with IC₅₀ values ranging from 1.0 to >10 µM versus >10 µM for PEG-linked analogues. Notable exceptions were CV0a and CV3a (Supplementary Fig. 3c). The poor engagement of CV0a (IC₅₀ > 10 µM) is likely due to its short linker, which increases overall polarity and limits membrane diffusion. In contrast, the reduced activity of CV3a (IC₅₀ = 1.4 µM) is hypothesized to arise from the hydrophobic nature of its long aliphatic linker, potentially compromising solubility and effective ligand engagement. Combined, the results of the FA assays and VHL target engagement experiments demonstrated that the ^MG^PROTACs effectively engaged both 14-3-3/ERα and VHL complexes.

### ^MG^PROTACs cooperatively recruit VHL to the 14-3-3/ERα complex

Recruitment of the VCB complex to the 14-3-3/ERα complex was then investigated using our established fluorescence anisotropy assay by titrating VCB to a fixed concentration of compound (1 µM), fluorescently labeled ERα peptide (10 nM), and 14-3-3 (500 nM). This 14-3-3 concentration corresponds to approximately 40% bound ERα peptide. In the presence of vehicle (DMSO), FC-NAc, or VH032, no response was elicited (Fig. 2b), indicating that VCB, either in its unbound or VH032-bound state, has no intrinsic affinity for the 14-3-3/ERα complex. In contrast, VCB titration in the presence of ^MG^PROTACs modulated ERα-associated anisotropy, consistent with quaternary complex formation. Structure–activity relationship (SAR) analysis revealed distinct linker-dependent effects on ^MG^PROTAC-induced VCB recruitment. PEG-based ^MG^PROTACs displayed moderate VCB recruitment across the series, yielding similar EC₅₀ values (0.37–0.59 μM), with the shortest linker (CV1p) producing the smallest anisotropy shift. The minimal response observed for CV1p suggests that the short PEG-based linker comprising five heavy atoms is insufficient to effectively recruit VCB to the 14-3-3/ERα complex, consistent with steric constraints. Beyond a linker length of 11 heavy atoms (CV2p), PEG-based linkers exerted limited additional influence on VCB recruitment.

In contrast, alkyl-linked ^MG^PROTACs exhibited greater diversity in cooperative VCB recruitment. The shortest alkyl-linked ^MG^PROTAC (CV0a; two heavy atoms) disrupted 14-3-3/ERα complex formation, indicative of negative cooperativity, likely reflecting steric limitations imposed by insufficient linker length, similar to CV1p. Extension of the alkyl linker (CV1a–CV3a) restored cooperative recruitment, with CV2a and CV3a (linkers of 11 and 17 heavy-atom, respectively) displaying the largest anisotropy shifts across both linker series, albeit with a ~ 20-fold difference in EC₅₀ values (0.10 and 1.95 μM, respectively; Fig. 2b). Notably, CV2a elicited increased anisotropy at very low VCB concentrations, below the intrinsic affinity of VH032 for VHL, indicative of highly cooperative complex formation relative to PEG-based and other alkyl-linked ^MG^PROTACs. In contrast, recruitment of VCB by CV3a was only observed at high VCB concentrations, consistent with its reduced stabilization activity. This is likely due to the hydrophobic linker promoting nonproductive conformations, thereby limiting effective engagement of the 14-3-3/ERα complex.

Having established that seven of the eight ^MG^PROTACs recruit VCB to the 14-3-3/ERα complex, we assessed glue-like enhancement of 14-3-3/ERα avidity. FA-based 14-3-3 titrations were performed in the presence of a ^MG^PROTAC (1 µM) and VCB (5 µM). At these concentrations, >95% of the ^MG^PROTAC is bound to the VCB complex, enabling the detection of cooperative interactions between the VCB/^MG^PROTAC complex and the 14-3-3/ERα complex. All ^MG^PROTACs stabilized the 14-3-3/ERα complex, except the negatively cooperative CV0a, consistent with the VCB titration assay (Fig. 2c and Supplementary Fig. 4). The greatest effects were observed for compounds with 11- and 17-atom linkers (CV2a and CV3a), showing 23- and 4.4-fold stabilization, respectively. A two-dimensional FA titration^25^ varying both the concentrations of CV2a and VCB revealed cooperative interplay between the ^MG^PROTAC and VCB (Supplementary Fig. 5a–d). CV2a titration in the absence of VCB dose-dependently enhanced ERα binding to 14-3-3 (up to 2.5-fold), which was further amplified in the presence of VCB, reaching over 23-fold enhancement at high CV2a and VCB concentrations. These findings show that VCB binding can promote an over 11-fold improvement in the apparent affinity (*K*_*D*_^app^) of ERα at 1 μM of CV2a.

To validate that the observed *K*_*D*_ shifts were due to VCB binding, we synthesized an epimer control of CV2a bearing inverted stereochemistry at the proline 4-position of the VHL ligand, designated as (*S*-OH)-CV2a, which disrupts VCB binding (Supplementary Fig. 5e, f)^44^. As expected, (*S*-OH)-CV2a stabilized the 14-3-3/ERα complex similarly to CV2a, but failed to recruit VCB (Fig. 2d and Supplementary Fig. 5f).

Finally, we examined VCB recruitment by CV2a across all seven human 14-3-3 isoforms using the previously described 14-3-3 titration. Although 14-3-3 isoforms share a highly conserved binding groove and engage ERα similarly, their peripheral regions differ, potentially influencing VCB interaction. To account for isoform-dependent differences in ERα affinity (*K*_*D*_ values ranging from 2 to 29 μM), we compared fold stabilization for different 14-3-3 isoforms following treatment with CV2a in the presence or absence of VCB (Fig. 2e and Supplementary Fig. 6). CV2a-mediated 14-3-3/ERα stabilization in the absence of VCB was relatively consistent across isoforms (1.2–3.8-fold), but VCB recruitment in the presence of CV2a varied substantially, ranging from 5.6- to 176-fold (Fig. 2e). The σ isoform showed the weakest VCB stabilization (5.6-fold); τ, β, γ, and η displayed moderate effects (29-, 34-, 48-, and 61-fold); while ζ and ε exhibited the strongest stabilization (109- and 176-fold, respectively).

These findings show that both linker length and chemical composition govern cooperative stabilization. Specifically, longer apolar linkers promote strong positive cooperativity up to an optimal length (CV2a, 11 atoms). The results also support formation of a de novo VCB–14-3-3/ERα interface, which is influenced by isoform-specific residues outside the conserved binding groove.

### Cooperative VHL recruitment extends to the 14-3-3/ERα^(LBD_F)^ protein–protein complex

**Fig. 3:**
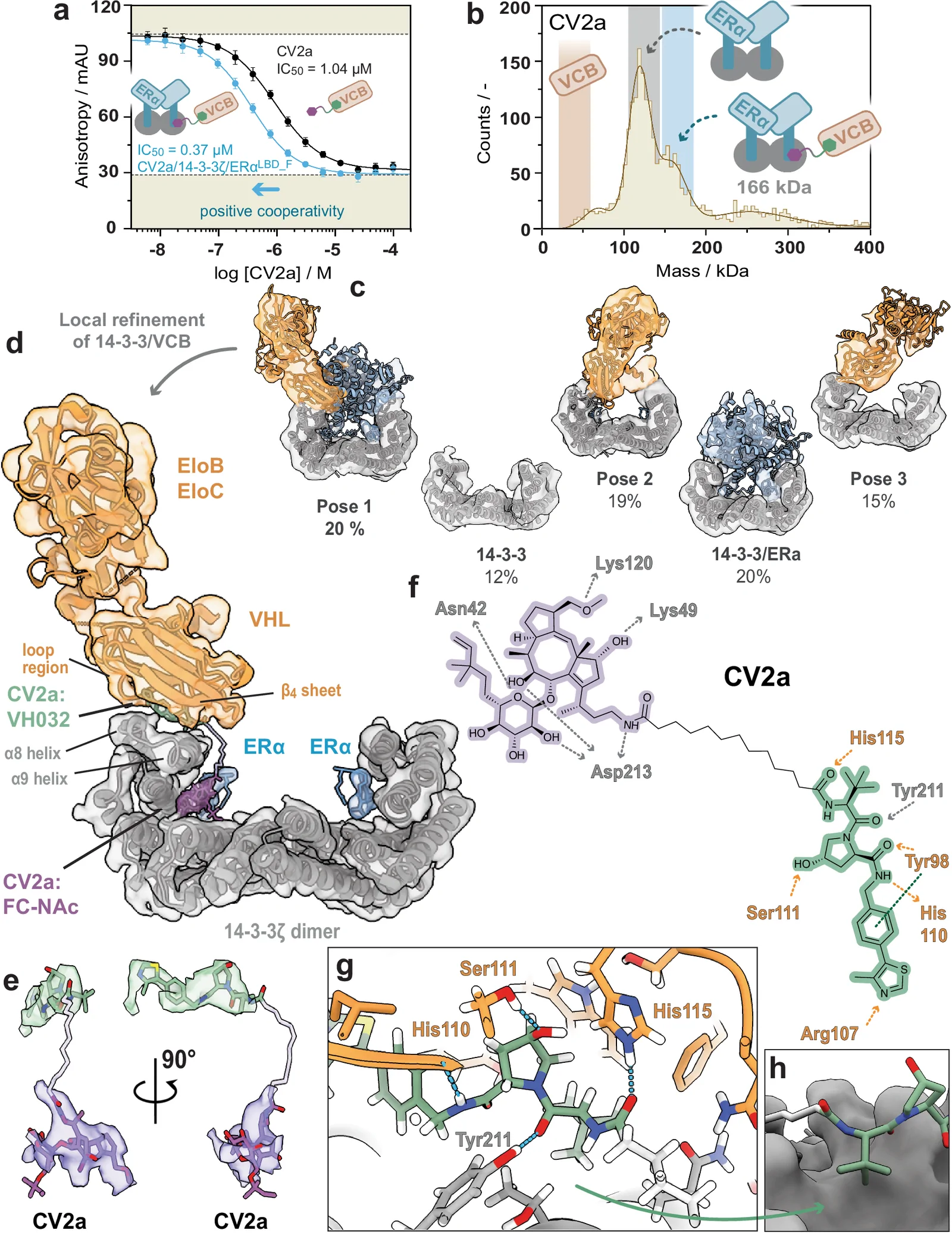
Structure of the VCB/CV2a/14-3-3ζ/ERα^(LBD_F)^ complex. **a** FA assay data of CV2a titration to 25 nM fluorescently labeled HIF1α peptide and 50 nM VCB protein complex in the absence (black) or presence of 10 µM 14-3-3/ERα^(LBD_F)^ complex (blue). Data are presented as mean values ± standard deviations of independent biological replicates (*n* = 3). **b** Mass photometry data of a 1:1 mixture of the 14-3-3/ERα^(LBD_F)^ complex (2:2 stoichiometry, 124 kDa) and the VCB complex (41 kDa) with three equivalents of CV2a. 14-3-3 (grey), ERα protein (blue), VCB complex (light tan), and ^MG^PROTAC (green/purple) schematically depicted. **c** 3D classes obtained displaying the conformational and compositional landscape of the sample. **d** Cryo-EM map and model of the best resolved VCB/CV2a/14-3-3ζ/ERα species showing a 1:1:2:2 stoichiometry. 14-3-3, ERα protein, VCB complex, and ^MG^PROTAC coloured grey, blue, light tan, and green/purple, respectively. **e** Local volume of the locally refined cryo-EM map corresponding to CV2a. Volumes represent electron density of the VH032 (green) and FC-NAc (purple) ligands. **f** Residues on VHL and 14-3-3, which were shown to form contacts with CV2a in the MD simulation. Hydrogen bonds are shown for VHL (orange) and 14-3-3 (grey). The pi-stacking interaction with VHL Tyr98 is shown in dark green. Arrow direction indicate hydrogen bond donor or acceptor. **g** Selected residues making contact with VH032; 14-3-3 (grey) and CV2a VHL ligand (green), and VHL (orange). **h** The tert-butyl group on the VH032 moiety of CV2a occupies a pocket on the 14-3-3 (grey) surface. Source data for panels a and b are provided in the Source Data file.

Using 14-3-3 and an ERα^(LBD_F)^ construct (Supplementary Fig. 7), which includes the LBD and disordered F domain containing phosphorylated T594, we assessed ^MG^PROTAC recruitment of the 14-3-3/ERα^(LBD_F)^ protein–protein complex to VCB using the HIF1α displacement assay (Fig. 3a and Supplementary Fig. 8a)^45^.

Cotreatment with the 14-3-3/ERα^(LBD_F)^ protein–protein complex and either CV2a or CV3a significantly enhanced displacement of HIF1α probe from VCB by the ^MG^PROTACs compared to ^MG^PROTAC alone (Fig. 3a and Supplementary Fig. 8a). This resulted in a 2.8-fold reduction in the IC₅₀ for HIF1α probe displacement (IC_50_ = 0.37 μM) for CV2a and *a* > 34-fold reduction (IC_50_ = 2.9 μM) for CV3a. In contrast, the negatively cooperative ^MG^PROTAC CV0a reduced HIF1α probe displacement in the presence of the complex, increasing the IC_50_ by fourfold to 5.4 μM. Together, this biophysical dataset is consistent with 14-3-3/ERα^(LBD_F)^/^MG^PROTAC protein complex engagement with VHL.

### Cooperativity is driven by favourable protein–protein contacts between 14-3-3 and VHL

To gain a structural insight into ^MG^PROTAC cooperativity, we turned to single-particle cryogenic electron microscopy (cryo-EM). Given the high cooperativity and low affinity observed for CV2a, this compound was selected for structural analysis. The multicomponent complex was prepared by incubating VCB, CV2a, and the 14-3-3ζ/ERα^(LBD_F)^ complex in a 1.2:1.2:1:1 molar ratio, followed by grid preparation without further purification. This molar ratio was selected to minimize hook effects and corresponds to a 1:2 ratio of ligand to available 14-3-3/ERα binding sites. Mass photometry analysis revealed a higher-molecular-weight species (~166 kDa), consistent with asymmetric binding of VCB to the 14-3-3ζ/ERα^(LBD_F)^ dimer-of-dimers assembly with a 2:2:1 14-3-3/ERα/VCB stoichiometry (Fig. 3b and Supplementary Fig. 8b). Cryo-EM analysis revealed a compositionally and conformationally heterogeneous population, with a single VCB complex recruited to the 14-3-3ζ dimer in multiple orientations (Fig. 3c and Supplementary Fig. 9). Although multiple cryo-EM poses are observed, all classes are interpreted as ERα-dependent ternary assemblies, with heterogeneity arising from the conformational flexibility of the ERα C-terminal region and LBD. Despite the heterogeneity in the cryo-EM sample, a major species corresponding to VCB positioned “in plane” with the 14-3-3ζ dimer was resolved to ~4.3 Å resolution, enabling confident model building of VCB and 14-3-3ζ (Fig. 3d and Supplementary Figs. 10–12). Although volume corresponding to the ERα LBD was present, the LBD was poorly defined (Fig. 3c; “Pose 1”) likely due to the high degree of flexibility conferred by the ERα F-domain, and only the final four residues of the phosphorylated C-terminal F-domain were modelled.

CV2a induces a neo-protein–protein interface between 14-3-3ζ and VHL (Fig. 3d), with the loop region and β4 sheet of VHL directly engaging the α8 and α9 helices of one 14-3-3ζ monomer. Well-defined density was observed for the VH032 and FC-NAc ligands of CV2a (Fig. 3e), which adopted binding modes consistent with the previously reported structures for VH032 bound to VHL and FC-NAc bound to the 14-3-3/ERα complex (Fig. 3f and Supplementary Fig. 13)^36,37^. Notably, only electron density corresponding to a single ^MG^PROTAC was observed, consistent with CV2a being present at approximately half the molar ratio relative to available 14-3-3/ERα binding sites. The 11-atom hydrocarbon linker of CV2a has no interpretable volume. We therefore performed energy minimization and 500 ns molecular dynamics (MD) simulation to explore plausible conformations and interactions of the complex (Supplementary Figs. 14 and 15). Specifically, we excluded the poorly resolved ERα LBD, opting to model the VCB complex, a single 14-3-3 monomer, and CV2a. The 14-3-3ζ/VHL/CV2a complex proved to be highly stable throughout the 500 ns simulation (Supplementary Movie 1). Superimposing the apo-14-3-3ζ AlphaFold structure with our cryo-EM model (Supplementary Fig. 16) revealed minimal backbone rearrangement. Analysis of the 14-3-3/VHL interface revealed rotation of Tyr211 of 14-3-3ζ to accommodate the VH032 ligand of CV2a, enabling formation of a putative hydrogen bond between phenolic hydrogen of Tyr211 and the carbonyl oxygen of the CV2a *tert*-leucine moiety (Fig. 3g). Further, the *tert*-leucine moiety of CV2a occupies a pre-existing pocket on 14-3-3ζ (Fig. 3h). Finally, Arg222 of 14-3-3ζ reorients to avoid steric clashes with VHL and engages a hydrogen bond with Asp67 of VHL. The protein residues involved in CV2a ^MG^PROTAC binding remained relatively stable throughout all MD replicates, as evidenced by the RMSF profiles of the Cα carbons (Supplementary Fig. 14a–c, green bars).

**Fig. 4:**
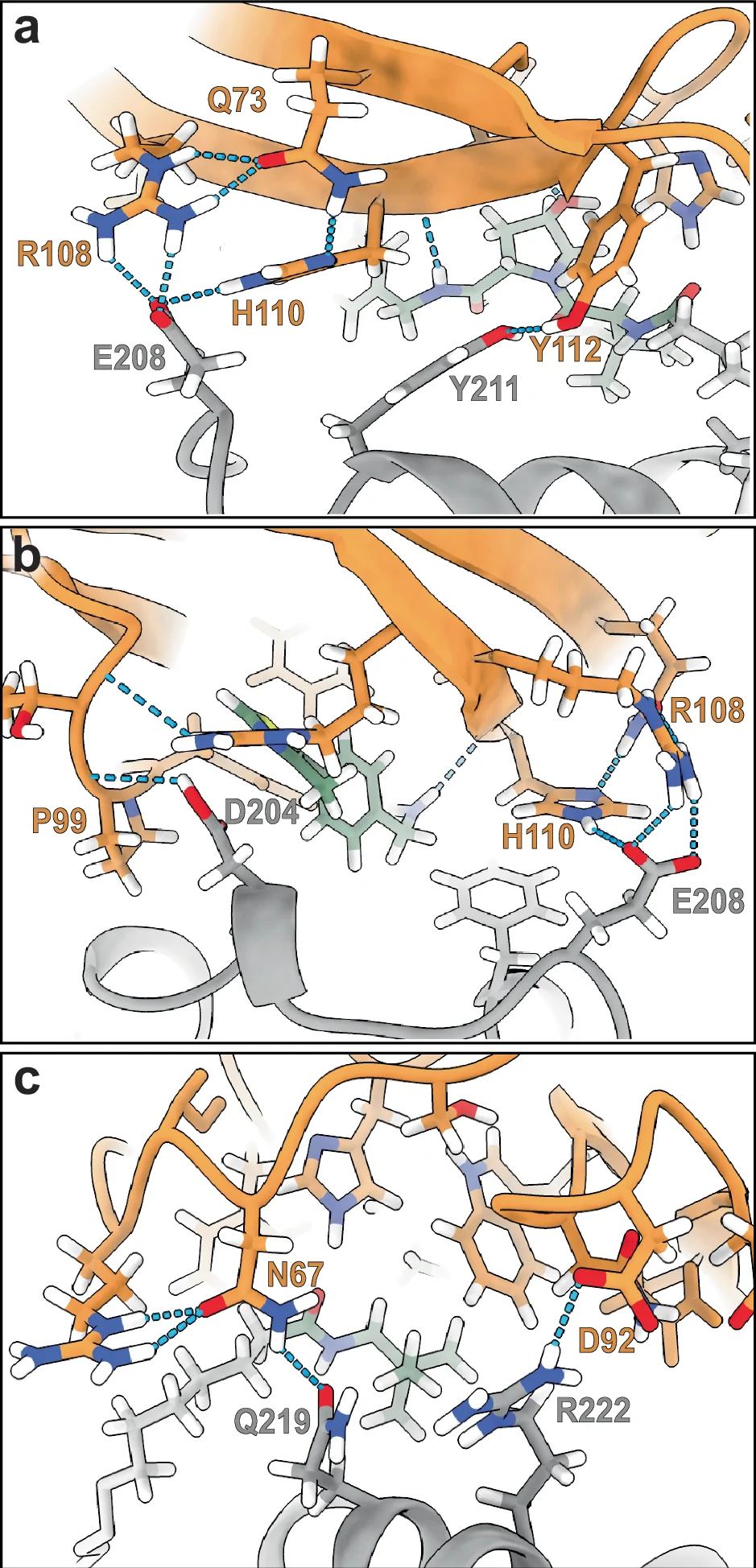
Protein-protein interactions between VHL and 14-3-3ζ identified by cryo-EM analysis and MD simulation. Enlarged views of the 14-3-3ζ/VHL interface. VHL is shown in orange and 14-3-3ζ in grey. **a** depicts hydrogen bonding between VHL Tyr112 and 14-3-3ζ Tyr211 on the α9 helix, with Tyr211 also hydrogen bonding to CV2a; **b** a dynamic hydrogen-bonding network involving VHL Arg108, Asn73, and His110 with 14-3-3ζ Glu208; and **c** hydrogen bonding between VHL Asn67 and 14-3-3ζ Gln219. Each view is rotated by 120° around the vertical axis.

The MD simulation identified a network of stabilizing inter-protein interactions (Supplementary Fig. 15). VHL Tyr112 forms a hydrogen bond with Tyr211 of 14-3-3ζ (α9 helix), which in turn forms a hydrogen bond with CV2a (Fig. 4a). This interaction proved to be highly stable throughout the 500 ns simulation (Supplementary Fig. 15c), suggesting this trivalent interaction functions as an anchor point. On the same β-sheet, VHL Arg108, Asn72, and His110 form a dynamic hydrogen-bonding network with 14-3-3ζ Glu208 (Fig. 4b). Visual inspection of the MD simulation over time showed that VHL Arg108 moves in and out of hydrogen-bonding distance with 14-3-3ζ Glu208 (Supplementary Movie 2 and Supplementary Fig. 15b). When VHL Arg108 moves out of phase, VHL Gln73 moves in to form a hydrogen bond with 14-3-3ζ Glu208. Towards the CV2a alkylic exit point on the VHL-binding moiety, we observed that VHL Asn67 and 14-3-3ζ Gln219 form a hydrogen bond (Fig. 4c). Further along the α9 helix, 14-3-3ζ Arg222 forms a hydrogen bond with the carboxylate group of Asp92 of VHL. Sequence alignment across the seven human 14-3-3 isoforms (β, ε, η, γ, σ, θ, ζ) showed high conservation of the previously described interface residues (Supplementary Fig. 17). Notably, Asp204 of 14-3-3ζ, which hydrogen bonds with Pro99 of VHL, is substituted by His in 14-3-3σ—introducing potential electrostatic repulsion with nearby VHL Arg107 and providing a structural rationale for the reduced cooperativity observed for 14-3-3σ. Taken together, these analyses suggest that the hydrogen bond between VHL Tyr112 and 14-3-3ζ Tyr211 functions as the key hotspot interaction, given its stability over time around short pairwise distances of <5 Å, while the additional interactions support protein–protein contacts that are likely more transient in nature.

Together, our cryo-EM and MD analyses provide structural context for the biophysical data, illustrating favourable interprotein contacts between the VHL β-domain and the 14-3-3ζ α8/α9 helices induced by the VHL-recruiting moiety of the ^MG^PROTAC. In addition, MD simulations of CV2a shows that its linker adopts an extended conformation compatible with simultaneous engagement of the VH032 and FC-NAc binding sites, whereas shorter linkers (e.g., CV0a and CV1a; 2 and 5 atoms, respectively) are less able to span this distance. These observations are consistent with the structure–activity relationships between linker length and cooperative complex formation.

### VHL recruitment to 14-3-3 is driven by ERα

**Fig. 5:**
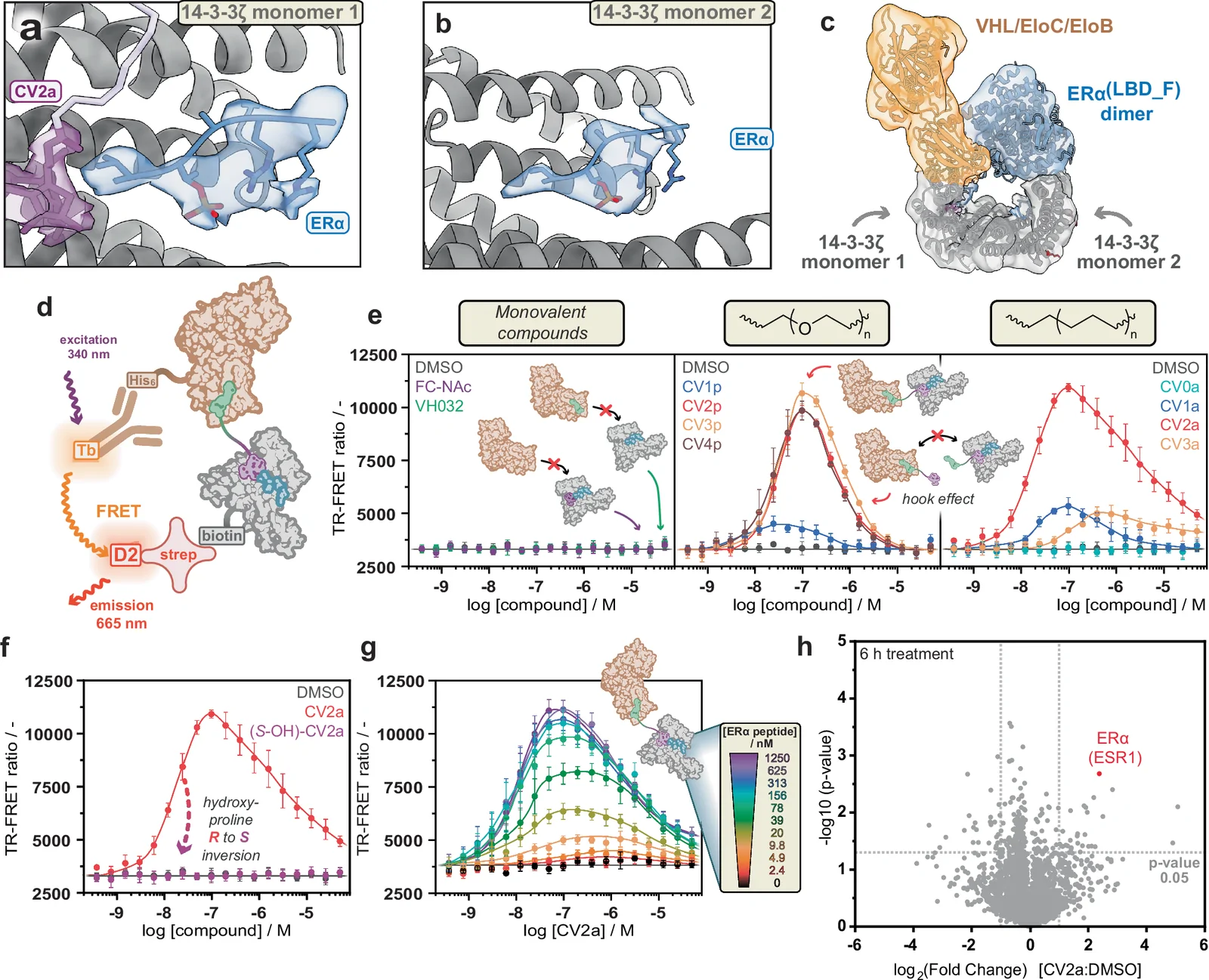
ERα drives quaternary complex formation. **a** Locally refined cryo-EM map from this study showing the volume around the ERα (blue) C-terminus associated with 14-3-3ζ (grey) monomer 1, and the FC-NAc ligand (purple) of CV2a. **b** Monomer 2 of the 14-3-3 dimer showing 14-3-3 (grey) and ERα (blue) C-terminus. **c** Unsharpened and Gaussian-smoothed cryo-EM map from this study showing volume corresponding to the ERα LBD domain (blue), bound to 14-3-3 (grey) and the VCB complex (light tan). **d** Schematic of the TR-FRET assay setup. **e** ^MG^PROTAC (green/purple; or monovalent compound (purple)) titration to 20 µM unlabeled ERα (blue) peptide, 50 nM His-tagged VCB complex (light tan), 50 nM biotinylated 14-3-3γ (grey), 0.5 nM anti-His Tb-conjugated mAb (light brown) and 12.5 nM streptavidin D2-conjugate (red) using a TR-FRET assay. **f** Comparison between the CV2a (red) and (*S*-OH)-CV2a (purple) generated TR-FRET signal. **g** Titration of unlabeled ERα and CV2a. **h** 14-3-3 IP–MS data of MCF-7 cells CV2a-treated for 6 h. Data presented in (**e**–**h**) are represented as mean values ± standard deviations of independent biological replicates (*n* = 3) and source data are provided in the Source Data file.

Structural analysis of ERα within the complex proved more challenging due to the conformational heterogeneity of ERα, which prevented the reconstruction of a single stable high-resolution structure. Nonetheless, structural analysis revealed that ERα engages with both monomers of the 14-3-3 dimer, as volume corresponding to the phosphorylated F-domain was observed within both binding grooves of the 14-3-3 dimer(Fig. 5a, b and Supplementary Fig. 18). This observation is consistent with previously published 14-3-3/ERα and 14-3-3/ERα/FC-NAc crystal structures (Fig. 1e, f) and in solution data^36,45^. Moreover, additional density was detected near helices α8 and α9 of monomer 2 in 14-3-3ζ, likely corresponding to the ERα LBD (Fig. 5c).

To directly assess recruitment of the VCB complex to 14-3-3, we optimized a time-resolved Förster resonance energy transfer (TR-FRET) assay (Supplementary Fig. 19), employing unlabeled ERα peptide, His-tagged VCB complex, and biotinylated 14-3-3γ (Fig. 5d). An analogous correlation between linker properties and VCB recruitment was observed in the FA and TR-FRET assays (Fig. 5). Notably, CV2a exhibited an extended recruitment window that persisted to higher compound concentrations compared with the PEG series, consistent with its higher cooperativity delaying the onset of the hook effect. In contrast, CV3a—although classified as cooperative—showed markedly reduced recruitment. This lack of induced proximity likely arises from the weaker intrinsic affinity of CV3a for both 14-3-3ζ/ERα and VCB at nanomolar concentrations. Further, the control compound (S-OH)-CV2a elicited no TR-FRET signal, indicating that induced proximity was directly mediated by the VHL ligand (Fig. 5f). To examine ERα dependence, a two-dimensional titration of ERα and CV2a was performed (Fig. 5g). VCB recruitment was strictly dependent on ERα concentration, with efficient VCB recruitment observed above 10 nM ERα. This biophysical behaviour is consistent with the proposed mechanism in which 14-3-3 acts as a scaffold to mediate E3 ligase recruitment to ERα. This result validates our interpretation that all cryo-EM classes are ERα-dependent ternary assemblies. Together, these data show that VCB recruitment is ERα-dependent, eliciting high selectivity for the 14-3-3/ERα complex over *apo*-14-3-3.

### CV2a-dependent VHL recruitment drives ubiquitination of the 14-3-3ζ/ERα^(LBD_F)^ complex

Having established robust ^MG^PROTAC-mediated recruitment of VHL to the 14-3-3/ERα complex, we next assessed degradation of ERα in ERα-positive MCF-7 breast cancer cells. MCF-7 cells were cultured in hormone-free medium and treated for 24 h with vehicle (DMSO), fulvestrant (ICI, 1 µM)—a known ERα degrader—or increasing concentrations of FC-NAc or CV2a (0.3–30 µM). Levels of T594-phosphorylated ERα (pERα) were analysed using a pT594-specific antibody. As expected, DMSO and FC-NAc treatments showed no reduction in pERα levels, whereas ICI induced complete degradation of pERα (Supplementary Fig. 20a, b)^36^. However, CV2a failed to reduce pERα levels.

Given the absence of cellular degradation observed with CV2a, we performed 14-3-3 immunoprecipitation–mass spectrometry (IP–MS) experiments in MCF-7 cells to determine whether CV2a promotes enrichment of the 14-3-3/ERα complex in cells (Supplementary Fig. 20c, d). Briefly, 14-3-3 IP–MS provides a snapshot of the cellular 14-3-3 interactome and enables assessment of how compound treatment influences 14-3-3 partner binding. As most 14-3-3 client proteins are phosphorylated, this approach primarily enables detection of phosphorylated proteins, such as phosphorylated ERα (pERα).

To assess CV2a enrichment of ERα, MCF-7 cells were cultured and treated with either DMSO (0.1% (v/v)) or 10 μM CV2a for 6 or 24 h. Endogenous 14-3-3 was then pulled down using a pan-14-3-3 antibody coupled to magnetic beads. The resulting pulldown was then subjected to washing, on-bead digestion, and LC-MS/MS analysis. IP–MS analysis following CV2a treatment revealed enrichment of the 14-3-3/ERα interaction, and several other 14-3-3 interactions. At 6 h, ERα was enriched ~fivefold in the 14-3-3 pull-down upon CV2a treatment, with the strongest statistical significance among detected proteins (Fig. 5h). At 24 h, ERα enrichment decreased to ~2.5-fold and was no longer statistically significant (*p* > 0.05; Fig. S20c, d). This finding indicates that while CV2a does not degrade ERα, it effectively engages and stabilizes the 14-3-3/ERα complex in MCF-7 cells, and several other 14-3-3 interactors. This observation is consistent with previous proteomics work using FC-A and its semisynthetic derivatives, which demonstrated that FC-NAc induces polypharmacological perturbation of the 14-3-3 interactome^46,47^.

**Fig. 6:**
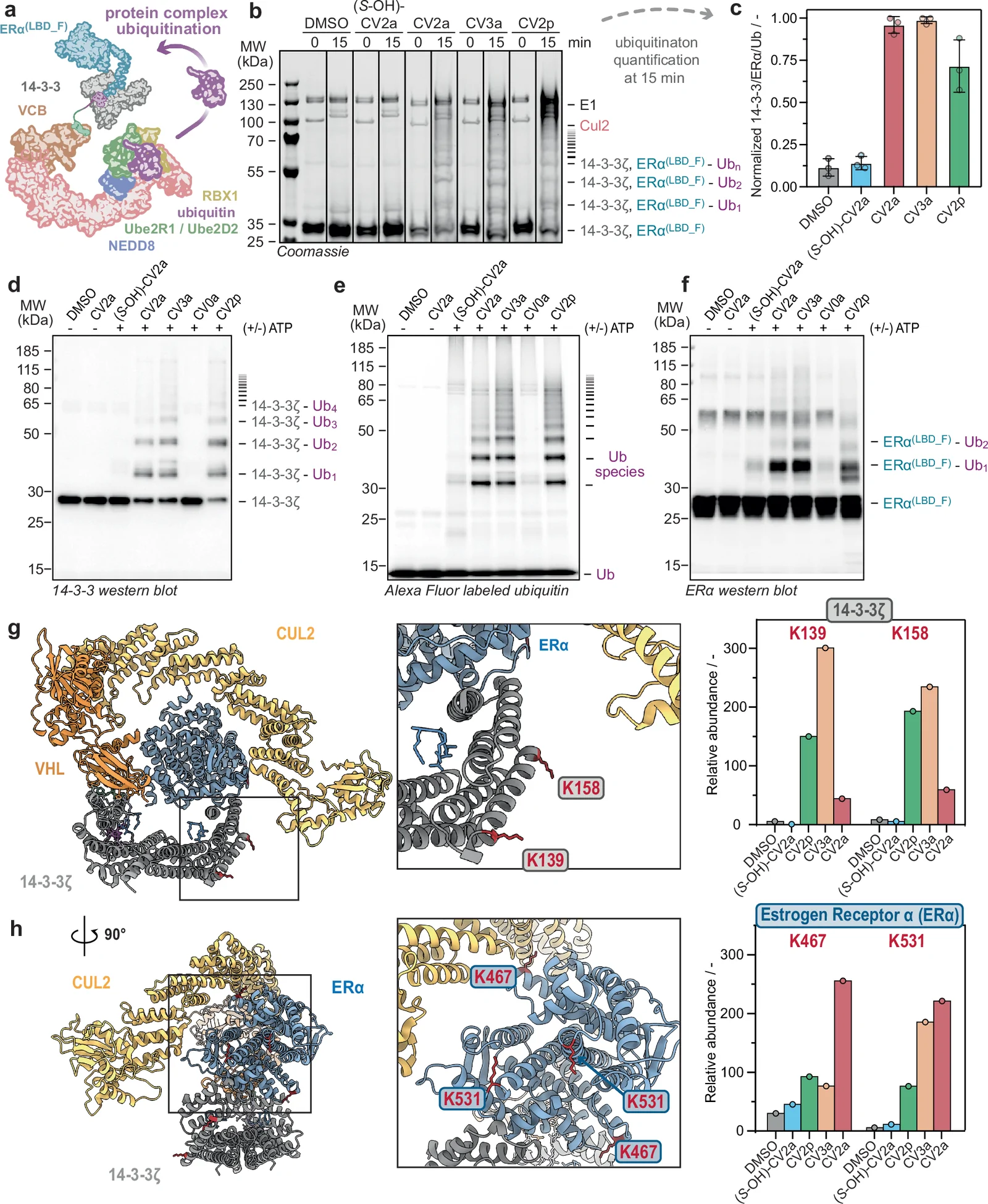
Ubiquitination of the 14-3-3/ERα complex. **a** Schematic of the ubiquitination reaction. 14-3-3 (grey), ERα^(LBD-F)^ (blue), ^MG^PROTAC (green/purple), ubiquitin (purple), VCB complex (light tan), Cullin 2 (pink), NEDD8 (violet), RBX1 (yellow), and UBE2R1/UBE2D2 (green). **b** In vitro ubiquitination reaction results over time visualized by SDS-PAGE and Coomassie staining, extended timepoints in Supplementry Fig. 14. **c** Quantification of in vitro ubiquitination at 15 min. Data are presented as mean values ± standard deviations of independent biological replicates (*n* = 3). **d** 14-3-3 western blot, **e** in-gel Alexa Fluor 488 fluorescence of labeled ubiquitin, and **f** ERα western blot of in vitro ubiquitination assay at 24 h. **d**–**f** are representative images of 2 independent biological replicates. Mass spectrometry results after 15 min incubation indicate two ubiquitination sites on 14-3-3 (**g**) and two on ERα (**h**). 14-3-3 (grey), ERα^(LBD-F)^ (blue), VCB complex (light tan), Cullin 2 (yellow). Labelled lysine shown in red on 14-3-3 (grey) and ERα^(LBD-F)^ (blue). Only DiGly peptides with peptide spectrum match (PSM)>10 is shown. An overview of all detected peptides is presented in SI Supplementary Table 1. Source data presented for (**c**–**h**) are provided in the Source Data file.

As the in-cell VHL target engagement assay and 6h 14-3-3 IP–MS showed significant VHL engagement and enrichment of the 14-3-3/ERα complex, respectively, we next examined whether the 14-3-3ζ/ERα^(LBD_F)^ complex undergoes ubiquitination in vitro as a preceding mechanistic step. Briefly, the 14-3-3ζ/ERα^(LBD_F)^ complex was incubated with recombinantly expressed and purified UBE1, UBE2D2, UBE2R1, CRL2^VHL^ and ubiquitin in the presence of ^MG^PROTACs and analysed time-dependently using Coomassie-stained SDS–PAGE (Fig. 6a). Ubiquitin conjugation was rapid with higher molecular weight species evident within 5 min (Supplementary Fig. 21) and were quantified at 15 min by Coomassie-stained SDS–PAGE (Fig. 6b, c and Supplementary Fig. 22). No ubiquitination was seen in the DMSO control, consistent with ubiquitination being contingent on induced proximity. By contrast, CV2p, CV3a, and CV2a each promoted robust polyubiquitination of the 14-3-3ζ/ERα^(LBD_F)^ complex, with modification levels correlating with the degree of VCB cooperativity measured in biophysical assays. CV2a and CV3a produced the strongest effects, whereas stereochemical inversion of CV2a ((*S*–OH)-CV2a) completely abrogated ubiquitination, confirming dependence on cooperative ^MG^PROTAC–VCB engagement. Given 14-3-3ζ and ERα^(LBD_F)^ migrate at similar apparent molecular weights by SDS-PAGE, ATP-dependent and ^MG^PROTAC-dependent ubiquitination of both proteins was validated by western blot analysis and using Alexa Fluor 488-labelled ubiquitin (Fig. 6d–f and Supplementary Fig. 23).

To define E2 requirements, we compared UBE2D2 and UBE2R1 using CV2p as a characteristic moderately cooperative ^MG^PROTAC. UBE2D2 was selected as a representative of the promiscuous UBE2D family which is known to work with Cullin RING E3 ligases such as CRL2^VHL^^48–52^. The UBE2R family is known to be more apt at extending polyubiquitin chains^49–51^, however recent studies have shown that UBE2R1 and UBE2R2 are also capable of installing the first ubiquitin on degrader-targeted substrates with CRL2^VHL^^53,54^. In this system, efficient ubiquitination of the 14-3-3ζ/ERα^(LBD_F)^ complex was observed only with UBE2D2 (Supplementary Fig. 24), whereas UBE2R1 failed to promote detectable substrate labelling under the same conditions. Furthermore, CV2p induced preferential ubiquitination of the assembled 14-3-3ζ/ERα^(LBD_F)^ complex over apo-14-3-3, with the latter showing less modification even after 24 h of incubation.

### ^MG^PROTAC design controls ubiquitination sites within the protein complex

To define site selectivity, we mapped ubiquitination sites by mass spectrometry after in vitro ubiquitination and proteolytic digestion of the 14-3-3ζ/ERα^(LBD_F)^ assembly. Ubiquitination was detected at Lys139 and Lys158 of 14-3-3ζ and at Lys467 and Lys531 of ERα^(LBD_F)^; a modification at residue 253 of the 14-3-3 construct was excluded because it resides in the protease cleavage scar and is absent from the native sequence. Only peptides with PSM > 10 were considered; a full peptide list is provided in Supplementary Table 1. To place these sites in structural context, we aligned VHL–EloC–EloB from our cryo-EM model with a previously reported VHL–EloC–EloB–Cul2–Rbx1–UBE2R1–Ub structure (Fig. 6g, h). In this overlay, the Cullin scaffold projected from the VHL–EloC–EloB module bound to 14-3-3ζ monomer 1 toward 14-3-3ζ monomer 2, orienting over the top of the 14-3-3ζ/ERα^(LBD_F)^ complex. This places the Cullin C-terminus proximal to Lys139 and Lys158 of 14-3-3ζ monomer 2 (Fig. 6g, left). Complementary AlphaFold modelling of the ERα^(LBD_F)^ dimer positioned Lys467 and Lys531 of ERα^(LBD_F)^ on the same face of the dimer, oriented toward the Cullin C-terminus, supporting a geometry in which these ERα lysines are accessible to ubiquitin transfer (Fig. 6g, right).

Comparison with our biochemical data showed that high cooperativity correlated with increased ubiquitination levels and that ^MG^PROTAC linker chemistry directed site selectivity (Supplementary Fig. 25). CV3a biased modification towards 14-3-3ζ, whereas the most cooperative ^MG^PROTAC, CV2a, preferentially targeted ERα^(LBD_F)^. This increased labelling of ERα may arise from cooperative contacts between ERα and VHL within the VCB module (Fig. 5c); however, that region of the cryo-EM map is poorly resolved, precluding in-depth structural analysis.

Together, these observations indicate that tuning linker length and composition orients the ligase–substrate assembly and can favour ubiquitination of one protein within the complex over the other.

In conclusion, this study establishes a structural and mechanistic framework for chemically induced ubiquitination of targeted protein complexes. By linking a molecular glue stabilizer ligand—which recruits a disordered region of the ERα target protein to 14-3-3—with an E3 ligase ligand (VHL), we demonstrate that induced proximity can be extended beyond single, well-folded proteins. Furthermore, we show that tuning the linker length and composition modulates ^MG^PROTAC cooperativity, E3 ligase recruitment, and site-selective ubiquitination efficiency. Cryo-EM analysis of the most cooperative ^MG^PROTAC, CV2a, revealed a de novo interface between VHL and 14-3-3ζ mediated by the α8/α9 helices of 14-3-3ζ—regions previously implicated in endogenous complexes such as BRAF/MEK1/14-3-3^55^, LRRK2/14-3-3^56^, HSPB6/14-3-3^57^, and KRAS/BRAF/MEK1/14-3-3^58^. These helices have also been reported to form secondary binding sites for small-molecule fragments^59^. Uniquely, the interaction described here represents an unprecedented ligand-induced interface between a 14-3-3:client complex and a neo-substrate, illustrating an additional cooperativity driven by the bifunctional molecule. This cooperative engagement between 14-3-3ζ and VHL, established from our biophysical data, explains the VHL-dependent enhancement of 14-3-3/ERα stabilization observed in vitro. Crucially, we find that cooperative formation of stable complexes is conducive to productive and efficient protein ubiquitination at specific lysine residues, including on ERα at its globular domain far from the disordered region directly recruited to the E3 ligase by our ^MG^PROTAC. Lysine ubiquitination preference is fine-tuned depending on the chemical nature of the compound inducing proximity, demonstrating that ^MG^PROTACs can selectively ubiquitinate specific protein species within a biomolecular complex.

Despite efficient ubiquitination of both 14-3-3ζ and ERα^(LBD_F)^ in biochemical assays and CV2a engagement of VHL and the 14-3-3/ERα complex, no measurable reduction in ERα levels was detected in cells. Proteomic studies with FC-A and its semisynthetic analogues have been shown to elicit polypharmacological effects, specifically, stabilizing several 14-3-3 interactions^46,47^, which our findings support. Such multi-complex engagement could dilute effective ternary complex formation with ERα and reduce degradation efficiency of any single client protein. Alternatively, the lifetime of the quaternary complex is too short under the cellular conditions to induce sufficient ubiquitination and may require improved biophysical properties (cooperativity and/or affinity) or physical chemistry properties. Recent work has highlighted the role of the efflux pump ABCC1 in PROTAC degradation efficiency^60^. Together, these constraints potentially restrict cellular degradation activity and explain why total ERα levels were unchanged even with the best compounds.

This study should be regarded as a proof-of-concept, rather than a fully validated chemical tool for studying ERα biology. Future focus on highly abundant 14-3-3-interacting phosphoproteins, or protein interactions beyond 14-3-3 complexes, could enhance the probability to observe induced ubiquitination translating into degradation. Further, investigating alternative E3 ligases, such as Cereblon or other Cullin RING ligases, offers an exciting opportunity to enable degradation given the high-quality ligands available^61,62^. CRBN ligands are an attractive alternative, with Arvinas’ ERα PROTAC vepdegestrant having entered phase III clinical trials for advanced breast cancer^63^. Notably, constitutive photomorphogenic 1 (COP1) has been reported to ubiquitinate and promote the degradation of 14-3-3σ^64^. However, the lack of small molecule binders complicates ligand development.

Future work should focus on translating this proof-of-concept strategy toward more drug-like molecules. To this end, moving beyond the semisynthetic natural product FC-NAc toward fully synthetic, lower-molecular-weight molecular glue scaffolds will be essential. For example, the synthetic 14-3-3/ERα molecular glue stabilizer 181 provides a promising starting point^65^. A notabl challenge with this compound is that it is a covalent molecular glue stabilizer. In parallel, optimization of linker architecture and E3 ligase ligands—through reduction of hydrogen-bond donors, increased molecular rigidity, and minimization of polar surface area—should improve overall physicochemical properties and potential efflux liabilities.

The findings shown here expand the conceptual drug discovery scope of induced proximity approaches by recruiting a protein complex for induced ubiquitination through a hybrid molecular glue PROTAC strategy. By demonstrating that degrader architecture can tune both the extent and topology of induced ubiquitination through cooperative interface formation. This work provides a blueprint for designing ^MG^PROTACs capable of reprogramming the ubiquitin–proteasome system toward protein assemblies composed of dynamic and disordered regions previously considered undruggable.

## Methods

### Protein expression and purification

#### 14-3-3 proteins

N-terminally His_6_-tagged 14-3-3 proteins (sigma isoform residues 1–248, beta isoform residues 1–246, gamma isoform residues 1–247, epsilon isoform residues 1–255, zeta isoform residues 1–245, eta isoform residues 1–246, sigma isoform residues 1–248, tau isoform residues 1–245) were transformed into *Escherichia coli* BL21(DE3) and cells were grown overnight in lysogeny broth (LB) supplemented with ampicillin (100 µg mL^−1^) at 37 °C with shaking^66^. The overnight culture was diluted 1:100 in LB supplemented with ampicillin (100 µg mL^−1^) and grown to an optical density (OD_600_) of ~1.2. Protein expression was induced overnight at 18 °C with 0.5 mM isopropyl β-d-1-thiogalactopyranoside (IPTG). The cells were harvested by centrifugation and frozen at −80 °C as pellets until further purification. The bacterial pellets were resuspended in buffer (50 mM HEPES, 300 mM NaCl, 12.5 mM imidazole, 2 mM β-mercaptoethanol, pH 8.0) supplemented with 5 mM MgCl_2_, benzonase (5 μL/100 mL), and 1× EDTA-free Roche protease inhibitor cocktail, and lysed by cell disruption using a C3 Emulsiflex-C3 homogenizer (Avestin) operating at 4 °C. Cellular debris was removed by centrifugation. The His_6_-tagged proteins were purified on a Ni-NTA Superflow cardridge (Qiagen) and eluted with 250 mM imidazole. The proteins were dialyzed into 25 mM HEPES, 100 mM NaCl, 10 mM MgCl_2_, 0.5 mM TCEP, pH 7.5, concentrated, flash frozen with liquid nitrogen, and stored at −80 °C.

N-terminally biotinylated 14-3-3 protein (gamma isoform, residues 1–247) was prepared by BirA enzyme co-expression (pETDUet-1 vector, protein insert sequences presented in Source Data file). In short, single colonies of His-AVI-14-3-3 and BirA cDNA were transformed into *E. coli* BL21(DE3) cells and grown overnight in LB supplemented with ampicillin (100 µg mL^−1^) at 37 °C with shaking. The overnight culture was diluted 1:100 in LB supplemented with ampicillin (100 µg mL^−1^) and biotin (500 µM) and grown to an optical density (OD_600_) of ~1.2. Protein expression was induced overnight at 18 °C with 0.5 mMl IPTG. The cells were harvested by centrifugation and frozen at −80 °C as pellets until further purification. The bacterial pellets were resuspended in buffer (50 mM HEPES, 300 mM NaCl, 12.5 mM imidazole, 2 mM β-mercaptoethanol, pH 8.0) supplemented with 5 mM MgCl_2_, benzonase (5 μL/100 mL), and 1× EDTA-free Roche protease inhibitor cocktail, and lysed by cell disruption using a C3 Emulsiflex-C3 homogenizer (Avestin) operating at 4 °C. Cellular debris was removed by centrifugation. The His_6_-tagged proteins were purified on a Ni-NTA Superflow cardridge (Qiagen) and eluted with 250 mM imidazole. The protein was dialyzed into a low-concentration imidazole buffer, incubated with Tobacco Etch Virus (TEV) protease, and flowed through a Ni-NTA Superflow cardridge (Qiagen). The protein sample was finally purified by size exclusion chromatography on a HiLoad 16/600 Superdex 75 pg column (Cytiva) in 25 mM HEPES, 100 mM NaCl, 10 mM MgCl_2_, 0.5 mM TCEP, pH 7.5, concentrated, flash frozen with liquid nitrogen, and stored at −80 °C.

#### 14-3-3ζ/ERα^(LBD_F)^ complex

The 14-3-3ζ/ERα^(LBD_F)^ protein complex (14-3-3ζ residues 1–245, ERα residues 302-595 with F591R and P592R mutations to make the 14-3-3 binding epitope PKA responsive and S305A mutation to block phosphorylation of this residue) was produced by co-transformation of His_6_-SUMO-ERα, 14-3-3ζ-strep and SUMO-PKA cDNA in *E. coli* NiCo21 (DE3) cells^45^. The cells were then grown overnight in LB supplemented with ampicillin (100 µg mL^−1^), streptomycin (100 µg mL^−1^), and chloramphenicol (25 µg mL^−1^) at 37 °C with shaking. The overnight culture was diluted 1:100 in Zyp-5052 medium supplemented with ampicillin (100 µg mL^−1^), streptomycin (100 µg mL^−1^), and chloramphenicol (25 µg mL^−1^) and and incubated at 37 °C, 180 rpm until an OD600 of ∼2 was reached. Protein expression was induced overnight at 18 °C with 0.5 mM IPTG. The cells were harvested by centrifugation and frozen at −80 °C as pellets until further purification. The bacterial pellets were resuspended in buffer (20 mM Tris, 300 mM NaCl, 12.5 mM imidazole, 5% (v/v) glycerol, 2 mM β-mercaptoethanol, pH 8.0) supplemented with 5 mM MgCl_2_, benzonase (5 μL/100 mL), and 1× EDTA-free Roche protease inhibitor cocktail, and lysed by cell disruption using a C3 Emulsiflex-C3 homogenizer (Avestin) operating at 4 °C. Cellular debris was removed by centrifugation. The His_6_-tagged protein complex was purified on a Ni-NTA Superflow cartridge (Qiagen) and eluted with 250 mM imidazole. The protein complex was dialyzed into a low-concentration imidazole buffer and incubated with SUMO hydrolase. The complex was further purified on a Strep-Tactin XT 4Flow cartridge (IBA) and eluted with 50 mM biotin. The protein complex was finally purified by size exclusion chromatography on a HiLoad 16/600 Superdex 75 pg column (Cytiva) in 20 mM Tris, 150 mM NaCl, 10 mM MgCl_2_, 0.5 mM TCEP, pH 7.5, concentrated, flash frozen with liquid nitrogen, and stored at −80 °C.

#### VHL/EloB/EloC complex

cDNA encoding for VHL (residues 54 to 213) in a pHAT4 vector and EloB (residues 1 to 104) and EloC (residues 17 to 112) in pCDF-1b-DUET were obtained from our previous study^26^ were transformed into *E. coli* BL21(DE3) cells and grown overnight in LB supplemented with ampicillin (100 µg mL^−1^) and streptomycin (50 µg mL^−1^) at 37 °C with shaking. The overnight culture was diluted 1:100 in LB supplemented with ampicillin (100 µg mL^−1^) and streptomycin (50 µg mL^−1^) and grown to an optical density (OD_600_) of ~0.6. Protein expression was induced overnight at 18 °C with 0.5 mM IPTG. The cells were harvested by centrifugation and frozen at −80 °C as pellets until further purification. The bacterial pellets were resuspended in buffer supplemented with 5 mM MgCl_2_, DNase I (1 µg/mL), and 1× EDTA-free Roche protease inhibitor cocktail, and lysed by cell disruption using One Shot Cell Disruptor (Constant Systems) operating at 4 °C. Cellular debris was removed by centrifugation. The His_6_-tagged protein complex was purified on a HisTrap FF Ni NTA affinity column (Cytiva) and eluted with an imidazole concentration gradient. The protein was dialyzed into a low-concentration imidazole buffer, incubated with TEV protease, and flowed through a HisTrap FF Ni NTA affinity column. The His₆-tagged VCB protein complex used in the TR-FRET assays was directly used in the next step without TEV cleavage or further Ni-NTA purification. VCB was additionally purified by anion exchange using a HiTrap Q HP column. The protein sample was finally purified by size exclusion chromatography on a HiLoad 16/600 Superdex 75 pg column (Cytiva) in 20 mM HEPES, pH 7.5, 150 mM NaCl, and 1 mM DTT, concentrated, flash frozen with liquid nitrogen, and stored at −80 °C.

#### APPBP1-UBA3 and UBE2M

APPBP1-UBA3 or UBE2M cDNA in a pGEX4TI vector were transformed into *E. coli* BL21(DE3) cells and grown overnight in LB supplemented with ampicillin (100 µg mL^−1^) at 37 °C with shaking^54,67^. The overnight culture was diluted 1:100 in LB supplemented with ampicillin (100 µg mL^−1^) and grown to an optical density (OD_600_) of ~0.6. Protein expression was induced overnight at 16 °C with 0.5 mM IPTG. The cells were harvested by centrifugation and frozen at −80 °C as pellets until further purification. The bacterial pellets were resuspended in 50 mM Tris, 200 mM NaCl, and 5 mM DTT, pH 7.5, supplemented with 5 mM MgCl_2_, DNase I (1 µg/mL), and 1× EDTA-free Roche protease inhibitor cocktail, and lysed by cell disruption using One Shot Cell Disruptor (Constant Systems) operating at 4 °C. Cellular debris was removed by centrifugation. The lysate was incubated with 10 mL of glutathione Sepharose 4B (Cytiva) resin for 1 h at room temperature. The resin was washed with buffer and the protein was eluted with 10 mM reduced L-glutathione. One unit of thrombin per milligram of total protein was added and the mixture was incubated overnight at −4 °C. APPBP1-UBA3 was further purified by size exclusion chromatography on a HiLoad 16/600 Superdex 200 pg column (Cytiva) in 20 mM HEPES, pH 7.5, 150 mM NaCl, and 0.5 mM TCEP. UBE2M was further purified by size exclusion chromatography on a HiLoad 16/600 Superdex 75 pg column (Cytiva) in 20 mM HEPES, pH 7.5, 150 mM NaCl, and 0.5 mM TCEP. The resulting proteins were flowed over glutathione Sepharose 4B resin to remove any trace amounts of GST-APPBP1-UBA3, GST-UBE2M, and GST. The purified protein samples were each concentrated, flash frozen with liquid nitrogen, and stored at −80 °C.

#### NEDD8 (residues 1–76)

NEDD8 cDNA in a pGEX4TI vector was transformed into *E. coli* BL21(DE3) cells and grown overnight in LB supplemented with ampicillin (100 μg mL^−1^) at 37 °C with shaking^67,68^. The overnight culture was diluted 1:100 in LB supplemented with ampicillin (100 μg mL^−1^) and grown to an optical density (OD_600_) of ~0.6. Protein expression was induced overnight at 16 °C with 0.5 mM IPTG. The cells were harvested by centrifugation and frozen at −80 °C as pellets until further purification. The bacterial pellets were resuspended in 30 mM Tris, 200 mM NaCl, and 5 mM DTT, pH 7.5, supplemented with 5 mM MgCl_2_, DNase I (1 μg/mL), and 1× EDTA-free Roche protease inhibitor cocktail, and lysed by cell disruption using One Shot Cell Disruptor (Constant Systems) operating at 4 °C. Cellular debris was removed by centrifugation. The His_6_-tagged protein was purified on a HisTrap FF Ni NTA affinity column (Cytiva) and eluted with an imidazole concentration gradient (20–500 mM). The resulting eluate was incubated with 10 mL of glutathione Sepharose 4B resin (Cytiva). The protein-bound resin was washed with buffer, TEV protease was added to the resin, and NEDD8 was cleaved at room temperature for 3 h. NEDD8 was finally purified by size exclusion chromatography on a HiLoad 16/600 Superdex 75 pg column (Cytiva) in 20 mM HEPES, pH 7.5, 150 mM NaCl, and 0.5 mM TCEP, concentrated, flash frozen with liquid nitrogen, and stored at −80 °C.

#### NEDD8-CRL2^VHL^

The NEDD8-CRL2^VHL^ protein complex was produced by Cul2-Rbx1 cDNA transformation into *E. coli* BL21(DE3) cells and grown overnight in LB supplemented with ampicillin (100 μg mL^−1^) at 37 °C with shaking^69^. The overnight culture was diluted 1:100 in LB supplemented with ampicillin (100 μg mL^−1^) and grown to an optical density (OD_600_) of ~0.6. Protein expression was induced for 16 h at 16 °C with 0.2 mM IPTG. The cells were harvested by centrifugation and frozen at −80 °C as pellets until further purification. The bacterial pellets were resuspended in 30 mM Tris, 200 mM NaCl, and 5 mM DTT, pH 7.5, supplemented with 5 mM MgCl_2_, DNase I (1 μg/mL), and 1× EDTA-free Roche protease inhibitor cocktail, and lysed by cell disruption using One Shot Cell Disruptor (Constant Systems) operating at 4 °C. Cellular debris was removed by centrifugation. Cul2-Rbx1 was purified on a HisTrap FF Ni NTA affinity column (Cytiva) and eluted with an imidazole concentration gradient from 0 to 300 mM. The protein was desalted into 20 mM HEPES, 150 mM NaCl, 0.5 mM TCEP, and 5% (v/v) glycerol, pH 7.5, with a HiPrep 26/10 desalting column (Cytiva) and incubated with TEV protease and an excess of recombinant VHL-EloB-EloC. VHL-EloB-EloC-Cul2-Rbx1 (CRL2^VHL^) was purified on a StrepTrap XT column (IBA), and eluted with 50 mM biotin. Purified CRL2^VHL^ (1.7 μM) was incubated with APPBP1-UBA3 (250 nM), UBE2M (1.2 μM), NEDD8 (20 μM), adenosine triphosphate (ATP) (1 mM), and MgCl_2_ (5 mM) for 10 min at 37 °C. NEDD8-CRL2^VHL^ was finally purified by size exclusion chromatography on a HiLoad 16/600 Superdex 200 pg column (Cytiva) in 20 mM HEPES, 150 mM NaCl, 0.5 mM TCEP, and 5% (v/v) glycerol, pH 7.5, concentrated, flash frozen with liquid nitrogen, and stored at −80 °C.

#### GACG-ubiquitin

His_6_-TEV-GACG-ubiquitin cDNA^70^ was transformed into SoluBL21(DE3) competent cells were grown overnight in LB supplemented kanamycin (50 μg mL^−1^) at 37 °C with shaking^54^. The overnight culture was diluted 1:100 in LB supplemented with kanamycin (50 μg mL^−1^) and grown to an optical density (OD_600_) of ~0.75. Protein expression was induced for 4 h at 37 °C with 1 mM IPTG. The cells were harvested by centrifugation and frozen at −80 °C as pellets until further purification. The bacterial pellets were resuspended in 20 mM HEPES, 500 mM NaCl, and 0.5 mM TCEP, pH 7.5, supplemented with 5 mM MgCl_2_, DNase I (1 μg/mL), and 1× EDTA-free Roche protease inhibitor cocktail, and lysed by cell disruption using One Shot Cell Disruptor (Constant Systems) operating at 4 °C. Cellular debris was removed by centrifugation. His_6_-TEV-GACG-ubiquitin was purified on a HisTrap FF Ni NTA affinity column (Cytiva) and eluted with an imidazole concentration gradient from 0 to 500 mM. The protein was dialyzed into 50 mM HEPES, 150 mM NaCl, and 0.5 mM TCEP, pH 7.5, incubated with TEV protease, and flowed through a HisTrap FF Ni NTA affinity column. GACG-ubiquitin was finally purified by size exclusion chromatography on a HiLoad 16/600 Superdex 75 pg column (Cytiva) in 50 mM Tris, 150 mM NaCl, and 0.5 mM TCEP, pH 7.5, concentrated, flash frozen with liquid nitrogen, and stored at −80 °C.

#### Ubiquitin-activating enzyme 1

The ubiquitin-activating enzyme 1 (UBA1) was expressed and purified by UBA1-His_6_ cDNA^52^ transformation into *E. coli* Rosetta 2(DE3) competent cells and were grown overnight in LB supplemented with kanamycin (50 μg mL^−1^) at 37 °C with shaking. The overnight culture was diluted 1:100 in LB supplemented with kanamycin (50 μg mL^−1^) and 1 mM MgSO_4_ and grown to an optical density (OD_600_) of ~0.8. Protein expression was induced for 16 h at 16 °C with 0.2 mM IPTG. The cells were harvested by centrifugation and frozen at −80 °C as pellets until further purification. The bacterial pellets were resuspended in IMAC buffer A (20 mM sodium phosphate, 500 mM NaCl, 50 mM imidazole, and 1 mM TCEP, pH 7.4), supplemented with 5 mM MgCl_2_ DNase I (1 μg/mL), and 1× EDTA-free Roche protease inhibitor cocktail, and lysed by cell disruption using One Shot Cell Disruptor (Constant Systems) operating at 4 °C. Cellular debris was removed by centrifugation, and UBA1 was purified on a HisTrap FF Ni NTA affinity column (Cytiva) and eluted with 100% IMAC Buffer B (20 mM sodium phosphate, 500 mM NaCl, 350 mM imidazole, and 1 mM TCEP, pH 7.4). The elution was concentrated to ~2 mL and diluted 1:100 in Anion Buffer A (50 mM Tris and 5 mM β-mercaptoethanol, pH 8.0) and loaded on an equilibrated Q-HP column (Cytiva). A 50% gradient over 40 CV with Anion Buffer B (50 mM Tris, 1 M NaCl, and 5 mM β-mercaptoethanol, pH 8.0) was applied and UBA1 containing fractions were concentrated and finally purified by size exclusion chromatography on a 16/600 Superdex 200 column (Cytiva) equilibrated in 20 mM HEPES, 150 mM NaCl, and 1 mM TCEP, pH 8.0, concentrated, flash frozen with liquid nitrogen, and stored at −80 °C.

#### UBE2D2 and UBE2R1

The UBE2D2 and UBE2R1 proteins were expressed and purified by His_6_-SUMO-E2 cDNA transformation into *E. coli* Rosetta 2 (DE3) competent cells and were grown overnight in LB at 37 °C until optical density (OD600) of 0.8, at which the temperature was reduced to 16 °C and cultures were induced with 0.3 mM IPTG for ~16 h^71^. Cells were collected at 3500 × *g*, resuspended in lysis buffer (20 mM Tris–HCl, 500 mM NaCl, 1 mM TCEP, pH 7.4), and lysed using microfluidizer. The cellular debris was removed by centrifugation (45 min, 18,000 × *g*). The resulting protein-containing supernatant was subjected to His-Trap affinity purification. Specifically, the HisTrap FF Ni NTA affinity column was washed with wash buffer (20 mM Tris–HCl, 500 mM NaCl) and the protein eluted with elution buffer (300 mM imidazole, 1 mM TCEP, pH 7.4), SUMO tag was then cleaved by ULP1 (5 µM), and a further round of His-Trap affinity purification was performed to remove the protease, before final purification by size exclusion chromatography using a TRIS-buffer (20 mM Tris–HCl, 200 mM NaCl, 0.5 mM TCEP, pH 7.5).

#### Ubiquitin labelling with maleimide Alexa Fluor 488

Alexa Fluor 488 C5 maleimide dye was dissolved to 5 mM in anhydrous DMSO. Ubiquitin cysteine mutant recombinant protein (GACG-ubiquitin) was buffer exchanged into 20 mM HEPES and 150 mM NaCl, pH 7.0, using a 10/300 GL Superdex 75 Increase prepacked column (Cytiva). The fractions containing the protein were combined, concentrated to 100 µM and labelled with a five times molar excess of fluorescent dye for 2 h at room temperature. The excess dye was removed by gel filtration on a 10/300 GL Superdex 75 Increase prepacked column (Cytiva), eluting in 20 mM HEPES, 150 mM NaCl, and 0.5 mM TCEP, pH 7.5. The dye and subsequently dye-labelled protein were protected from light throughout, flash frozen, and stored at −80 °C.

#### Fluorescence anisotropy assays

Fluorescence anisotropy assays were conducted using FAM labeled ERα 8mer peptide (10 nM, FAM-AEGFPA phosphoT V-COOH, Genscript)^72^, or FAM labeled HIF1α 10mer peptide (25 nM, FAM-DEALA hydroxyPYIPD-COOH, Tocris)^73^. All assays were conducted using freshly prepared buffer (20 mM HEPES, 100 mM NaCl, 100 µM TCEP, 0.1 % (v/v) Tween20, 1 mg/mL BSA, pH 7.5, 1 % (v/v) DMSO) in 384-well plates (black, round bottom, low binding surface, 10 μL sample per well, Corning). Titrations were performed using a Thermo Scientific E1-ClipTip autopipette (1:1 dilution series). Measurements were performed directly after plate preparation, using a Tecan Spark plate reader (25 °C; *λ*_ex_ 485 ± 20 nm; *λ*_em_ 535 ± 25 nm; dichroic 510 mirror; 30 flashes; 40 µs integration time; 0 µs lag time, 1 ms settle time; gain 66 for ERα peptide and 55 for HIF1α peptide; Z-position calculated from well). Wells containing only labeled peptide in buffer were used to calculate the G-factor. The data was plotted using Origin software. Using the same software, sigmoidal functions were fitted to the data using the following formula: anisotropy = start + (end–start)∙(*x*^n^)/(*k*^n^ + *x*^n^); start = bottom asymptote; end = top asymptote; *x* = titrant concentration; *k* = *K*_*D*_; *n* = Hill coefficient. Fluorescence anisotropy measurements are given as milli-Arbitrary Units.

#### Cellular VHL engagement assays

VHL NanoBRET target engagement assays (N2931, Promega) were conducted following the manufacturer’s guidelines. The day before transfection, MCF7 cells (obtained from ATCC) were seeded in a 6-well plate at 4 × 10^5^ cells per well. Transfection complexes were prepared by mixing 100 μL of Opti-MEM (Thermo Fisher Scientific #11058021), 6 µL of FuGENE® HD (Promega #E2311), 1 µg Transfection Carrier DNA, and 1 µg of the VHL-NanoLuc fusion vector. This was incubated for 20 min at room temperature, then added to the cells. The following day, 75 µL of transfected cells (at 2.2 × 10^5^ cells/mL) were plated in White, flat-bottom, Non-Binding Surface 96-well plates (Corning #3600). PROTACs and vehicle controls were prepared at 10× final concentrations in Opti-MEM. For the live-cell assay, 10 µL of Opti-MEM was added to each well, followed by 10 µL of each 10× PROTAC and vehicle control dilution. A 100× solution of NanoBRET tracer (50 µM for VHL live-cell assay, from kit N2931, Promega) was prepared in 100% DMSO and then diluted to a 20× solution using tracer dilution buffer. A final volume of 5 µL of the tracer was added to each well for a final concentration of 0.5 µM. Plates were mixed on an orbital shaker at 300 RPM for 15 s and then incubated at 37 °C with 5% CO₂ for 4 h. Following incubation, 50 µL of a substrate solution (1/400 NanoBRET Nano-Glo substrate, 1/500 Extracellular NanoLuc inhibitor in Opti-MEM) was added to each well. After 2–3 min of incubation at room temperature, donor (450 nm) and acceptor (610 nm) emissions were measured on a BMG Labtech PHERAstar luminescence plate reader. For the permeabilized-cell assay, 10 µL of digitonin was added instead of Opti-MEM. In each well, 10 µL of the 10× PROTAC and vehicle control dilutions were dispensed. A 100× NanoBRET tracer solution (25 µM for the VHL permeabilized-cell assay, from kit N2931, Promega) was prepared with 100% DMSO and then diluted to a 20× solution with tracer dilution buffer. A 5 µL volume of tracer was dispensed into each well at a final concentration of 0.25 µM. This was incubated for 10 min, and the plate was mixed at 300 RPM for 15 s. Finally, 50 µL of a substrate solution (1/400 NanoBRET Nano-Glo substrate in Opti-MEM) was added, and plates were incubated for 2–3 min at room temperature before reading on a BMG Labtech PHERAstar. The data was processed and analyzed using Origin software.

#### Mass photometry

Interferometric scattering microscopy was carried out using the oneMP (Refeyn). The buffer (20 mM HEPES, 150 mM NaCl, and 0.5 mM TCEP, pH 8.0) was filtered through a 0.22-μm syringe filter, and gasket wells (Grace Bio-labs CW-50R-1.0) along with high-precision 24 × 50 mm coverslips (Marienfeld) were prepared. Each sample was prepared to contain 5 μM 14-3-3ζ/ERα(PKA)-LBD complex, 5 μM VCB, and 12.5 μM compound, with 5% DMSO. Samples were incubated at room temperature for an hour, then diluted to 50 nM in buffer. Buffer (10 μL) was used for focusing in regular mode (128 × 34 binned pixels 18.0 μm^2^ detection area) and all data were recorded for 60 s using AcquireMP software (Refeyn). Calibration standards for mass calibration consisting of conalbumin (Mr = 75,000), aldolase (Mr = 158,000), ferritin (Mr = 440,000), and thyroglobulin (Mr = 669,000) were prepared from the gel filtration HMW calibration kit (Cytiva). All data were processed and analyzed in DiscoverMP (Refeyn).

#### Cryo-EM sample preparation

The purified 1:1 complex of 14-3-3ζ/ERα^(LBD_F)^ (50 μM) was incubated with VCB and CV2a at a molar ratio of 14-3-3ζ:ERα^(LBD_F)^:VCB:CV2a = 1:1:1.2:1.2 on ice for 1 h in cryo-EM buffer (20 mM HEPES, 100 mM NaCl, 0.5 mM TCEP, pH 7.5). Right before plunge freezing, the complex was diluted 20-fold in cryo-EM buffer and 3.5 μL was applied to the carbon side of 400 mesh copper (Cu) R1.2/1.3 holey carbon Quantifoil grids. TEM grids were glow discharged for 60 s under vacuum at 30 mA using a Quorum SC7620 just before use. A Vitrobot Mark IV (Thermo Fisher Scientific) operating at 4 °C and 100% humidity was set to blot force = 4 and blot time = 4 s for plunging grids into liquid ethane.

#### Cryo-EM data acquisition

All grids were clipped and screened at the University of Dundee Electron Microscopy Facility using the Glacios (Thermo Fisher Scientific) operating at 200 kV equipped with a Falcon4i direct electron detector. The dataset for the 14-3-3ζ:ERα^(LBD_F)^:VCB:CV2a complex was collected at eBIC (Diamond Light Source, UK) under BAG access BI-31827-16 using Krios-4 (m07) operating a Schottky X-FEG at 300 kV and Ametek-Gatan BioQuantum K3 Imaging Filter with slit width of 20 eV. Data were recorded using single particle EPU v2.1 (Thermo Fisher Scientific) at a nominal magnification of 105,000×, calibrated pixel size of 0.829 Å, C2 aperture 70 μm, and objective aperture of 100 μm. All movies were collected using a Gatan K3 direct electron detector (5760 × 4092 pixels) in counting mode over 40 dose fractions, recorded as Tiff LZW non-gain normalised, with total measured dose of 32.59 e−/Å², exposure time of 1.75 sec, and dose rate of = 15.7e/px/s. A total of 14,091 movies were acquired using aberration-free image shift. The data acquisition parameters are summarised in Supplementary Table 2.

#### Cryo-EM image processing, validation and model building

Image processing pipelines are described in Supplementary Fig. 9. Cryo-EM movies were imported into CryoSPARC v.4.5^74^ for patch motion correction, patch CTF estimation and manual curation. Particle picking was performed with crYOLO^75^ using a general model for low-pass filtered images. Particles were extracted with a 300 pixel box size and 4 rounds of 2D classification yielded a particle stack of 745,250 particles. These particles were separated into 8 classes using ab initio classification. Particles corresponding to Pose 1 were used for TOPAZ training, the resulting particles were 2D classified and the best particles were taken forward for non-uniform refinement, resulting in the 4.6 Å Pose 1 structure. Local refinement using a mask around EloB/EloC/VHL/CV2a/14-3-3ζ(monomer) resulted in the 4.3 Å locally refined Pose 1 structure. DeepEMhancer^76^ was used for Pose 1 figure generation and model building. Particles corresponding to Pose 1 were used for TOPAZ^74^ training, the resulting particles were 2D classified and the best particles were taken forward for ab initio classification into 3 classes. The full particle stack was taken forwards and refined against the two best volumes from ab initio classification, resulting in Pose 2.1 and Pose 2.2 structures. The particles corresponding to Pose 3 were refined using non-uniform refinement resulting in the Pose 3 structure. All classification was achieved by 2D classification, and 3D reconstruction was performed with ab initio reconstruction, heterogeneous refinement and non-uniform refinement in cryoSPARC^74^. Representative 2D classes, orientation diagnostics, local resolution estimations and gold-standard Fourier shell correlation curves were generated with cryoSPARC and are shown in Supplementary Figs. 9–12. Model building was achieved using atomic models from AlphaFold and PDB entries 4W9H, 8C43 and 8BZH, which were docked into the cryo-EM maps using rigid-body fitting with UCSF ChimeraX^77–79^. The models were refined using ISOLDE^80^ and Phenix^81^ until reasonable agreement between the model and data were achieved. Structural figures were generated using the UCSF ChimeraX programme^77–79^. The data image analysis, atomic modelling, refinement and validation statistics are summarised in Supplementary Tables 3 and 4.

#### Molecular dynamics simulations

Modelling of CV2a into the cryo-EM structure was performed using Maestro (Schrödinger), in triplicate. The docked structures of VH032 in complex with VHL and of fusicoccin A in complex with 14-3-3 were used for MD simulations. Modifications to the chemical structures and modelling of the aliphatic linker region were performed using the build tool in Maestro (Schrödinger). The resulting ternary VHL/CV2a/14-3-3 complex was pre-treated using the Protein Preparation Workflow (PPW) implemented in the Schrödinger Suite (Release 2024-3) prior to MD simulations. Protein preparation was carried out at pH 7.5 by adding hydrogen atoms and building missing side-chain atoms. The hydrogen-bonding network was optimised by reorienting hydroxyl and thiol groups, water molecules, amide groups of asparagine (Asn) and glutamine (Gln), and the imidazole ring of histidine (His). Protonation states of histidine, aspartic acid (Asp), and glutamic acid (Glu), as well as tautomeric states of histidine, were assigned automatically by the PPW. MD simulations were performed using the Desmond MD System^82^ implemented within the Schrödinger Suite Release 2024-3 (Schrödinger, LLC). The prepared system was solvated in an orthorhombic box of explicit SPC water molecules, with a buffer distance of 12 Å between the solute and the box boundaries. The pH of the system was set to 7.5 and sodium and chloride ions were added to neutralise the system and to achieve a final salt concentration of 0.1 M. Ligand parameters, including partial atomic charges, were assigned using the OPLS4 force field as implemented in Schrödinger. Prior to production MD, the system was relaxed using the default Desmond relaxation protocol consisting of a short MD simulation (100 ps) under NVT conditions at 10 K, in three sequential steps. In the first step, positional restraints were applied to all atoms of the proteins and the ligand. In the second step, restraints were applied only to the Cα atoms of the proteins. In the final step, all positional restraints were removed, allowing full relaxation of the system while preserving the overall structural integrity. Production MD simulations were carried out in the NPT ensemble at 300 K and 1 bar using Desmond default settings, a Nosé–Hoover chain thermostat (relaxation time 1 ps), and a Martyna–Tobias–Klein barostat (relaxation time 2 ps). A multi-time-step RESPA integrator was employed, using a time step of 2 fs for bonded and short-range interactions and 6 fs for long-range interactions. Long-range electrostatic interactions were treated using the u-series decomposition with a real-space cut-off of 9 Å. 500 ns MD simulations were initiated from randomised initial velocities. Coordinates and energies were saved every 250 ps, resulting in 2000 frames per trajectory. Interactions that occurred more than 30% of the simulation time (0–500 ns) were shown in Fig. 3g (E208^14-3-3^:H110^VHL^, E208^14-3-3^:R108^VHL^, Y211^14-3-3^:Y112^VHL^, D204^14-3-3^: P99^VHL^, Q219^14-3-3^:N67^VHL^, R222^14-3-3^:D92^VHL^). The geometric criteria for protein-ligand hydrogen-bonds was: a distance of 2.5 Å between the donor and acceptor atoms, a donor angle of ≥120° between the donor and hydrogen-acceptor atoms, and an acceptor angle of ≥90° between the hydrogen-acceptor-bonded atoms. The three-dimensional structures and trajectories were inspected using Maestro (Schrödinger) and all analyses reported in this study are based on these production trajectories.

#### Time-resolved FRET assays

TR-FRET assays were conducted using His-tagged VCB, biotinylated 14-3-3γ, 20 µM ERα 8mer peptide (Ac-AEGFPA phosphoT V-COOH, Genscript)^70^, anti-His mAb Tb-conjugate (61HISTLF, Revvity), and streptavidin D2-conjugate (610SADLF, Revvity). All assays were conducted using freshly prepared buffer (10 mM HEPES, 150 mM NaCl, 5 mM DTT, 100 μM CHAPS, 1 mg/mL BSA, pH 7.5, 1% (v/v) DMSO) in 384-well plates (white, round bottom, low binding surface, 5 μL sample per well, Corning). Titrations were performed using a Thermo Scientific E1-ClipTip autopipette (1:1 dilution series). Measurements were performed directly after plate preparation, using a Tecan Spark plate reader (25 °C; *λ*_ex_ 340 ± 35 nm; *λ*_em_ 620 ± 10 nm (gain 159) and 665 ± 8 nm (gain 178); dichroic 510 mirror; 30 flashes; 500 µs integration time; 150 µs lag time, 1 ms settle time; Z-position calculated from well). The TR-FRET ratio was calculated by dividing the emission signal at 665 nm by the emission signal at 620 nm of the respective well. The data was plotted using Origin software and fitted using a freehand fit to approximate the trend, with no underlying model applied. All datapoints are based on three independent experiments from which the average and standard deviation were calculated.

Compound titrations were performed using 20 µM unlabeled ERα peptide, 50 nM His-tagged VCB complex, 50 nM biotinylated 14-3-3γ, 0.5 nM anti-His Tb-conjugated mAb and 12.5 nM streptavidin D2-conjugate. For two-dimensional titrations unlabeled ERα peptide (from 12.5 µM) and CV2a (from 100 µM) were titrated to 50 nM His-tagged VCB complex, 50 nM biotinylated 14-3-3γ, 0.5 nM anti-His Tb-conjugated mAb and 12.5 nM streptavidin D2-conjugate.

#### In vitro ubiquitination assays

For the time-course ubiquitination assay and mass spectrometry analysis, the E1 Ube1 (2 μM), the E2 UBE2D2 (5 μM), the E2 UBE2R1 (1 μM), 14-3-3zeta/ERa^LBD_F^ (12 μM), CRL2^VHL^ (500 nM), and ^MG^PROTAC (5 μM) were mixed with ubiquitin (300 μM) and incubated in 30 mM Tris, 150 mM NaCl, 5% glycerol, and 15 mM MgCl_2_, pH 8.0, for 15 min at room temperature. ATP (10 mM) was added, and the mixture was incubated at 37 °C. The reaction was quenched with SDS sample buffer. The protein species were either taken forwards for mass spectrometry analysis or resolved by SDS-PAGE on a 4–12% bis-tris NuPAGE gel using MES running buffer, run at 200 V for 35 min. The SDS-PAGE gel was stained with Coomassie Instant Blue.

For all other ubiquitination assays, the E1 Ube1 (150 nM), the E2 UBE2D2 or UBE2R1 (5 μM), 14-3-3zeta/ERa^LBD_F^ (5 μM), NEDD8-CRL2^VHL^ (150 nM), and PROTAC (1 μM) were mixed with ubiquitin (98 μM) and Alexa Fluor 488-labelled ubiquitin (2 μM) and incubated in 20 mM HEPES, 150 mM NaCl, 0.5 mM TCEP, and 5 mM MgCl_2_, pH 7.5, for 5 min at room temperature. ATP (3 mM) was added, and the mixture was incubated at room temperature. The reaction was quenched with reducing SDS sample buffer. The protein species were resolved by SDS-PAGE on a 10% bis-tris NuPAGE gel using MOPS running buffer, run at 175 V for 50 min. The SDS-PAGE gel was stained with Coomassie Instant Blue, or taken forward for Western Blot. Proteins were transferred to a nitrocellulose membrane either by wet transfer at 90 V for 90 min, or using an iBlot 2 Gel Transfer Device (Invitrogen). Membranes were blocked for 1 h in 5% milk in TBS-T and incubated overnight with primary antibodies (1:1000, for ERα rabbit mAb CST #8644, for 14-3-3 CST #8312), washed with TBS-T, then incubated for 1 h with secondary antibodies (1:5000) before imaging on the ChemiDoc (BioRad). For an example of presentation of the full scan blots, see Supplementary Fig. S23 or the Source Data file.

#### Cell treatments and Western blot

MCF7 cells were maintained in high glucose DMEM with GlutaMAX (Gibco), supplemented with 10% FBS (ThermoFisher Scientific) and grown in a humidified incubator at 37 °C and 5% CO2.

For Western Blot degradation assays, cells were plated in 6-well plates at 4 × 10^5^ cells per ml. The next day, the media was changed to hormone-free media (high glucose DMEM with GlutaMAX supplemented with 10% Charcoal Stripped FBS (Gibco)) and cultured for three days. The media was then exchanged for fresh hormone-free media containing working solutions of the compounds (1:1000 from DMSO stock) and incubated for 24 h.

For cell collection, cells were washed once with PBS before lysis on ice for 20 min with RIPA buffer supplemented with Protease inhibitor cocktail (1:100, ab271306, abcam) and Phosphatase inhibitor cocktail PhosSTOP (4906845001, Roche). After removal of the insoluble fraction by centrifugation at 15,000 × *g* at 4 °C for 15 min, protein concentration was determined using the Pierce BCA Protein Assay (23225, ThermoFisher Scientific) and 20 µg of lysate was prepared using 4× LDS sample buffer (NP0007, Thermo Fisher) and 50 mM dithiothreitol (DTT) and heated at 95 °C for 5 min. The samples were run on NuPAGE 4–12% bis-tris gels (Thermo Fisher). Proteins were transferred to nitrocellulose membranes (GE Healthcare, Amersham Protran supported 0.4 mm NC), blocked for 1 h with 5% BSA in Tris-buffered saline with Tween-20 (TBS-T) at room temperature, before incubating with primary antibody against phosphorylated Threonine 594 of ERα (pT594-ERα-antibody, 1:200, GL Biochem)^38^ overnight at 4 °C. Membranes were then washed in TBS-T and incubated with IRDye 800CW anti-rabbit (1:5000, 926-32211, Li-Cor) and hFABTM rhodamine anti-tubulin (1:5000, 12004165, Bio-Rad) for 1 h at room temperature, before further washes and imaging on a ChemiDoc Touch imaging system (Bio-Rad) operated on Image Lab software (version 2.4.0.03)

### 14-3-3 immuno-precipitation mass spectrometry

#### Cell treatment

MCF-7 cells (commercial supplier; ATCC) were maintained in DMEM medium supplemented with 10% (v/v) fetal bovine serum (FBS) at 37 °C in a humidified 5% CO₂ incubator. MCF-7 cell lines were authenticated by STR profiling (Cell Line Authentication Service of Eurofins Genomics, Ebersberg, Germany), Cells were seeded in 15-cm tissue culture dishes (5.0 · 106 cells per dish) and grown to ~80% confluency prior to treatment. Culture medium was replaced with fresh medium containing DMSO (0.1% (v/v)) or 10 μM CV2a. Treatments were performed for 6 and 24 h. After treatment, cells were washed twice with ice-cold PBS, harvested by scraping, and pelleted by centrifugation (4 °C, 300 × *g*, 5 min). Cell pellets were snap-frozen in liquid nitrogen and stored at −80 °C until processing.

#### Immuno-affinity purification

Cells were lysed in for 15 min at 4 °C in 3× pellet volume IP lysis buffer (Thermo Fisher Scientific) supplemented with 1× cOmplete mini EDTA-free protease inhibitor cocktail (Roche Diagnostics) and 1× PhosSTOP (Roche Diagnostics). Subsequently, the lysate was cleared by centrifugation for 15 min at 4 °C at 21,000 × *g*. The protein concentration was determined with the Pierce Rapid Gold BCA protein assay (Thermo Fisher Scientific).

The 14-3-3 complexes were immunoprecipitated using 15 µg pan-14-3-3 antibody (in-house) coupled to 50 µl protein A/G magnetic beads (slurry, Thermo Fisher Scientific) from 1 mg protein lysate. Incubation took place for approximately 16 h at 4 °C. After immunoprecipitation, the beads were washed with 3 times 500 µl cold TBST. For on-bead digestion, the proteins were reduced, alkylated and digested sequentially with 0.5 µg trypsin. The samples were desalted using Sep-Pak C18 columns (Waters), dried by vacuum centrifugation and reconstituted in 2% formic acid prior to LC-MS/MS analysis. Experiments were performed as biological triplicates (*n* = 3) for both compound treatment and DMSO control samples.

#### LC-MS/MS analyses

The data were acquired with an Vanquish Neo UHPLC system (Thermo Fisher Scientific) coupled to an Orbitrap Exploris 480 mass spectrometer (Thermo Fisher Scientific). Peptides were trapped (PepMap Neo 5 µM C18 300 µm × 5 mm Trap Cartridge, Thermo Fisher Scientific) for 5 min in solvent A (0.1% formic acid in water) before being separated on an analytical column (Acclaim PepMap HPLC column 3 µM C18 50 cm × 75 µM, Thermo Fisher Scientific). Solvent B consisted of 0.1% formic acid in 80% acetonitrile. The gradient was as follows: first 5 min of trapping, followed by 68 min of gradient from 7 to 28% solvent B and, subsequently, 10 min of washing with 90% solvent B and 10 min of re-equilibration with 100% solvent A.

The mass spectrometer operated in data-dependent mode. Full scan MS spectra from m/z 375–1600 were acquired at a resolution of 60.000 to a target value of 2.5 × 106 or a maximum injection time of 120 ms. The most intense precursors with a charge state of 2+ to 5+ were chosen for fragmentation. High collision dissociation (HCD) fragmentation was performed at 28% normalized collision energy on selected precursors with 30 s dynamic exclusion at a 1.4 m/z isolation window. Tandem mass spectrometry (MS/MS) spectra were acquired at a resolution of 15.000.

#### Data analysis

Raw files were searched using Proteome Discoverer 2.3 using the Sequest HT search engine against the human uniprot database (20434 entries, downloaded in May 2024). Enzyme specificity was set to trypsin and up to 2 missed cleavages were allowed. Cysteine carbamidomethylation was set as fixed modification. Methionine oxidation and N-terminal acetylation were set as variable modifications. The false discovery rate (FDR) was restricted to 1% in both protein and peptide identification. Data normalization, imputation and statistics were performed using an in-house R-script.

#### Mass spectrometry to identify ubiquitination sites

The in vitro ubiquitination assay samples were used for mass spectrometry analysis (see In vitro ubiquitination assays). Reduction and alkylation of cysteine residues were carried out using a final concentration of 10 mM DTT at 60 °C for 20 min; and 20 mM iodoacetamide at room temperature for 30 min in the dark, respectively. In-solution digestion was carried out with 50 µg of protein from each sample. Digestion of the samples was carried out using trypsin (sequencing grade) in the ratio of 1:20 (enzyme:protein) at 37 °C overnight at 500 rpm in a thermomixer. Samples were then acidified with an aqueous solution of 1% formic acid to stop the reaction and were desalted using Sep-Pak C18 tip. Eluted samples were dried in a speed vacuum. The dried samples were reconstituted in 1% formic acid and analyzed on an Orbitrap Ascend mass spectrometer coupled to a Thermo Fisher Scientific Vanquish Neo UHPLC. Samples were prepared as a single experimental set (*n* = 1). Specifically, one sample was analysed from an in vitro ubiquitination experiment performed on a single day using the same stock reagents. The peptides were enriched on a trap column and resolved on an analytical column (Easy-Spray PepMap Neo 2 μm C18 75 μm × 150 mm) with 300 nl/min. The gradient for separation was used as 35% B at 70 min. Total run time used was 100 min. The MS data acquisition was carried out from 350 to 1600 m/z range using an Orbitrap mass analyzer. The automatic gain control target was set to 400,000 with an ion injection time of auto and a dynamic exclusion of 45 s. The Orbitrap Ascend mass spectrometer was operated in the data-dependent acquisition mode. Precursor ions were fragmented using 27 HCD and were analyzed using an ion trap mass analyzer. The mass spectrometry raw data were searched using SEQUEST HT search engines with Proteome Discoverer 3.0 (Thermo Fisher Scientific). The following parameters were used for searches: Precursor mass tolerance 10 ppm, fragment mass tolerance 0.6, enzyme: trypsin, Mis-cleavage: −2, fixed modification: carbamidomethylation of cysteine residues and dynamic modification: oxidation of methionine, DiGly of lysine. The data were filtered for 1% PSM, peptide and protein level FDR. For quantitation label free workflow based on precursor area was used.

### Reporting summary

Further information on research design is available in the Nature Portfolio Reporting Summary linked to this article.

## Supplementary information

Supplementary Information

Description of Additional Supplementary Files

Supplementary Movie 1

Supplementary Movie 2

Reporting Summary

Transparent Peer Review file

## Source data

Source Data

## Data Availability

All data needed to evaluate the conclusions in the paper are present in the paper and/or the Supplementary Materials. The Atomic models generated in this study have been deposited in the protein data bank (PDB) database under accession code 9SV3. The Electron microscopy reconstructions generated in this study have been deposited under accession codes EMD-55233, EMD-55235, EMD-55236, and EMD-55237. The mass spectrometry proteomics data have been deposited to the ProteomeXchange consortium via the PRIDE partner repository with the dataset identifiers PDX071378 [https://proteomecentral.proteomexchange.org/cgi/GetDataset?ID=PXD071378] and PXD075638. Source Data not available via dedicated data repositories are accessible via the Discovery repository (https://discovery.dundee.ac.uk/en/datasets/induced-ubiquitination-of-the-partially-disordered-estrogen-recep/; 10.15132/10000290) or the source data file. Source data are provided with this paper.
