# Supplementary material for: Induced ubiquitination of the partially disordered estrogen receptor alpha via a 14-3-3 directed molecular glue-PROTAC
Source: Nat Commun. 2026 Jul 15;17:8696. doi: 10.1038/s41467-026-75333-w (PMC13490399; doi:10.1038/s41467-026-75333-w)
Supplement: Supplementary file 1 [file 41467_2026_75333_MOESM1_ESM.pdf]

Induced ubiquitination of the partially disordered estrogen receptor alpha via a 14-3-3 directed molecular glue-PROTAC

Carlo J. A. Verhoef[1,‡], Charlotte Crowe[2,‡], Mark A. Nakasone[2,4], Aitana DeLaCuadra-Basté[2], Tessa Harzing[1,2], Naomi A. S. Span[1], Gajanan Sathe[2], Laura C. Demmers[3], Kentaro Iso[2], Christian Ottmann[1,3], Luc Brunsveld[1], Alessio Ciulli[2 *], and Peter J. Cossar[1,2 *]

Affiliations:

[1] Laboratory of Chemical Biology, Department of Biomedical Engineering and Institute for Complex Molecular Systems, Eindhoven University of Technology, Netherlands.

[2] Centre for Targeted Protein Degradation, School of Life Sciences, University of Dundee, 1 James Lindsay Place, Dundee DD1 5JJ, U.K.

3 Ambagon Therapeutics, Catalyst Business Center, De Lismortel 31, 5612 AR Eindhoven, Netherlands

[4] Current address: Birkbeck University of London, ISMB EM Facility. Malet Street, London, WC1E 7HX, UK.

Table of Contents

1. Supplementary Figures 2
2. Supplementary Tables 26
3. Supplementary Methods 28
4. Supplementary References 58

1. Supplementary Figures

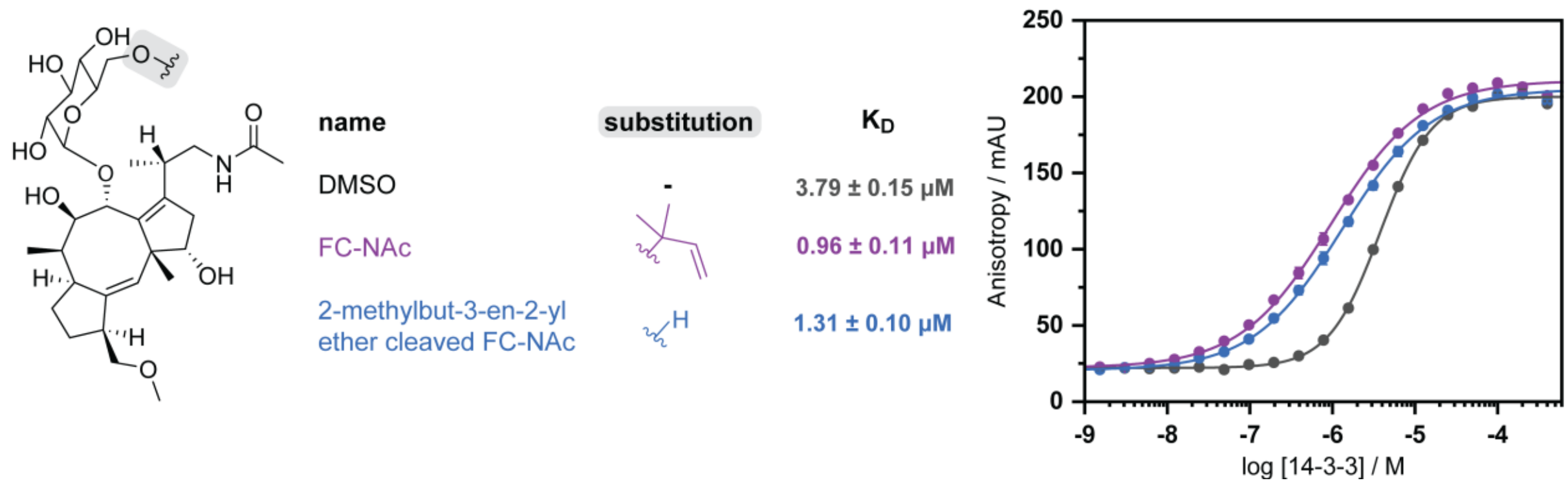

Figure S1. The 2-methylbut-3-en-2-yl ether cleaved FC-NAc stabilizes the 14-3-3/ERα interaction. FA 14-3-3 titrations in the presence of 1 µM FC-NAc or 1 µM 2-methylbut-3-en-2-yl ether cleaved FC-NAc, and 10 nM fluorescently labeled ERα peptide. Data are presented as mean values ± standard deviations (error bars) of independent scientific replicates (n = 3). Source data are provided in the Source Data file.

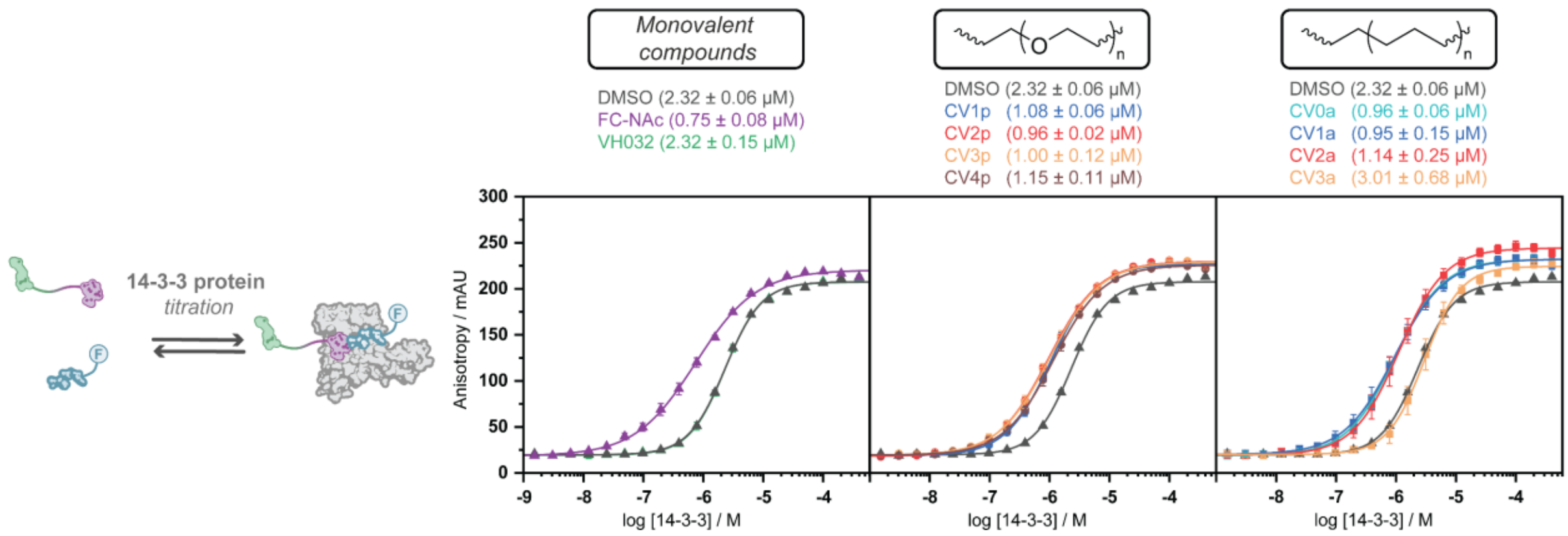

Figure S2. All synthesized PROTACs, except CV3a, stabilize the 14-3-3/ERα interaction. FA 14-3-3 titrations in the presence of 1 µM monovalent control compound, PEG linker based ^{MG}PROTAC, or alkyl linker based ^{MG}PROTAC, and 10 nM fluorescently labeled ERα peptide. Data are presented as mean values ± standard deviations (error bars) of independent scientific replicates (n = 3). Source data are provided in the Source Data file.

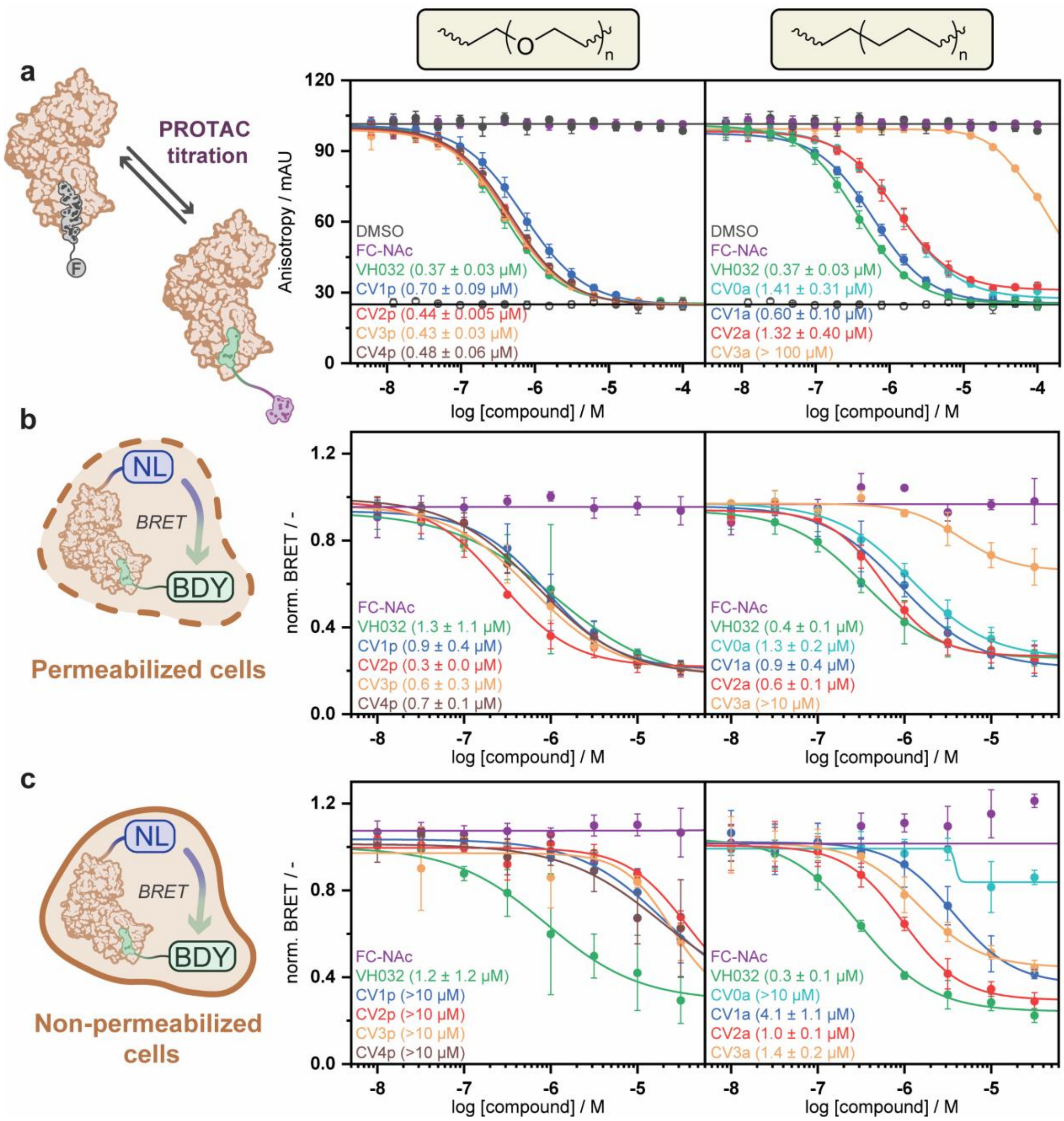

Figure S3. Biophysical and in cell VHL engagement. (a) FA displacement assay titrating monovalent control compound, PEG linker-based MGPROTAC, or alkyl linker-based MGPROTAC to 50 nM VCB and 25 nM fluorescently labeled HIF1α peptide. (b) MCF7 cells were transiently transfected with NanoLuc-VHL to from a BRET pair with the VHL tracer (0.25 µM, Promega). Normalized BRET signal of permeabilized cells after 10 min treatment with the MGPROTAC. (c) Normalized BRET signal of non-permeabilized cells after 4 h treatment with the MGPROTAC. Data are presented as mean values ± standard deviations (error bars) of independent scientific replicates (n = 3). Source data are provided in the Source Data file.

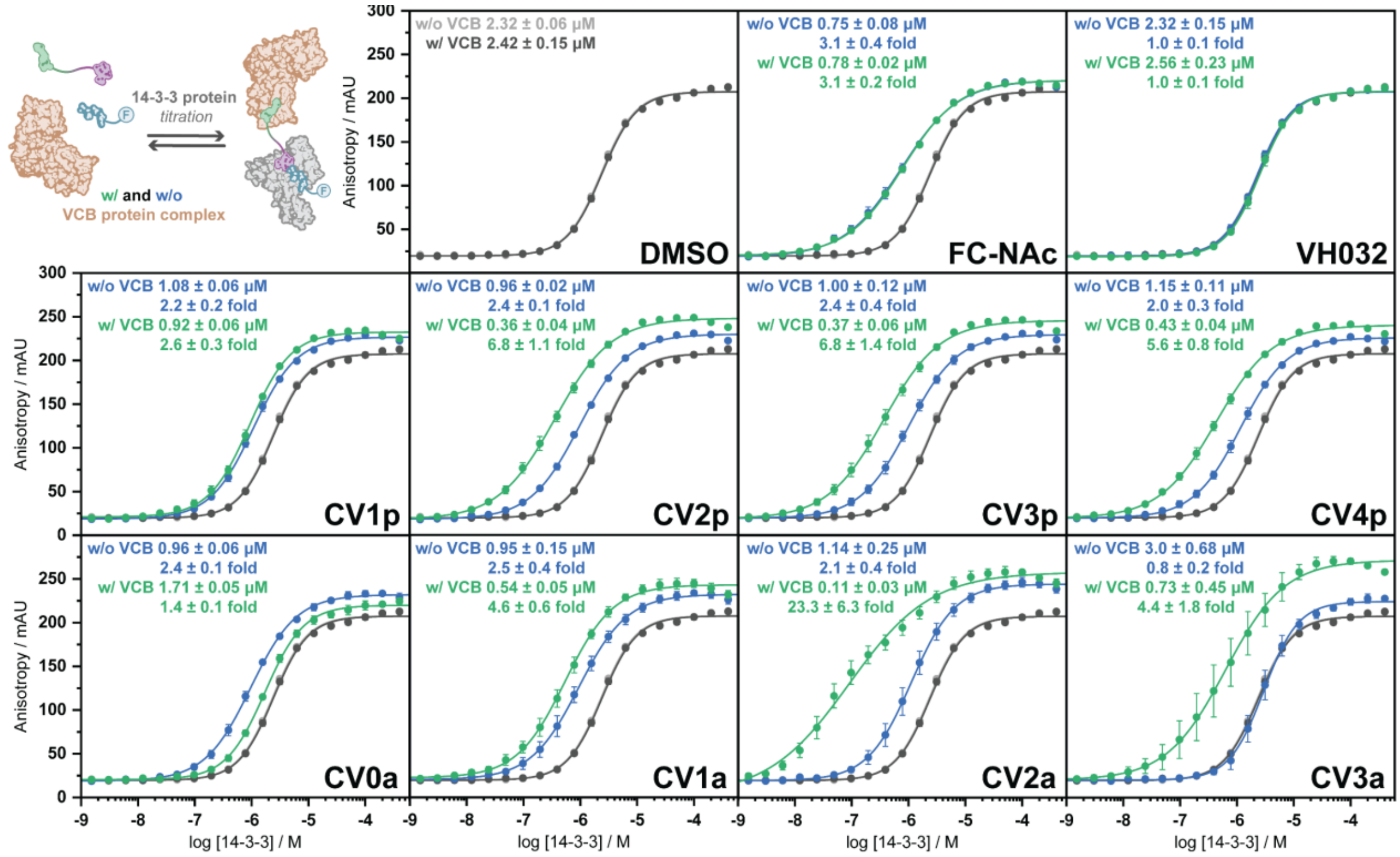

Figure S4. FA 14-3-3 titrations in the presence of 1 µM monovalent compound or MGPROTAC, and 10 nM fluorescently labeled ERα peptide in the absence of VCB (w/o VCB, blue dots) and presence of 5 µM VCB (w/ VCB, green dots). The DMSO control is indicated in grey (w/o VCB light grey dots, w/ VCB dark grey dots, data overlaps). Data are presented as mean values ± standard deviations (error bars) of independent scientific replicates (n = 3). Source data are provided in the Source Data file.

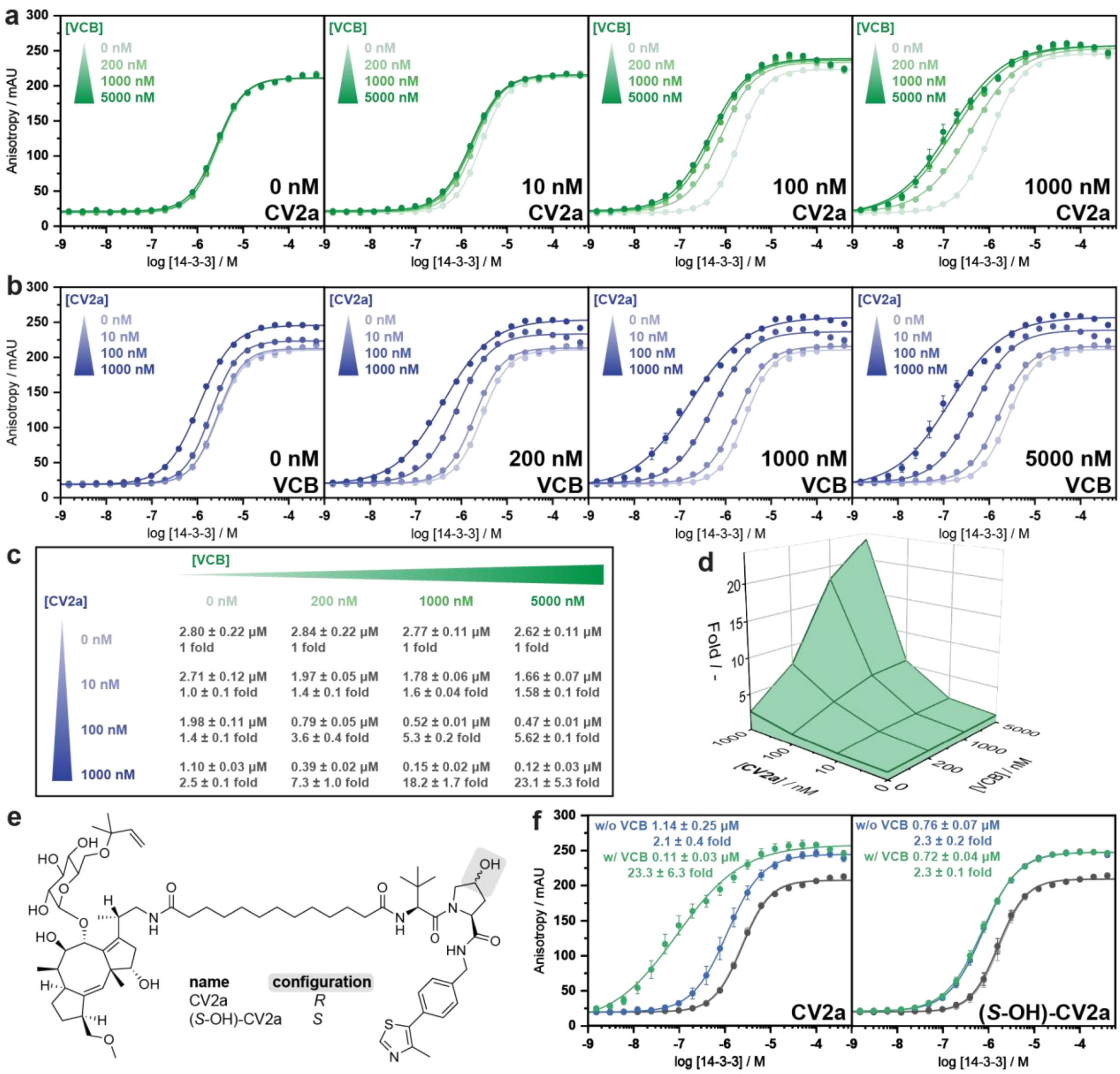

Figure S5. ERα protein stabilization is driven by cooperative protein interactions between VHL and the 14-3-3/ERα complex. FA 14-3-3 titrations in the presence of various CV2a concentrations (0, 10, 100, 1000 nM) and various VCB concentrations (0, 200, 1000, 5000 nM), and 10 nM fluorescently labeled ERα peptide. (a) Titration data grouped per CV2a concentration, and (b) per VCB concentration. (c) Overview of the fitted KDapp values and fold left shift relative to the 14-3-3 titration w/o CV2a and a similar VCB concentration. (d) 3-dimensional plot of the obtained fold left shifts. (e) Chemical structure of CV2a and (*S*-OH)-CV2a. (f) FA 14-3-3 titrations in the presence of 1 µM CV2a or its diastereoisomer (S-OH)-CV2a, and 10 nM fluorescently labeled ERα peptide in the absence of VCB (w/o VCB, blue dots) and presence of 5 µM VCB (w/ VCB, green dots). The DMSO control is indicated in grey (w/o VCB light grey dots, w/ VCB dark grey dots, data overlaps). Data are presented as mean values ± standard deviations (error bars) of independent scientific replicates (n = 3). Source data are provided in the Source Data file.

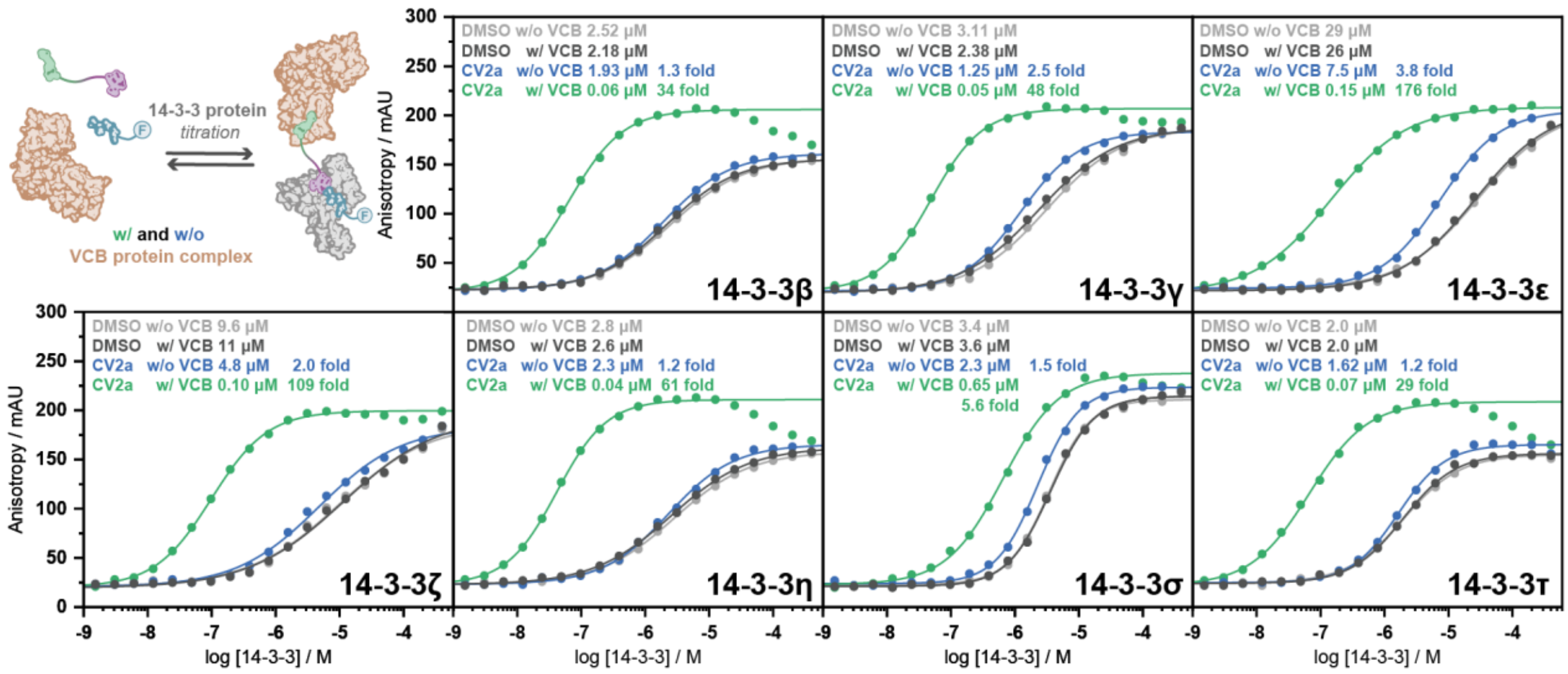

Figure. S6. VHL selective recruitment to different 14-3-3 isoforms. FA titration of all 7 human 14-3-3 isoforms in the presence of 500 nM monovalent compound or ^{MG}PROTAC, and 10 nM fluorescently labeled ERα peptide in the absence of VCB (w/o VCB, blue dots) and presence of 1 µM VCB (w/ VCB, green dots). The DMSO control is indicated in grey (w/o VCB light grey dots, w/ VCB dark grey dots, data overlaps). Data are presented as single independent scientific replicate (n = 1). Source data are provided in the Source Data file.

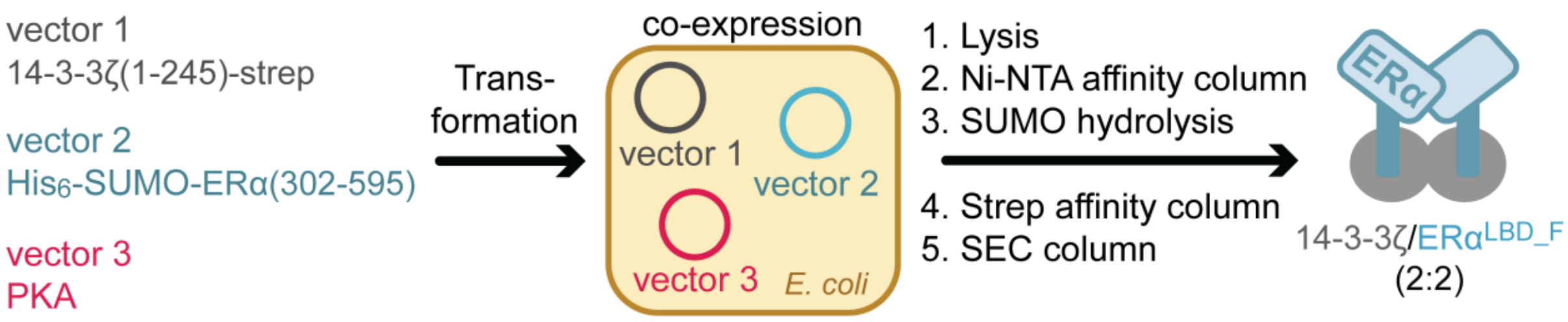

Figure S7. Overview of recombinant expression of 14-3-3ζ/ERα$^{(LBD_F)}$ complex. Adapted from Somsen *et al.* [1]

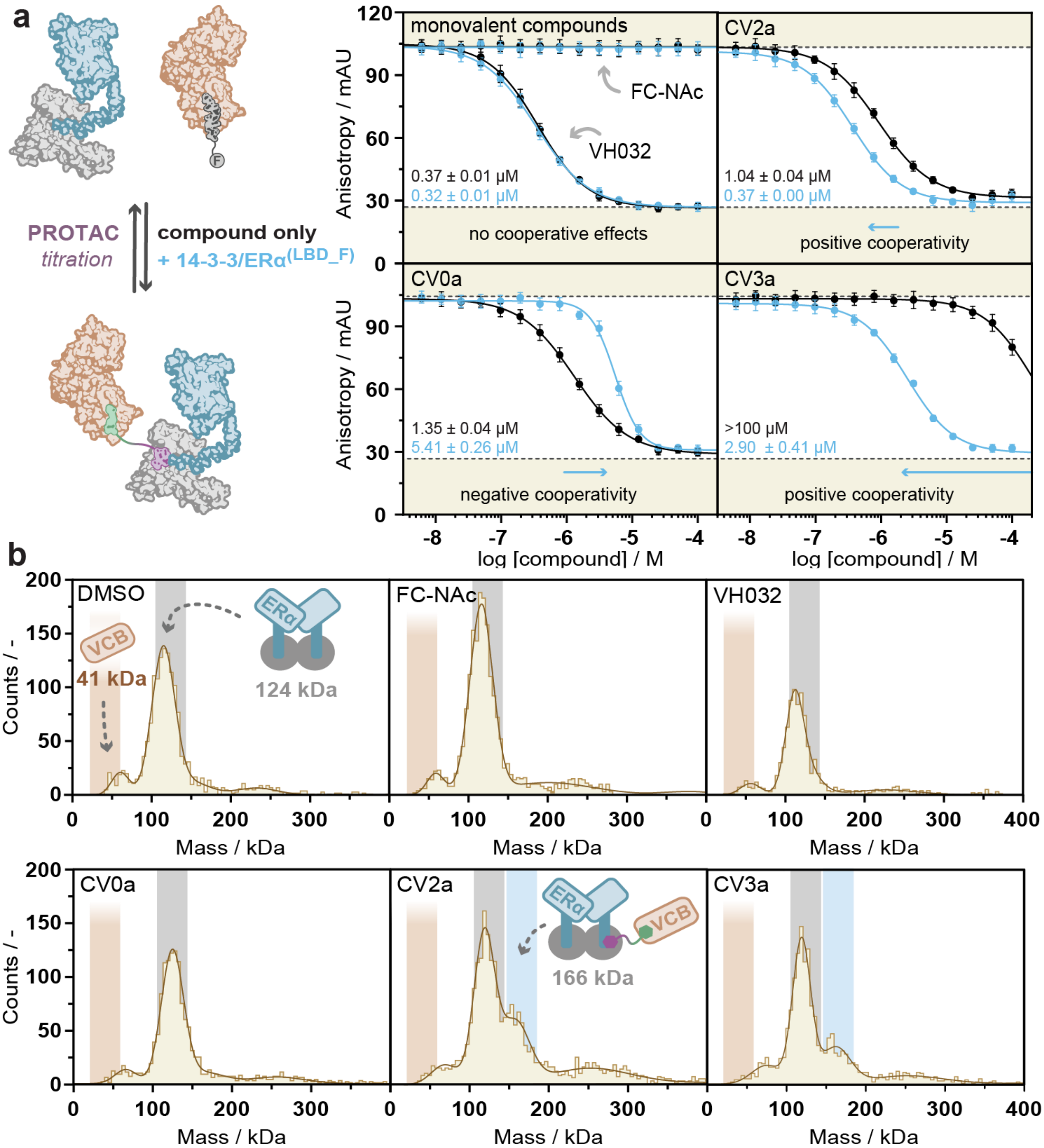

Figure S8. Cooperative VHL recruitment extends to the 14-3-3/ERα$^{(LBD_F)}$ protein-protein complex. (a) FA assay data of ^{MG}PROTAC (or monovalent compound) titration to 25 nM fluorescently labeled HIF1α peptide and 50 nM VCB protein complex in the absence (black) or presence of 10 µM 14-3-3/ERα$^{(LBD_F)}$ complex (blue). IC_{50} values of displacement curves are indicated. Data are presented as mean values ± standard deviations (error bars) of independent scientific replicates (n = 3). (b) Mass photometry shows a single molecule of VCB binding the 14-3-3/ERα$^{(LBD_F)}$ complex. A 1:1 mixture of 14-3-3/ERα$^{(LBD_F)}$ complex (2:2 stoichiometry, 124 kDa) and the VCB complex (41 kDa) was treated with three equivalents of monovalent binder, bivalent compound or DMSO. Analysis of the histograms upon treatment of the protein mixture with DMSO, FC-NAc, VH032, or negatively cooperative CV0a showed two distinct peaks, corresponding to the VCB complex and the 14-3-3/ERα$^{(LBD_F)}$ complex. In contrast, treatment with CV2a and CV3a promoted the formation of a larger complex consistent with a molecular weight of ~166 kDa, corresponding to a 2:2:1 14-3-3/ ERα$^{(LBD_F)}$ /VCB stoichiometry, suggesting an asymmetric binding of VCB to the complex. Source data are provided in the Source Data file.

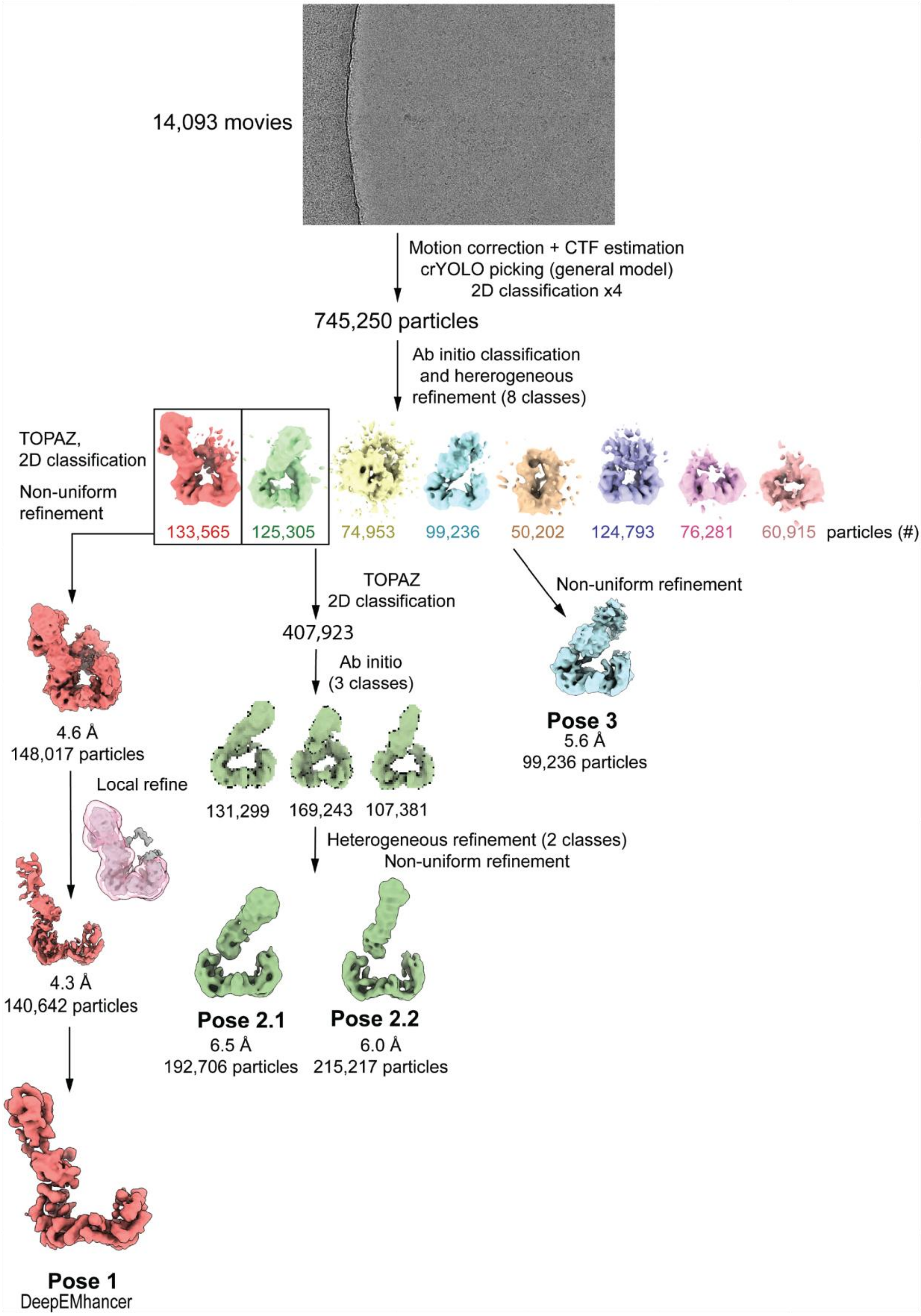

Figure S9. Cryo-EM analysis workflow of the 14-3-3/ERα$^{(LBD_F)}$/CV2a/VCB assembly.

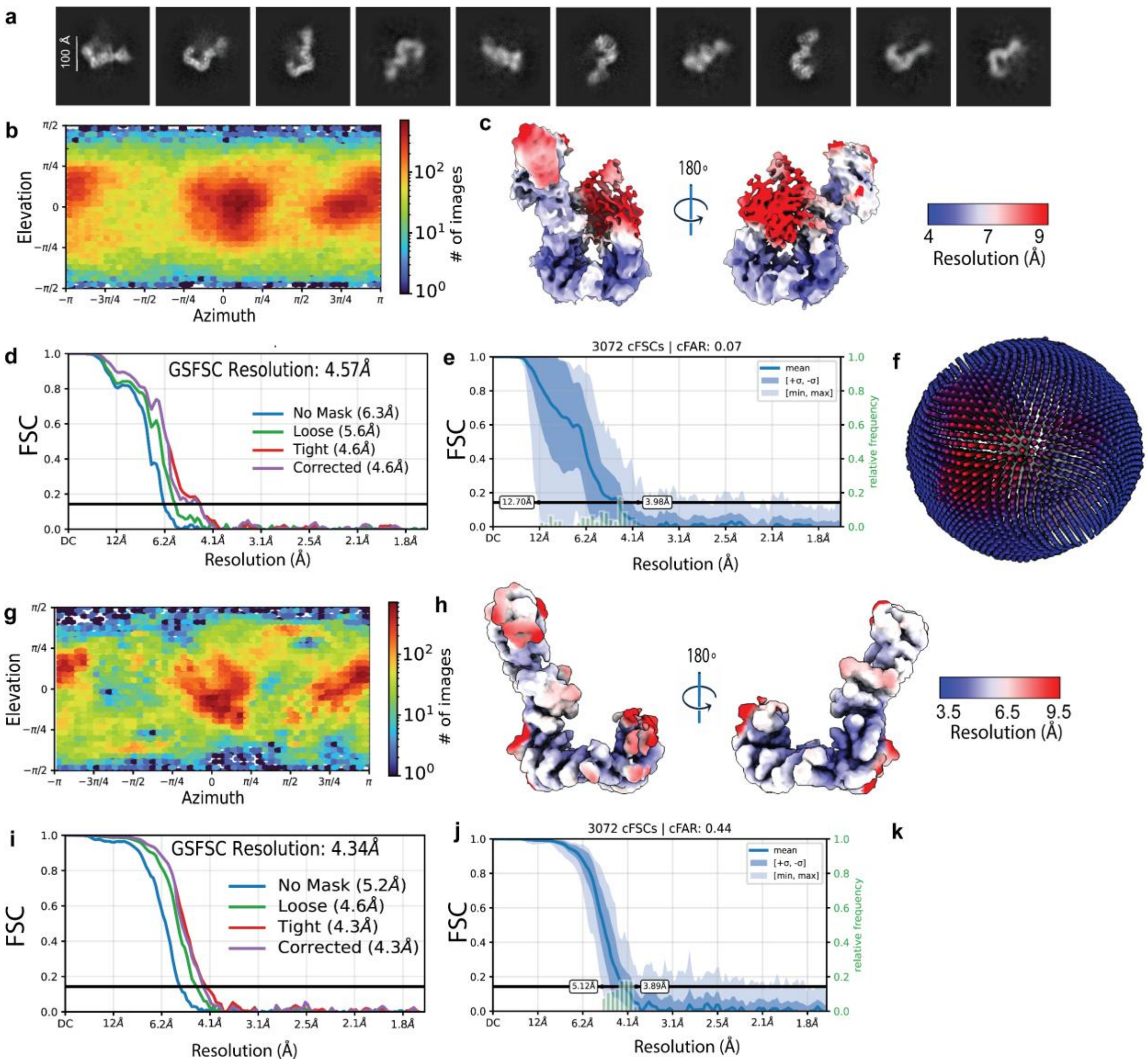

Figure S10. (a) Representative 2D classes associated with particles from Pose 1. (b) 2D viewing distribution for the Pose 1 volume before local refinement. (c) Local resolution estimation for the Pose 1 volume before local refinement. (d) Gold-standard Fourier shell correlation plot at 0.143 for the Pose 1 volume before local refinement. (e) Conical FSC distribution range for the Pose 1 volume before local refinement. (f) 3D viewing distribution for the Pose 1 volume before local refinement. (g) 3D viewing distribution for the Pose 1 volume after local refinement. (h) Local resolution estimation for the Pose 1 volume after local refinement. (i) Gold-standard Fourier shell correlation plot at 0.143 for the Pose 1 volume after local refinement. (j) Conical FSC distribution range for the Pose 1 volume after local refinement. (k) 3D viewing distribution for the Pose 1 volume after local refinement.

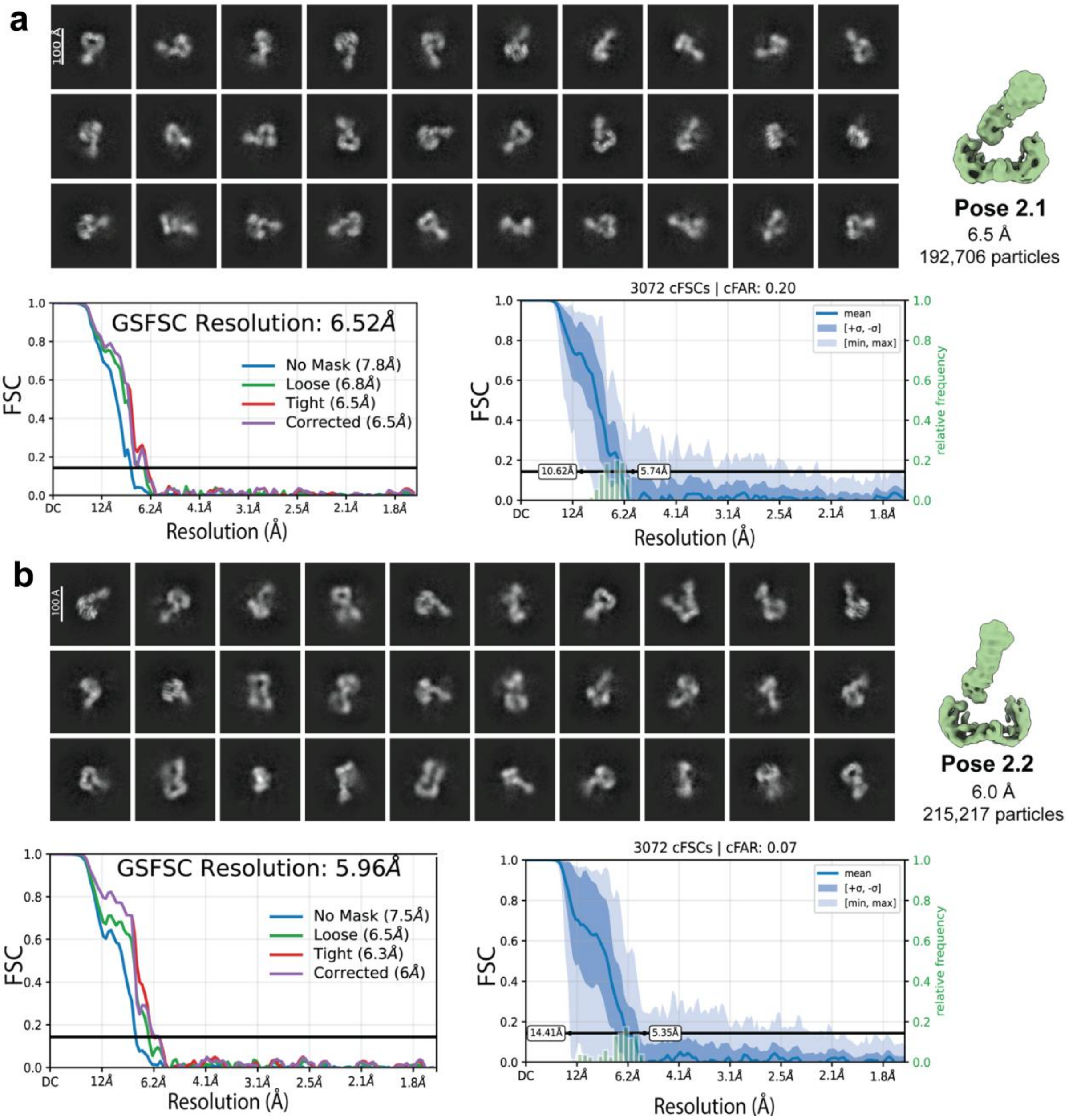

Figure S11. (a) Pose 2.1 Representative 2D classes, Gold-standard Fourier shell correlation plot at 0.143 and conical FSC distribution range. (b) Pose 2.2 Representative 2D classes, Gold-standard Fourier shell correlation plot at 0.143 and conical FSC distribution range.

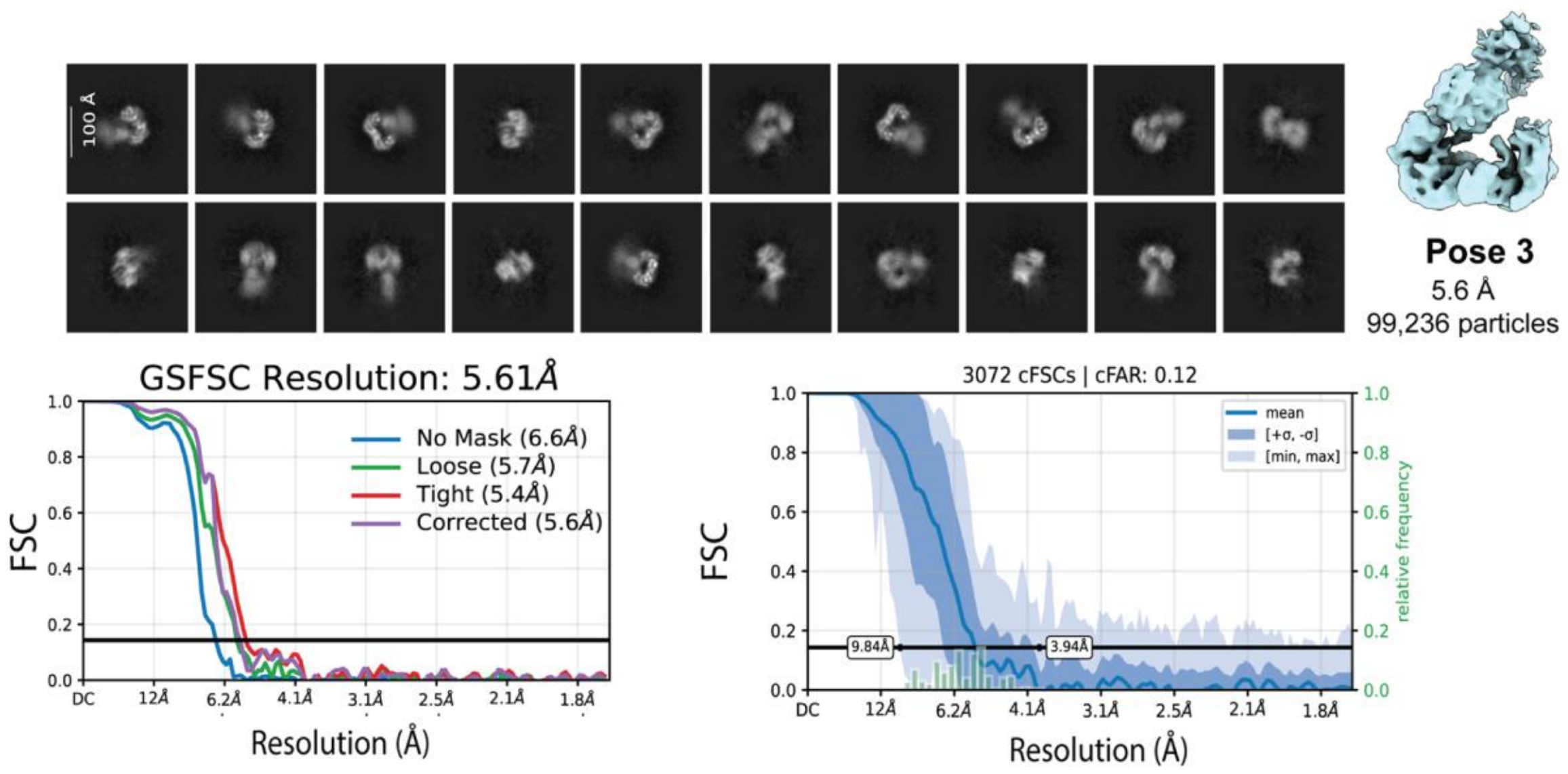

Figure S12. Pose 3 Representative 2D classes, Gold-standard Fourier shell correlation plot at 0.143 and conical FSC distribution range.

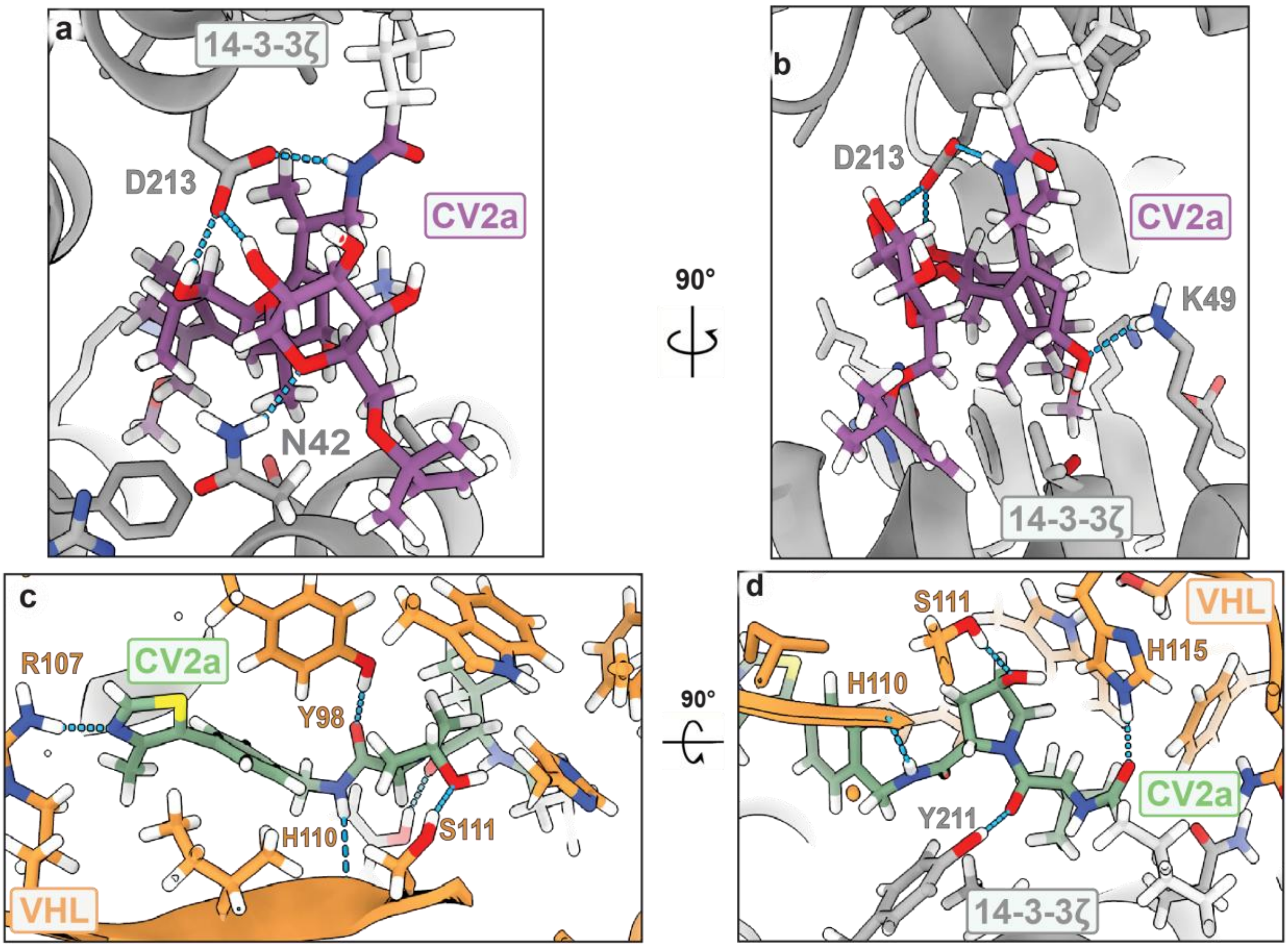

Figure S13. (a-b) Highlighted protein-ligand interactions between the fusicoccin ligand of CV2a and 14-3-3. The FC-NAc ligand of CV2a binds within the composite binding interface formed between binding groove of 14-3-3 and the C-terminal phosphorylated 14-3-3 binding motif of ERα. (c-d) Highlighted protein-ligand interactions between the VH032 ligand of CV2a, VHL, and 14-3-3.

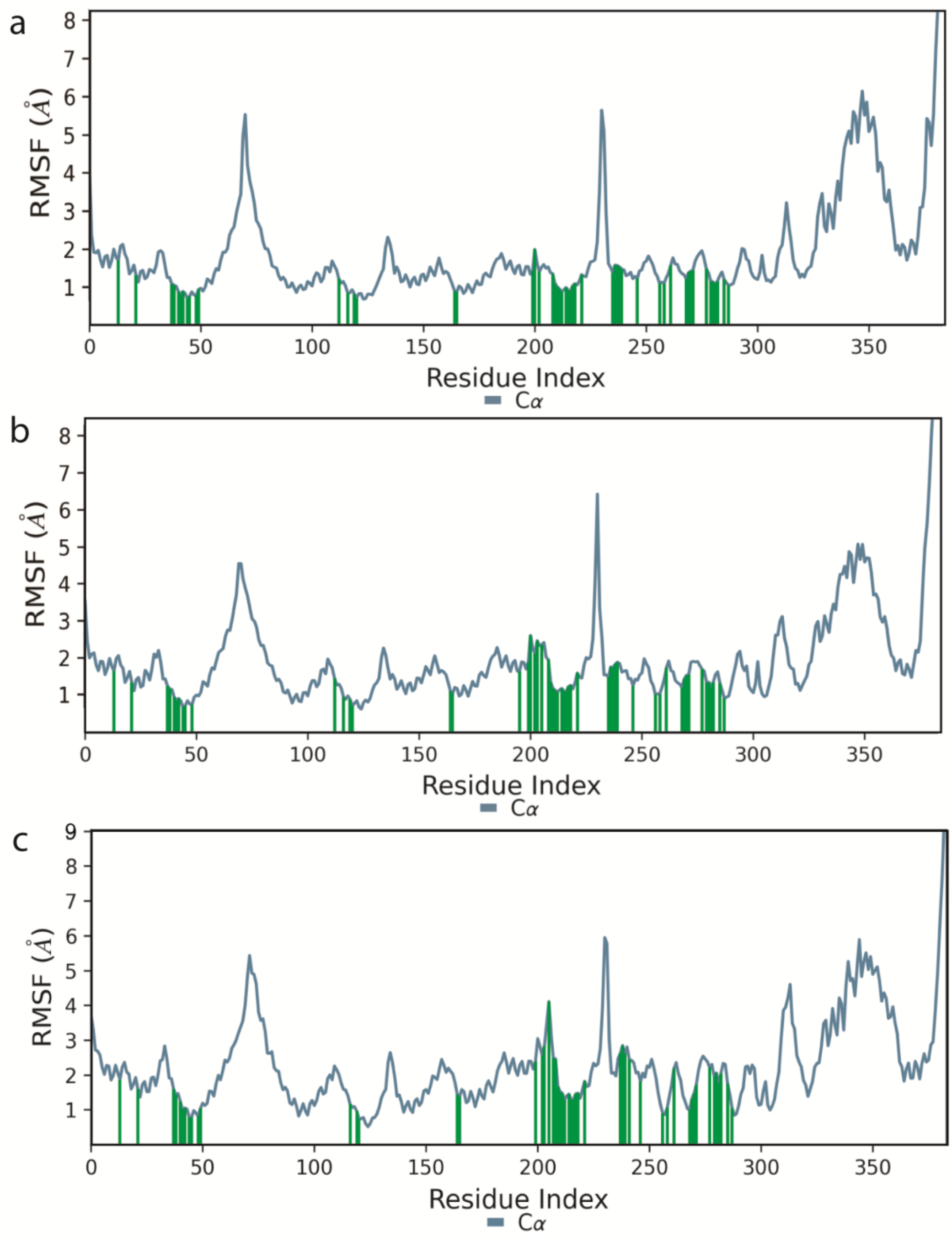

Figure S14. Root mean square fluctuation (RMSF) of the Ca carbons in the protein complex during the MD simulation. Panels (a), (b) and (c) correspond to MD replicates 1, 2 and 3, respectively. The protein residues that interact with the CV2a [MG]PROTAC are marked with green-colored vertical bars.

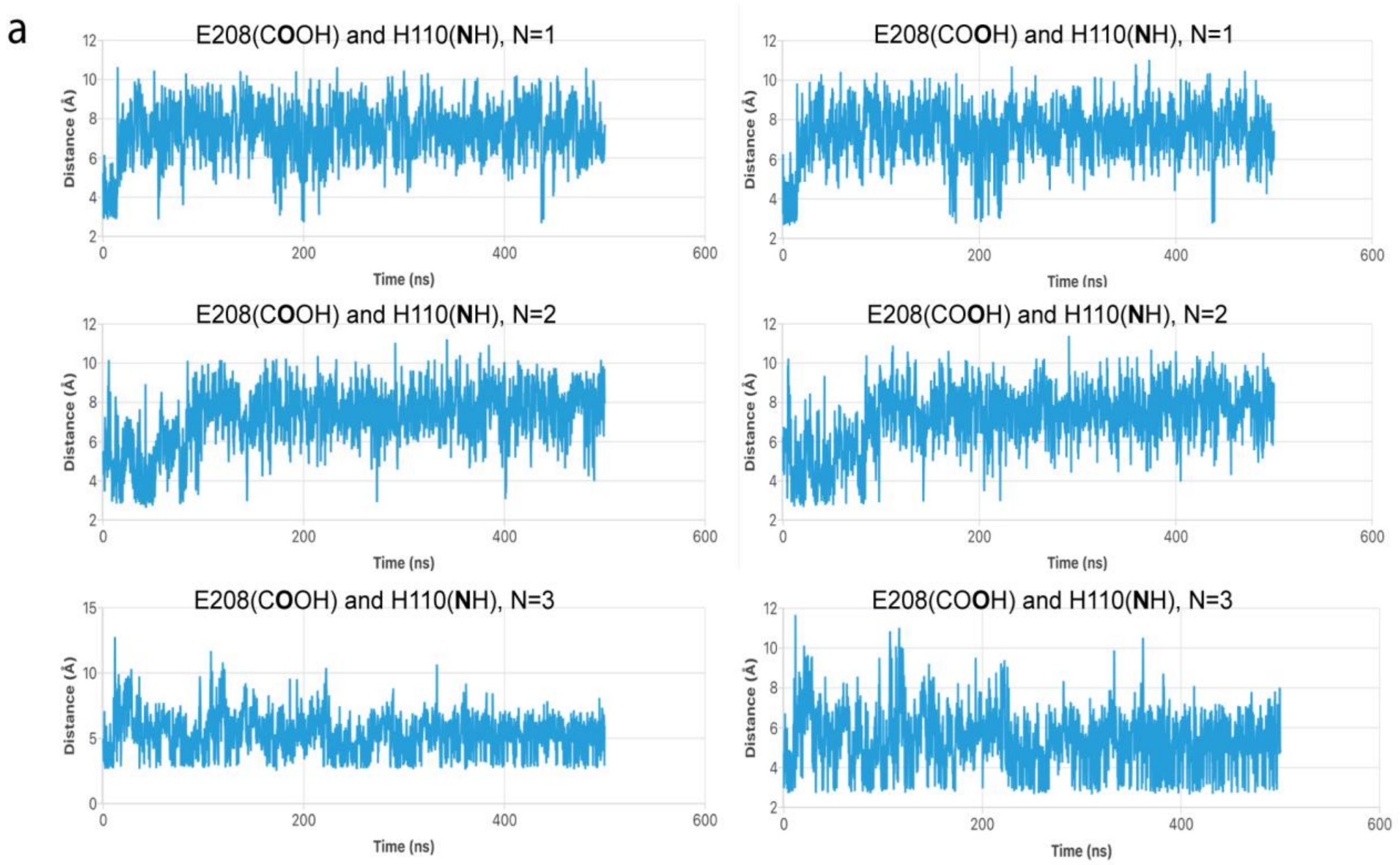

Figure S15. Time-dependent distance diagrams for the heavy atoms throughout the course of the 500 ns MD simulation, for residues (a) 14-3-3ζ Glu208 side-chain COOH oxygens and VHL β4 sheet His110 NH nitrogen.

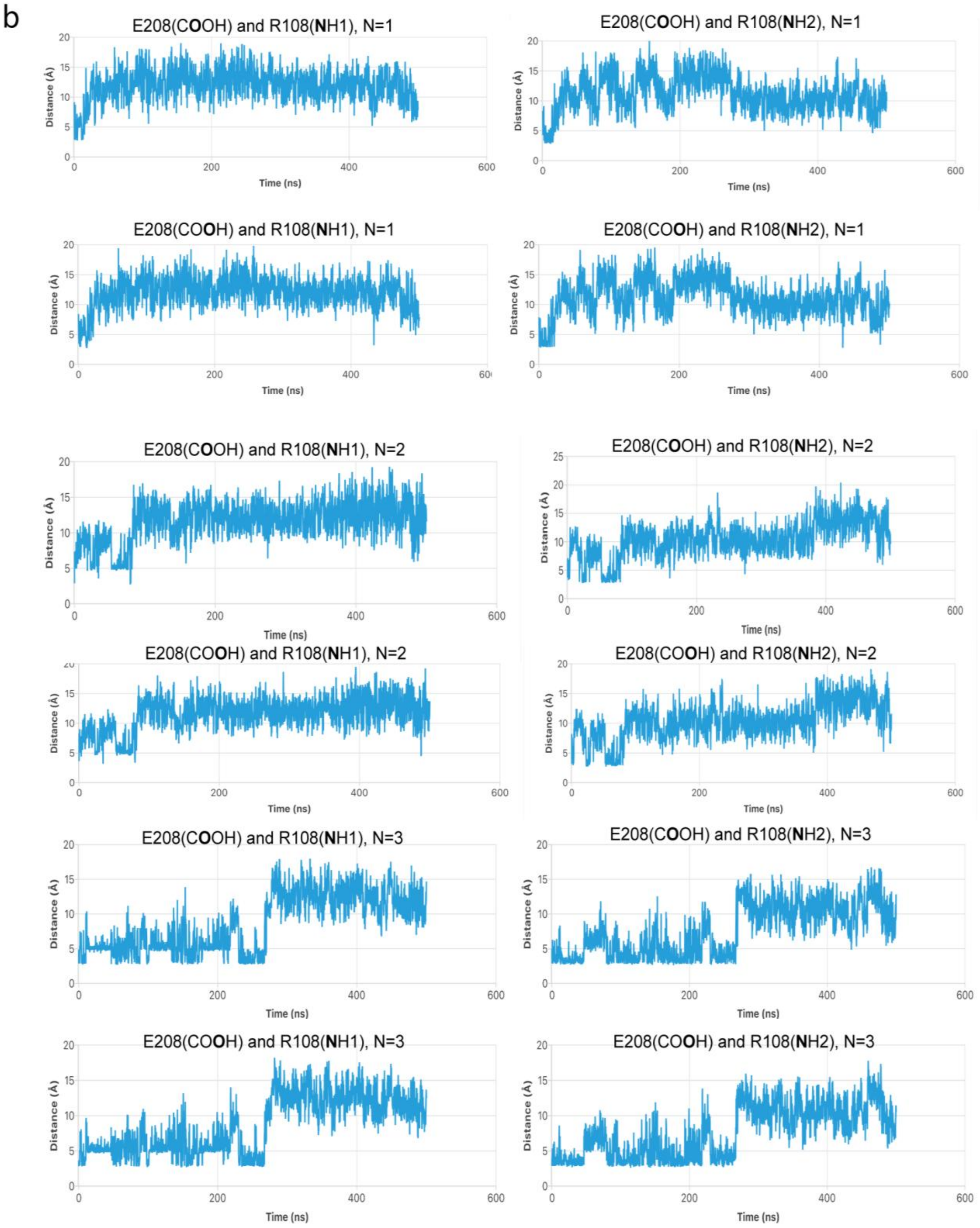

Figure S15 [continued]. (b) 14-3-3ζ Glu208 side-chain COOH oxygen atoms and VHL β4 sheet Arg108 side-chain NH2 nitrogen atoms.

c

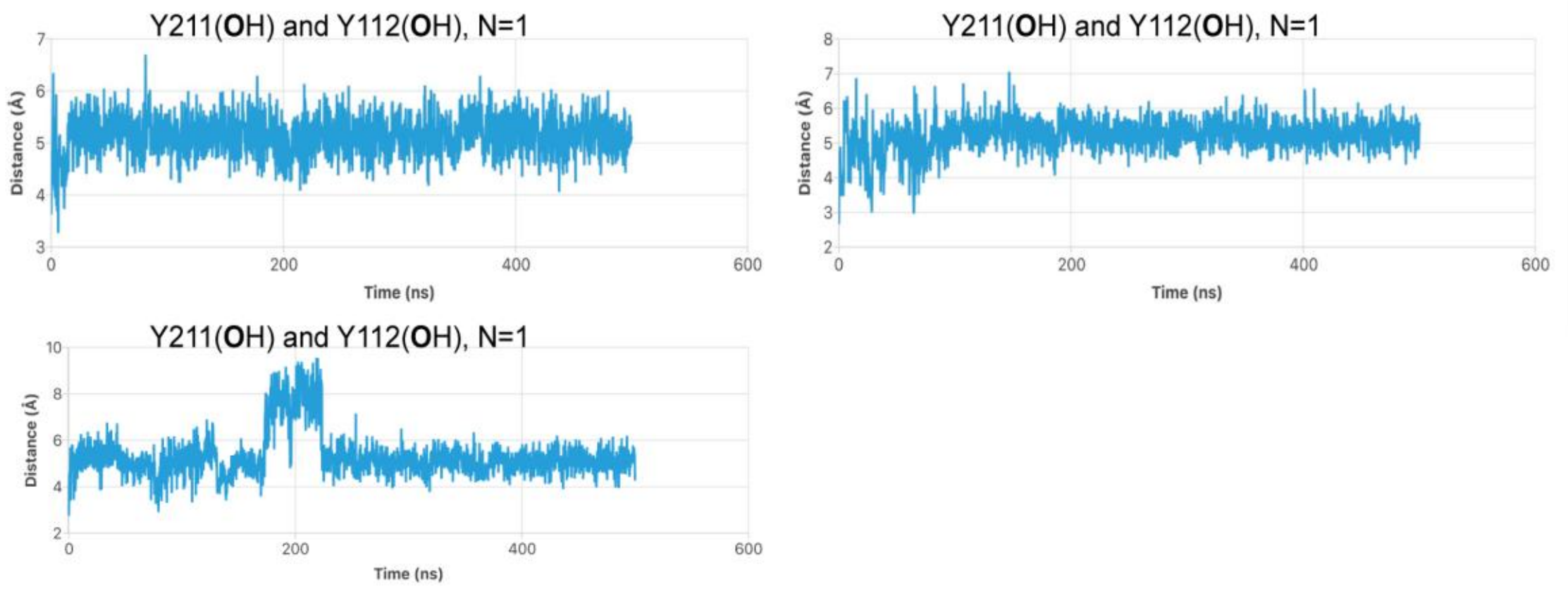

Y211(OH) and Y112(OH), N=1
Y211(OH) and Y112(OH), N=1
Y211(OH) and Y112(OH), N=1
Distance (Å)
Time (ns)

d

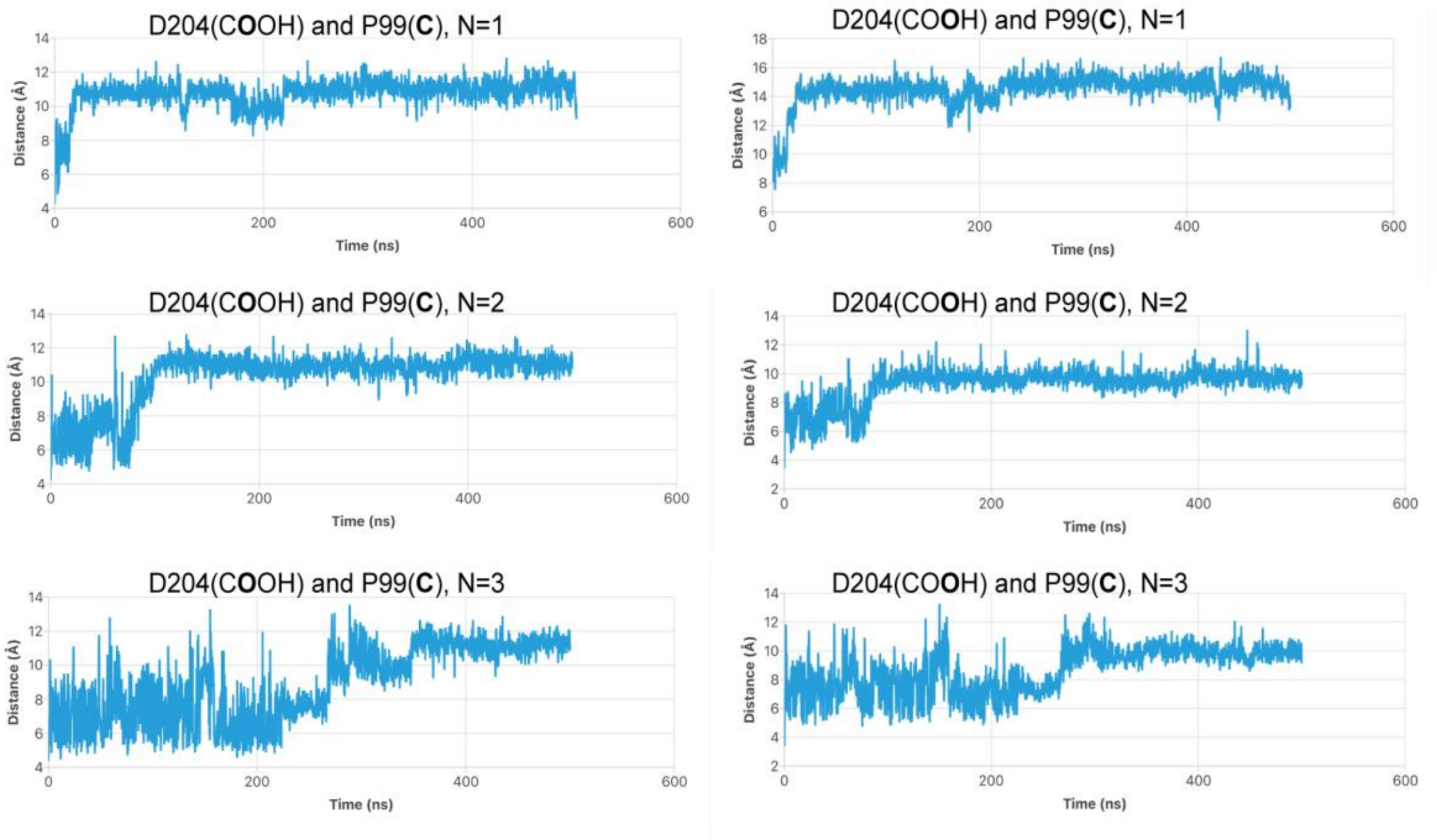

D204(COOH) and P99(C), N=1
D204(COOH) and P99(C), N=1
D204(COOH) and P99(C), N=2
D204(COOH) and P99(C), N=2
D204(COOH) and P99(C), N=3
D204(COOH) and P99(C), N=3
Distance (Å)
Time (ns)

e

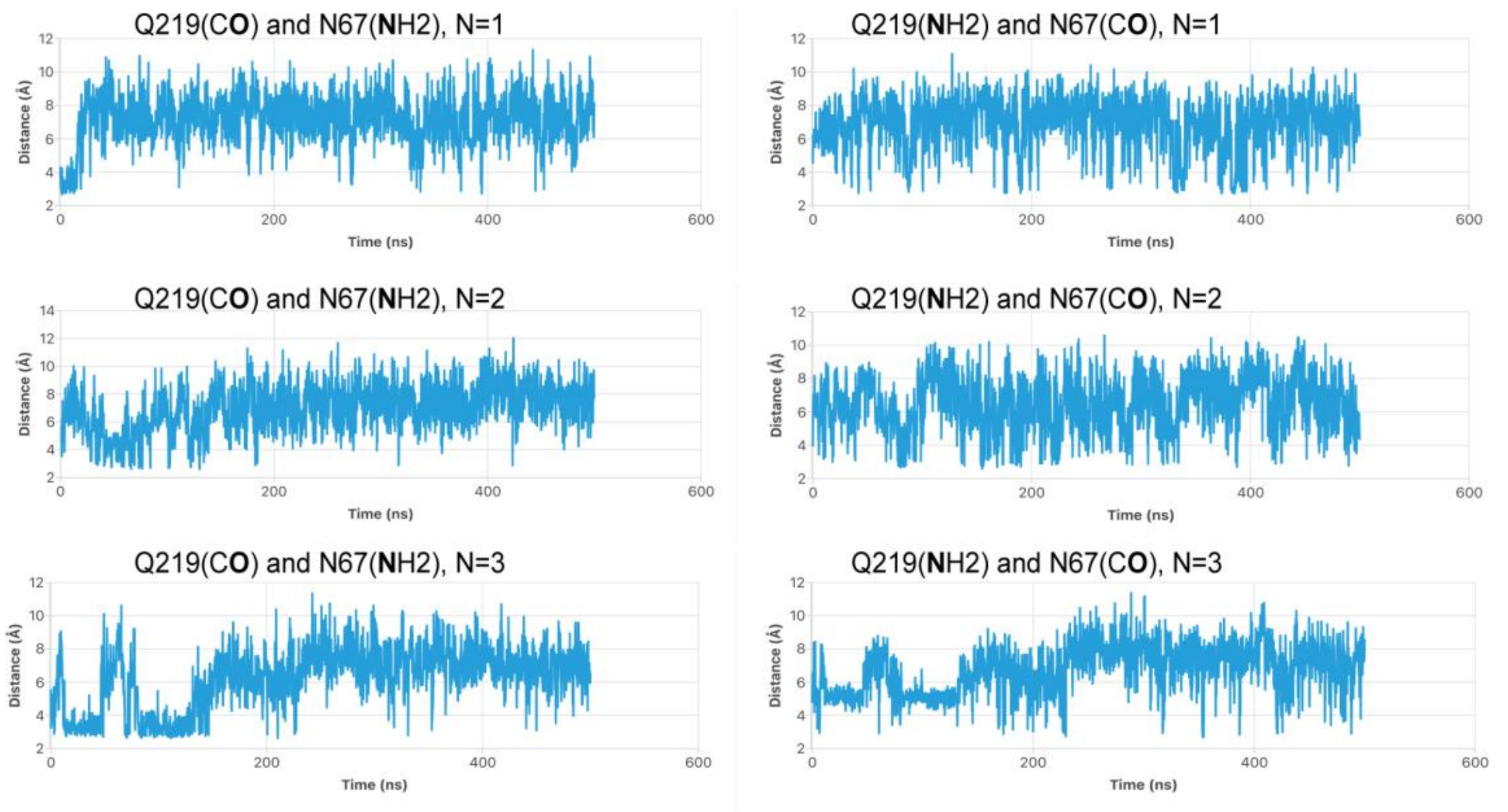

Q219(CO) and N67(NH2), N=1
Q219(NH2) and N67(CO), N=1
Q219(CO) and N67(NH2), N=2
Q219(NH2) and N67(CO), N=2
Q219(CO) and N67(NH2), N=3
Q219(NH2) and N67(CO), N=3
Distance (Å)
Time (ns)

Figure S15 [continued]. (c) α9 helix 14-3-3ζ Tyr211 phenolic OH oxygen and VHL β4 sheet Tyr112 phenolic OH oxygen, (d) 14-3-3ζ Asp204 side-chain COOH oxygen atoms and VHL Pro99 backbone carbonyl carbon atom, (e) 14-3-3ζ Gln219 and VHL Asn67 side-chain amide CO oxygen atoms and NH2 nitrogen atoms.

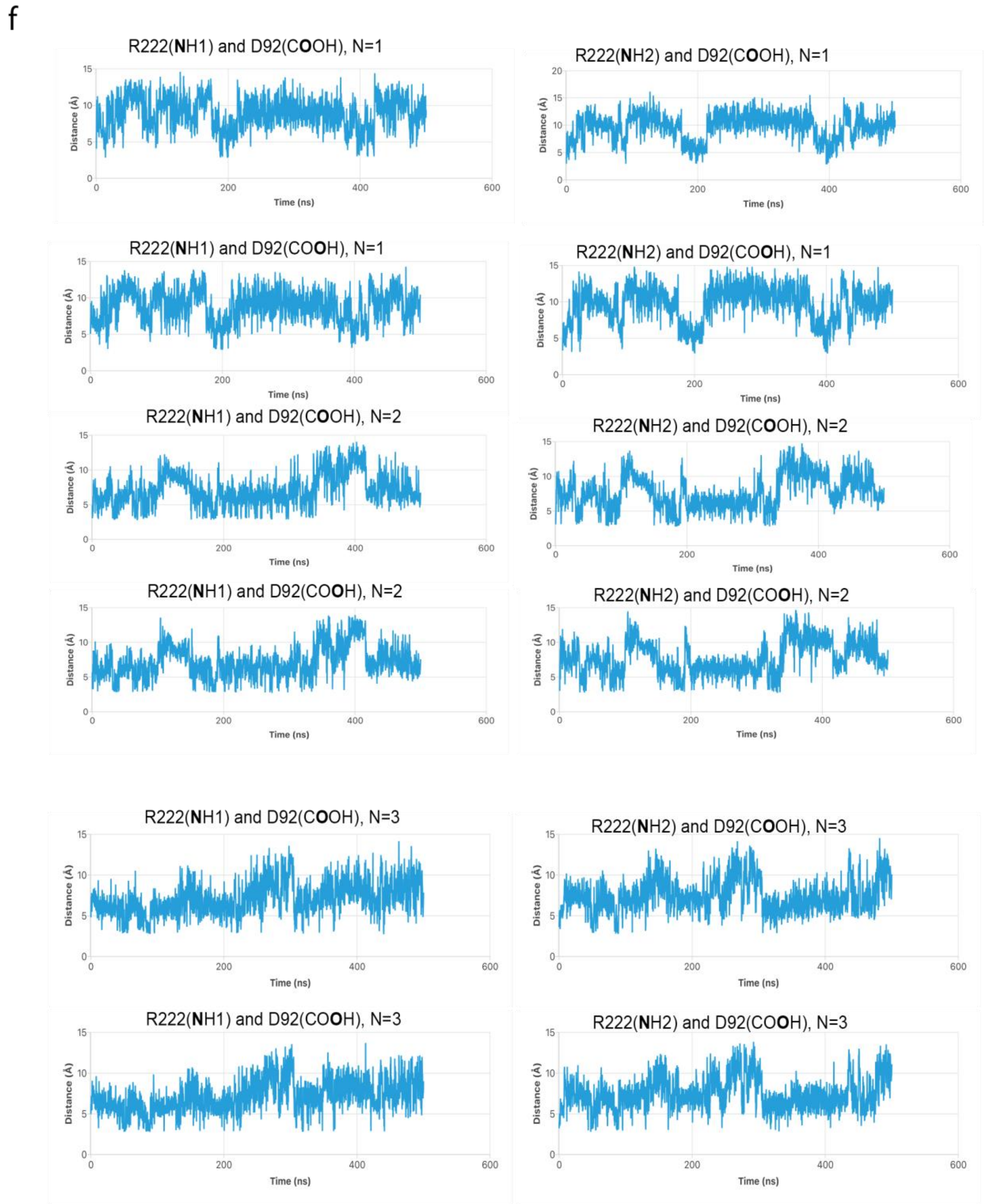

Figure S15 [continued]. (f) α9 helix 14-3-3ζ Arg222 side-chain NH2 nitrogen atoms and VHL Asp92 side-chain COOH oxygen atoms.

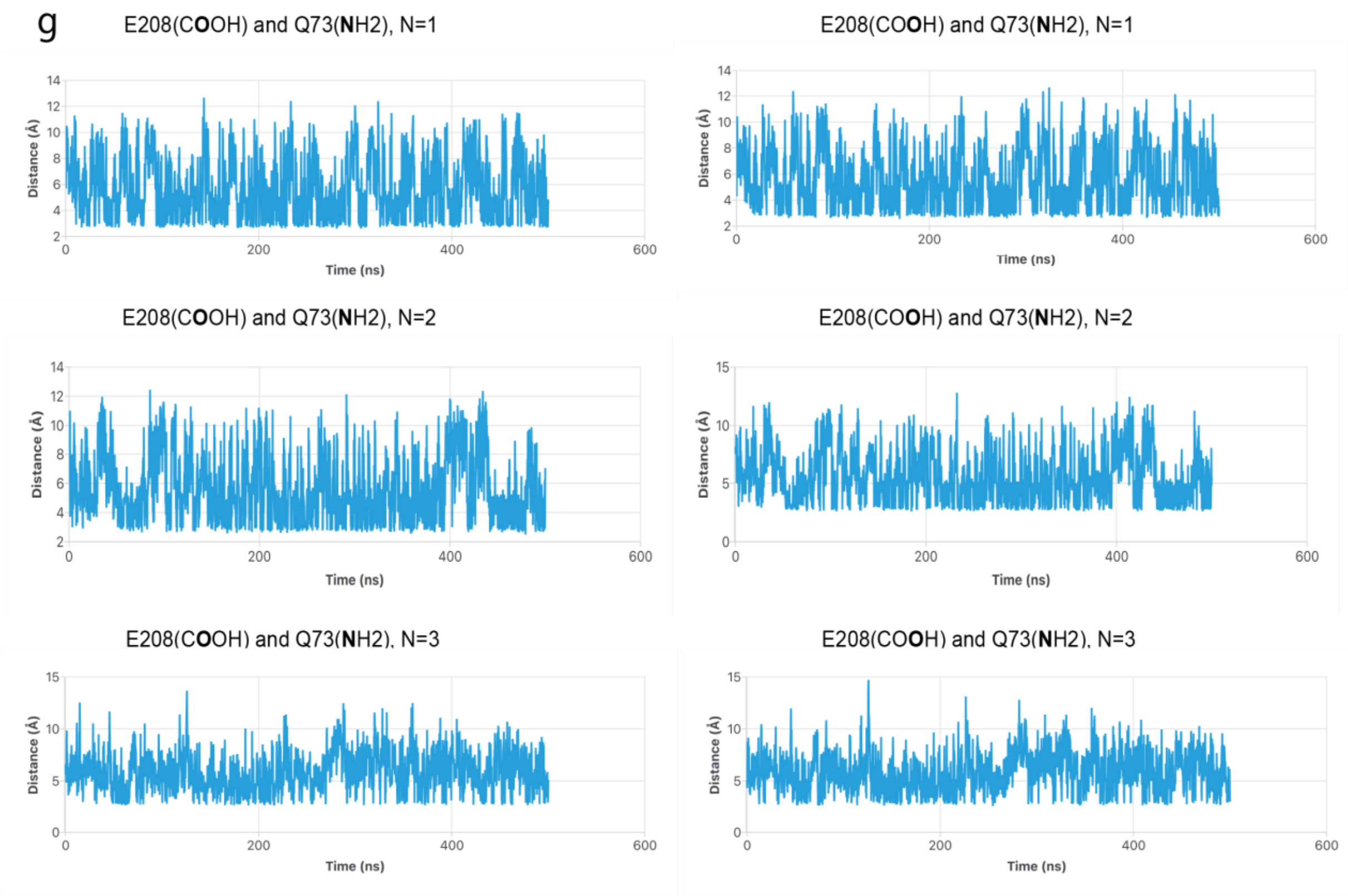

Figure S15 [continued]. (g)14-3-3ζ Glu208 side-chain COOH oxygen atoms and VHL β4 sheet Gln73 side-chain NH2 nitrogen atom.

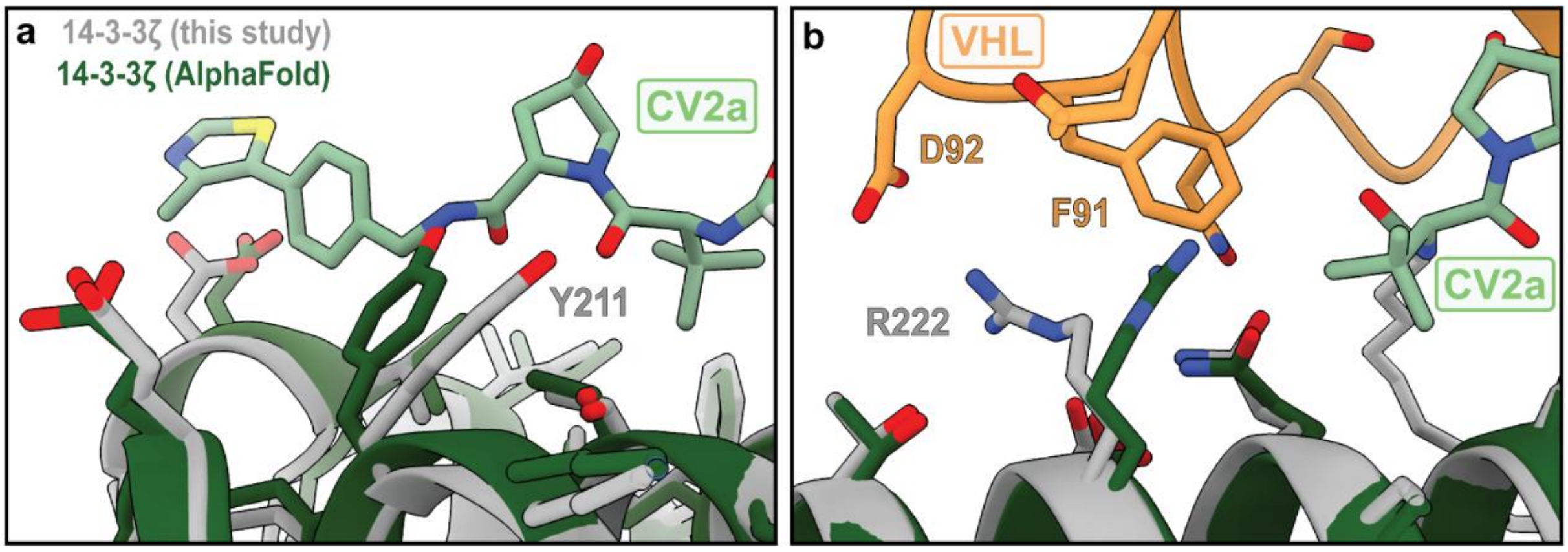

Figure S16. Superposition of apo 14-3-3 zeta (AlphaFold, dark green) with 14-3-3 zeta from the model in this study (silver). (a) Shows Tyr211 rotate to accommodate the VH032 moiety of CV2a. (b) Shows R222 flip to avoid clashing with Phe91 and to form contacts with Asp92.

```
14-3-3β (P31946)  3    MDKSELVQKAKLAEQAERYDDMAAAMKAVTEQGHELSNEERNLLSVAYKNVVGARRSSWR  62
14-3-3ε (P62258)  3     DREDLVYQAKLAEQAERYDEMVESMKKVAGMDVELTVEERNLLSVAYKNVIGARRASWR  61
14-3-3η (Q04917)  3     DREQLLQRARLAEQAERYDDMASAMKAVTELNEPLSNEDRNLLSVAYKNVVGARRSSWR  61
14-3-3γ (P61981)  2    VDREQLVQKARLAEQAERYDDMAAAMKNVTELNEPLSNEERNLLSVAYKNVVGARRSSWR  61
14-3-3σ (P31947)  1    MERASLIQKAKLAEQAERYEDMAAFMKGAVEKGEELSCEERNLLSVAYKNVVGGQRAAWR  60
14-3-3θ (P27348)  1    MEKTELIQKAKLAEQAERYDDMATCMKAVTEQGAELSNEERNLLSVAYKNVVGGRRSAWR  60
14-3-3ζ (P63104)  1    MDKNELVQKAKLAEQAERYDDMAACMKSVTEQGAELSNEERNLLSVAYKNVVGARRSSWR  60
                          helix 1       helix 2             helix 3

14-3-3β (P31946)  63   VISSIEQKT--ER--NEKKQQMGKEYREKIEAELQDICNDVLELLDKYLIPNA--T--QP  114
14-3-3ε (P62258)  62   IISSIEQKE--ENKGGEDKLKMIREYRQMVETELKLICCDILDVLDKHLIPAA--N--TG  115
14-3-3η (Q04917)  62   VISSIEQKTMADG--NEKKLEKVKAYREKIEKELETVCNDVLSLLDKFLIKNC--NDFQY  117
14-3-3γ (P61981)  62   VISSIEQKTSADG--NEKKIEMVRAYREKIEKELEAVCQDVLSLLDNYLIKNCSET--QY  117
14-3-3σ (P31947)  61   VLSSIEQKSNEEG--SEEKGPEVREYREKVETELQGVCDTVLGLLDSHLIKEA--G--DA  114
14-3-3θ (P27348)  61   VISSIEQKT--DT--SDKKLQLIKDYREKVESELRSICTTVLELLDKYLIANA--T--NP  112
14-3-3ζ (P63104)  61   VVSSIEQKT--EG--AEKKQQMAREYREKIETELRDICNDVLSLLEKFLIPNA--S--QA  112
                                          helix 4

14-3-3β (P31946)  115  ESKVFYLKMKGDYFRYLSEVASGDNKQTTVSNSQQAYQEAFEISKKEMQPTHPIRLGLAL  174
14-3-3ε (P62258)  116  ESKVFYYKMKGDYHRYLAEFATGNDRKEAAENSLVAYKAASDIAMTELPPTHPIRLGLAL  175
14-3-3η (Q04917)  118  ESKVFYLKMKGDYYRYLAEVASGEKKNSVVEASEAAYKEAFEISKEQMQPTHPIRLGLAL  177
14-3-3γ (P61981)  118  ESKVFYLKMKGDYYRYLAEVATGEKRATVVESSEKAYSEAHEISKEHMQPTHPIRLGLAL  177
14-3-3σ (P31947)  115  ESRVFYLKMKGDYYRYLAEVATGDDKKRIIDSARSAYQEAMDISKKEMPPTNPIRLGLAL  174
14-3-3θ (P27348)  113  ESKVFYLKMKGDYFRYLAEVACGDDRKQTIDNSQGAYQEAFDISKKEMQPTHPIRLGLAL  172
14-3-3ζ (P63104)  113  ESKVFYLKMKGDYYRYLAEVAAGDDKKGIVDQSQQAYQEAFEISKKEMQPTHPIRLGLAL  172
                          helix 5                  helix 6              helix 7

                                                        204  208 211    219 222
14-3-3β (P31946)  175  NFSVFYYEILNSPEKACSLAKTAFDEAIAELDTLNEESYKDSTLIMQLLRDNLTLWTSEN  234
14-3-3ε (P62258)  176  NFSVFYYEILNSPDRACRLAKAAFDDAIAELDTLSEESYKDSTLIMQLLRDNLTLWTSDM  235
14-3-3η (Q04917)  178  NFSVFYYEIQNAPEQACLLAKQAFDDAIAELDTLNEDSYKDSTLIMQLLRDNLTLWTSDQ  237
14-3-3γ (P61981)  178  NYSVFYYEIQNAPEQACHLAKTAFDDAIAELDTLNEDSYKDSTLIMQLLRDNLTLWTSDQ  237
14-3-3σ (P31947)  175  NFSVFHYEIANSPEEAISLAKTTFDEAMADLHTLSEDSYKDSTLIMQLLRDNLTLWTADN  234
14-3-3θ (P27348)  173  NFSVFYYEILNNPELACTLAKTAFDEAIAELDTLNEDSYKDSTLIMQLLRDNLTLWTSDS  232
14-3-3ζ (P63104)  173  NFSVFYYEILNSPEKACSLAKTAFDEAIAELDTLSEESYKDSTLIMQLLRDNLTLWTSDT  232
                                      helix 8                 helix 9

14-3-3β (P31946)  235  QGDEGDAGEGEN  246
14-3-3ε (P62258)  236  QGD----GEEQN  243
14-3-3η (Q04917)  238  QDEE--AGEG    245
14-3-3γ (P61981)  238  QDDDG--GEGNN  247
14-3-3σ (P31947)  235  AGEEG         239
14-3-3θ (P27348)  233  AGEECDAAEG    242
14-3-3ζ (P63104)  233  QGDEAEAGEG    242
```

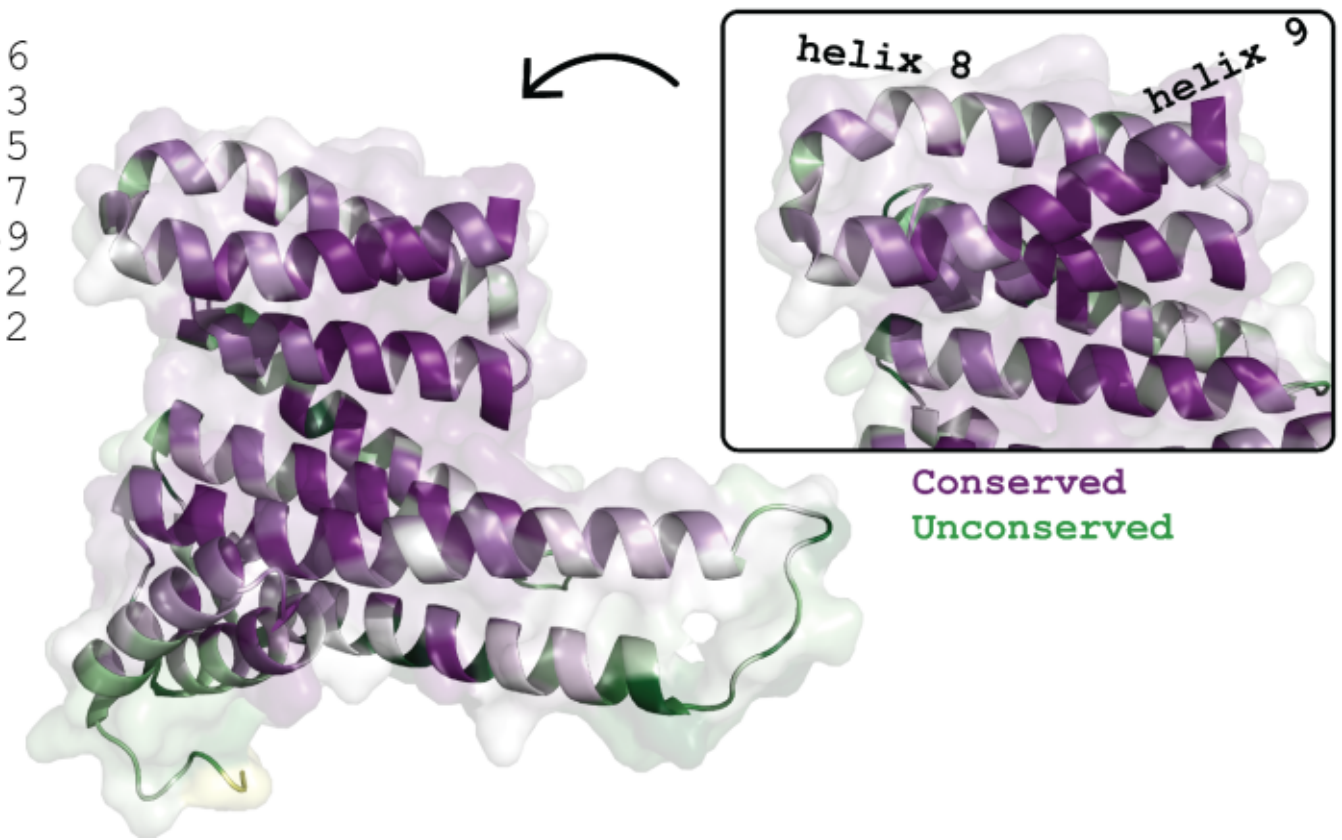

Figure S17: Sequence alignments of 14-3-3 isoforms (β, ε, η, γ, σ, θ, and ζ). The residues forming contacts with VHL in the cryo-EM model in this study are highlighted (purple). Alignment performed by BLAST.[2]

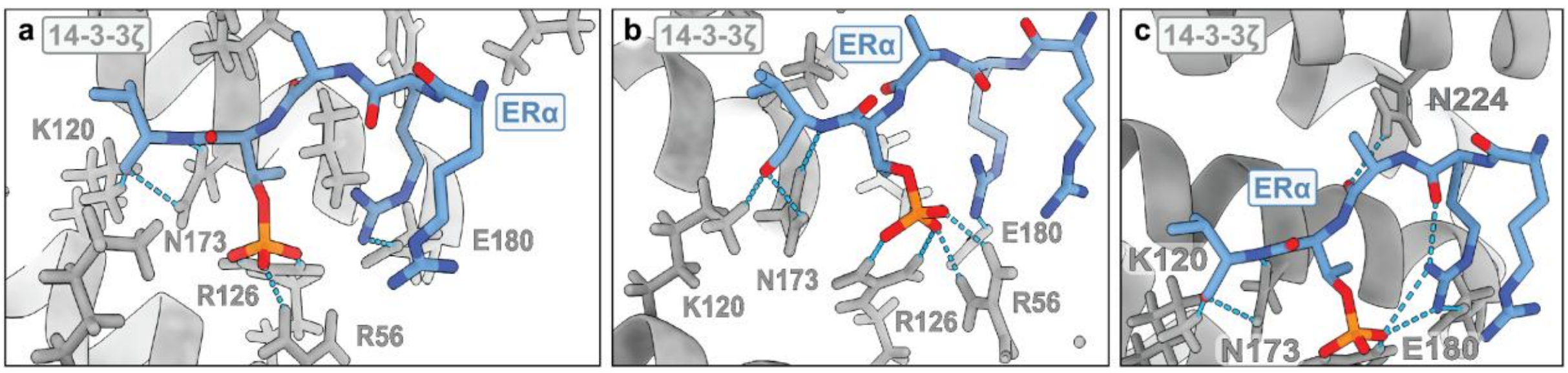

Figure S18. Highlighted protein-protein interactions between 14-3-3 and the ERα C-terminus.

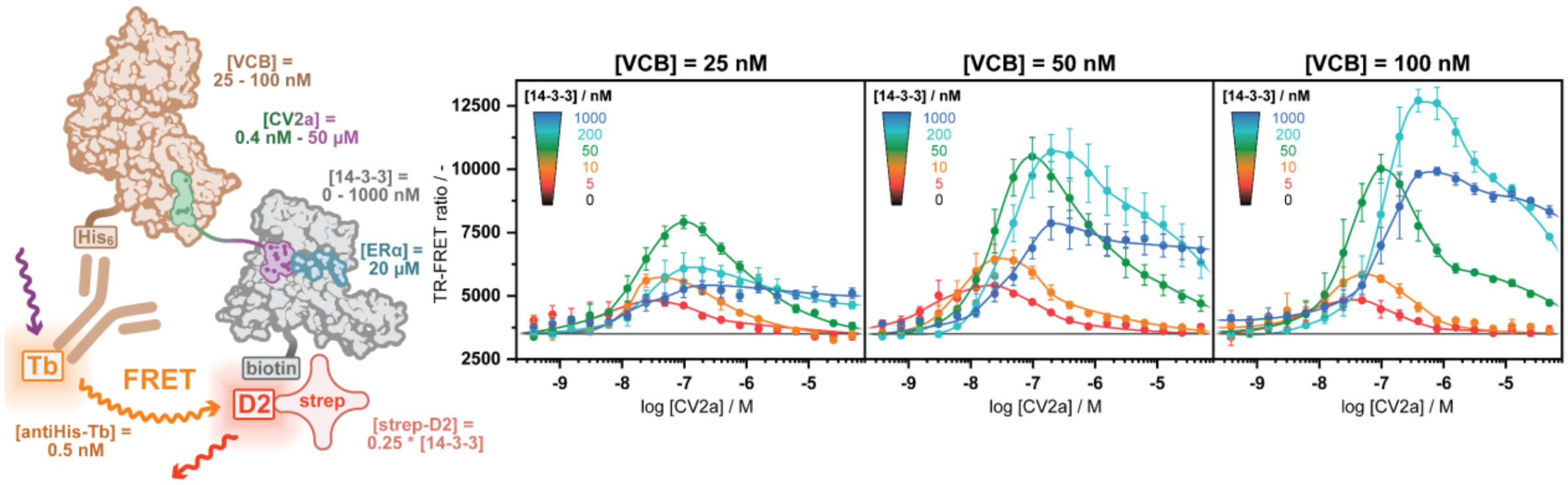

Figure S19. TR-FRET assay optimization. Concentrations of His_6-tagged VCB protein complex (25 nM – 100 nM), biotinylated 14-3-3 (0 nM – 1 µM; each concentration with 0.25 eq. Strep-D2), and CV2a (0.4 nM – 50 µM) were varied, and the concentrations of Anti-His-Tb (0.5 nM), and ERα peptide (20 µM) were kept constant. 50 nM His_6-tagged VCB, 50 nM biotinylated 14-3-3 (with 12.5 nM Strep-D2) were selected as optimal concentrations due to the large TR-FRET signal at low CV2a concentrations. Data are presented as mean values ± standard deviations (error bars) of independent scientific replicates (n = 3). Source data are provided in the Source Data file.

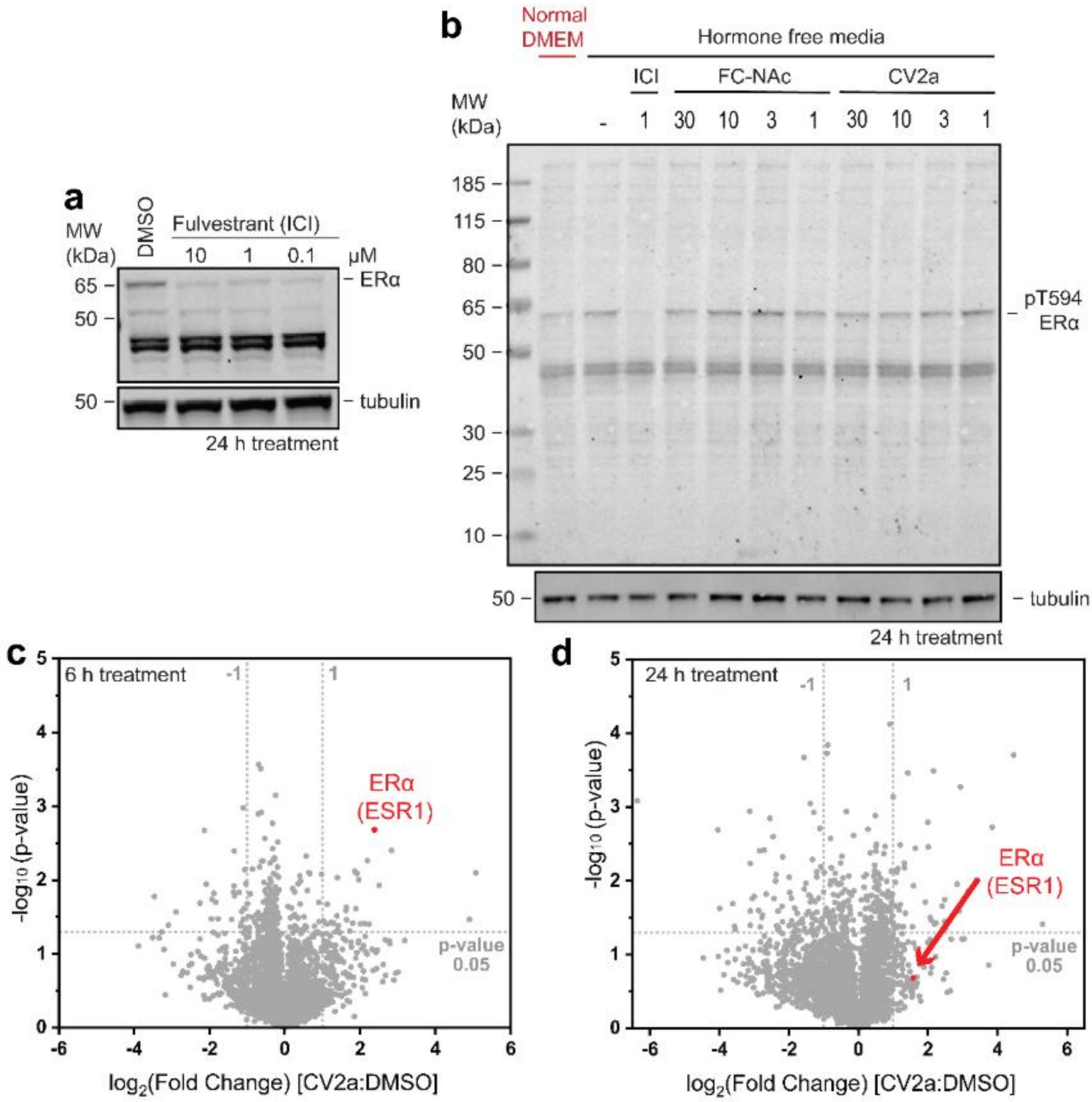

Figure S20. ERα levels after compound treatment. (a) ERα western blot of treated MCF-7 cells with Fulvestrant (ICI) (24 hour treatment). (b) pT594-ERα western blot of treated MCF-7 cells with either DMSO, ICI, FC-NAc or CV2a (24 hour treatment). (c) 14-3-3 IP-MS data of MCF-7 cells CV2a-treated for 6 hours and (d) 24 hours. Source data are for panels a and b are provided in the Source Data file.

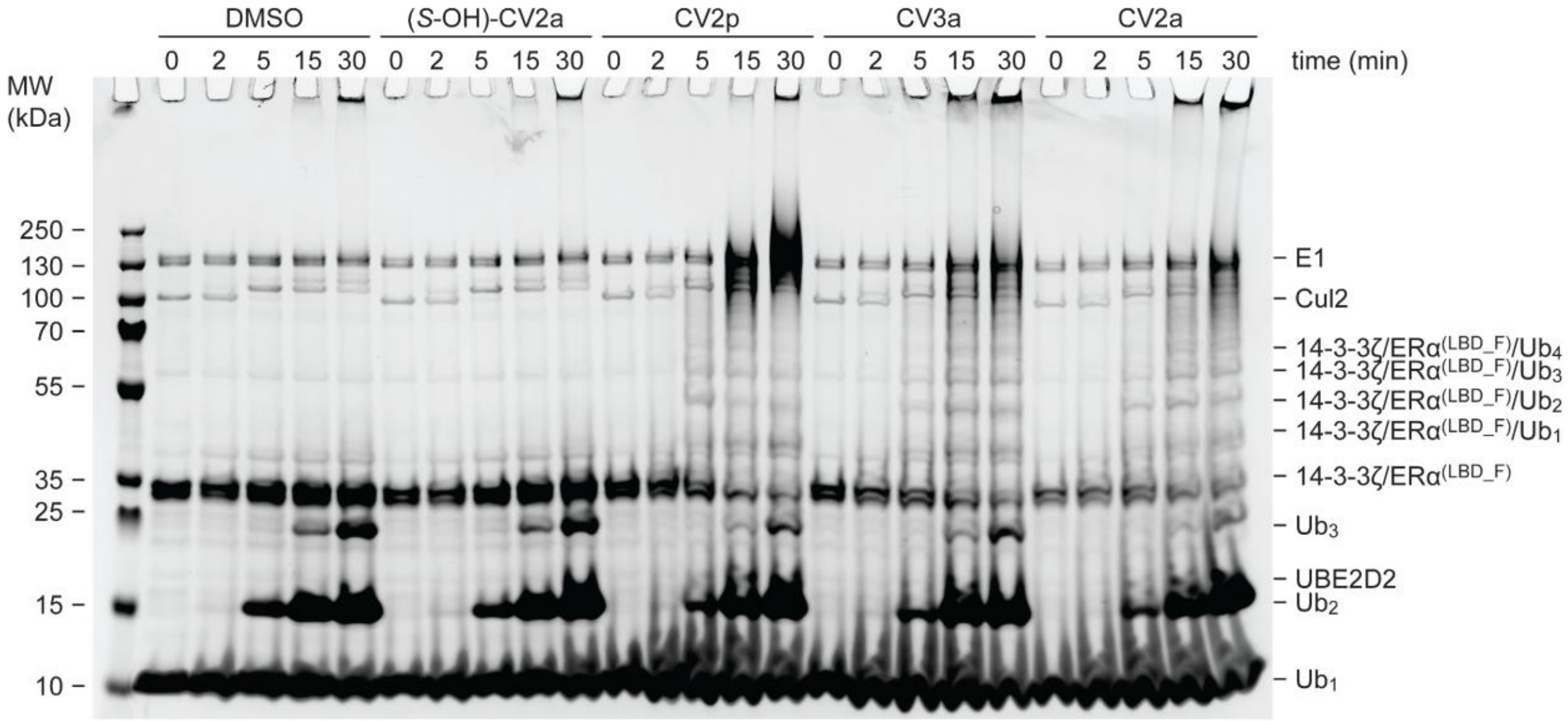

Figure S21. Full SDS-PAGE gel of in-vitro time course ubiquitination experiment from time point 0 to 30 mins, Coomassie staining. Source data are provided in the Source Data file.

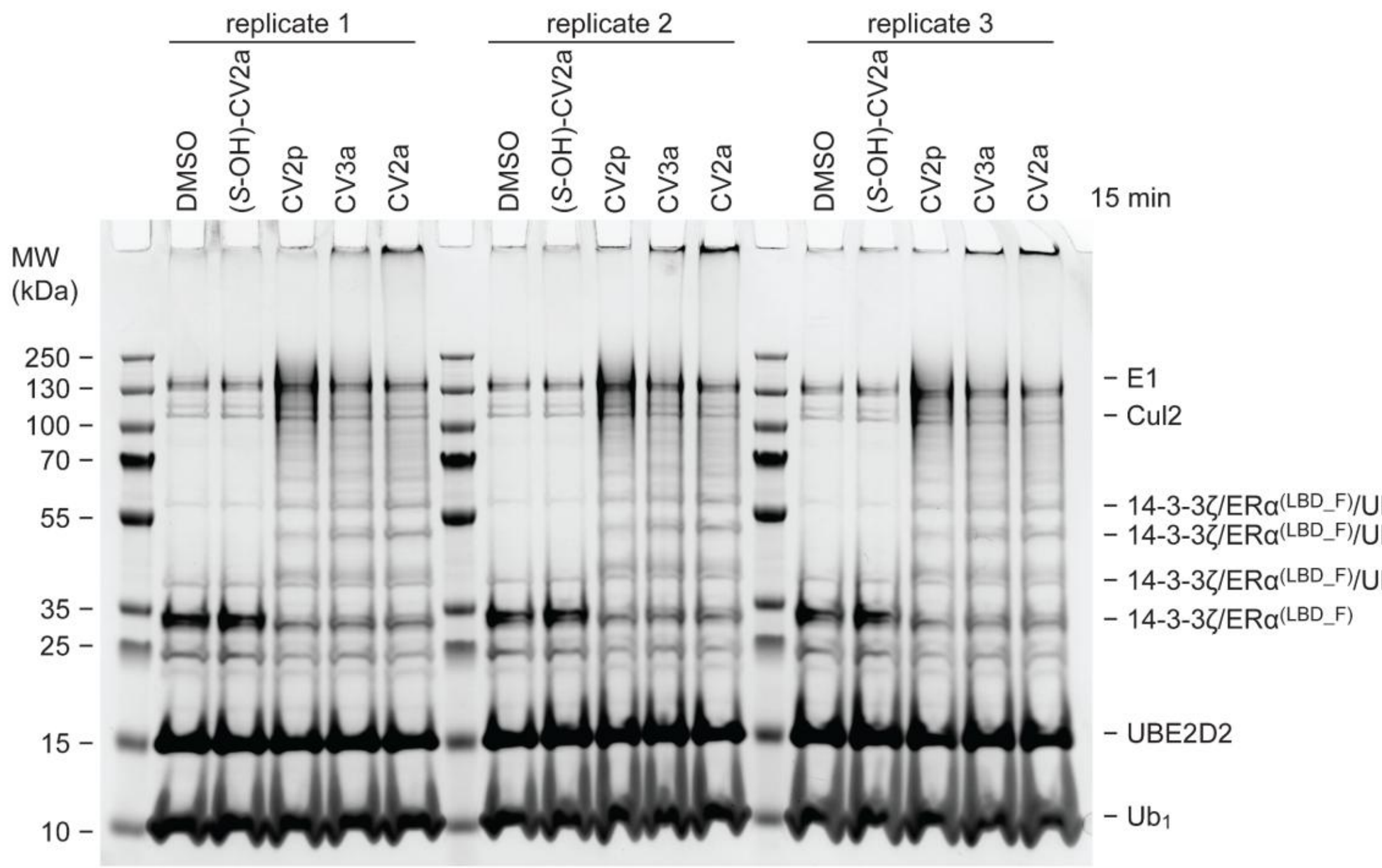

Figure S22. Full SDS-PAGE gel of in-vitro ubiquitination experiment comparing control molecules and [MG]PROTAC replicates, Coomassie staining. Overnight incubation. Source data are provided in the Source Data file.

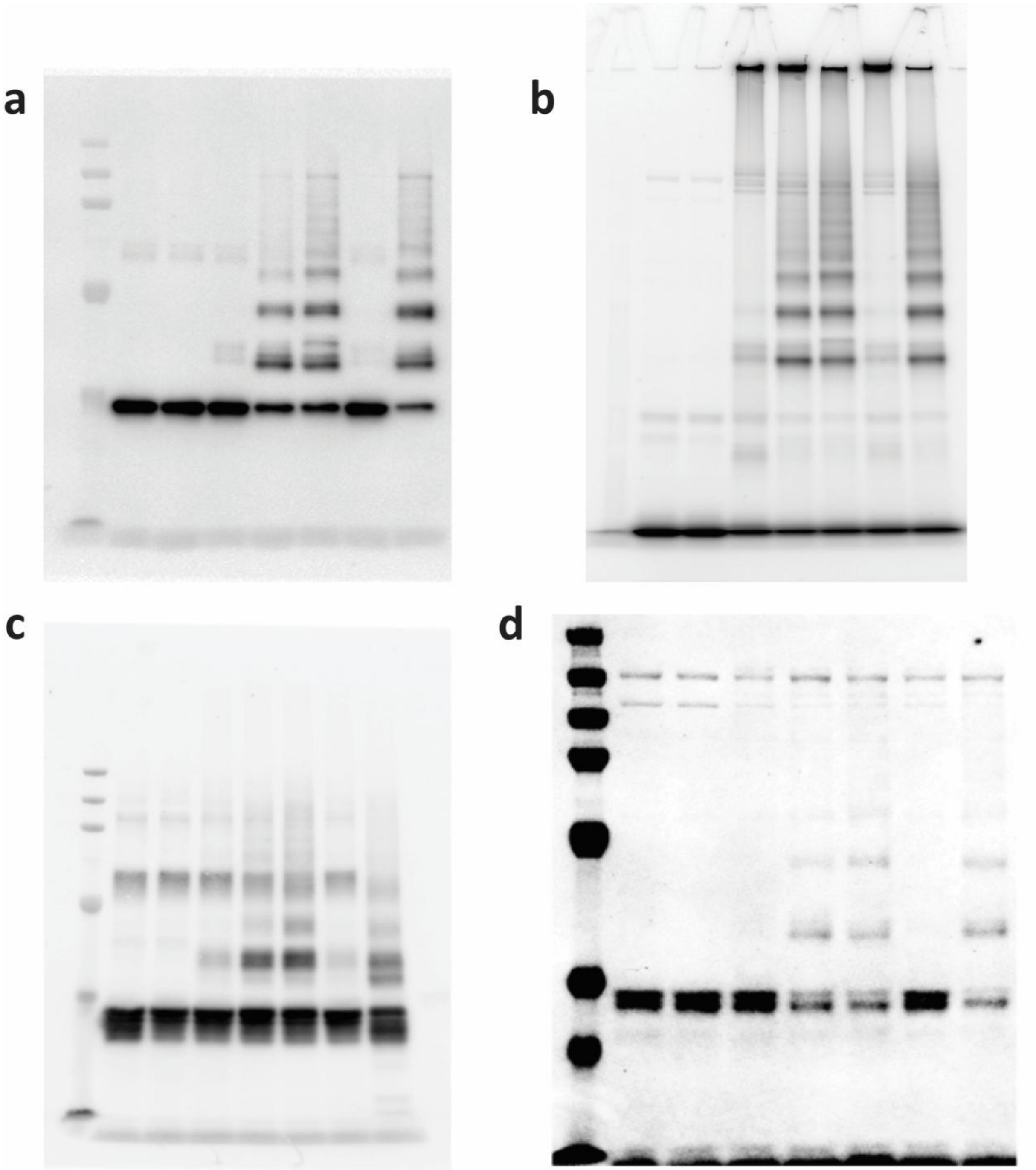

Figure S23. Unprocessed full Western blot depicting ladder or SDS gel (a) 14-3-3 antibody, (b) Gel image at wavelength 519 nm to visualize ubiquitin-labelled Alexa Fluor 488, (c) ERα antibody and (d) Ponceau stain of blot. Source data are provided in the Source Data file.

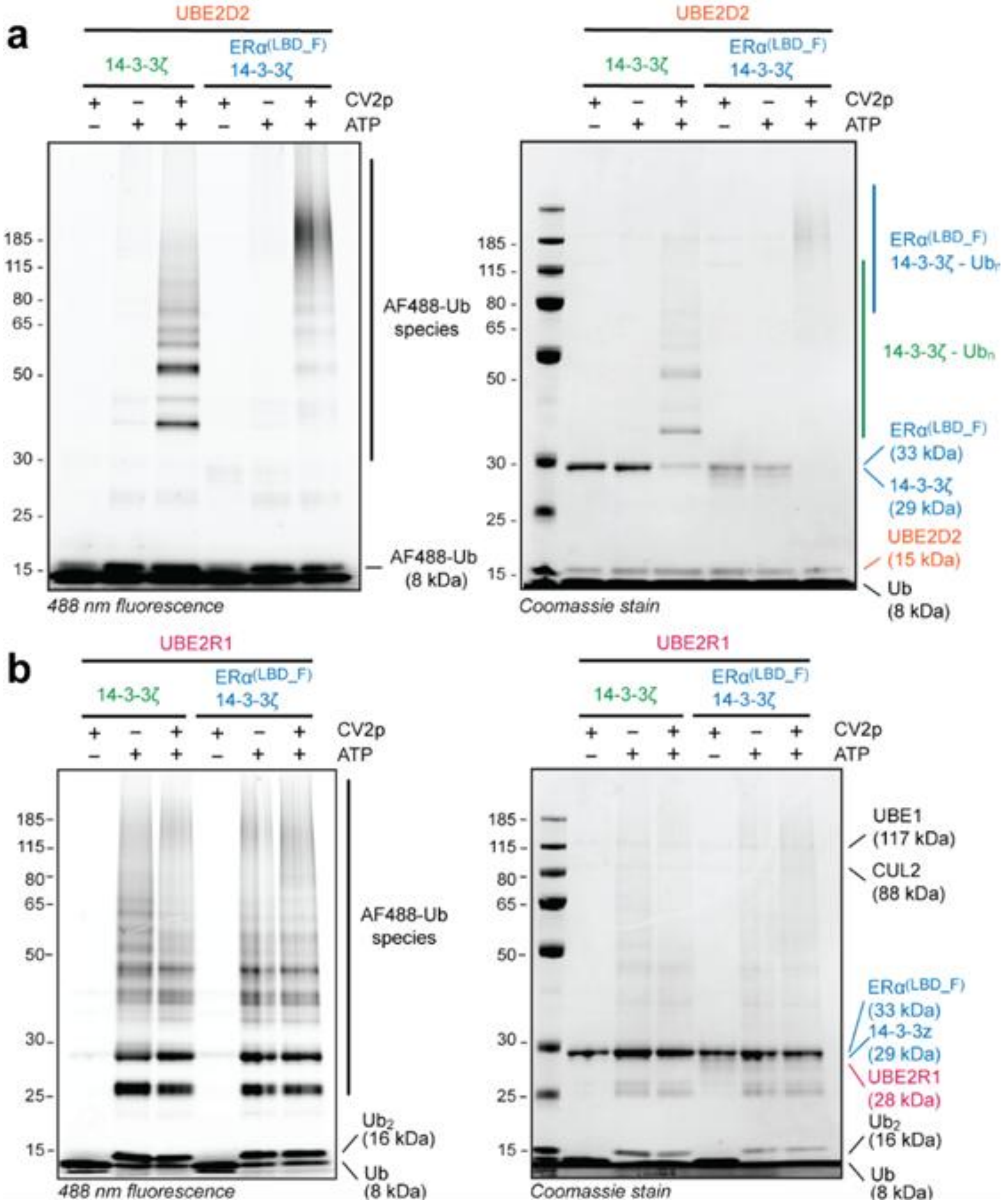

Figure S24. Ubiquitination assays with (a) UBE2D2 or (b) UBE2R1, and 14-3-3ζ or the 14-3-3ζ/ERα(LBD_F) complex, visualized at 488 nm (Alexa Fluor 488-labelled ubiquitin) or Coomassie stain. Source data are provided in the Source Data file.

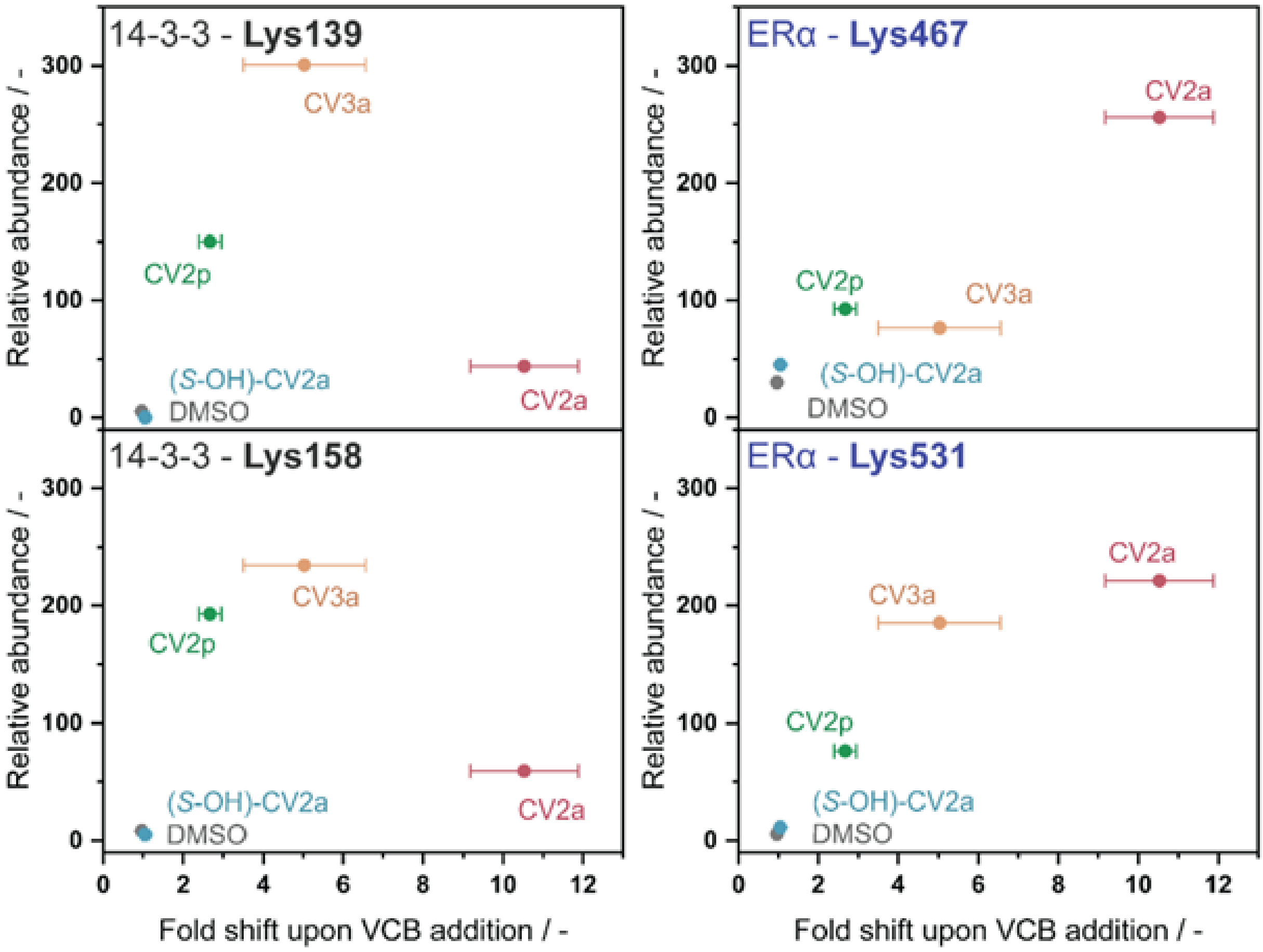

Figure S25. Comparison between ubiquitination in the in-vitro ubiquitination mass spectrometry experiment (vertical) and the cooperativity observed in the FA experiment (horizontal), expressed as fold 14-3-3 binding curve shift upon addition of VCB. Data are presented as mean values ± standard deviations (error bars) of independent scientific replicates (n = 3) for the horizontal axis and as single scientific replicate (n = 1) for the vertical axis. Source data are provided in the Source Data file.

2. Supplementary Tables

Table S1. Overview of tryptic peptides measured by mass spectrometry in the in-vitro ubiquitination experiment.

Protein	Annotated Sequence	Modifications	PSMs	Missed cleavages	Compound				
					**DMSO**	**CV2p**	**CV2a**	**(*S*-OH)-CV2a**	**CV3a**
ERα	[K].CKNVVPLYDLLLEMLDAHR.[L]	1x Carbamidomethyl [C1]; 1x GG [K2]	29	1	30	92.5	255.7	45.3	76.5
ERα	[K].SIILLNSGVYTFLSSTLK.[S]	1x GG [K18]	3	0					500
ERα	[K].SIILLNSGVYTFLSSTLKSLEEK.[D]	1x GG [K18]	56	1	5.7	76.2	221.3	11.3	185.5
ERα	[R].GGASVEETDQSHLATAGSTSSHSLQKYYITGEAEGR.[R]	1x GG [K26]	1	1					
ERα	[R].GGASVEETDQSHLATAGSTSSHSLQK.[Y]	1x Phospho [S/T]	5	0	143.1	110.4	115.8	130.7	
ERα	[R].GGASVEETDQSHLATAGSTSSHSLQK.[Y]	1x Phospho [S23]; 1x GG [K26]	4	0	58.2	168.7	77.3	195.8	
ERα	[K].GMEHLYSMK.[C]	2x Oxidation [M2; M8]	1	0	40.5	130.9	121	103.2	104.5
14-3-3ζ	[K].KGIVDQSQQAYQEAFEISKK.[E]	1x GG [K1]	4	2			500		
14-3-3ζ	[K].KEMQPTHPIR.[L]	1x GG [K1]	27	1	8.3	192.9	59.2	5.2	234.4
14-3-3ζ	[K].KGIVDQSQQAYQEAFEISK.[K]	1x GG [K1]	16	1	5.3	150.1	44		300.7
14-3-3ζ	[R].YLAEVAAGDDKK.[G]	1x GG [K11]	3	1		125.5	91.4		283.1
14-3-3ζ	[K].GIVDQSQQAYQEAFEISKK.[E]	1x GG [K18]	1	1					
14-3-3ζ	[K].TAFDEAIAELDTLSEESYKDSTLIMQLLR.[D]	1x GG [K19]	10	1	56.8		68.2		375
14-3-3ζ	[K].AKLAEQAER.[Y]	1x GG [K2]	1	1		371.6			128.4
14-3-3ζ	[-].MDKNELVQK.[A]	1x GG [K3]	5	1		343.5	78		78.5
14-3-3ζ	[R].DNLTLWTSDTQGDEAEAGEGGENWSHPQFEK.[-]	1x GG [K31]	15	0	18.6	171.9	141.2	18.4	149.9
14-3-3ζ	[K].DSTLIMQLLRDNLTLWTSDTQGDEAEAGEGGENWSHPQFEK.[-]	1x GG [K41]	16	1	0.3	134	349.1	4.4	12.3
14-3-3ζ	[K].NELVQKAK.[L]	1x GG [K6]	4	1					
14-3-3ζ	[R].VVSSIEQKTEGAEK.[K]	1x GG [K8]	1	1					
14-3-3ζ	[K].KEMQPTHPIR.[L]	1x Oxidation [M3]; 1x GG [K1]	10	1		166.7	18.2		315.1
14-3-3ζ	[K].DSTLIMQLLRDNLTLWTSDTQGDEAEAGEGGENWSHPQFEK.[-]	1x Oxidation [M6]; 1x GG [K41]	2	1			500		
14-3-3ζ	[K].SVTEQGAELSNEER.[N]	1x Phospho [S1]	5	0	80.9	133.9	85.7	90.7	108.7
14-3-3ζ	[K].FLIPNASQAESK.[V]	1x Phospho [S11]; 1x GG [K12]	2	0	117.5	16.2	71.7	278.8	15.7
14-3-3ζ	[K].TAFDEAIAELDTLSEESYK.[D]	1x Phospho [S17]	1	0					
14-3-3ζ	[K].GIVDQSQQAYQEAFEISKK.[E]	1x Phospho [S17]; 1x GG [K]	3	1	57.2	72.5	121.7	248.7	
14-3-3ζ	[K].TAFDEAIAELDTLSEESYK.[D]	1x Phospho [Y18]; 1x GG [K19]	8	0	100.6	114.6	111.2	161	12.5
14-3-3ζ	[R].YLAEVAAGDDKKGIVDQSQQAYQEAFEISK.[K]	2x GG [K11; K12]	1	2					

Table S2. Cryo-EM data collection parameters.

Data collection	*Dataset which yielded volumes: EMD-55233, EMD-55234, EMD-55235, EMD-55236, EMD-55237*
Microscope	Krios
Detector	Gatan K3 (counting mode)
Voltage (kV)	300
Magnification (nominal)	105,000x
Number of fractions	40
Total electron exposure ($e^{-}/Å^{2}$)	33
Defocus range (μm)	-1.2 to -2.7
Pixel size at detector (Å/pixel)	0.829
Total exposure (s)	1.75
Dose rate (e^{-}/px/s)	15.7
Automation software	EPU v2.1
Energy filter slit width (eV)	20
C2 aperture (μm)	70
Movies collected (#)	14,093

Table S3. Cryo-EM data image analysis.

Reconstruction	*Pose 1 EMDB-55234*	*Local refinement of 'Pose 1' EMDB-55233 PDB-9SV3*	*Pose 2.1 EMDB-55235*	*Pose 2.2 EMDB-55236*	*Pose 3 EMDB-55237*
Image processing package	CryoSPARC	CryoSPARC	CryoSPARC	CryoSPARC	CryoSPARC
Symmetry imposed	C1	C1	C1	C1	C1
Initial particles (#)	148,017	148,017	192,706	215,217	99,236
Final particles (#)	148,017	140,642	192,706	215,217	99,236
Map resolution (Å) at FSC 0.143	4.6	4.3	6.5	6.0	5.6
B Factor ($Å^{2}$)	280	382	489	475	436
Map resolution range (Å)	4.0 to 12.7	3.9 to 5.1	5.6 to 10.6	5.3 to 14.4	3.9 to 9.8
*SCF**	0.892	0.814	0.971	0.943	0.944
cFAR	0.07	0.44	0.20	0.07	0.12

Table S4. Cryo-EM data atomic modelling, refinement, and validation statistics.

Model composition	*Local refinement of 'Pose 1' EMDB-55233 PDB-9SV3*
Proteins	VHL, EloC, EloB, 14-3-3zeta, estrogen receptor alpha
Ligands	CV2a
Model refinement	
Atomic modelling packages	iSOLDE, Phenix
Initial model(s) used	4W9H, 8C43, 8BZH, AlphaFold
Cross-correlation (map/model)	0.35
R.m.s deviations from ideal values:	
- Bond lengths (Å)	0.014
- Bond angles (°)	1.854
Protein residues B Factor(Å)	12.68
Ligand B Factor (Å)	16.52
Validation	
MolProbity score	1.84
Clashscore (all atoms)	13.42
Poor rotamers (%)	0.83
Ramachandran - favoured (%)	96.77
Ramachandran outliers (%)	0.00
EMRinger score	0.39
CaBLAM outliers (%)	0.63

3. Supplementary Methods

Biology

N-terminally biotinylated 14-3-3 protein (gamma isoform, residues 1-247) was prepared by BirA enzyme co-expression using a pETDUet-1.

BirA sequence (based on Addgene plasmid #20857) :
ATGAAGGATAACACCGTGCCACTGAAATTATTGCGCTGCTGGCCAATGGCGGAGAATTTC
ACTCGGGAGAACAGCTGGGTGAAACTCTGGGCATGTCTCGTGCTGCCATCAATAAACACA
TTCAGACACTGCGTGACTGGGGCGTTGACGTGTTCACTGTGCCGGGCAAAGGCTATAGCC
TGCCGGAAATTCAAATTGCTGAACGCCAAGATTCTGGGCCAGCTGGACGGCGGCTCGGTG
GCCGTGCTGCCGGTGATTGACAGCACCAACTATCTGCTGGACCGTATCGGCGAGCTGAAA
AGCGGCGACGCGTGTATCGCCGAGTACCAGCAGGCGCGTGGGCGTCGCGGCGTAAATGG
TTCTCGCCGTTTGGTGCCAACCTGTACCTGTCCATGTTCTGGCGTCTGGAGCAGGGCCCGG
CCGCCGCGATTGGCTCGCTGTCGGTGATTGGGATTGTCATGGCCGAAGTGCTGCGTAAAC
TGGGCGCGGACAAGGTGCGTGTGAAATGGCCGAACGACCTGTACCTGCAGGACCGTAAG
CTGGCGGGCATTCTGGTGGAGCTGACGGGCAAGACCGGCGACGCGGCGCAGATTGTCAT
TGGCGCGGGCATCAATATGGCCATGCGTCGTGTGGAGGAGTCCGTGGTGAACCAGGGCT
GGATTACGCTGCAGGAGGCGGGGATCAATCTGGATCGCAACACGCTGGCCGCGATGCTG
ATTAGGGAGCTGCGTGCCGCGCTGGAGCTGTTTGAGCAGGAGGGCCTGGCGCCGTATCTG
TCCCGTTGGGAGAAGCTGGACAATTTTATCAACCGTGTTAAACTGATTATCGGCGATAAA
GAGATTTTCGGGATCAGCCGTGGGATTGACAAGCAGGGCGCGCTGCTGCTGGAGCAGGA
CGGCATTATTAAACCGTGGATGGGCGGCGAATCAGTCTGCGTAGCGCGGAAA

biotin-14-3-3s sequence:

ATGAGCTATTACCACCACCACCACCACCACGACTACGACATTCCGACCACCGAGAACCTG
TATTTCCAAGGCGCGATGGGCAGCGGCCTGAACGACATCTTCGAGGCGCAGAAGATTGA
GTGGCACGAAGGTAGCATGGAACGTGCGAGCCTGATCCAGAAGGCGAAACTGGCGGAGC
AAGCGGAACGTTACGAGGATATGGCGGCGTTTATGAAGGGTGCGGTGGAGAAAGGCGAG
GAACTGAGCTGCGAGGAACGTAACCTGCTGAGCGTGGCGTATAAAAACGTGGTTGGTGG
CCAACGTGCGGCGTGGCGTGTTCTGAGCAGCATCGAACAAAAGAGCAACGAGGAAGGTA
GCGAGGAAAAAGGCCCGGAAGTGCGTGAGTACCGTGAAAAGGTTGAAACCGAACTGCAG
GGTGTGTGCGACACCGTTCTGGGCCTGCTGGATAGCCACCTGATTAAAGAGGCGGGTGAC
GCGGAAAGCCGTGTGTTCTACCTGAAGATGAAGGGTGATTACTATCGTTATCTGGCGGAA
GTGGCGACCGGCGACGATAAGAAACGTATCATTGACAGCGCGCGTAGCGCGTACCAAGA
GGCGATGGATATCAGCAAGAAAGAAATGCCGCCAACCAACCCGATTCGTCTGGGCCTGG
CGCTGAACTTCAGCGTTTTTCACTATGAAATCGCGAACAGCCCGGAGGAAGCGATTAGCC
TGGCGAAGACCACCTTTGACGAGGCGATGGCGGATCTGCACACCCTGAGCGAAGACAGC
TATAAAGATAGCACCCTGATTATGCAACTGCTGCGTGACAACCTGACCCTGTGGACCGCG
GACAACGCGGGTGAAGAAGGTGGTGAAGCGCCGCAGGAACCGCAGAGCTAA

Chemistry

General chemistry information

All reagents were purchased from TCI, Sigma-Aldrich, or BLDPharm and were used without further purification. All solvents were of analytical grade and supplied by Biosolve. Reaction glassware was dried at 130 °C for more than 24 hours prior to use. TLC was carried out on aluminum-backed silica

(Merck silica gel 60 F_{254}) plates supplied by Merck. Visualization of the plates was achieved using an ultraviolet lamp (λ_{max} = 254 nm) or by phosphomolybdic acid staining. Reversed-phase preparative HPLC was performed using a C18 Atlantis T3 OBD 150 x 19 mm column using miliQ water with 0.1% formic acid (FA) and acetonitrile (ACN) with 0.1% FA. Analytical (LR) LC-MS (ESI) analysis was performed on a system using comprising a C18 Jupiter SuC4300A 150 x 2.0 mm column using water with 0.1% FA and acetonitrile with 0.1% FA, using a gradient of 5% to 100% acetonitrile over 8 minutes, connected to a Thermo Fisher LTQ XL Linear Ion Trap Mass Spectrometer. High resolution mass spectra (HRMS) were recorded using a Waters ACQUITY UPLC I-Class LC system coupled to a Xevo G2 Quadrupole Time of Flight (Q-TOF) mass spectrometer equipped with a Phenomex kinetex® 2.6 µm EVO C18 100 x 2.1 mm column. Proton (^{1}H) and carbon (^{13}C) NMR spectra were collected on a 400 MHz Bruker Cryomagnet. Chemical shifts (δ) are referenced to the residual solvent peak. Splitting patterns are reported in an abbreviated manner: s (singlet), d (doublet), t (triplet), q (quartet), and m (multiplet). The percentages of 2-methylbut-3-en-2-yl ether cleavage of the final PROTAC compounds are estimated by HPLC and ^{1}H NMR.

Synthesis overview

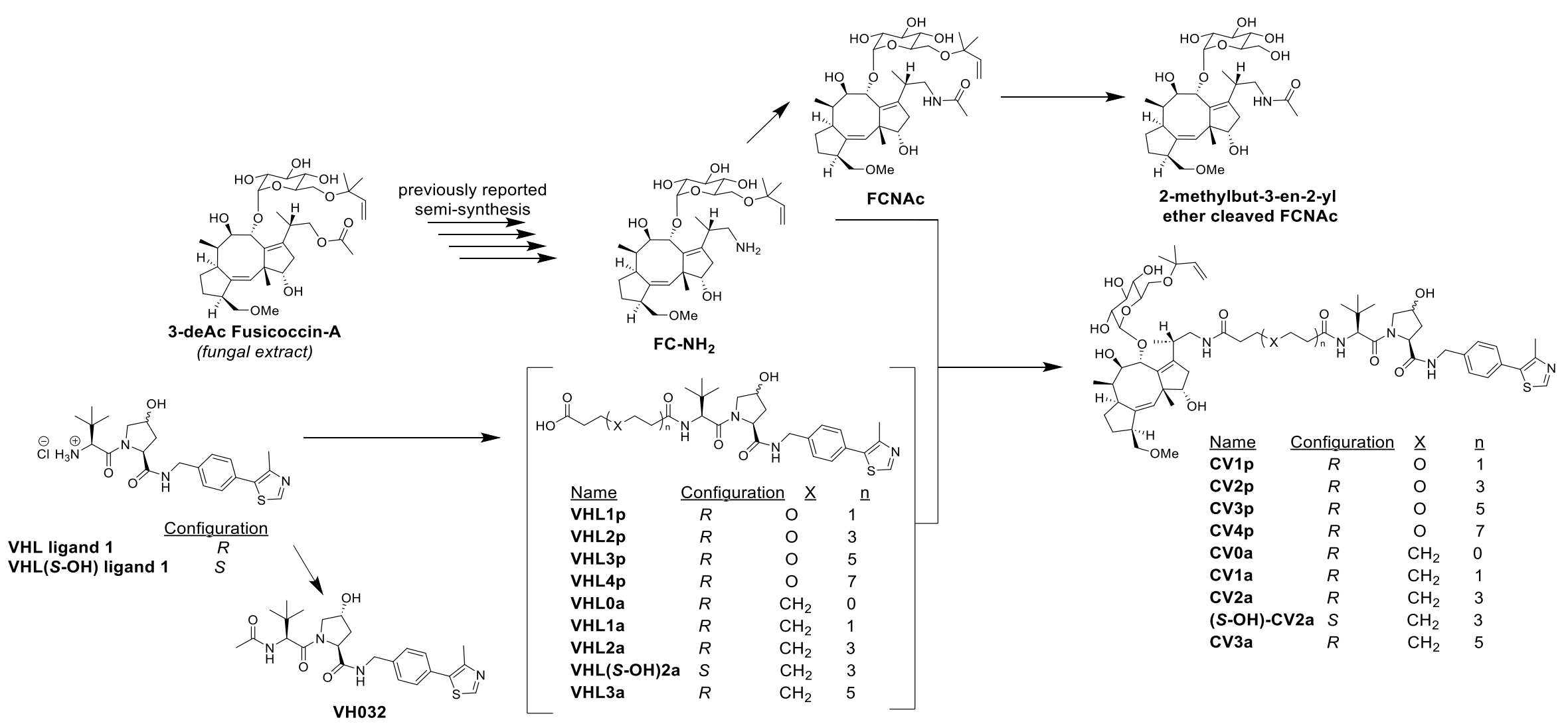

Scheme 1. Synthesis route to access the focused library of ^{MG}PROTAC.

General procedure A: acetylation

To a solution of amine or suspension of amine HCl salt (0.0588-0.214 mmol, 1 eq) in dry chloroform (3-5 mL) at 0° C was added DIPEA (0.294-1.07 mmol, 5 eq). The resulting solution was stirred for 10 min and acetic anhydride (0.0588-0.214 mmol, 1 eq) was slowly added over 5 min. The reaction mixture was allowed to warm to room temperature, stirred for 16h and quenched by the addition of water (5 mL per mmol). The resulting mixture was concentrated under reduced pressure and purified by reversed-phase preparative HPLC to yield the desired product.

General procedure B: coupling VHL to diacid

To a solution of diacid (0.642-1.07 mmol, 3-5 eq) and HATU (0.600-0.964 mmol, 2.8-4.5 eq) in dry DMF (2-17 mL) was added DIPEA (6.42 mmol, 30 eq). The resulting solution was stirred for 10 min and VHL ligand 1 HCl salt (0.214 mmol, 1 eq) dissolved in dry DMF (3 mL) was slowly added over 5 min. The reaction mixture was then stirred for 16h and quenched by the addition of water (5 mL per mmol). The resulting mixture was concentrated under reduced pressure and purified by reversed-phase preparative HPLC to yield the desired product.

General procedure C: coupling FC-NH_2 to linker-VHL

To a solution of linker-VHL (0.0646 mmol, 1.1 eq) and HATU (0.0764 mmol, 1.3 eq) in dry DMF (4 mL) was added DIPEA (0.587 mmol, 10 eq). The resulting solution was stirred for 10 min and FC-NH_2 (0.0588 mmol, 1 eq) dissolved in dry DMF (1 mL) was added. The reaction mixture was then stirred for 16h and quenched by the addition of water (5 mL per mmol). The resulting mixture was concentrated under reduced pressure and purified by reversed-phase preparative HPLC to yield the desired product.

Detailed synthetic procedures and characterization

FC-NH_2 - ((2*S*,3*R*,4*S*,5*S*,6*R*)-2-(((1*S*,4*R*,5*R*,6*R*,6a*S*,9*S*,10a*R*,*E*)-3-((*S*)-1-aminopropan-2-yl)-1,5-dihydroxy-9-(methoxymethyl)-6,10a-dimethyl-1,2,4,5,6,6a,7,8,9,10a-decahydrodicyclopenta[*a*,*d*][8]annulen-4-yl)oxy)-6-(((2-methylbut-3-en-2-yl)oxy)methyl)tetrahydro-2*H*-pyran-3,4,5-triol)

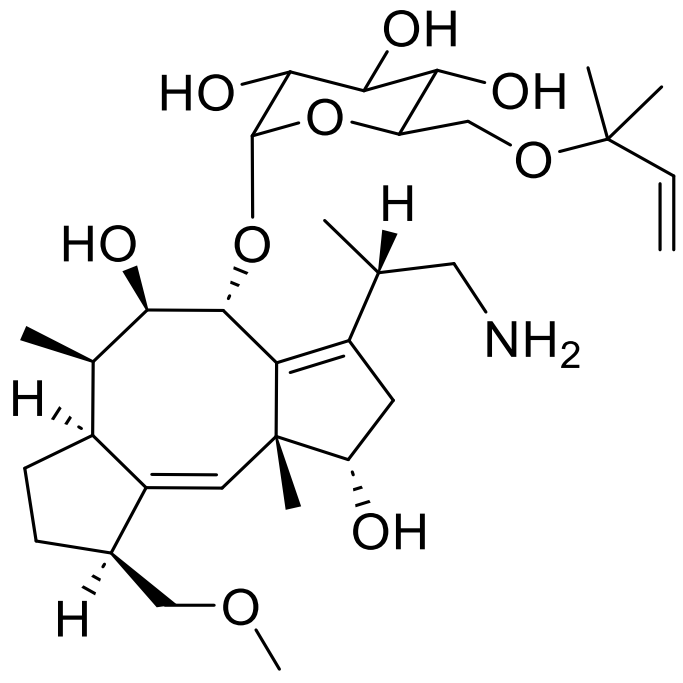

FC-NH_2 was semi-synthesized from **FC-A** (obtained as a metabolite of *Phomopsis amygdali*) in accordance with the previous report, and isolated as white solid (469 mg, 0.735 mmol, 38% yield over deacetylation, tosylation, azide installation, and azide reduction) that matched the reported spectral data.[3] **LC-MS (ESI)**: t_R = 3.03 min, *m/z* = 637.41 calcd. for $[C_{32}H_{53}NO_9$ +acetonitrile $H]^+$, found 637.75. **HRMS (ESI-TOF)**: *m/z* = 596.3799 calcd. for $[C_{32}H_{53}NO_9 + H]^+$, found 596.3805 [δ 1.0 ppm].

FC-NAc - (*N*-((*S*)-2-((1*S*,4*R*,5*R*,6*R*,6a*S*,9*S*,10a*R*,*E*)-1,5-dihydroxy-9-(methoxymethyl)-6,10a-dimethyl-4-(((2*S*,3R,4*S*,5*S*,6*R*)-3,4,5-trihydroxy-6-(((2-methylbut-3-en-2-yl)oxy)methyl)tetrahydro-2*H*-pyran-2-yl)oxy)-1,2,4,5,6,6a,7,8,9,10a-decahydrodicyclopenta[*a*,*d*][8]annulen-3-yl)propyl)acetamide)

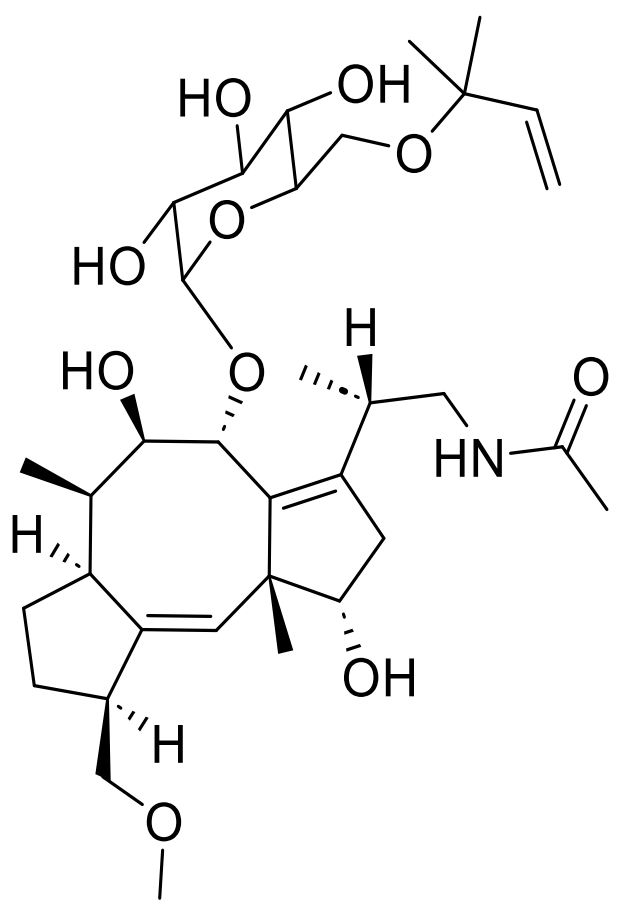

FC-NAc was synthesized via general procedure **A** using **FC-NH_2** (35.0 mg, 58.8 µmol, 1 eq), DIPEA (51.2 µL, 0.294 mmol, 5 eq) and acetic anhydride (5.55 µL, 58.8 µmol, 1 eq) in dry chloroform (3 mL).

The crude product was purified by reversed-phase preparative HPLC (30-35% acetonitrile (+0.1% FA) in water (+0.1% FA) gradient) to afford **FC-NAc** as a white solid (28.6 mg, 44.9 µmol, 76.4% yield) that matched the reported spectral data.[3] **LC-MS (ESI)**: t_R = 3.63 min, *m/z* = 638.39 calcd. for $[C_{34}H_{55}NO_{10} + H]^+$, found 638.58. **HRMS (ESI-TOF)**: *m/z* = 638.3904 calcd. for $[C_{34}H_{55}NO_{10} + H]^+$, found 638.3909 [$\delta$ 0.8 ppm]. **^{1}H NMR** (399 MHz, DMSO) δ 7.73 – 7.65 (m, 1H), 5.83 – 5.68 (m, 2H), 5.30 (s, 1H), 5.09 (dd, *J* = 10.9, 1.1 Hz, 2H), 4.86 – 4.70 (m, 5H), 3.76 – 3.62 (m, 3H), 3.61 – 3.54 (m, 1H), 3.49 – 3.40 (m, 2H), 3.28 – 3.13 (m, 8H), 3.07 – 2.91 (m, 3H), 2.69 – 2.60 (m, 2H), 2.13 – 2.04 (m, 1H), 1.92 – 1.77 (m, 6H), 1.51 – 1.37 (m, 3H), 1.17 (s, 6H), 1.13 (s, 3H), 0.92 (d, *J* = 5.9 Hz, 3H), 0.72 (d, *J* = 7.1 Hz, 3H). **^{13}C NMR** (100 MHz, DMSO) δ 171.3, 169.6, 144.1, 140.8, 139.4, 137.1, 127.0, 113.7, 102.3, 80.9, 77.9, 77.6, 75.3, 74.6, 73.1, 72.2, 70.6, 62.5, 57.9, 52.3, 48.2, 42.9, 41.0, 35.4, 35.2, 33.5, 27.1, 25.9, 25.7, 23.3, 22.6, 16.1, 9.2.

2-methylbut-3-en-2-yl ether cleaved FC-NAc - N-((S)-2-((1S,4R,5R,6R,6aS,9S,10aR,E)-1,5-dihydroxy-9-(methoxymethyl)-6,10a-dimethyl-4-(((2S,3R,4S,5S,6R)-3,4,5-trihydroxy-6-(hydroxymethyl)tetrahydro-2H-pyran-2-yl)oxy)-1,2,4,5,6,6a,7,8,9,10a-decahydrodicyclopenta[a,d][8]annulen-3-yl)propyl)acetamide

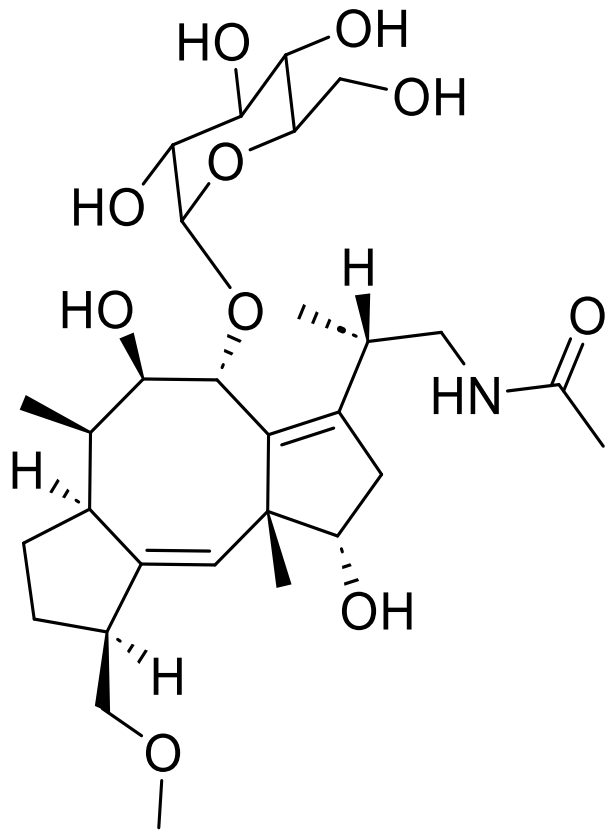

To a solution of **FC-NAc** (9.85 mg , 15.4 µmol, 1 eq) in dry DCM (1 mL) at 0° C was added TFA (59.1 µL, 772 µmol, 50 eq). The reaction mixture was stirred for 20 min at 0° C and quenched with 0.5 mL saturated $NaHCO_3$ solution. The resulting mixture was concentrated under reduced pressure and the crude material was purified by reversed-phase preparative HPLC (20-25% acetonitrile (+0.1% FA) in water (+0.1% FA) gradient) to afford **2-methylbut-3-en-2-yl ether cleaved FC-NAc** as a white solid (5.56 mg, 9.76 µmol, 63.2% yield). **LC-MS (ESI)**: t_R = 3.01 min, *m/z* = 570.33 calcd. for $[C_{29}H_{47}NO_{10} + H]^+$, found 570.33. **HRMS (ESI-TOF)**: *m/z* = 570.3278 calcd. for $[C_{29}H_{47}NO_{10} + H]^+$, found 570.3267 [$\delta$ -1.9 ppm]. **^{1}H NMR** (399 MHz, MeOD) δ 5.37 (s, 1H), 4.93 (d, *J* = 3.9 Hz, 1H), 4.55 (s, 1H), 3.96 – 3.84 (m, 2H), 3.78 – 3.57 (m, 5H), 3.42 (dd, *J* = 9.7, 3.8 Hz, 1H), 3.34 – 3.29 (m, 6H), 3.26 – 3.14 (m, 3H), 2.79 – 2.70 (m, 2H), 2.25 (dd, *J* = 14.5, 6.8 Hz, 1H), 2.07 – 1.88 (m, 6H), 1.61 – 1.46 (m, 3H), 1.20 (s, 3H), 0.99 (d, *J* = 1.9 Hz, 3H), 0.81 (d, *J* = 7.2 Hz, 3H) ppm. **^{13}C NMR** (100 MHz, MeOD) δ 173.4, 143.0, 142.8, 139.1, 127.9, 103.7, 82.7, 79.7, 79.1, 77.1, 75.0, 74.3, 73.8, 71.5, 62.2, 58.8, 54.3, 49.8, 45.0, 43.0, 42.7, 36.7, 36.6, 35.0, 28.4, 24.3, 22.7, 16.2, 10.0 ppm. Experimental procedure adapted from Ohkanda *et al.*[4]

VH032 - ((2*R*,4*S*)-1-((*R*)-2-acetamido-3,3-dimethylbutanoyl)-4-hydroxy-*N*-(4-(4-methylthiazol-5-yl)benzyl)pyrrolidine-2-carboxamide)

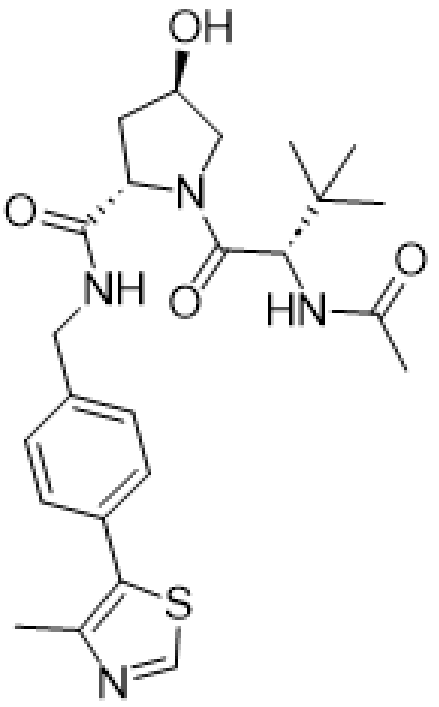

VH032 was synthesized via general procedure **A** using VHL ligand 1 HCl salt (100 mg, 0.214 mmol, 1 eq), DIPEA (186 µL, 1.07 mmol, 5 eq) and acetic anhydride (20.2 µL, 0.214 mmol, 1 eq) in dry chloroform (5 mL). The crude product was purified by reversed-phase preparative HPLC (30-40% acetonitrile (+0.1% FA) in water (+0.1% FA) gradient) to afford **VH032** as a white solid (94.5 mg, 0.200 mmol, 93.4% yield) that matched the reported spectral data.[5] **LC-MS (ESI)**: t_R = 3.42 min, *m/z* = 473.22 calcd. for $[C_{24}H_{32}N_4O_4S + H]^+$, found 473.17. **HRMS (ESI-TOF)**: *m/z* = 473.2223 calcd. for $[C_{24}H_{32}N_4O_4S + H]^+$, found 473.2219 [$\delta$ -0.8 ppm]. **^{1}H NMR** (399 MHz, $CDCl_3$) δ 8.68 (s, 1H), 7.41 – 7.27 (m, 5H), 6.16 (d, *J* = 8.7 Hz, 1H), 4.70 (t, *J* = 7.9 Hz, 1H), 4.59 – 4.47 (m, 3H), 4.33 (dd, *J* = 14.9, 5.2 Hz, 1H), 4.07 (d, *J* = 11.3 Hz, 1H), 3.61 (dd, *J* = 11.3, 3.7 Hz, 1H), 3.32 (s, 1H), 2.59 – 2.49 (m, 4H), 2.16 – 2.08 (m, 1H), 1.98 (s, 3H), 0.93 (s, 9H). **^{13}C NMR** (100 MHz, $CDCl_3$) δ 172.0, 170.9, 170.8, 150.5, 148.6, 138.2, 131.7, 131.2, 129.7, 128.3, 70.2, 58.6, 57.8, 56.9, 43.4, 36.0, 35.0, 26.5, 23.2, 16.2.

CV1p ((2*S*,4*R*)-1-((*S*)-2-(3-(3-(((*S*)-2-((1*S*,4*R*,5*R*,6*R*,6a*S*,9*S*,10a*R*,*E*)-1,5-dihydroxy-9-(methoxymethyl)-6,10a-dimethyl-4-(((2*S*,3*R*,4*S*,5*S*,6*R*)-3,4,5-trihydroxy-6-(((2-methylbut-3-en-2-yl)oxy)methyl)tetrahydro-2*H*-pyran-2-yl)oxy)-1,2,4,5,6,6a,7,8,9,10a-decahydrodicyclopenta[*a*,*d*][8]annulen-3-yl)propyl)amino)-3-oxopropoxy)propanamido)-3,3-dimethylbutanoyl)-4-hydroxy-*N*-(4-(4-methylthiazol-5-yl)benzyl)pyrrolidine-2-carboxamide)

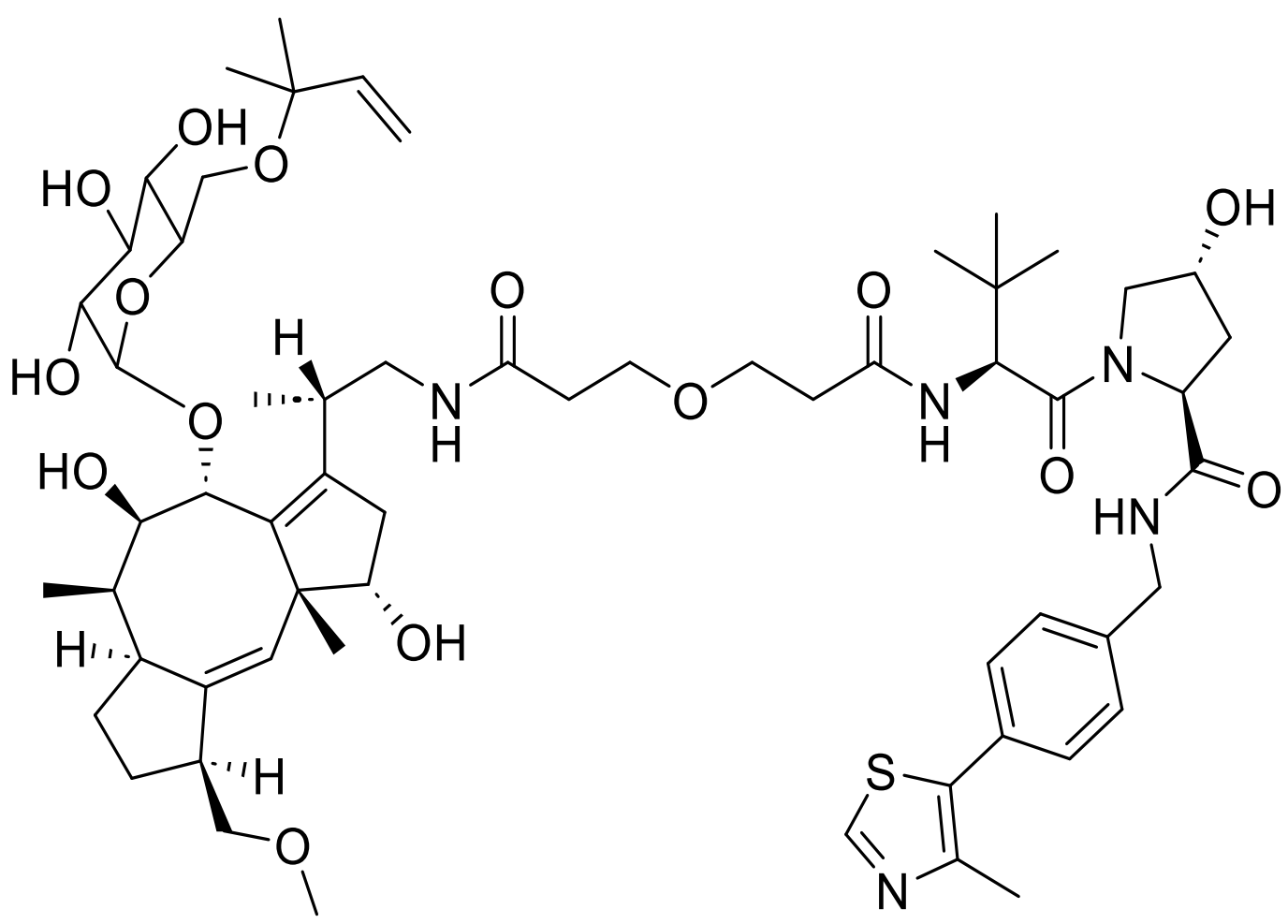

The intermediate **VHL1p** was synthesized via general procedure **B** using 3,3'-oxydipropionic acid (104 mg, 0.642 mmol, 3 eq), HATU (228 mg, 0.600 mmol, 2.8 eq) DIPEA (1.12 mL, 6.42 mmol, 30 eq) and VHL ligand 1 HCl salt (100 mg, 0.214 mmol, 1 eq) in dry DMF (5 mL). The crude product was purified by reversed-phase preparative HPLC (25-30% acetonitrile (+0.1% FA) in water (+0.1% FA) gradient) to afford intermediate **VHL1p** as a white solid (42.0 mg, 0.073 mmol, 34.2% yield). **LC-MS (ESI)**: t_R = 3.41 min, *m/z* = 575.25 calcd. for $[C_{28}H_{38}N_4O_7S + H]^+$, found 575.92. **HRMS (ESI-TOF)**: *m/z* = 575.2540 calcd. for $[C_{28}H_{38}N_4O_7S + H]^+$, found 575.2531 [$\delta$ -1.6 ppm].

CV1p was synthesized via general procedure **C** using **VHL1p** (37.1 mg, 0.0646 mmol, 1.1 eq), HATU (29.0 mg, 0.0764 mmol, 1.3 eq), DIPEA (102 µL, 0.587 mmol, 10 eq) and **FC-NH₂** (35 mg, 0.0588 mmol, 1 eq) in dry DMF (5 mL). The crude product was purified by reversed-phase preparative HPLC (40-45% acetonitrile (+0.1% FA) in water (+0.1% FA) gradient) to afford **CV1p** as a white solid (6.08 mg, 5.28 µmol, 9.0% yield), 13% 2-methylbut-3-en-2-yl ether cleaved. **LC-MS (ESI)**: t_R = 3.72 min, *m/z* = 1084.55 calcd. for [$C_{55}H_{81}N_5O_{15}S$ + H]$^+$, found 1084.42; t_R = 4.17 min, *m/z* = 1152.62 calcd. for [$C_{60}H_{89}N_5O_{15}S$ + H]$^+$, found 1152.58. **HRMS (ESI-TOF)**: *m/z* = 542.7803 calcd. for [$C_{55}H_{81}N_5O_{15}S$ + 2H]$^{2+}$, found 542.7787 [δ -2.9 ppm]; *m/z* = 576.8116 calcd. for [$C_{60}H_{89}N_5O_{15}S$ + 2H]$^{2+}$, found 576.8120 [δ 0.7 ppm]. **^{1}H NMR** (399 MHz, DMSO) δ 9.03 (s, 1H), 8.56 (t, *J* = 6.1 Hz, 1H), 7.93 (d, *J* = 9.3 Hz, 1H), 7.77 – 7.64 (m, 1H), 7.43 – 7.37 (m, 4H), 5.77 (dd, *J* = 17.7, 10.8 Hz, 1H), 5.32 – 5.27 (m, 1H), 5.11 (dd, *J* = 17.7, 1.5 Hz, 1H), 5.07 (dd, *J* = 10.8, 1.5 Hz, 1H), 4.74 – 4.73 (m, 1H), 4.55 (d, *J* = 9.4 Hz, 1H), 4.44 – 4.33 (m, 3H), 4.22 (dd, *J* = 15.9, 5.3 Hz, 1H), 3.68 – 3.42 (m, 12H), 3.25 – 2.97 (m, 11H), 2.68 – 2.59 (m, 2H), 2.45 (s, 3H), 2.41 – 2.31 (m, 3H), 2.11 – 2.01 (m, 2H), 1.93 – 1.80 (m, 4H), 1.47 – 1.37 (m, 3H), 1.29 – 0.97 (m, 10H), 0.94 – 0.88 (m, 12H), 0.72 (d, *J* = 7.0 Hz, 3H) ppm. **^{13}C NMR** (100 MHz, DMSO) δ 171.9, 170.4, 169.9, 169.5, 151.6, 147.4, 144.1, 140.8, 139.6, 139.4, 137.0, 131.3, 129.5, 128.6, 127.4, 127.1, 113.6, 102.2, 80.8, 77.8, 77.6, 75.3, 74.6, 73.0, 72.1, 72.1, 70.5, 68.9, 66.6, 62.5, 58.7, 57.9, 56.4, 56.3, 52.3, 48.2, 42.7, 41.6, 41.0, 40.9, 37.9, 36.0, 35.5, 35.4, 35.3, 35.2, 33.5, 27.1, 26.3, 25.8, 25.7, 23.3, 15.8, 15.8, 9.2 ppm.

CV2p - ((2S,4R)-1-((2S,19S)-2-(tert-butyl)-19-((1S,4R,5R,6R,6aS,9S,10aR,E)-1,5-dihydroxy-9-(methoxymethyl)-6,10a-dimethyl-4-(((2S,3R,4S,5S,6R)-3,4,5-trihydroxy-6-(((2-methylbut-3-en-2-yl)oxy)methyl)tetrahydro-2H-pyran-2-yl)oxy)-1,2,4,5,6,6a,7,8,9,10a-decahydrodicyclopenta[a,d][8]annulen-3-yl)-4,16-dioxo-7,10,13-trioxa-3,17-diazaicosanoyl)-4-hydroxy-N-(4-(4-methylthiazol-5-yl)benzyl)pyrrolidine-2-carboxamide)

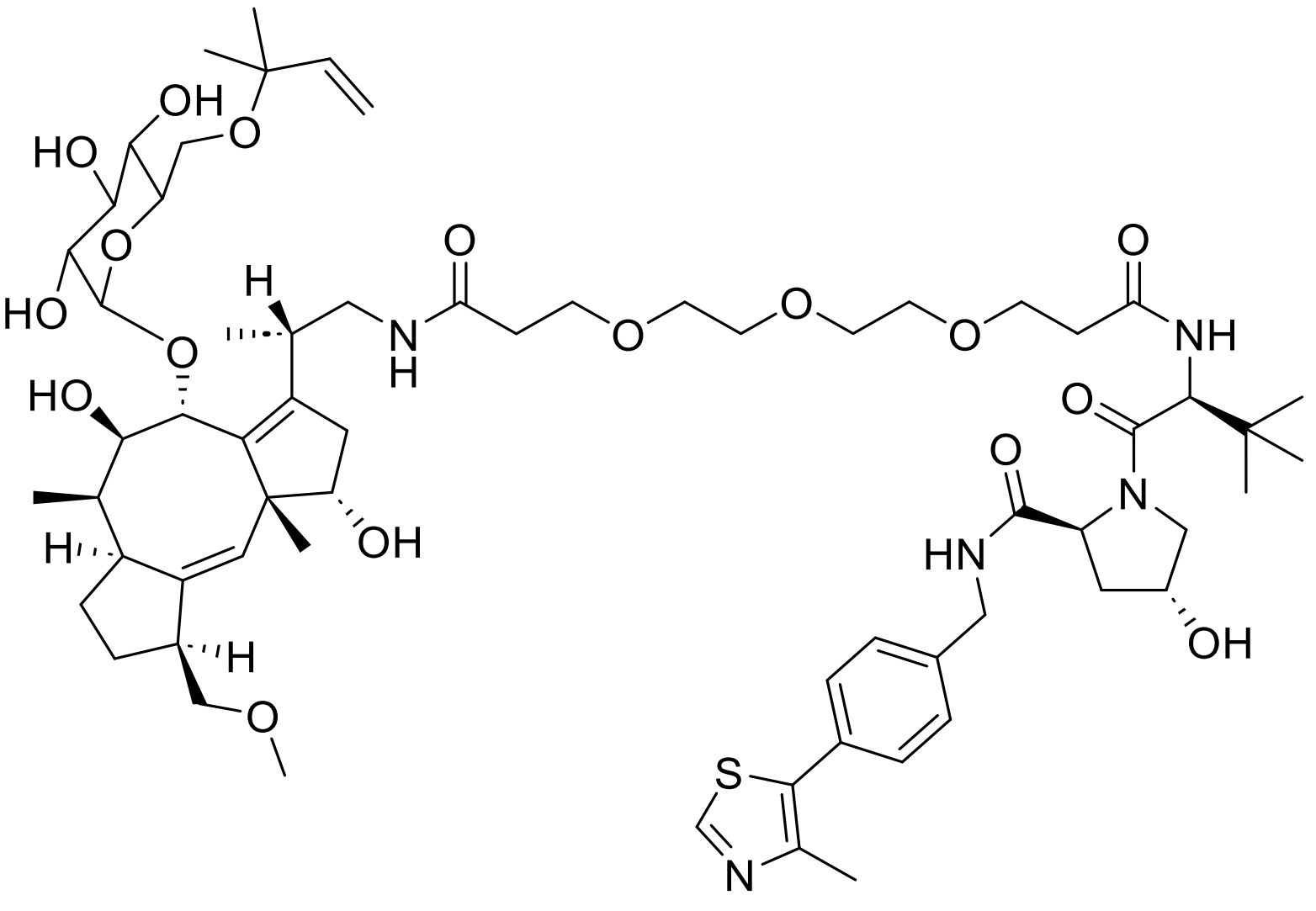

The intermediate **VHL2p** was synthesized via general procedure **B** using 3,3'-((oxybis(ethane-2,1-diyl))bis(oxy))dipropionic acid (161 mg, 0.642 mmol, 3 eq), HATU (228 mg, 0.600 mmol, 2.8 eq) DIPEA (1.12 mL, 6.42 mmol, 30 eq) and VHL ligand 1 HCl salt (100 mg, 0.214 mmol, 1 eq) in dry DMF (5 mL). The crude product was purified by reversed-phase preparative HPLC (35-40% acetonitrile (+0.1% FA) in water (+0.1% FA) gradient) to afford intermediate **VHL2p** as a white solid (86.6 mg, 0.131 mmol, 61.0% yield). **LC-MS (ESI)**: t_R = 3.50 min, *m/z* = 663.31 calcd. for [$C_{32}H_{46}N_4O_9S$ + H]$^+$, found 663.25. **HRMS (ESI-TOF)**: *m/z* = 663.3064 calcd. for [$C_{32}H_{46}N_4O_9S$ + H]$^+$, found 663.3063 [δ -0.2 ppm].

CV2p was synthesized via general procedure **C** using **VHL2p** (42.8 mg, 0.0646 mmol, 1.1 eq), HATU (29.0 mg, 0.0764 mmol, 1.3 eq), DIPEA (102 µL, 0.587 mmol, 10 eq) and **FC-NH₂** (35 mg, 0.0588 mmol, 1 eq) in dry DMF (5 mL). The crude product was purified by reversed-phase preparative HPLC (45-50% acetonitrile (+0.1% FA) in water (+0.1% FA) gradient) to afford **CV2p** as a white solid (11.9 mg, 9.58 µmol, 16.3% yield), 5% 2-methylbut-3-en-2-yl ether cleaved. **LC-MS (ESI)**: t_R = 3.72 min, *m/z* = 1172.61 calcd. for $[C_{59}H_{89}N_5O_{17}S + H]^+$, found 1172.33; t_R = 4.15 min, *m/z* = 1240.67 calcd. for $[C_{64}H_{97}N_5O_{17}S + H]^+$, found 1240.50. **HRMS (ESI-TOF)**: *m/z* = 586.8065 calcd. for $[C_{59}H_{89}N_5O_{17}S + 2H]^{2+}$, found 586.8062 [$\delta$ -0.5 ppm]; *m/z* = 620.8378 calcd. for $[C_{64}H_{97}N_5O_{17}S + 2H]^{2+}$, found 620.8384 [$\delta$ 1.0 ppm]. **^{1}H NMR** (399 MHz, CD_3CN) δ 9.25 (s, 1H), 7.50 – 7.40 (m, 5H), 7.03 (d, *J* = 8.8 Hz, 1H), 6.99 – 6.92 (m, 1H), 5.81 (dd, *J* = 17.7, 10.8 Hz, 1H), 5.32 – 5.29 (m, 1H), 5.14 (dd, *J* = 17.7, 1.4 Hz, 1H), 5.08 (dd, *J* = 10.8, 1.4 Hz, 1H), 4.87 (d, *J* = 3.8 Hz, 1H), 4.58 – 4.42 (m, 4H), 4.30 (dd, *J* = 15.8, 5.6 Hz, 1H), 3.81 – 3.54 (m, 21H), 3.38 – 3.24 (m, 9H), 3.10 – 3.05 (m, 2H), 2.81 – 2.67 (m, 4H), 2.50 – 2.39 (m, 4H), 2.20 – 2.02 (m, 3H), 1.90 – 1.85 (m, 2H), 1.52 – 1.46 (m, 3H), 1.23 – 1.17 (m, 10H), 1.02 – 0.93 (m, 12H), 0.78 (d, *J* = 7.1 Hz, 3H) ppm. **^{13}C NMR** (100 MHz, CD_3CN) δ 173.0, 172.7, 172.2, 171.8, 154.3, 145.2, 144.9, 143.1, 141.9, 141.9, 138.7, 135.3, 130.1, 128.9, 127.3, 114.2, 103.2, 82.0, 79.4, 79.1, 76.5, 76.0, 74.4, 73.7, 73.4, 72.1, 70.9, 70.8, 70.6, 67.9, 67.7, 63.6, 60.2, 58.6, 58.2, 57.7, 54.2, 49.5, 44.8, 43.1, 42.4, 42.2, 38.4, 37.1, 37.0, 36.7, 36.4, 36.2, 34.8, 28.3, 26.7, 26.3, 24.3, 16.6, 14.6, 10.0 ppm.

CV3p (*N*^{1}-((*S*)-2-((1*S*,4*R*,5*R*,6*R*,6a*S*,9*S*,10a*R*,*E*)-1,5-dihydroxy-9-(methoxymethyl)-6,10a-dimethyl-4-(((2*S*,3*R*,4*S*,5*S*,6*R*)-3,4,5-trihydroxy-6-(((2-methylbut-3-en-2-yl)oxy)methyl)tetrahydro-2*H*-pyran-2-yl)oxy)-1,2,4,5,6,6a,7,8,9,10a-decahydrodicyclopenta[*a*,*d*][8]annulen-3-yl)propyl)-*N*^{19}-((*S*)-1-((2*S*,4*R*)-4-hydroxy-2-((4-(4-methylthiazol-5-yl)benzyl)carbamoyl)pyrrolidin-1-yl)-3,3-dimethyl-1-oxobutan-2-yl)-4,7,10,13,16-pentaoxanonadecanediamide)

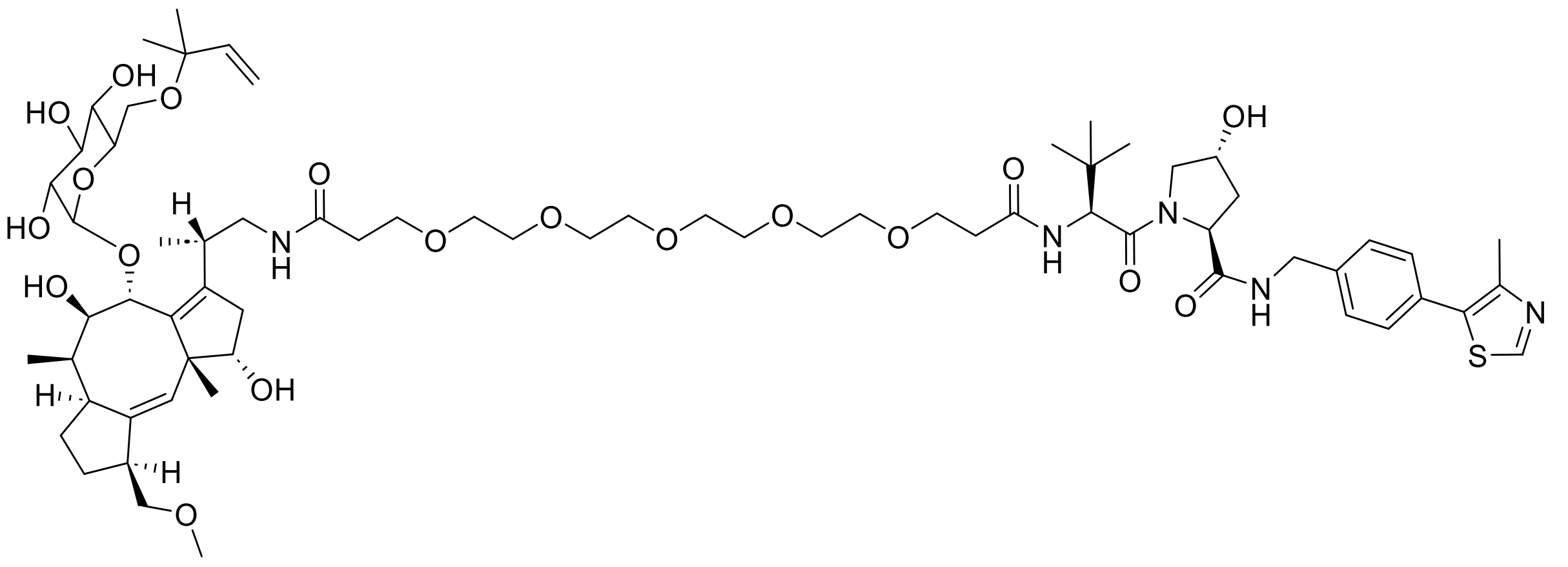

The intermediate **VHL3p** was synthesized via general procedure **B** using 4,7,10,13,16-pentaoxanonadecanedioic acid (217 mg, 0.642 mmol, 3 eq), HATU (228 mg, 0.600 mmol, 2.8 eq) DIPEA (1.12 mL, 6.42 mmol, 30 eq),and VHL ligand 1 HCl salt (100 mg, 0.214 mmol, 1 eq) in dry DMF (5 mL). The crude product was purified by reversed-phase preparative HPLC (35-40% acetonitrile (+0.1% FA) in water (+0.1% FA) gradient) to afford intermediate **VHL3p** as a white solid (90.5 mg, 0.121 mmol, 56.3% yield). **LC-MS (ESI)**: t_R = 3.55 min, *m/z* = 751.36 calcd. for $[C_{36}H_{54}N_4O_{11}S + H]^+$, found 751.42. **HRMS (ESI-TOF)**: *m/z* = 751.3588 calcd. for $[C_{36}H_{54}N_4O_{11}S + H]^+$, found 751.3586 [$\delta$ -0.3 ppm].

CV3p was synthesized via general procedure **C** using **VHL3p** (48.5 mg, 0.0646 mmol, 1.1 eq), HATU (29.0 mg, 0.0764 mmol, 1.3 eq), DIPEA (102 μL, 0.587 mmol, 10 eq) and **FC-NH₂** (35 mg, 0.0588 mmol, 1 eq) in dry DMF (5 mL). The crude product was purified by reversed-phase preparative HPLC (45-50% acetonitrile (+0.1% FA) in water (+0.1% FA) gradient) to afford **CV3p** as a white solid (22.2 mg, 16.7 μmol, 28.4% yield), 6% 2-methylbut-3-en-2-yl ether cleaved. **LC-MS (ESI)**: t_R = 3.74 min, *m/z* = 1260.66 calcd. for $[C_{63}H_{97}N_5O_{19}S + H]^+$, found 1260.42; t_R = 4.16 min, *m/z* = 1328.72 calcd. for $[C_{68}H_{105}N_5O_{19}S + H]^+$, found 1328.58. **HRMS (ESI-TOF)**: *m/z* = 630.8328 calcd. for $[C_{63}H_{97}N_5O_{19}S + 2H]^{2+}$, found 630.8316 [$\delta$ -1.9 ppm]; *m/z* = 664.8641 calcd. for $[C_{68}H_{105}N_5O_{19}S + 2H]^{2+}$, found 664.8644 [$\delta$ 0.5 ppm]. **^{1}H NMR** (399 MHz, CD_3CN) δ 9.24 (s, 1H), 7.50 – 7.43 (m, 4H), 7.33 (t, *J* = 6.2 Hz, 1H), 7.02 (d, *J* = 9.0 Hz, 1H), 6.98 – 6.92 (m, 1H), 5.81 (dd, *J* = 17.7, 10.8 Hz, 1H), 5.31 (s, 1H), 5.14 (dd, *J* = 17.7, 1.4 Hz, 1H), 5.08 (dd, *J* = 10.8, 1.4 Hz, 1H), 4.87 (d, *J* = 4.0 Hz, 1H), 4.57 – 4.42 (m, 4H), 4.29 (dd, *J* = 15.8, 5.5 Hz, 1H), 3.85 – 3.53 (m, 31H), 3.40 – 3.24 (m, 8H), 3.09 – 3.05 (m, 2H), 2.84 – 2.70 (m, 3H), 2.52 – 2.38 (m, 4H), 2.24 – 2.03 (m, 3H), 1.91 – 1.84 (m, 2H), 1.53 – 1.48 (m, 3H), 1.23 – 1.16 (m, 10H), 1.02 – 0.92 (m, 12H), 0.79 (d, *J* = 7.2 Hz, 3H) ppm. **^{13}C NMR** (100 MHz, CD_3CN) δ 172.9, 172.7, 172.1, 171.7, 154.2, 145.2, 145.2, 143.1, 141.9, 141.7, 138.7, 135.1, 130.1, 129.1, 128.9, 127.3, 114.2, 103.2, 82.0, 79.3, 79.1, 76.5, 76.0, 74.5, 73.6, 73.4, 72.1, 71.0, 70.9, 70.8, 70.7, 67.9, 67.8, 63.6, 60.2, 58.6, 58.1, 57.6, 54.2, 49.5, 44.8, 43.1, 42.4, 42.2, 38.4, 37.2, 37.0, 36.7, 36.4, 36.2, 34.7, 28.3, 26.8, 26.3, 24.3, 16.6, 14.8, 10.0 ppm.

CV4p (N^1-((*S*)-2-((1*S*,4*R*,5*R*,6*R*,6a*S*,9*S*,10a*R*,*E*)-1,5-dihydroxy-9-(methoxymethyl)-6,10a-dimethyl-4-(((2*S*,3*R*,4*S*,5*S*,6*R*)-3,4,5-trihydroxy-6-(((2-methylbut-3-en-2-yl)oxy)methyl)tetrahydro-2*H*-pyran-2-yl)oxy)-1,2,4,5,6,6a,7,8,9,10a-decahydrodicyclopenta[*a*,*d*][8]annulen-3-yl)propyl)-N^{25}-((*S*)-1-((2*S*,4*R*)-4-hydroxy-2-((4-(4-methylthiazol-5-yl)benzyl)carbamoyl)pyrrolidin-1-yl)-3,3-dimethyl-1-oxobutan-2-yl)-4,7,10,13,16,19,22-heptaoxapentacosanediamide)

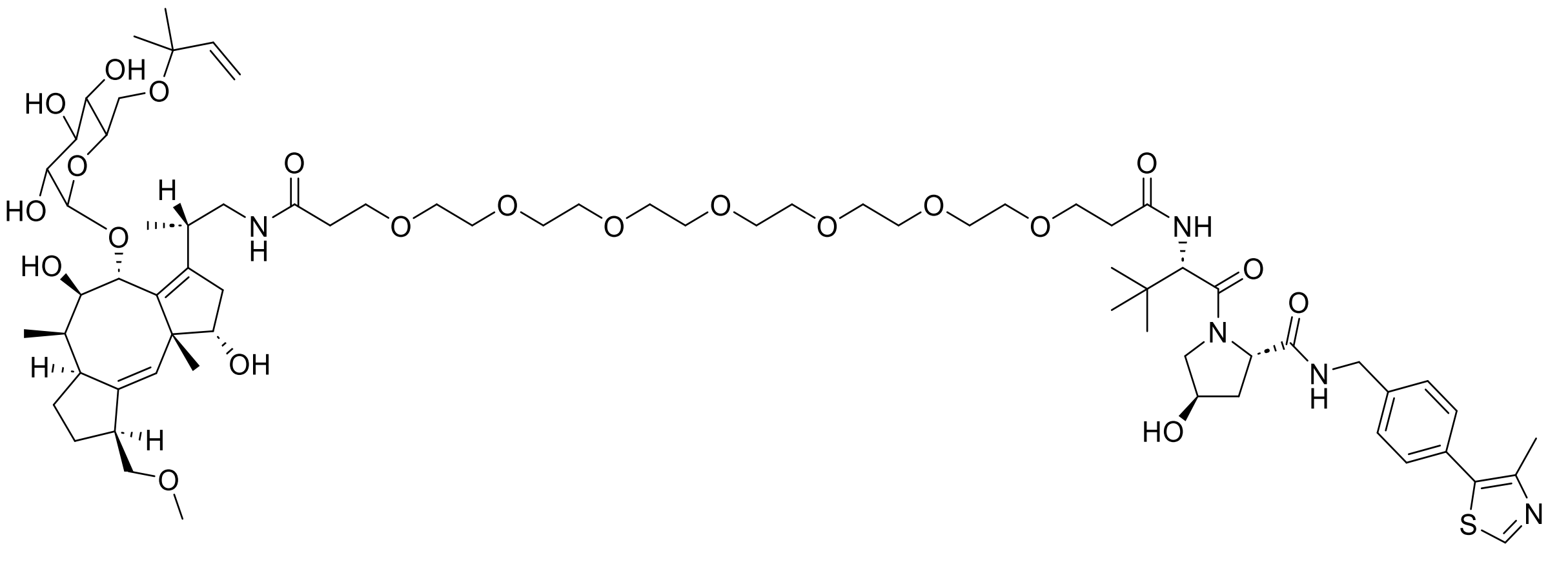

The intermediate **VHL4p** was synthesized via general procedure **B** using 4,7,10,13,16,19,22-heptaoxapentacosanedioic acid (274 mg, 0.642 mmol, 3 eq), HATU (228 mg, 0.600 mmol, 2.8 eq) DIPEA (1.12 mL, 6.42 mmol, 30 eq) and VHL ligand 1 HCl salt (100 mg, 0.214 mmol, 1 eq) in dry DMF (5 mL). The crude product was purified by reversed-phase preparative HPLC (35-40% acetonitrile (+0.1% FA) in water (+0.1% FA) gradient) to afford intermediate **VHL4p** as a white solid (58.0 mg, 0.0692 mmol, 32.3% yield). **LC-MS (ESI)**: t_R = 3.59 min, *m/z* = 839.41 calcd. for $[C_{40}H_{62}N_4O_{13}S + H]^+$, found 839.50. **HRMS (ESI-TOF)**: *m/z* = 839.4113 calcd. for $[C_{40}H_{62}N_4O_{13}S + H]^+$, found 839.4113 [$\delta$ 0.0 ppm].

CV4p was synthesized via general procedure **C** using **VHL4p** (54.2 mg, 0.0646 mmol, 1.1 eq), HATU (29.0 mg, 0.0764 mmol, 1.3 eq), DIPEA (102 μL, 0.587 mmol, 10 eq) and **FC-NH₂** (35 mg, 0.0588

mmol, 1 eq) in dry DMF (5 mL). The crude product was purified by reversed-phase preparative HPLC (45-50% acetonitrile (+0.1% FA) in water (+0.1% FA) gradient) to afford **CV4p** as a white solid (26.1 mg, 18.5 µmol, 31.4% yield), 5% 2-methylbut-3-en-2-yl ether cleaved. **LC-MS (ESI)**: t_R = 3.77 min, *m/z* = 1348.71 calcd. for $[C_{67}H_{105}N_5O_{21}S + H]^+$, found 1348.42; t_R = 4.16 min, *m/z* = 1416.77 calcd. for $[C_{72}H_{113}N_5O_{21}S + H]^+$, found 1416.58. **HRMS (ESI-TOF)**: *m/z* = 674.8589 calcd. for $[C_{67}H_{105}N_5O_{21}S + 2H]^{2+}$, found 674.8579 [$\delta$ -1.5 ppm]; *m/z* = 708.8903 calcd. for $[C_{72}H_{113}N_5O_{21}S + 2H]^{2+}$, found 708.8897 [$\delta$ -0.8 ppm]. **^{1}H NMR** (399 MHz, CD_3CN) δ 9.28 (s, 1H), 7.51 – 7.44 (m, 4H), 7.31 (t, *J* = 6.2 Hz, 1H), 7.01 (d, *J* = 9.0 Hz, 1H), 6.97 – 6.89 (m, 1H), 5.81 (dd, *J* = 17.7, 10.8 Hz, 1H), 5.32 – 5.29 (m, 1H), 5.14 (dd, *J* = 17.7, 1.4 Hz, 1H), 5.08 (dd, *J* = 10.8, 1.4 Hz, 1H), 4.87 (d, *J* = 3.8 Hz, 1H), 4.59 – 4.41 (m, 4H), 4.28 (dd, *J* = 15.8, 5.5 Hz, 1H), 3.82 – 3.52 (m, 39H), 3.42 – 3.21 (m, 8H), 3.10 – 3.04 (m, 2H), 2.80 – 2.72 (m, 3H), 2.52 – 2.42 (m, 4H), 2.22 – 2.03 (m, 3H), 1.92 – 1.85 (m, 2H), 1.54 – 1.47 (m, 3H), 1.24 – 1.15 (m, 10H), 1.02 – 0.94 (m, 12H), 0.79 (d, *J* = 7.2 Hz, 3H) ppm. **^{13}C NMR** (100 MHz, CD_3CN) δ 173.0, 172.6, 172.0, 171.7, 154.4, 145.2, 145.1, 143.1, 141.9, 141.8, 138.8, 135.1, 130.1, 129.0, 128.9, 127.3, 114.2, 103.2, 82.0, 79.3, 79.0, 76.5, 76.0, 74.4, 73.6, 73.4, 72.1, 71.0, 71.0, 71.0, 70.9, 70.9, 70.8, 70.6, 67.9, 67.8, 63.6, 60.2, 58.6, 58.1, 57.6, 54.2, 49.5, 44.8, 43.1, 42.4, 42.2, 38.4, 37.2, 37.0, 36.7, 36.4, 36.2, 34.7, 28.3, 26.7, 26.3, 26.3, 24.3, 16.6, 14.7, 10.0 ppm.

CV0a (N^1-((*S*)-2-((1*S*,4*R*,5*R*,6*R*,6a*S*,9*S*,10a*R*,*E*)-1,5-dihydroxy-9-(methoxymethyl)-6,10a-dimethyl-4-(((2*S*,3*R*,4*S*,5*S*,6*R*)-3,4,5-trihydroxy-6-(((2-methylbut-3-en-2-yl)oxy)methyl)tetrahydro-2*H*-pyran-2-yl)oxy)-1,2,4,5,6,6a,7,8,9,10a-decahydrodicyclopenta[*a*,*d*][8]annulen-3-yl)propyl)-N^4-((*S*)-1-((2*S*,4*R*)-4-hydroxy-2-((4-(4-methylthiazol-5-yl)benzyl)carbamoyl)pyrrolidin-1-yl)-3,3-dimethyl-1-oxobutan-2-yl)succinamide)

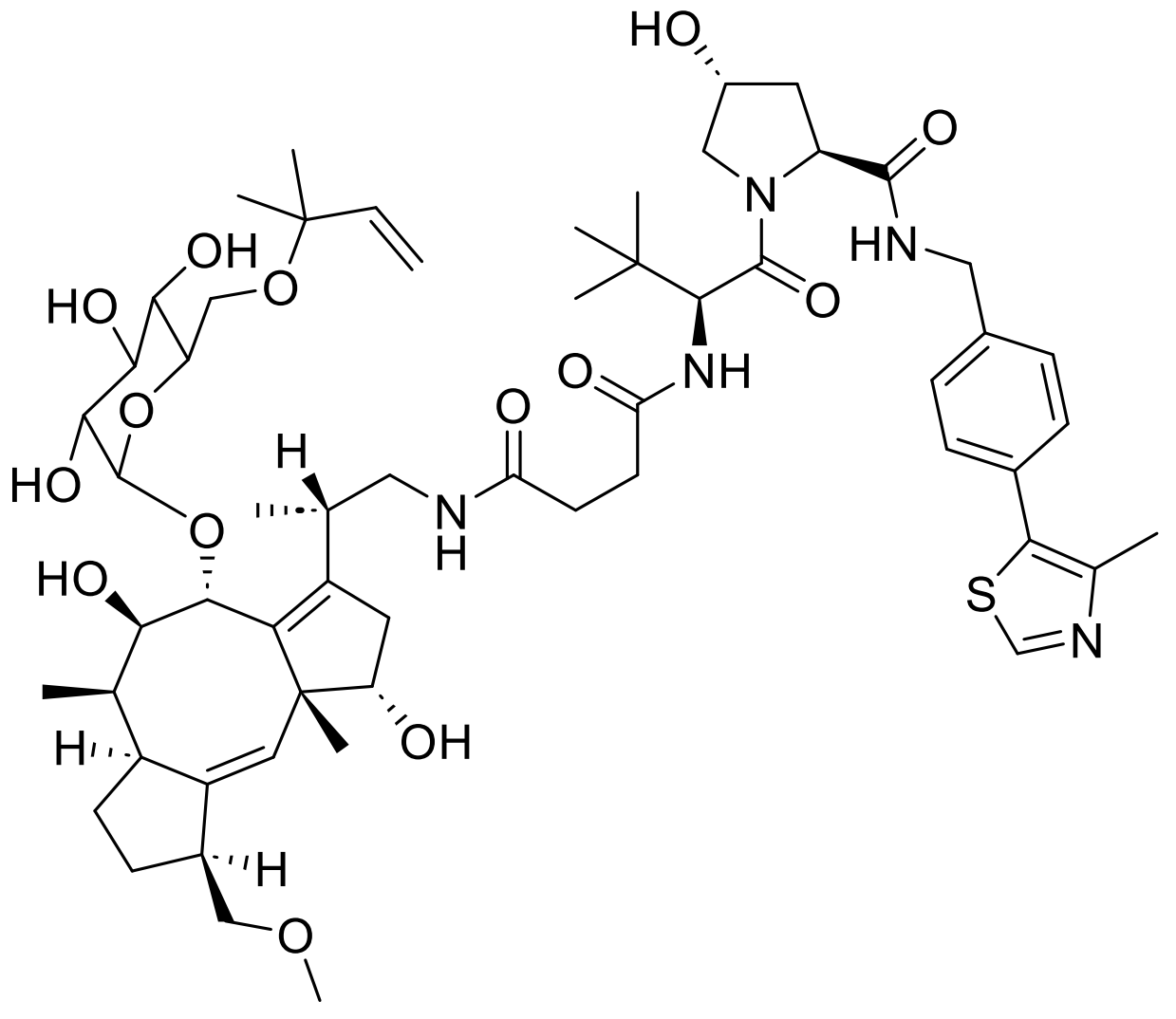

To a solution of VHL ligand 1 HCl salt (100mg, 0.214 mmol, 1 eq) and DIPEA (186 µL, 1.07 mmol, 5 eq) in dry chloroform (5 mL) was added succinic anhydride (25.7 mg, 0.257 mmol, 1.2 eq). The resulting solution was stirred for 3 h and subsequently concentrated under reduced pressure. The crude product was purified by reversed-phase preparative HPLC (30-35% acetonitrile (+0.1% FA) in water (+0.1% FA) gradient) to afford intermediate **VHL0a** as a white solid (77.5 mg, 0.146 mmol, 68.2% yield). **LC-MS (ESI)**: t_R = 3.38 min, *m/z* = 531.23 calcd. for $[C_{26}H_{34}N_4O_6S + H]^+$, found 531.25. **HRMS (ESI-TOF)**: *m/z* = 531.2277 calcd. for $[C_{26}H_{34}N_4O_6S + H]^+$, found 531.2272 [$\delta$ -0.9 ppm].

CV0a was synthesized via general procedure **C** using **VHL0a** (34.3 mg, 0.0646 mmol, 1.1 eq), HATU (29.0 mg, 0.0764 mmol, 1.3 eq), DIPEA (102 µL, 0.587 mmol, 10 eq) and **FC-NH₂** (35 mg, 0.0588 mmol, 1 eq) in dry DMF (5 mL). The crude product was purified by reversed-phase preparative HPLC (40-45% acetonitrile (+0.1% FA) in water (+0.1% FA) gradient) to afford **CV0a** as a white solid (8.46

mg, 7.64 μmol, 13.0% yield), 43% 2-methylbut-3-en-2-yl ether cleaved. **LC-MS (ESI)**: t_R = 3.73 min, *m/z* = 1040.53 calcd. for [$C_{53}H_{77}N_5O_{14}S$ + H]$^+$, found 1040.33; t_R = 4.19 min, *m/z* = 1108.59 calcd. for [$C_{58}H_{85}N_5O_{14}S$ + H]$^+$, found 1108.33. **HRMS (ESI-TOF)**: *m/z* = 520.7672 calcd. for [$C_{53}H_{77}N_5O_{14}S$ + 2H]$^{2+}$, found 520.7668 [*δ* -0.8 ppm], *m/z* = 554.7985 calcd. for [$C_{58}H_{85}N_5O_{14}S$ + 2H]$^{2+}$, found 554.7987 [*δ* 0.4 ppm]. ^{1}**H NMR** (399 MHz, CD_3CN) *δ* 9.50 (s, 1H), 7.50 – 7.44 (m, 4H), 7.01 – 6.97 (m, 1H), 5.82 (dd, *J* = 17.7, 10.8 Hz, 1H), 5.33 – 5.26 (m, 1H), 5.14 (dd, *J* = 17.7, 1.4 Hz, 1H), 5.08 (dd, *J* = 10.8, 1.4 Hz, 1H), 4.93 – 4.84 (m, 1H), 4.60 – 4.29 (m, 5H), 4.00 – 3.91 (m, 1H), 3.84 – 3.62 (m, 6H), 3.49 – 3.37 (m, 3H), 3.30 – 3.23 (m, 5H), 3.18 – 3.08 (m, 2H), 2.87 – 2.75 (m, 1H), 2.54 – 2.47 (m, 9H), 2.22 – 2.13 (m, 2H), 2.11 – 1.98 (m, 2H), 1.93 – 1.81 (m, 3H), 1.74 – 1.62 (m, 1H), 1.54 – 1.42 (m, 3H), 1.29 – 1.10 (m, 8H), 0.99 (d, *J* = 8.4 Hz, 12H), 0.78 (d, *J* = 2.6 Hz, 3H) ppm. ^{13}**C NMR** (100 MHz, CD_3CN) *δ* 173.7, 173.6, 172.7, 171.6, 155.2, 144.8, 142.8, 142.2, 141.6, 138.4, 136.5, 129.8, 128.6, 127.2, 127.0, 113.8, 102.7, 81.5, 78.8, 78.6, 76.2, 75.7, 74.2, 73.0, 72.8, 72.1, 70.7, 63.0, 59.9, 58.6, 58.3, 57.5, 53.9, 49.1, 44.8, 42.7, 42.0, 41.8, 38.0, 36.2, 36.0, 35.5, 33.9, 30.7, 30.6, 27.9, 26.4, 25.9, 25.9, 23.9, 16.0, 13.2, 9.6 ppm.

CV1a (*N*[1]-((*S*)-2-((1*S*,4*R*,5*R*,6*R*,6a*S*,9*S*,10a*R*,*E*)-1,5-dihydroxy-9-(methoxymethyl)-6,10a-dimethyl-4-(((2*S*,3*R*,4*S*,5*S*,6*R*)-3,4,5-trihydroxy-6-(((2-methylbut-3-en-2-yl)oxy)methyl)tetrahydro-2*H*-pyran-2-yl)oxy)-1,2,4,5,6,6a,7,8,9,10a-decahydrodicyclopenta[*a*,*d*][8]annulen-3-yl)propyl)-*N*[7]-((*S*)-1-((2*S*,4*R*)-4-hydroxy-2-((4-(4-methylthiazol-5-yl)benzyl)carbamoyl)pyrrolidin-1-yl)-3,3-dimethyl-1-oxobutan-2-yl)heptanediamide)

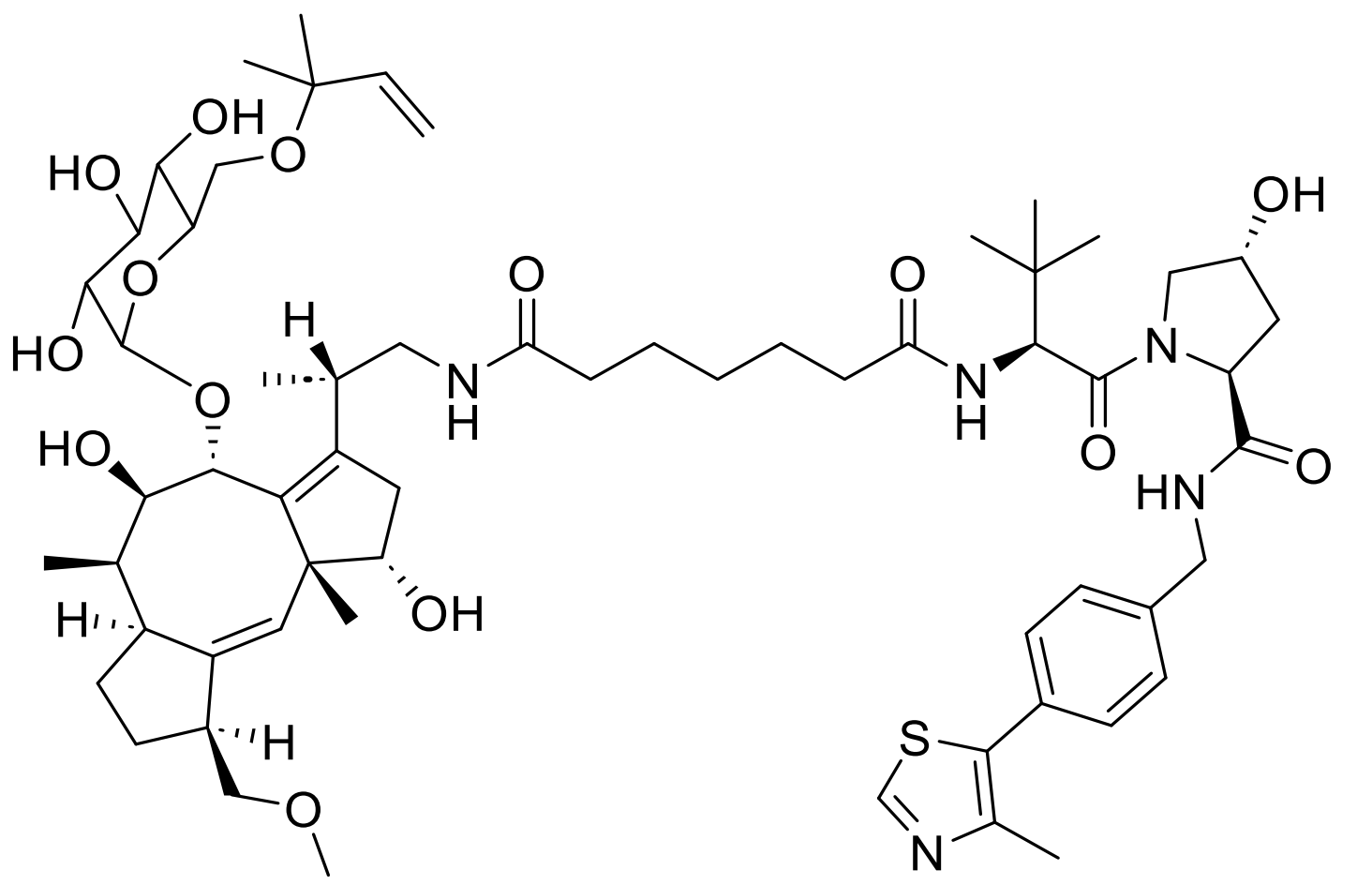

The intermediate **VHL1a** was synthesized via general procedure **B** using heptanedioic acid (171 mg, 1.07 mmol, 5 eq), HATU (366 mg, 0.964 mmol, 4.5 eq) DIPEA (1.12 mL, 6.42 mmol, 30 eq) and VHL ligand 1 HCl salt (100 mg, 0.214 mmol, 1 eq) in dry DMF (10 mL). The crude product was purified by reversed-phase preparative HPLC (30-35% acetonitrile (+0.1% FA) in water (+0.1% FA) gradient) to afford intermediate **VHL1a** as a white solid (50.5 mg, 0.0882 mmol, 41.2% yield). **LC-MS (ESI)**: t_R = 3.58 min, *m/z* = 573.27 calcd. for [$C_{29}H_{40}N_4O_6S$ + H]$^+$, found 573.42. **HRMS (ESI-TOF)**: *m/z* = 573.2747 calcd. for [$C_{29}H_{40}N_4O_6S$ + H]$^+$, found 573.2733 [*δ* -2.4 ppm].

CV1a was synthesized via general procedure **C** using **VHL1a** (37.0 mg, 0.0646 mmol, 1.1 eq), HATU (29.0 mg, 0.0764 mmol, 1.3 eq), DIPEA (102 μL, 0.587 mmol, 10 eq) and **FC-NH₂** (35 mg, 0.0588 mmol, 1 eq) in dry DMF (5 mL). The crude product was purified by reversed-phase preparative HPLC (40-45% acetonitrile (+0.1% FA) in water (+0.1% FA) gradient) to afford **CV1a** as a white solid (8.21 mg, 7.14 μmol, 12.2% yield), 7% 2-methylbut-3-en-2-yl ether cleaved. **LC-MS (ESI)**: t_R = 3.79 min, *m/z* = 1082.57 calcd. for [$C_{56}H_{83}N_5O_{14}S$ + H]$^+$, found 1082.42; t_R = 4.24 min, *m/z* = 1150.64 calcd. for [$C_{61}H_{91}N_5O_{14}S$ + H]$^+$, found 1150.58. **HRMS (ESI-TOF)**: *m/z* = 541.7907 calcd. for [$C_{56}H_{83}N_5O_{14}S$ + 2H]$^{2+}$, found 541.7891 [*δ* -3.0 ppm]; *m/z* = 575.8220 calcd. for [$C_{61}H_{91}N_5O_{14}S$ + 2H]$^{2+}$, found 575.8218 [*δ* -0.3 ppm]. ^{1}**H NMR** (399 MHz, DMSO) *δ* 9.01 (s, 1H), 8.56 (t, *J* = 6.1 Hz, 1H), 7.83 (d, *J* = 9.3 Hz,

1H), 7.63 – 7.56 (m, 1H), 7.43 – 7.37 (m, 4H), 5.76 (dd, *J* = 17.7, 10.8 Hz, 1H), 5.34 – 5.26 (m, 1H), 5.11 (dd, *J* = 17.8, 1.5 Hz, 1H), 5.06 (dd, *J* = 10.8, 1.4 Hz, 1H), 4.73 (d, *J* = 3.8 Hz, 1H), 4.54 (d, *J* = 9.3 Hz, 1H), 4.46 – 4.40 (m, 2H), 4.36 – 4.33 (m, 1H), 4.21 (dd, *J* = 16.1, 5.3 Hz, 1H), 3.45 – 3.42 (m, 2H), 3.31 – 3.10 (m, 10H), 3.06 – 2.95 (m, 3H), 2.66 – 2.59 (m, 2H), 2.45 – 2.44 (m, 3H), 2.28 – 2.21 (m, 1H), 2.15 – 1.98 (m, 6H), 1.93 – 1.80 (m, 4H), 1.55 – 1.35 (m, 8H), 1.32 – 1.00 (m, 13H), 0.94 – 0.87 (m, 12H), 0.72 (d, *J* = 7.0 Hz, 3H) ppm. **^{13}C NMR** (100 MHz, DMSO) δ 172.6, 172.1, 172.0, 169.7, 151.6, 147.5, 144.2, 140.9, 139.6, 139.5, 137.1, 131.3, 129.5, 128.7, 127.4, 127.1, 113.7, 102.2, 80.8, 77.9, 77.6, 75.3, 74.6, 73.1, 72.2, 72.1, 70.5, 68.9, 62.5, 58.7, 57.9, 56.4, 56.3, 52.4, 48.2, 42.7, 41.7, 41.0, 38.0, 35.4, 35.3, 35.2, 35.2, 34.9, 33.5, 28.5, 27.1, 26.4, 25.9, 25.7, 25.3, 25.2, 23.3, 16.0, 15.8, 9.2 ppm.

CV2a (*N*^{1}-((*S*)-2-((1*S*,4*R*,5*R*,6*R*,6a*S*,9*S*,10a*R*,*E*)-1,5-dihydroxy-9-(methoxymethyl)-6,10a-dimethyl-4-(((2*S*,3*R*,4*S*,5*S*,6*R*)-3,4,5-trihydroxy-6-(((2-methylbut-3-en-2-yl)oxy)methyl)tetrahydro-2*H*-pyran-2-yl)oxy)-1,2,4,5,6,6a,7,8,9,10a-decahydrodicyclopenta[*a*,*d*][8]annulen-3-yl)propyl)-*N*^{13}-((*S*)-1-((2*S*,4*R*)-4-hydroxy-2-((4-(4-methylthiazol-5-yl)benzyl)carbamoyl)pyrrolidin-1-yl)-3,3-dimethyl-1-oxobutan-2-yl)tridecanediamide)

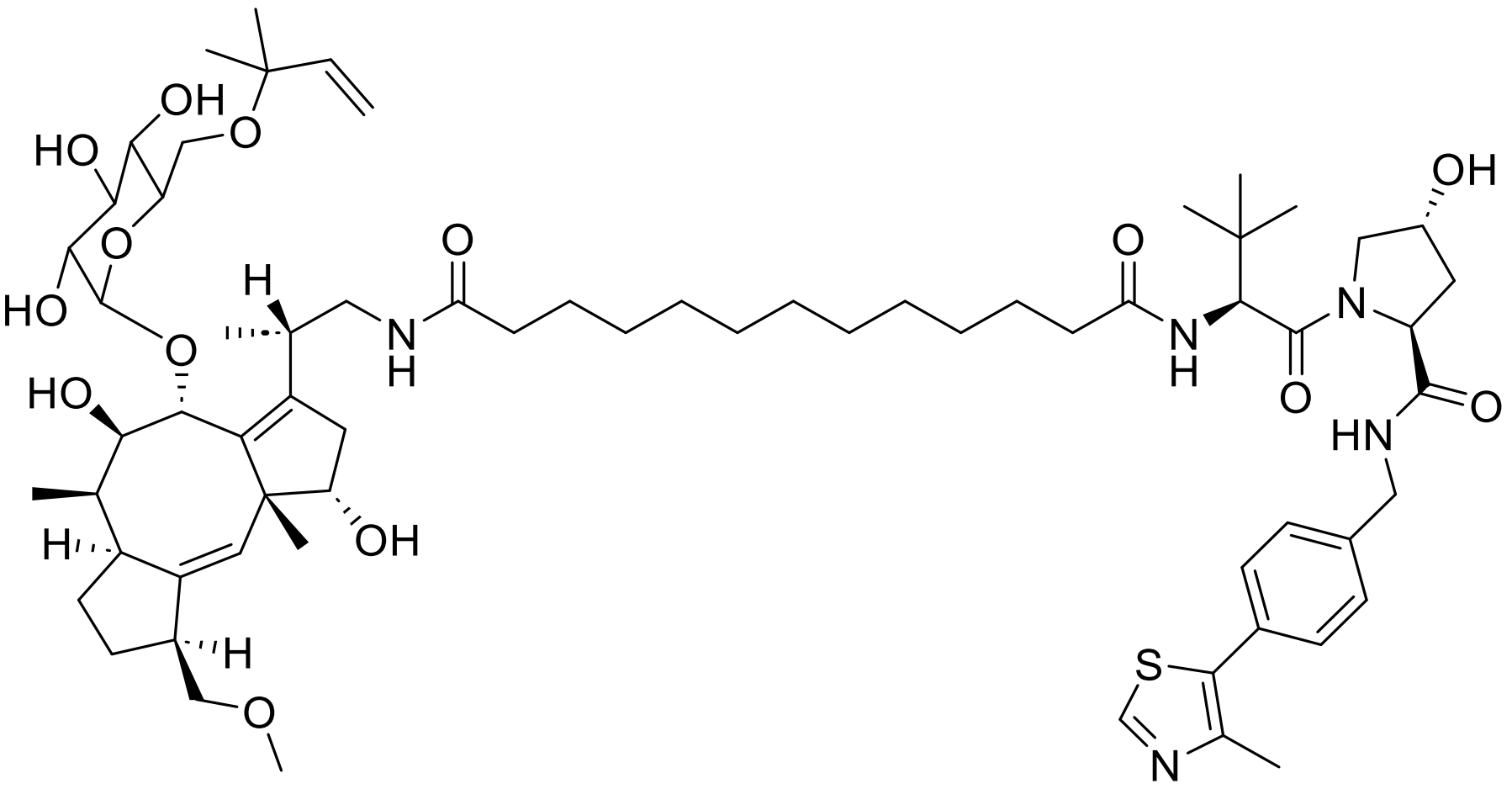

The intermediate **VHL2a** was synthesized via general procedure **B** using tridecanedioic acid (262 mg, 1.07 mmol, 5 eq), HATU (366 mg, 0.964 mmol, 4.5 eq) DIPEA (1.12 mL, 6.42 mmol, 30 eq) and VHL ligand 1 HCl salt (100 mg, 0.214 mmol, 1 eq) in dry DMF (15 mL). The crude product was purified by reversed-phase preparative HPLC (45-50% acetonitrile (+0.1% FA) in water (+0.1% FA) gradient) to afford intermediate **VHL2a** as a white solid (54.2 mg, 0.0825 mmol, 38.5% yield). **LC-MS (ESI)**: t_R = 4.44 min, *m/z* = 657.37 calcd. for $[C_{35}H_{52}N_4O_6S + H]^+$, found 657.50. **HRMS (ESI-TOF)**: *m/z* = 657.3686 calcd. for $[C_{35}H_{52}N_4O_6S + H]^+$, found 657.3676 [$\delta$ -1.5 ppm].

CV2a was synthesized via general procedure **C** using **VHL2a** (42.5 mg, 0.0646 mmol, 1.1 eq), HATU (29.0 mg, 0.0764 mmol, 1.3 eq), DIPEA (102 µL, 0.587 mmol, 10 eq) and **FC-NH$_2$** (35 mg, 0.0588 mmol, 1 eq) in dry DMF (5 mL). The crude product was purified by reversed-phase preparative HPLC (50-55% acetonitrile (+0.1% FA) in water (+0.1% FA) gradient) to afford **CV2a** as a white solid (6.75 mg, 5.46 µmol, 9.30% yield), 14% 2-methylbut-3-en-2-yl ether cleaved. **LC-MS (ESI)**: t_R = 4.26 min, *m/z* = 1166.67 calcd. for $[C_{62}H_{95}N_5O_{14}S + H]^+$, found 1166.58; t_R = 4.69 min, *m/z* = 1234.73 calcd. for $[C_{67}H_{103}N_5O_{14}S + H]^+$, found 1234.67. **HRMS (ESI-TOF)**: *m/z* = 583.8376 calcd. for $[C_{62}H_{95}N_5O_{14}S + 2H]^{2+}$, found 583.8370 [$\delta$ -1.0 ppm], *m/z* = 617.8690 calcd. for $[C_{67}H_{103}N_5O_{14}S + 2H]^{2+}$, found 617.8689 [$\delta$ -0.2 ppm]. **^{1}H NMR** (399 MHz, DMSO) δ 9.02 (s, 1H), 8.55 (t, *J* = 6.1 Hz, 1H), 7.82 (d, *J* = 9.3 Hz, 1H), 7.59 – 7.51 (m, 1H), 7.43 – 7.37 (m, 4H), 5.77 (dd, *J* = 17.7, 10.8 Hz, 1H), 5.30 (s, 1H), 5.11 (dd, *J* = 17.7, 1.5 Hz, 1H), 5.06 (dd, *J* = 10.8, 1.4 Hz, 1H), 4.73 (d, *J* = 3.8 Hz, 1H), 4.54 (d, *J* = 9.4 Hz, 1H), 4.41 – 4.35 (m, 3H), 4.23 (dd, *J* = 16.1, 5.3 Hz, 1H), 3.72 – 3.58 (m, 6H), 3.51 – 3.37 (m, 3H), 3.27 – 3.13 (m, 8H), 3.09 – 2.96 (m, 3H), 2.68 – 2.59 (m, 2H), 2.46 – 2.42 (m, 3H), 2.30 – 1.96 (m, 7H), 1.93

– 1.78 (m, 4H), 1.51 – 1.40 (m, 7H), 1.25 – 1.13 (m, 21H), 0.94 – 0.86 (m, 12H), 0.72 (d, *J* = 7.0 Hz, 3H) ppm. **^{13}C NMR** (100 MHz, DMSO) *δ* 172.5, 172.1, 171.9, 169.7, 151.6, 147.5, 144.1, 140.9, 139.6, 139.5, 137.1, 131.3, 129.5, 128.6, 127.4, 127.0, 113.6, 102.2, 80.8, 77.8, 77.6, 75.3, 74.5, 73.1, 72.2, 72.0, 70.5, 68.8, 62.5, 58.7, 57.9, 56.3, 56.3, 52.4, 48.2, 42.7, 41.6, 41.0, 37.9, 35.4, 35.2, 35.2, 34.9, 33.4, 29.0, 29.0, 28.8, 28.8, 28.7, 27.1, 26.4, 25.8, 25.7, 25.4, 25.4, 23.3, 16.0, 15.8, 9.2 ppm.

CV3a (*N*^{1}-((*S*)-2-((1*S*,4*R*,5*R*,6*R*,6a*S*,9*S*,10a*R*,*E*)-1,5-dihydroxy-9-(methoxymethyl)-6,10a-dimethyl-4-(((2*S*,3*R*,4*S*,5*S*,6*R*)-3,4,5-trihydroxy-6-(((2-methylbut-3-en-2-yl)oxy)methyl)tetrahydro-2*H*-pyran-2-yl)oxy)-1,2,4,5,6,6a,7,8,9,10a-decahydrodicyclopenta[*a*,*d*][8]annulen-3-yl)propyl)-*N*^{19}-((*S*)-1-((2*S*,4*R*)-4-hydroxy-2-((4-(4-methylthiazol-5-yl)benzyl)carbamoyl)pyrrolidin-1-yl)-3,3-dimethyl-1-oxobutan-2-yl)nonadecanediamide)

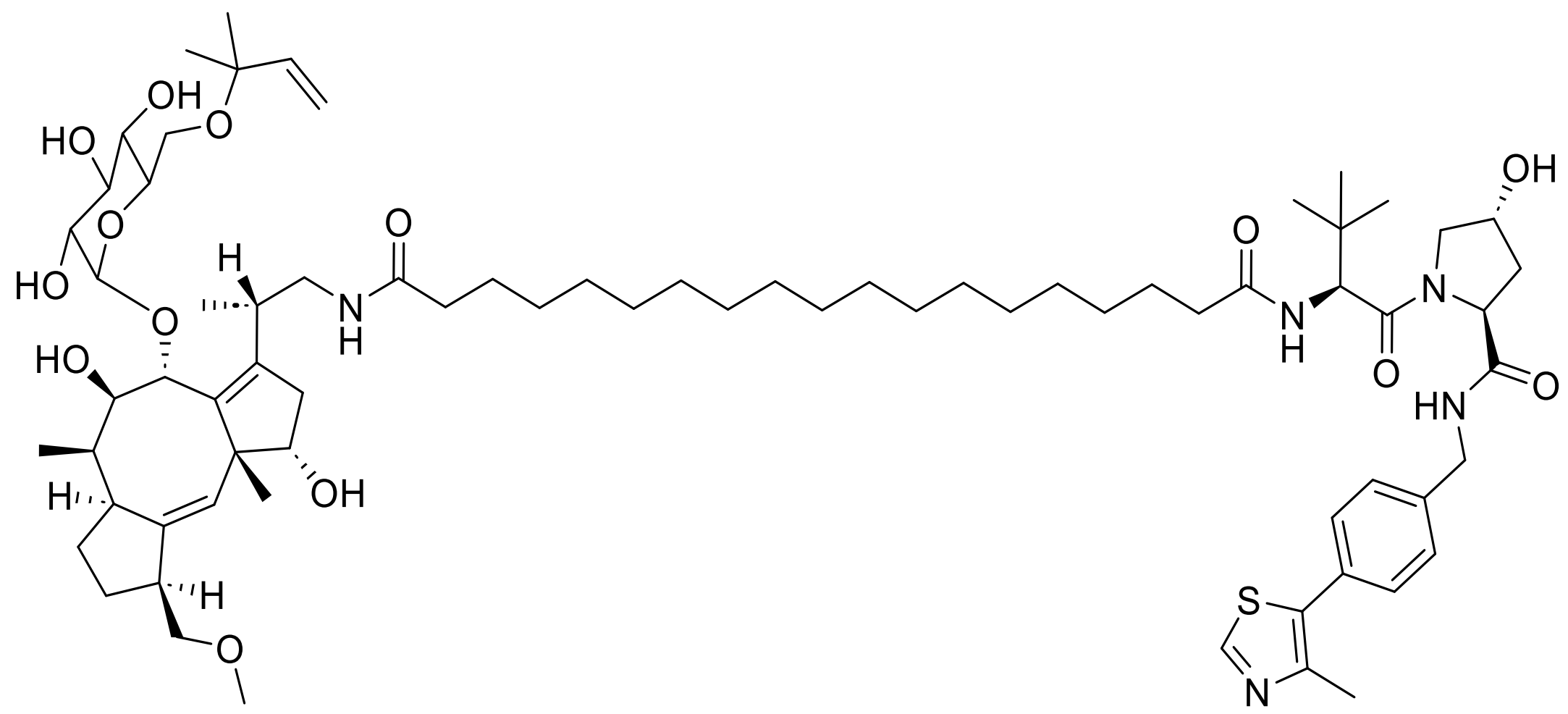

The intermediate **VHL3a** was synthesized via general procedure **B** using nonadecanedioic acid (352 mg, 1.07 mmol, 5 eq), HATU (366 mg, 0.964 mmol, 4.5 eq) DIPEA (1.12 mL, 6.42 mmol, 30 eq) and VHL ligand 1 HCl salt (100 mg, 0.214 mmol, 1 eq) in dry DMF (20 mL). The crude product was purified by reversed-phase preparative HPLC (85-90% acetonitrile (+0.1% FA) in water (+0.1% FA) gradient) to afford intermediate **VHL3a** as a white solid (87.4 mg, 0.118 mmol, 55.1% yield). **LC-MS (ESI)**: t_R = 7.22 min, *m/z* = 741.46 calcd. for $[C_{41}H_{64}N_4O_6S + H]^+$, found 740.92. **HRMS (ESI-TOF)**: *m/z* = 741.4625 calcd. for $[C_{41}H_{64}N_4O_6S + H]^+$, found 741.4627 [*δ* 0.3 ppm].

CV3a was synthesized via general procedure **C** using **VHL3a** (47.9 mg, 0.0646 mmol, 1.1 eq), HATU (29.0 mg, 0.0764 mmol, 1.3 eq), DIPEA (102 µL, 0.587 mmol, 10 eq) and **FC-NH$_2$** (35 mg, 0.0588 mmol, 1 eq) in dry DMF (5 mL). The crude product was purified by reversed-phase preparative HPLC (70-75% acetonitrile (+0.1% FA) in water (+0.1% FA) gradient) to afford **CV3a** as a white solid (6.82 mg, 5.17 µmol, 8.80% yield), 20% 2-methylbut-3-en-2-yl ether cleaved. **LC-MS (ESI)**: t_R = 4.99 min, *m/z* = 1250.76 calcd. for $[C_{68}H_{107}N_5O_{14}S + H]^+$, found 1250.67; t_R = 5.44 min, *m/z* = 1318.82 calcd. for $[C_{73}H_{115}N_5O_{14}S + H]^+$, found 1318.83. **HRMS (ESI-TOF)**: *m/z* = 625.8846 calcd. for $[C_{68}H_{107}N_5O_{14}S + 2H]^{2+}$, found 625.8867 [*δ* 3.4 ppm]; *m/z* = 659.9159 calcd. for $[C_{73}H_{115}N_5O_{14}S + 2H]^{2+}$, found 659.9147 [*δ* -1.8 ppm]. **^{1}H NMR** (399 MHz, DMSO) *δ* 9.05 (s, 1H), 8.55 (t, *J* = 6.1 Hz, 1H), 7.83 (d, *J* = 9.4 Hz, 1H), 7.59 – 7.52 (m, 1H), 7.43 – 7.38 (m, 4H), 5.77 (dd, *J* = 17.7, 10.8 Hz, 1H), 5.30 (s, 1H), 5.13 (dd, *J* = 17.7, 1.5 Hz, 1H), 5.05 (dd, *J* = 10.7, 1.4 Hz, 1H), 4.75 – 4.73 (m, 1H), 4.53 (d, *J* = 2.9 Hz, 1H), 4.44 – 4.34 (m, 3H), 4.21 (dd, *J* = 15.9, 5.3 Hz, 1H), 3.72 – 3.59 (m, 6H), 3.49 – 3.38 (m, 3H), 3.25 – 3.15 (m, 8H), 3.07 – 2.95 (m, 3H), 2.65 – 2.60 (m, 2H), 2.45 (s, 3H), 2.24 – 2.03 (m, 7H), 1.90 – 1.80 (m, 4H), 1.48 – 1.40 (m, 7H), 1.23 – 1.17 (m, 33H), 0.94 – 0.90 (m, 12H), 0.72 (d, *J* = 7.1 Hz, 3H) ppm. **^{13}C NMR** (100 MHz, DMSO) *δ* 172.5, 172.1, 171.9, 169.7, 151.7, 147.3, 144.1, 140.9, 139.6, 139.5, 137.1, 131.4, 129.4, 128.6, 127.4, 127.0, 113.6, 102.2, 80.8, 77.9, 77.6, 75.3, 74.5, 73.1, 72.2, 72.0, 70.5,

68.8, 62.5, 58.7, 57.9, 56.3, 52.4, 48.2, 42.7, 41.6, 41.0, 37.9, 35.3, 35.2, 34.8, 33.4, 29.1, 29.0, 29.0, 29.0, 28.8, 28.7, 28.6, 27.1, 26.4, 25.8, 25.7, 25.4, 25.3, 23.3, 16.0, 15.7, 9.2 ppm.

(S-OH)-CV2a (N1-((S)-2-((1S,4R,5R,6R,6aS,9S,10aR,E)-1,5-dihydroxy-9-(methoxymethyl)-6,10a-dimethyl-4-(((2S,3R,4S,5S,6R)-3,4,5-trihydroxy-6-(((2-methylbut-3-en-2-yl)oxy)methyl)tetrahydro-2H-pyran-2-yl)oxy)-1,2,4,5,6,6a,7,8,9,10a-decahydrodicyclopenta[a,d][8]annulen-3-yl)propyl)-N13-((S)-1-((2S,4S)-4-hydroxy-2-((4-(4-methylthiazol-5-yl)benzyl)carbamoyl)pyrrolidin-1-yl)-3,3-dimethyl-1-oxobutan-2-yl)tridecanediamide)

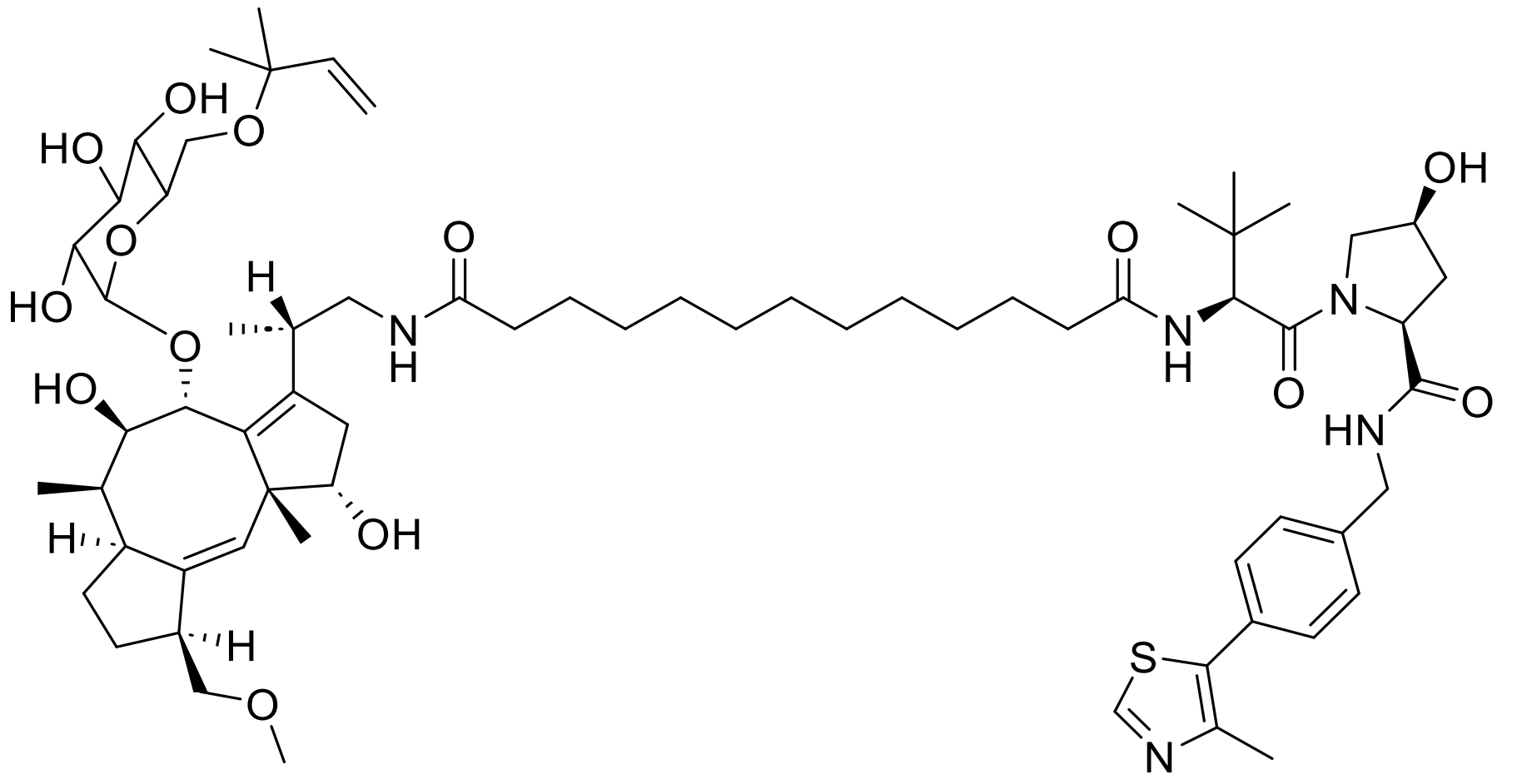

The intermediate **VHL(*S*-OH)3a** was synthesized via general procedure **B** using tridecanedioic acid (262 mg, 1.07 mmol, 5 eq), HATU (366 mg, 0.964 mmol, 4.5 eq) DIPEA (1.12 mL, 6.42 mmol, 30 eq) and VHL(*S*-OH) ligand 1 HCl salt (100 mg, 0.214 mmol, 1 eq) in dry DMF (15 mL). The crude product was purified by reversed-phase preparative HPLC (45-50% acetonitrile (+0.1% FA) in water (+0.1% FA) gradient) to afford intermediate **VHL(*S*-OH)3a** as a white solid (49.2 mg, 0.0749 mmol, 35.0% yield). **LC-MS (ESI)**: t_R = 4.46 min, *m/z* = 657.37 calcd. for $[C_{35}H_{52}N_4O_6S + H]^+$, found 657.58. **HRMS (ESI-TOF)**: *m/z* = 657.3686 calcd. for $[C_{35}H_{52}N_4O_6S + H]^+$, found 657.3685 [$\delta$ -0.2 ppm].

(*S*-OH)-CV2a was synthesized via general procedure **C** using **VHL(*S*-OH)3a** (42.5 mg, 0.0646 mmol, 1.1 eq), HATU (29.0 mg, 0.0764 mmol, 1.3 eq), DIPEA (102 µL, 0.587 mmol, 10 eq) and **FC-NH₂** (35 mg, 0.0588 mmol, 1 eq) in dry DMF (5 mL). The crude product was purified by reversed-phase preparative HPLC (50-55% acetonitrile (+0.1% FA) in water (+0.1% FA) gradient) to afford **(*S*-OH)-CV2a** as a white solid (5.83 mg, 4.72 µmol, 8.03% yield), no 2-methylbut-3-en-2-yl ether cleavage. **LC-MS (ESI)**: t_R = 4.72 min, *m/z* = 1234.73 calcd. for $[C_{67}H_{103}N_5O_{14}S + H]^+$, found 1234.92. **HRMS (ESI-TOF)**: *m/z* = 617.8690 calcd. for $[C_{67}H_{103}N_5O_{14}S + 2H]^{2+}$, found 617.8692 [$\delta$ 0.3 ppm]. **^{1}H NMR** (399 MHz, DMSO) δ 9.02 (s, 1H), 8.62 (t, *J* = 6.1 Hz, 1H), 7.83 (d, *J* = 8.8 Hz, 1H), 7.59 – 7.53 (m, 1H), 7.42 – 7.37 (m, 4H), 5.77 (dd, *J* = 17.7, 10.8 Hz, 1H), 5.31 – 5.28 (m, 1H), 5.11 (dd, *J* = 17.7, 1.5 Hz, 1H), 5.06 (dd, *J* = 10.8, 1.5 Hz, 1H), 4.73 (d, *J* = 3.8 Hz, 1H), 4.46 – 4.44 (m, 1H), 4.37 – 4.36 (m, 1H), 4.29 – 4.26 (m, 1H), 4.24 – 4.18 (m, 2H), 3.93 (dd, *J* = 10.2, 5.7 Hz, 1H), 3.78 – 3.48 (m, 5H), 3.48 – 3.41 (m, 3H), 3.26 – 3.15 (m, 8H), 3.06 – 2.95 (m, 3H), 2.67 – 2.60 (m, 2H), 2.45 (s, 3H), 2.35 – 2.04 (m, 7H), 1.88 – 1.71 (m, 4H), 1.51 – 1.40 (m, 7H), 1.24 – 1.13 (m, 21H), 0.96 – 0.88 (m, 12H), 0.72 (d, *J* = 7.0 Hz, 3H) ppm. **^{13}C NMR** (100 MHz, DMSO) δ 172.5, 172.5, 172.4, 170.0, 151.6, 147.6, 144.1, 140.9, 139.5, 139.3, 137.1, 131.2, 129.6, 128.7, 127.4, 127.0, 113.6, 102.2, 80.8, 77.9, 77.6, 75.3, 74.6, 73.1, 72.2, 72.1, 70.5, 69.1, 62.5, 58.5, 57.9, 56.6, 55.6, 52.4, 48.2, 42.7, 41.8, 41.0, 36.9, 35.4, 35.2, 34.8, 34.7, 33.4, 29.0, 28.9, 28.8, 28.8, 28.7, 27.1, 26.4, 25.8, 25.7, 25.4, 25.4, 23.3, 16.0, 15.9, 9.2 ppm.

NMR spectra

^{1}H NMR spectrum of FC-NAc

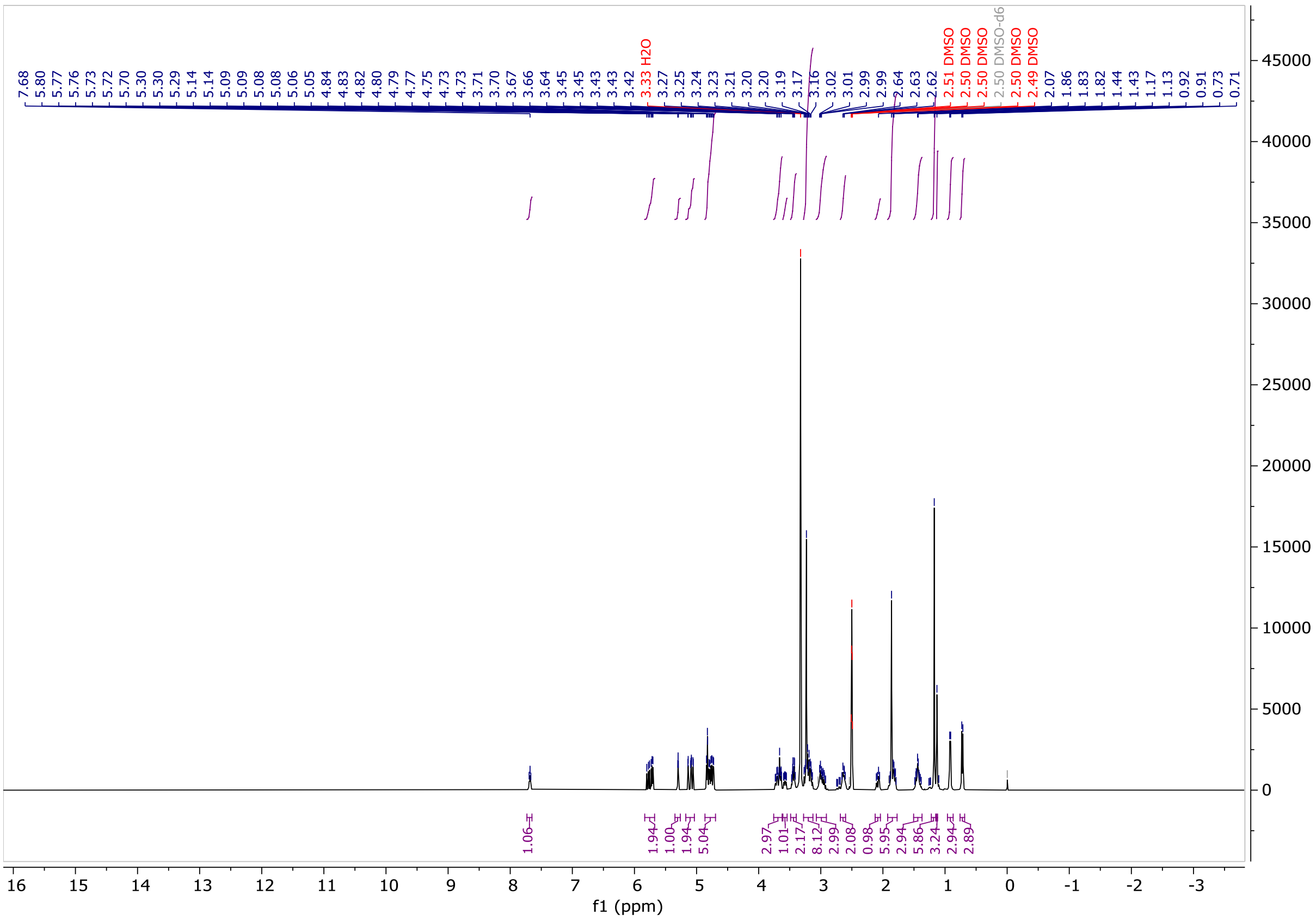

13C NMR spectrum of FC-NAc

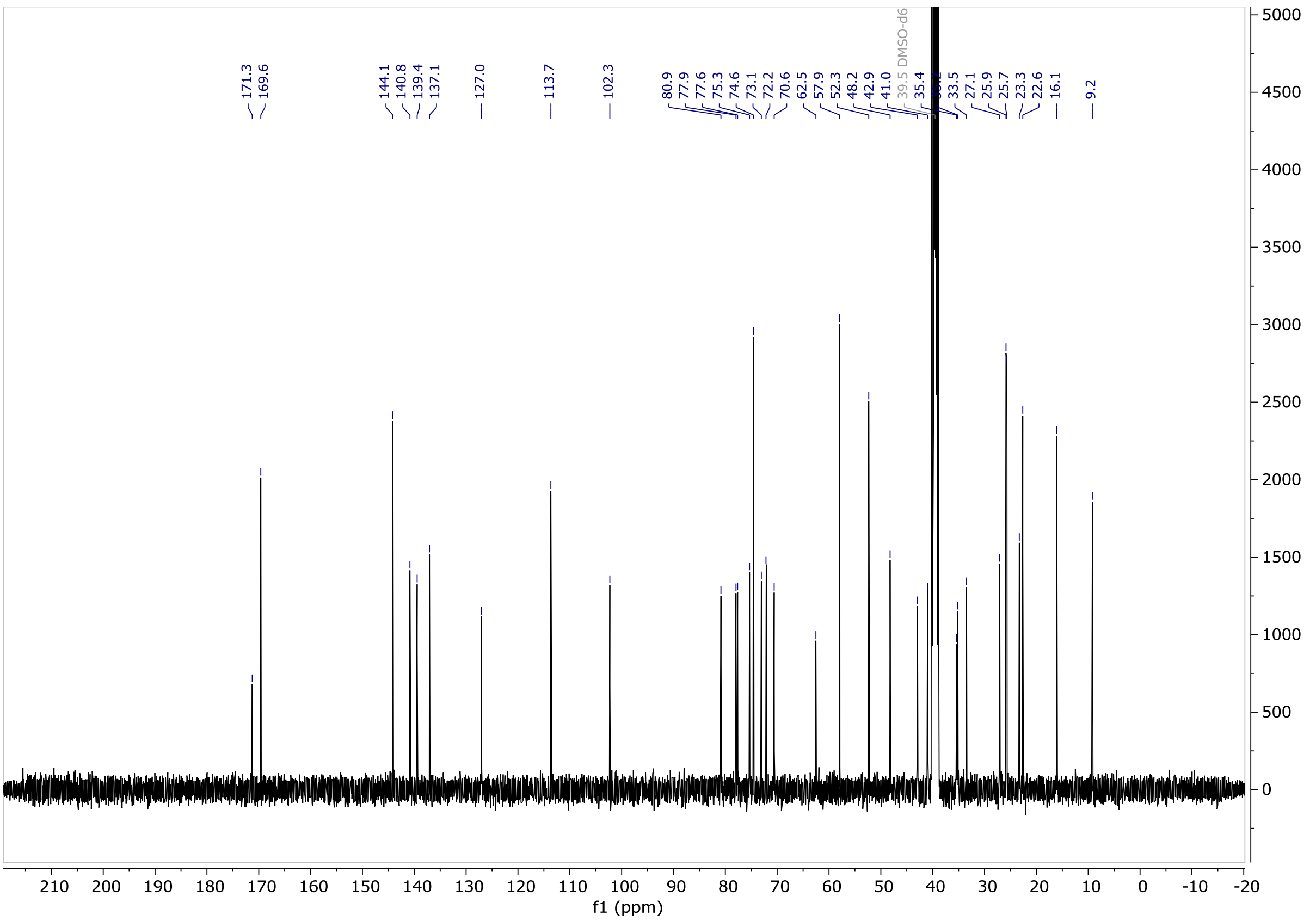

1H NMR spectrum of VH032

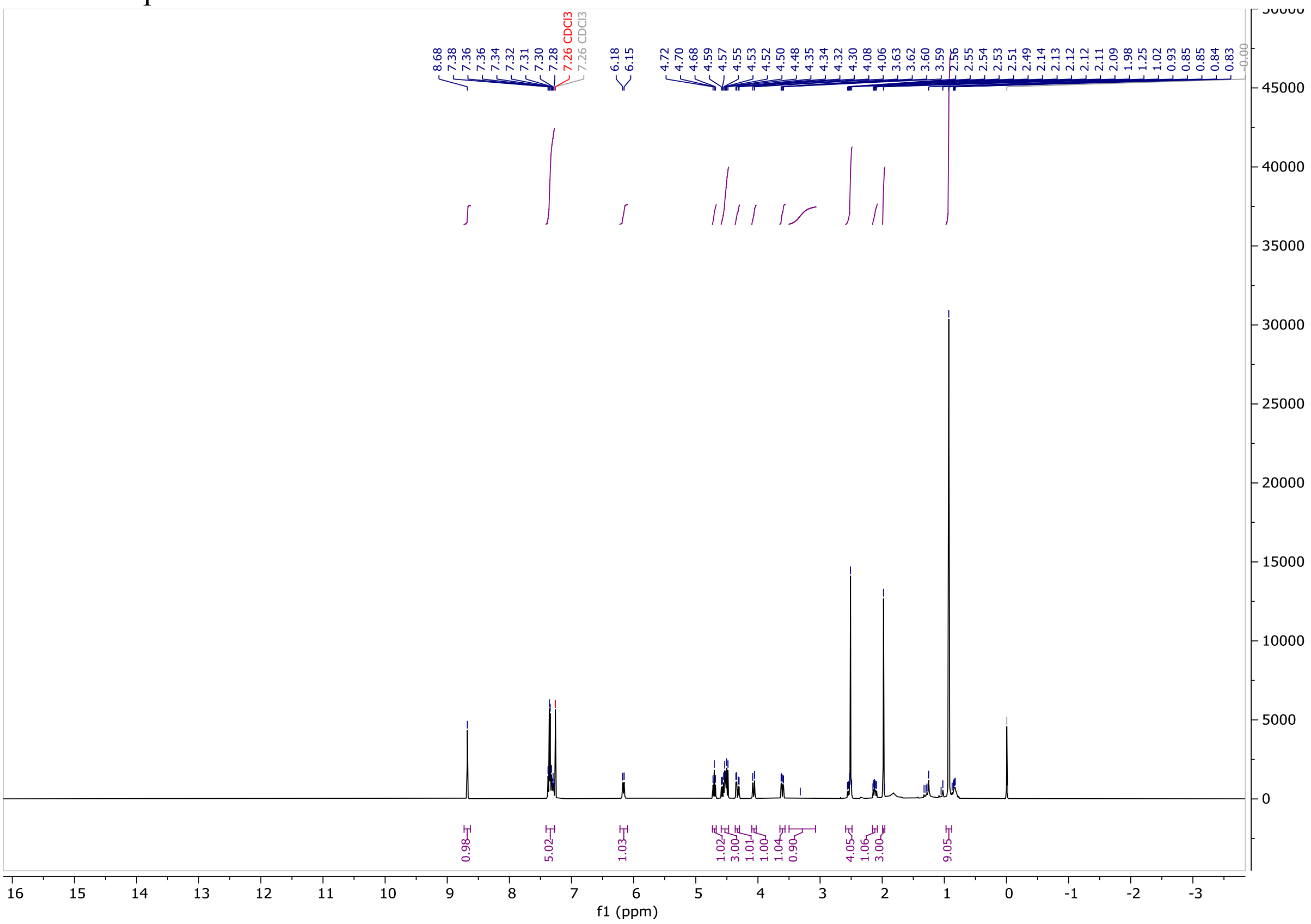

13C NMR spectrum of VH032

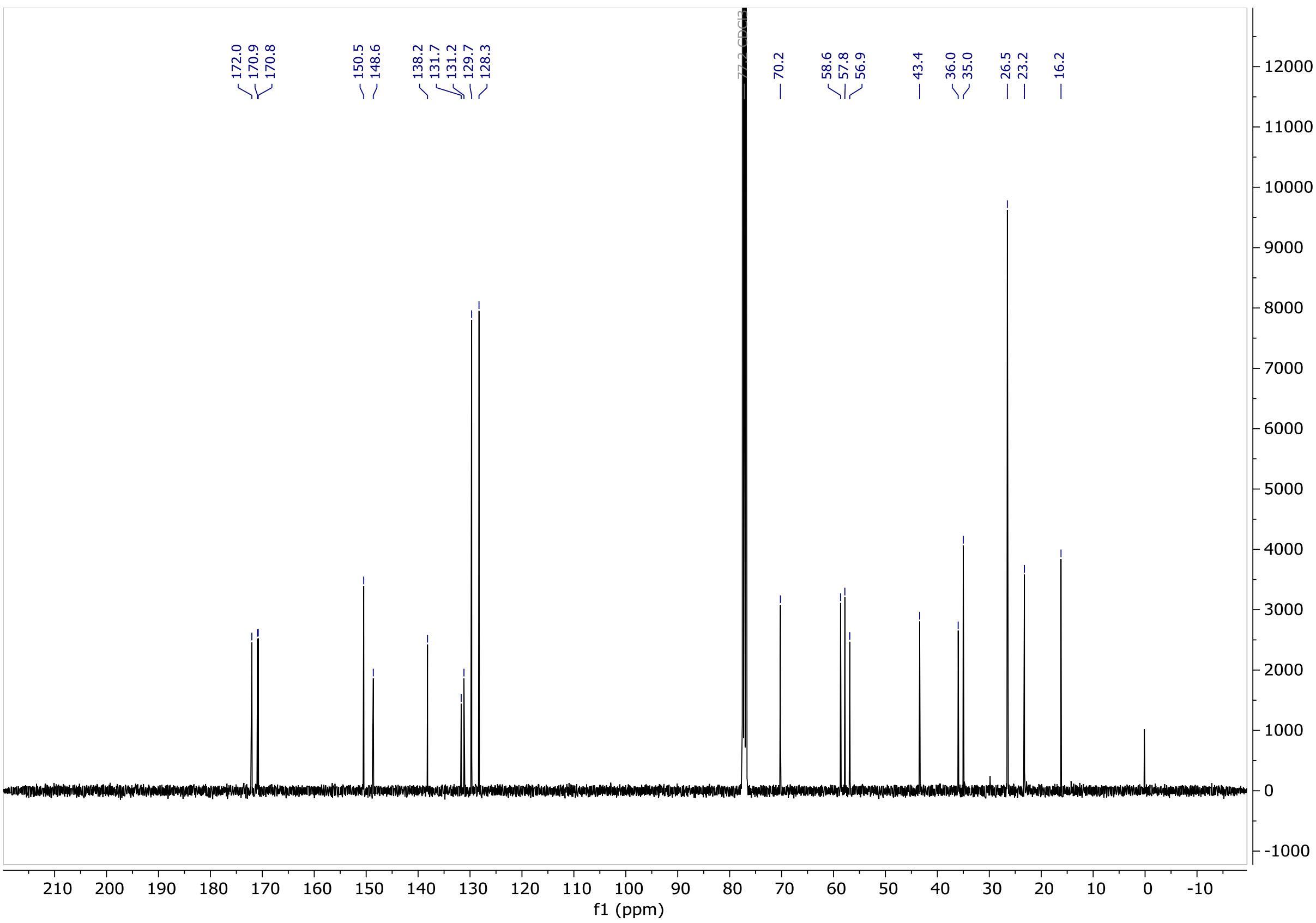

^{1}H NMR spectrum of 2-methylbut-3-en-2-yl ether cleaved FC-NAc

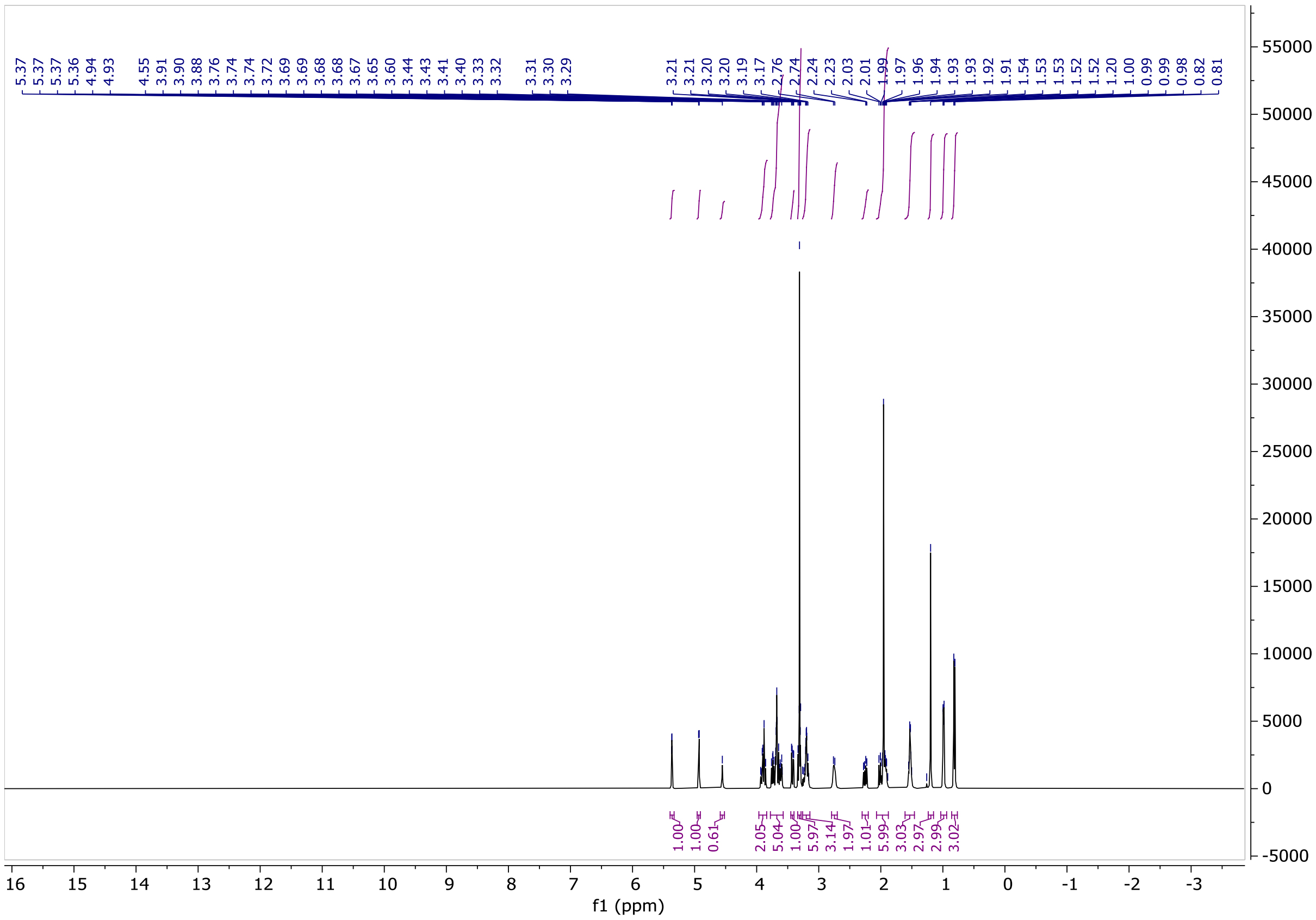

^{13}C NMR spectrum of 2-methylbut-3-en-2-yl ether cleaved FC-NAc

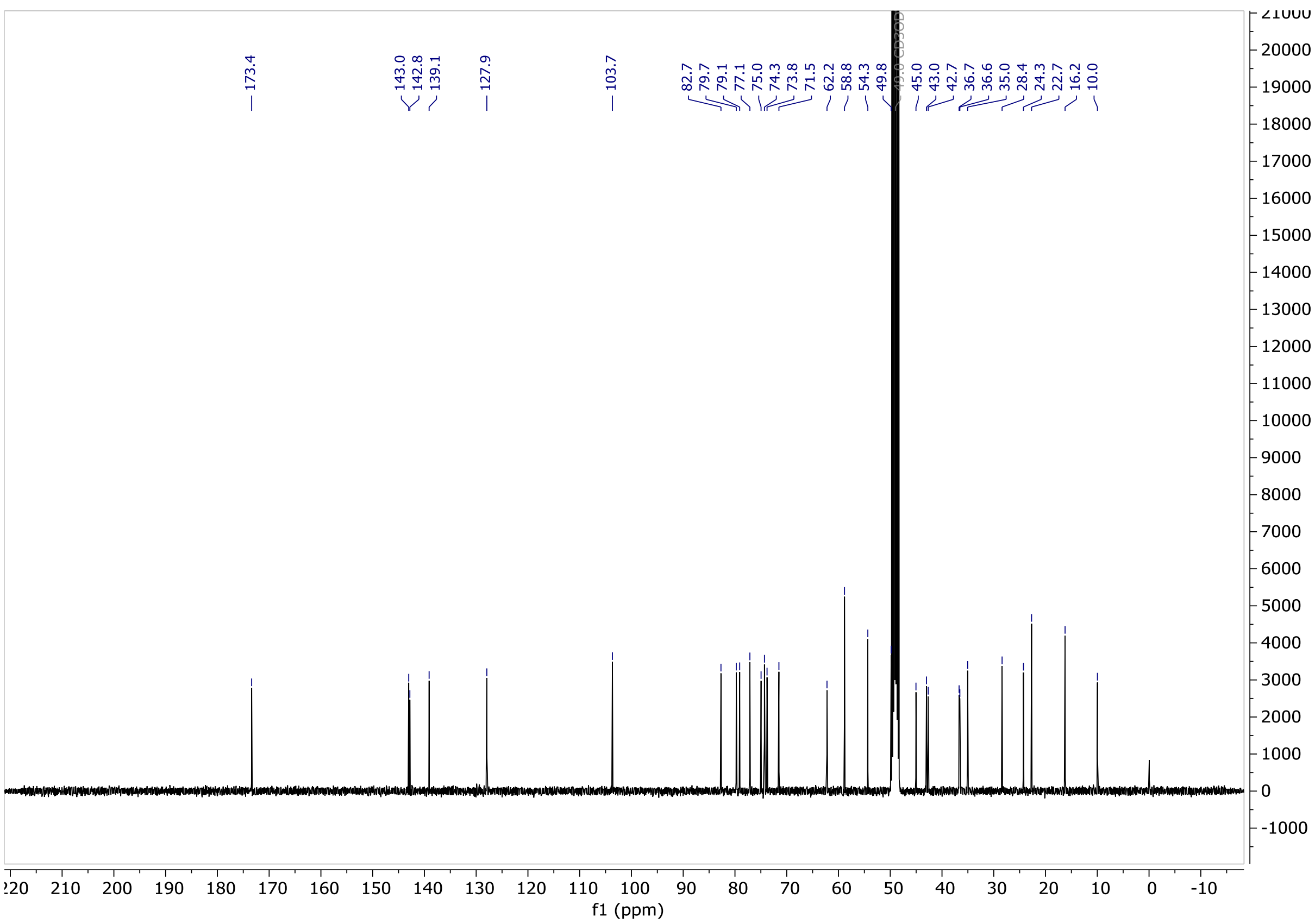

^{1}H NMR spectrum of **CV1p**

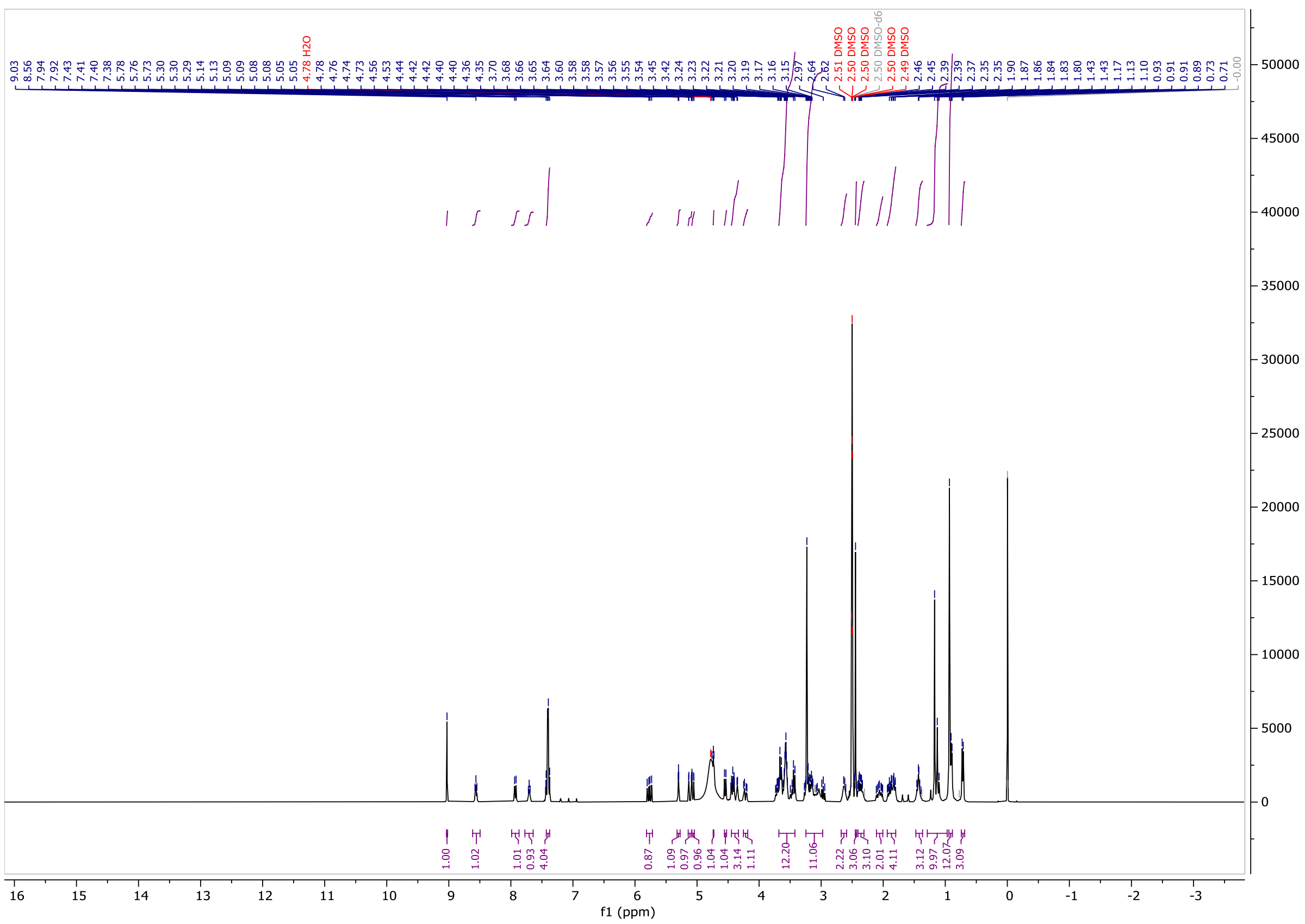

^{13}C NMR spectrum of **CV1p**

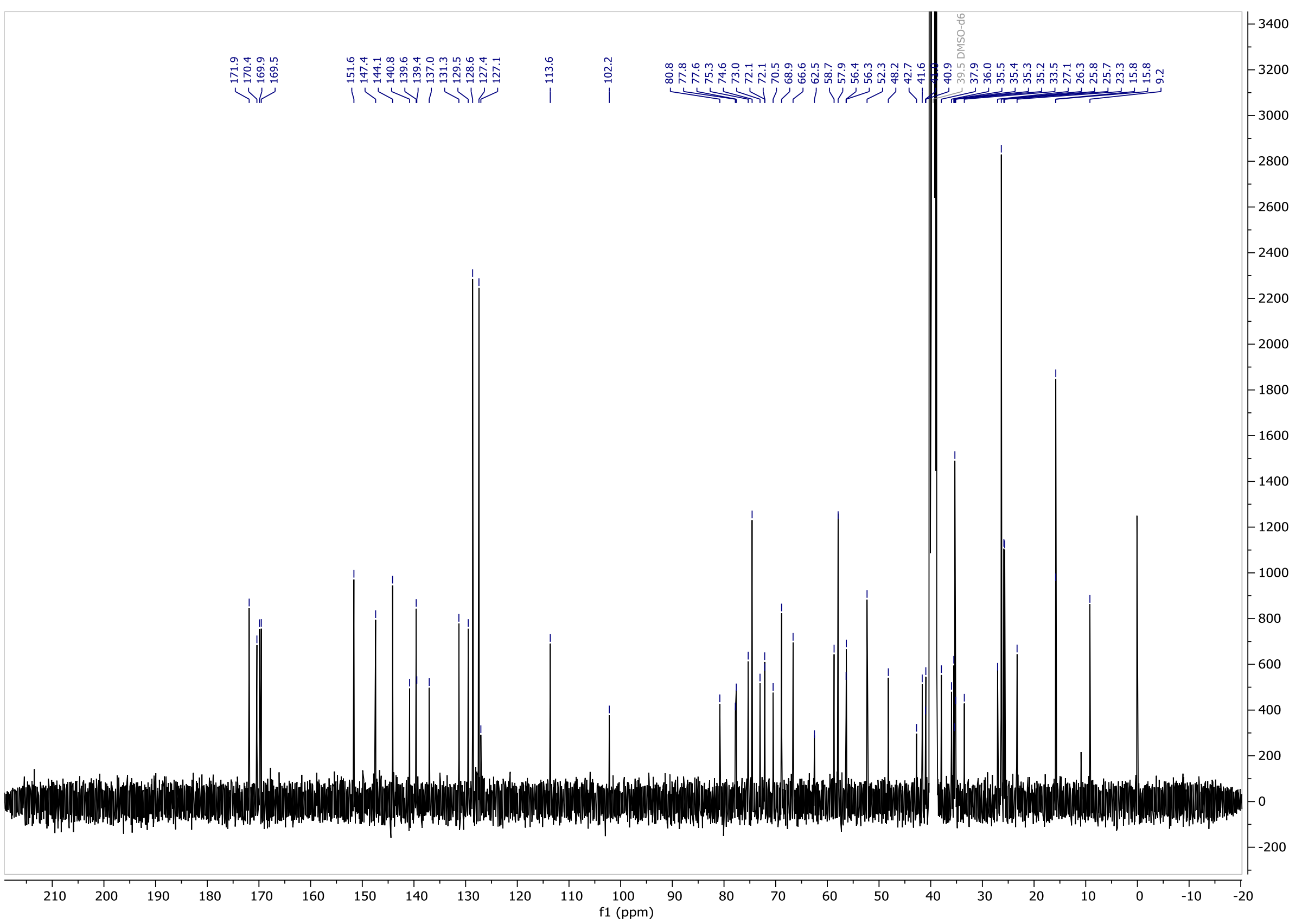

^{1}H NMR spectrum of **CV2p**

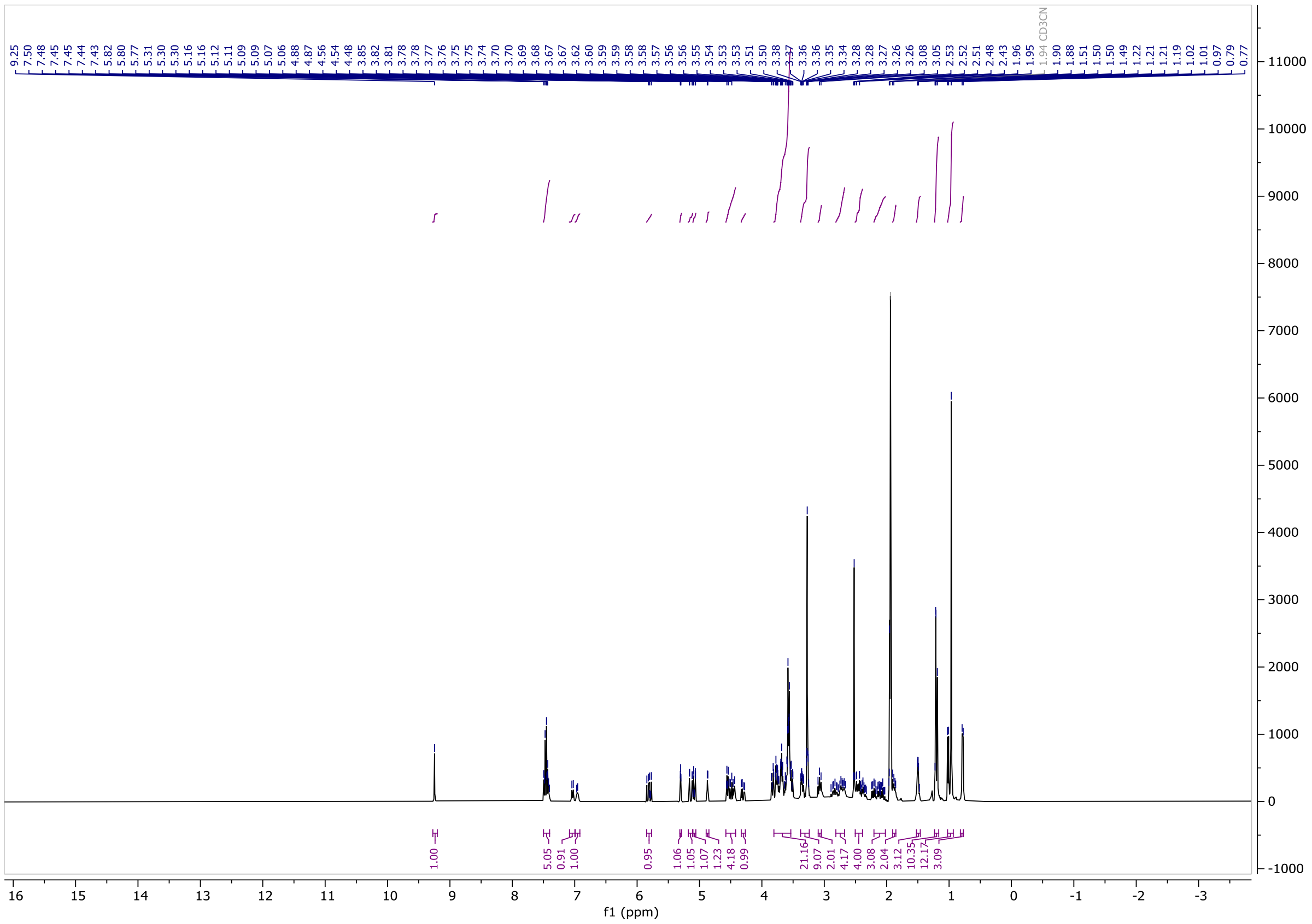

^{13}C NMR spectrum of **CV2p**

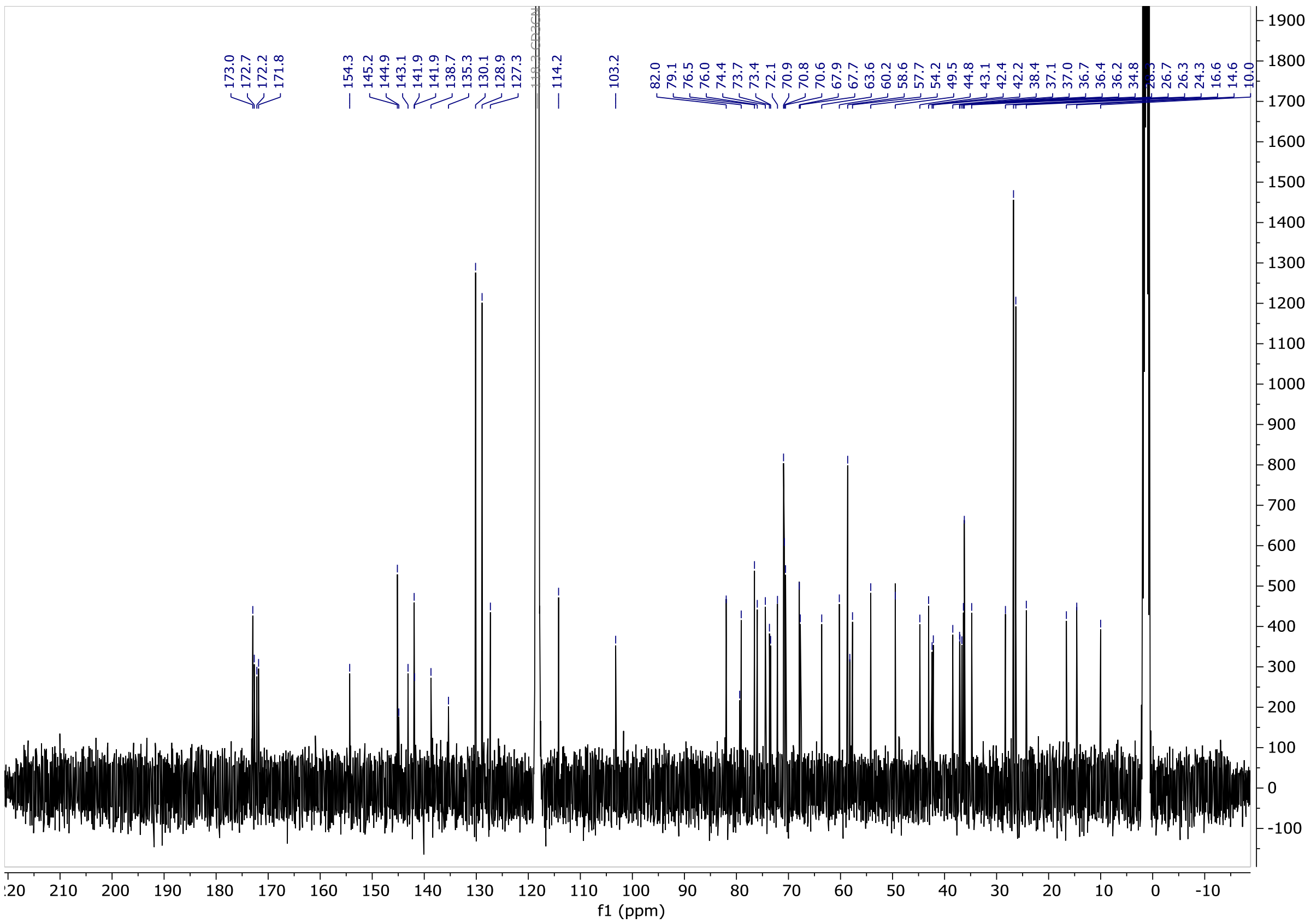

^{1}H NMR spectrum of **CV3p**

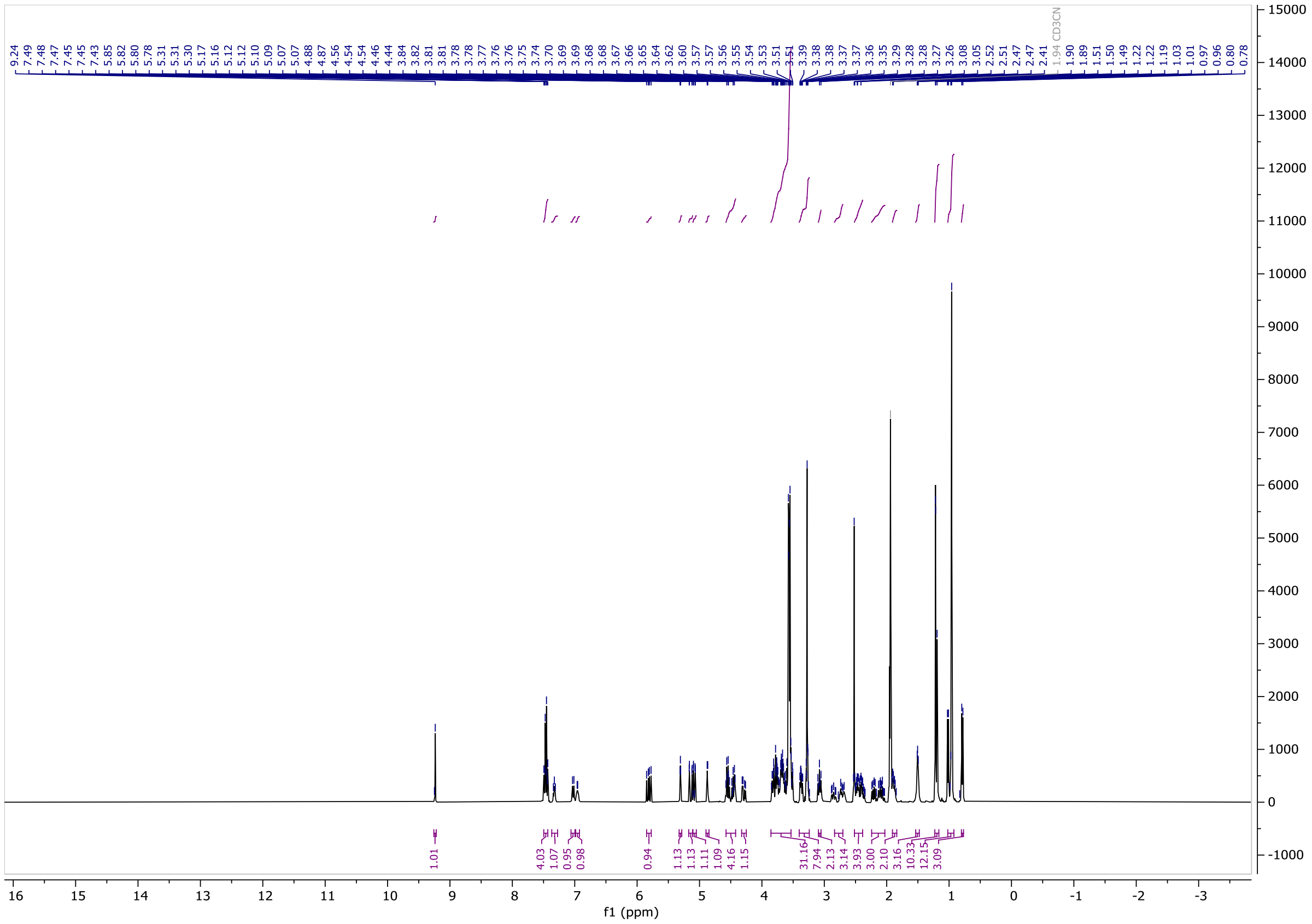

^{13}C NMR spectrum of **CV3p**

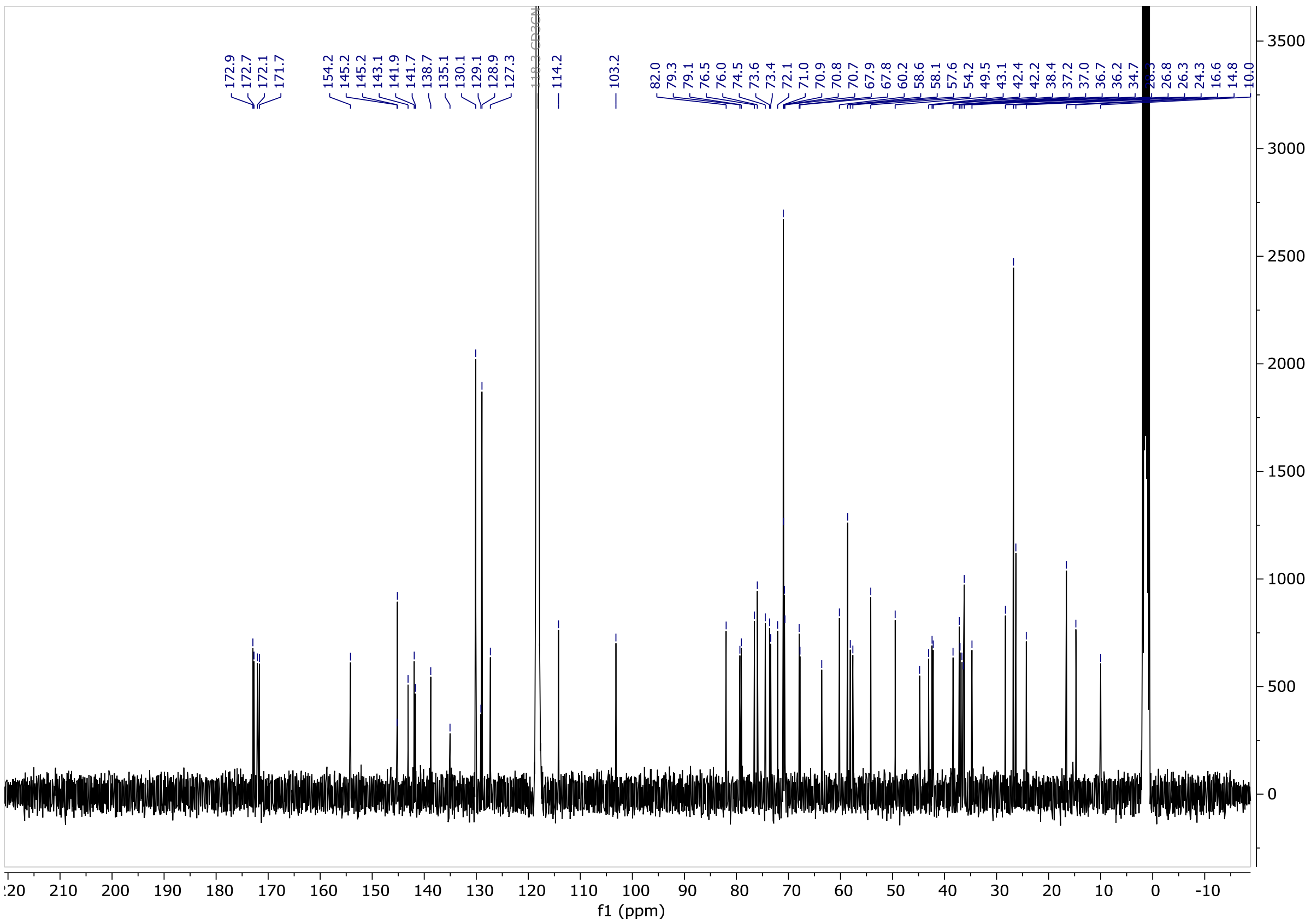

^{1}H NMR spectrum of **CV4p**

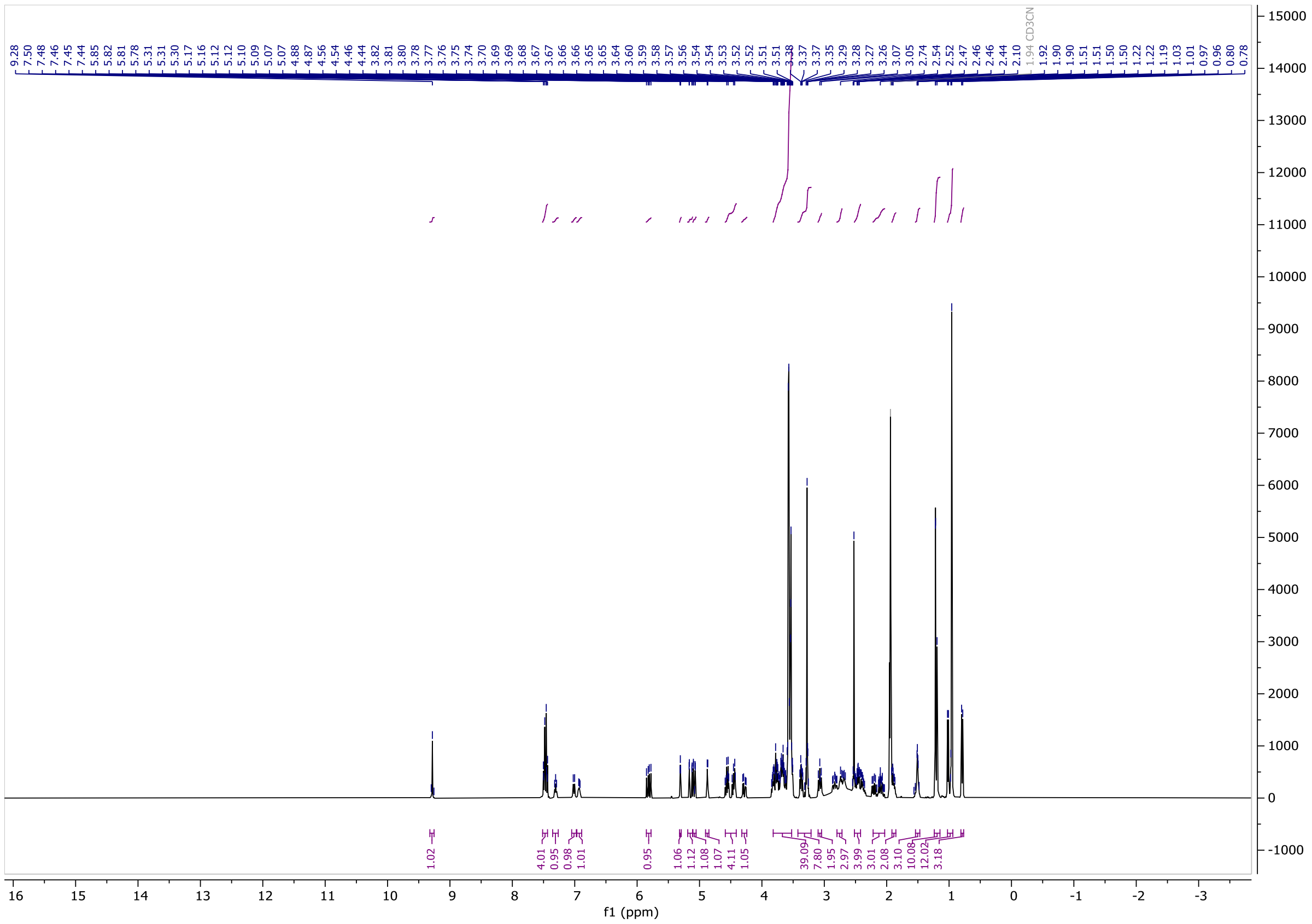

^{13}C NMR spectrum of **CV4p**

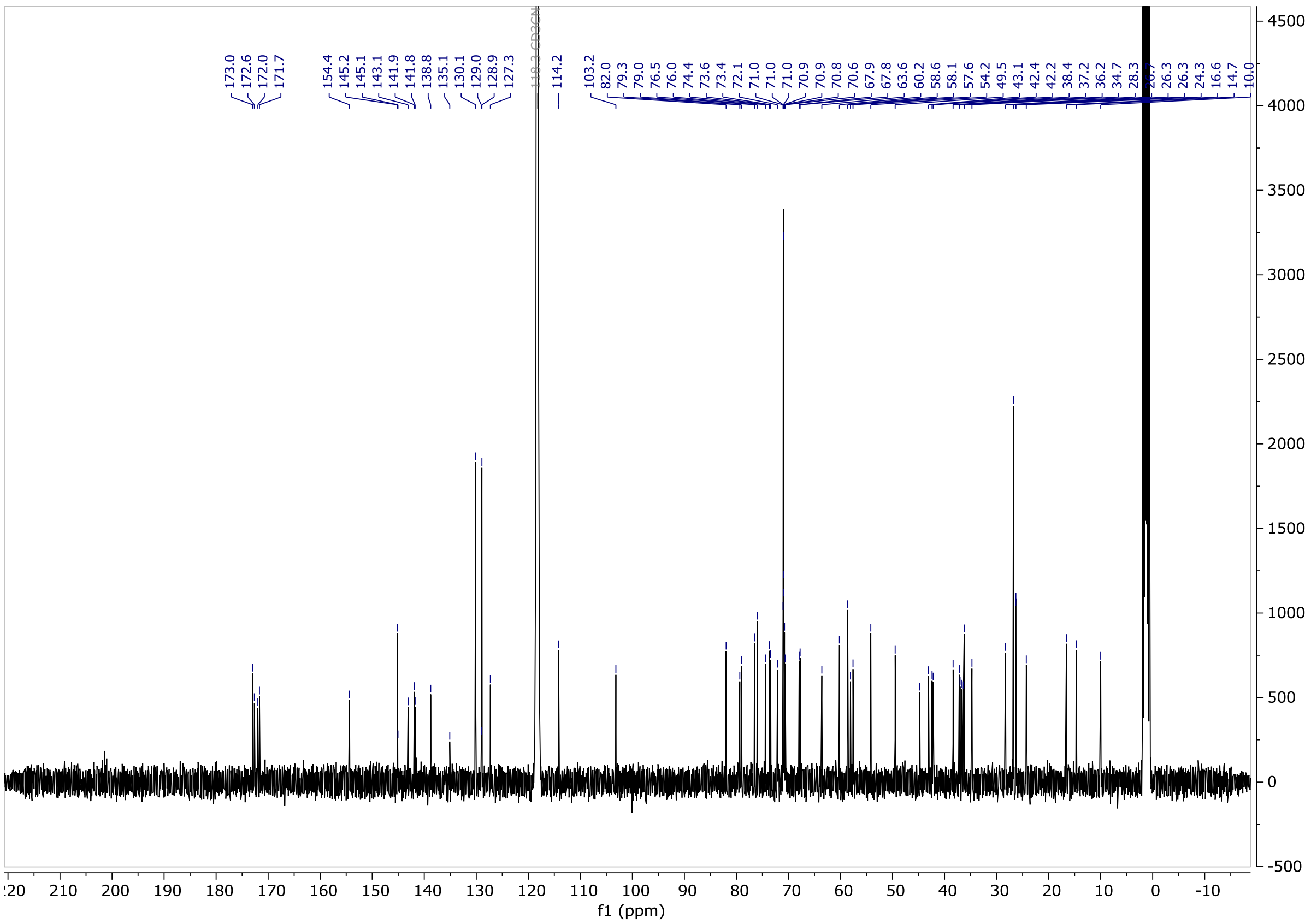

^{1}H NMR spectrum of **CV0a**

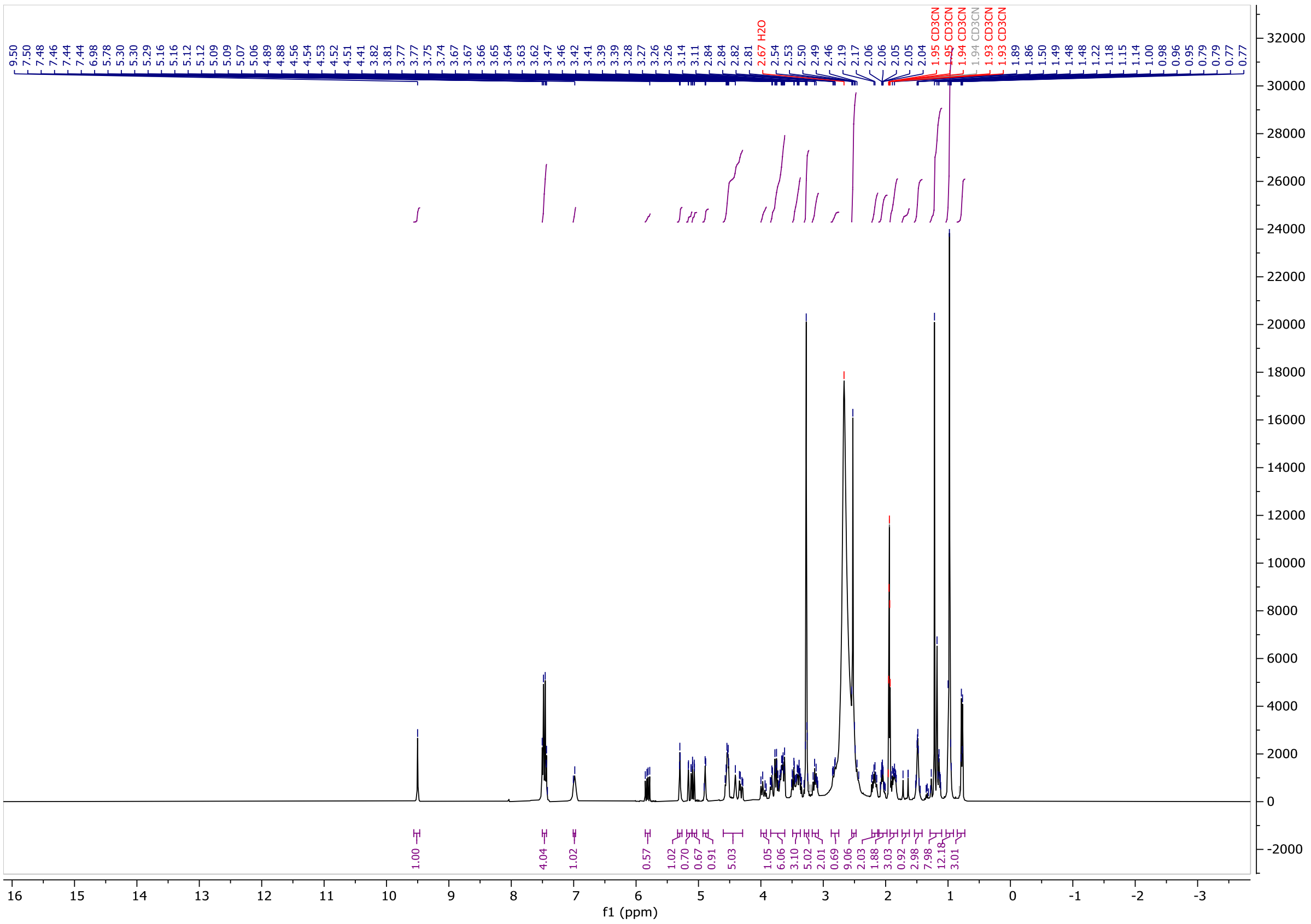

^{13}C NMR spectrum of **CV0a**

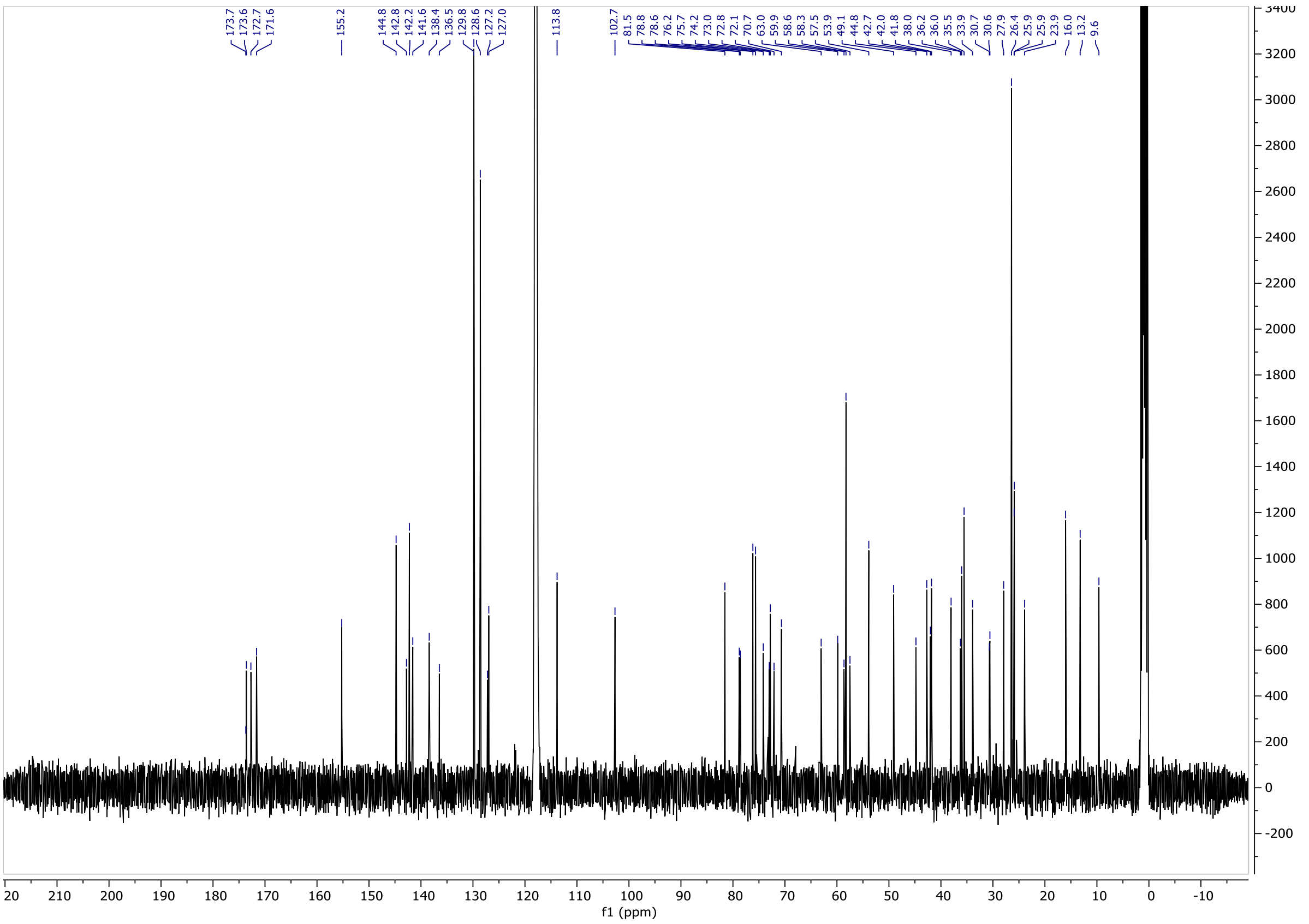

^{1}H NMR spectrum of **CV1a**

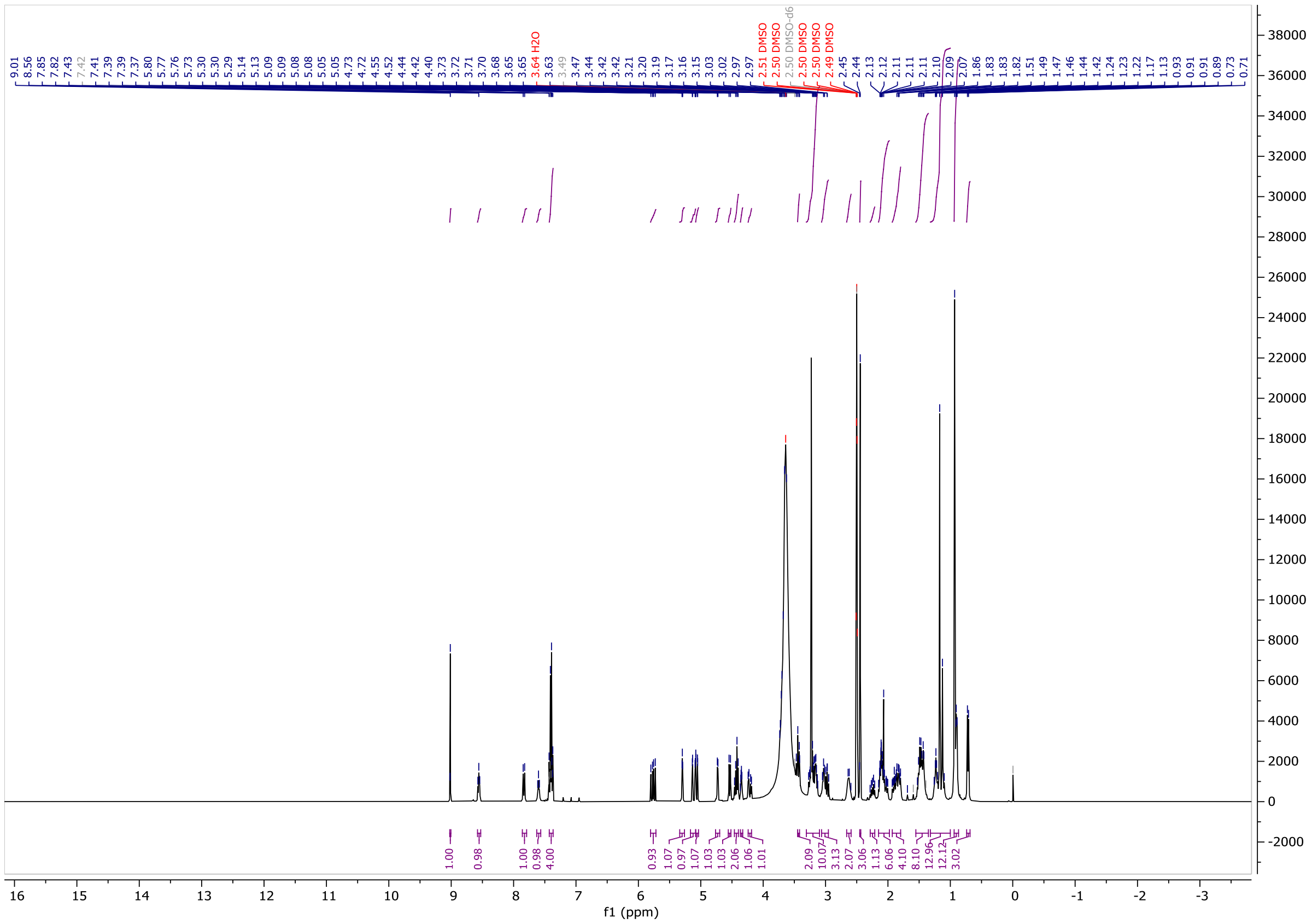

^{13}C NMR spectrum of **CV1a**

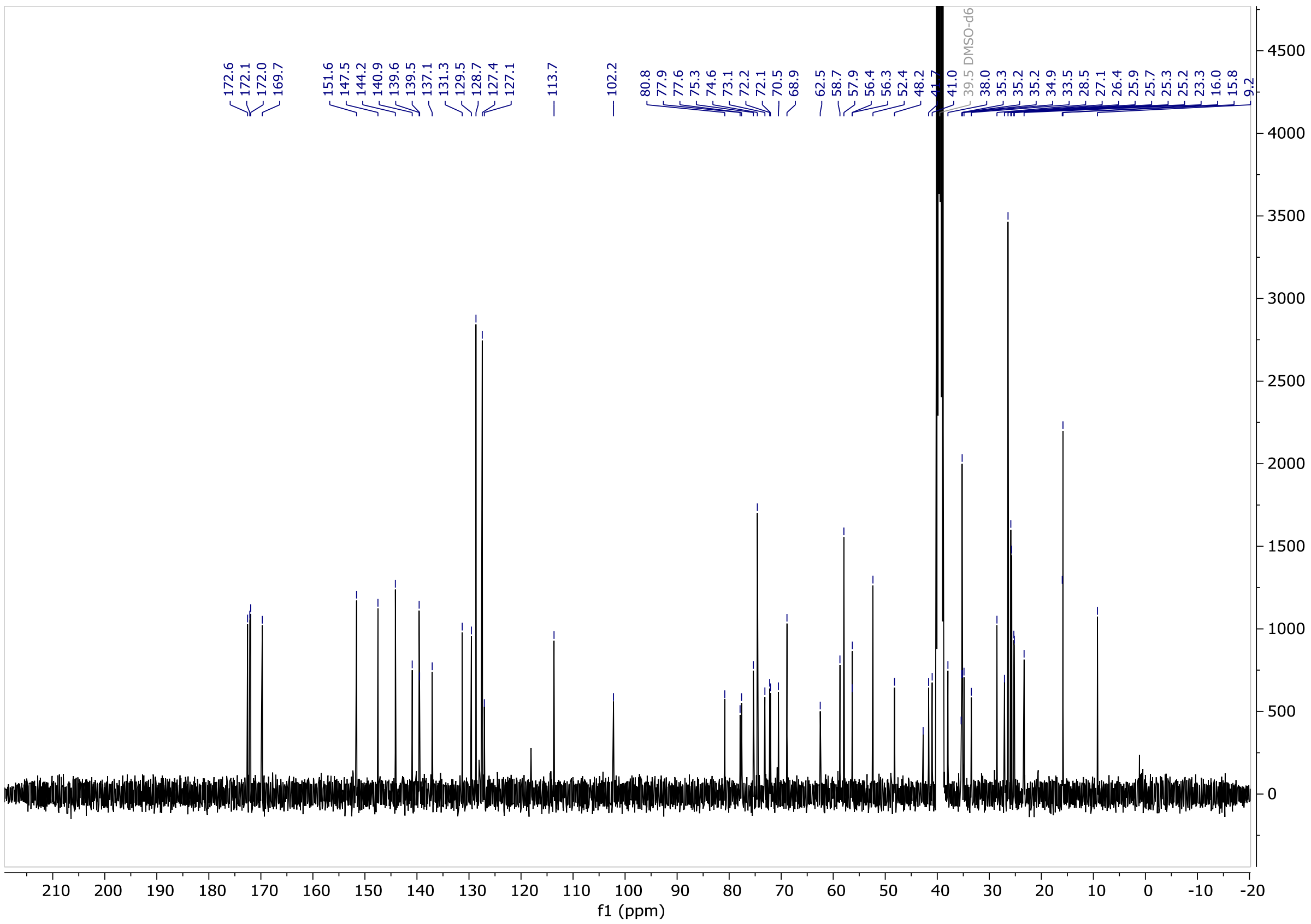

^{1}H NMR spectrum of **CV2a**

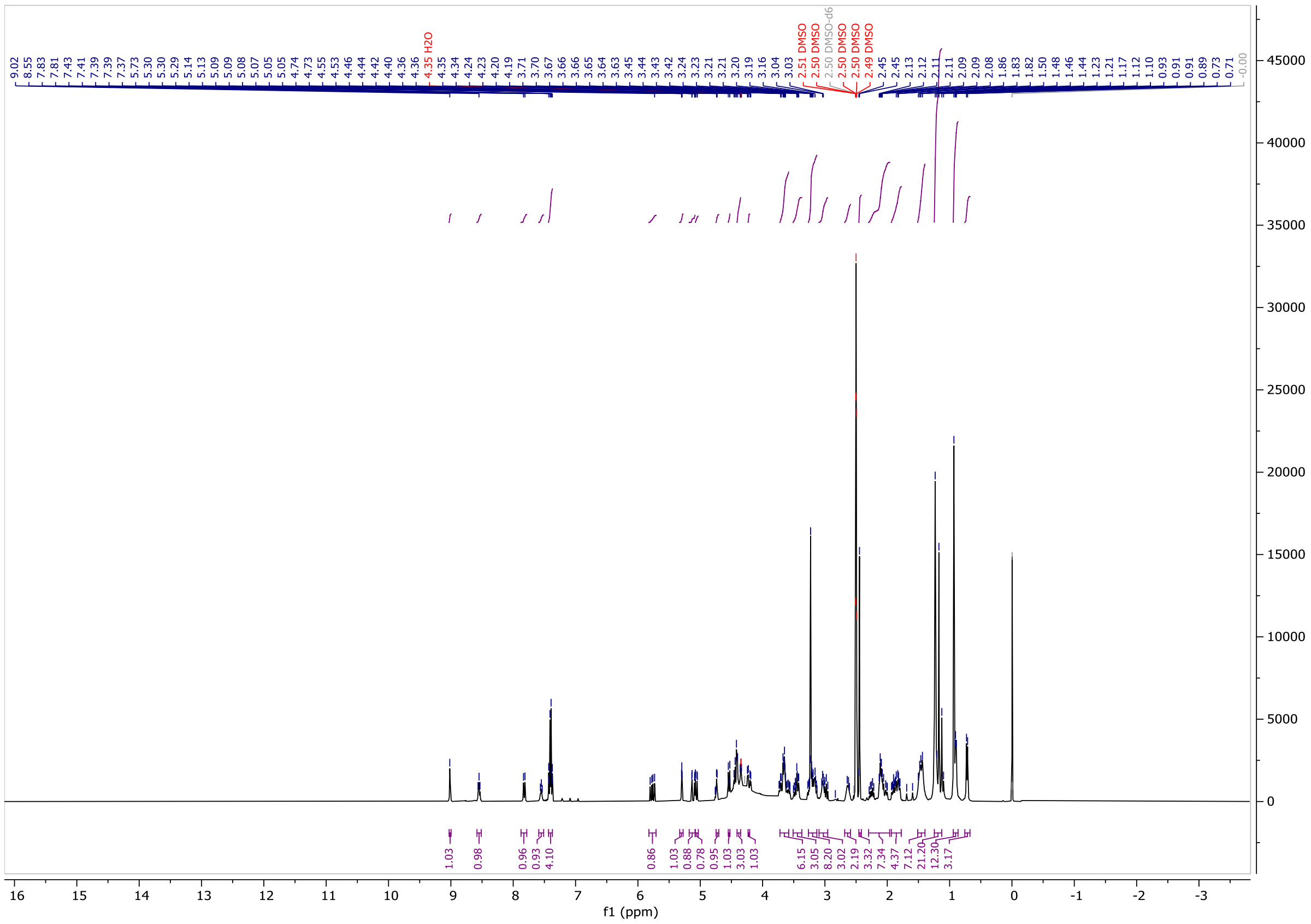

^{13}C NMR spectrum of **CV2a**

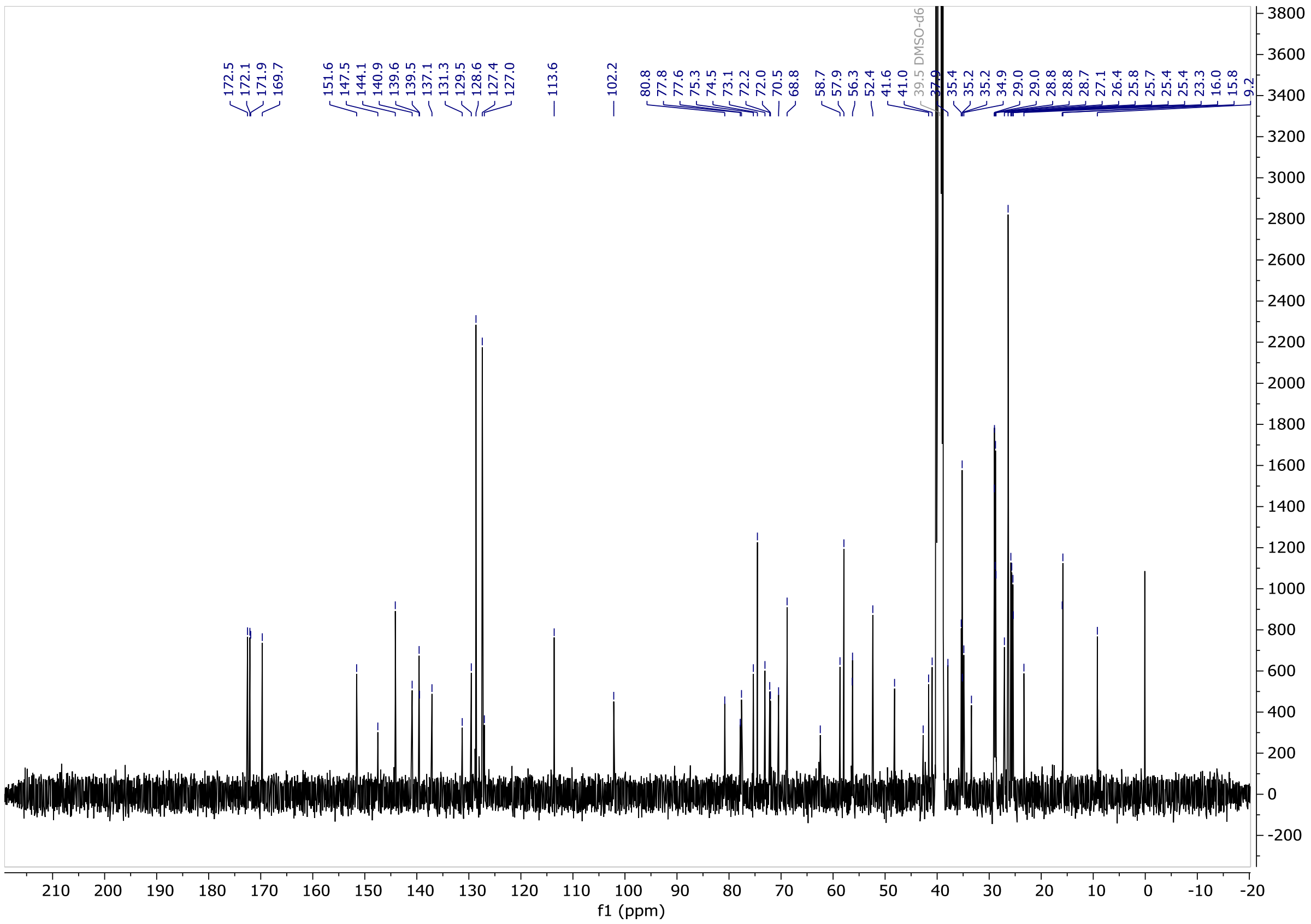

^{1}H NMR spectrum of **CV3a**

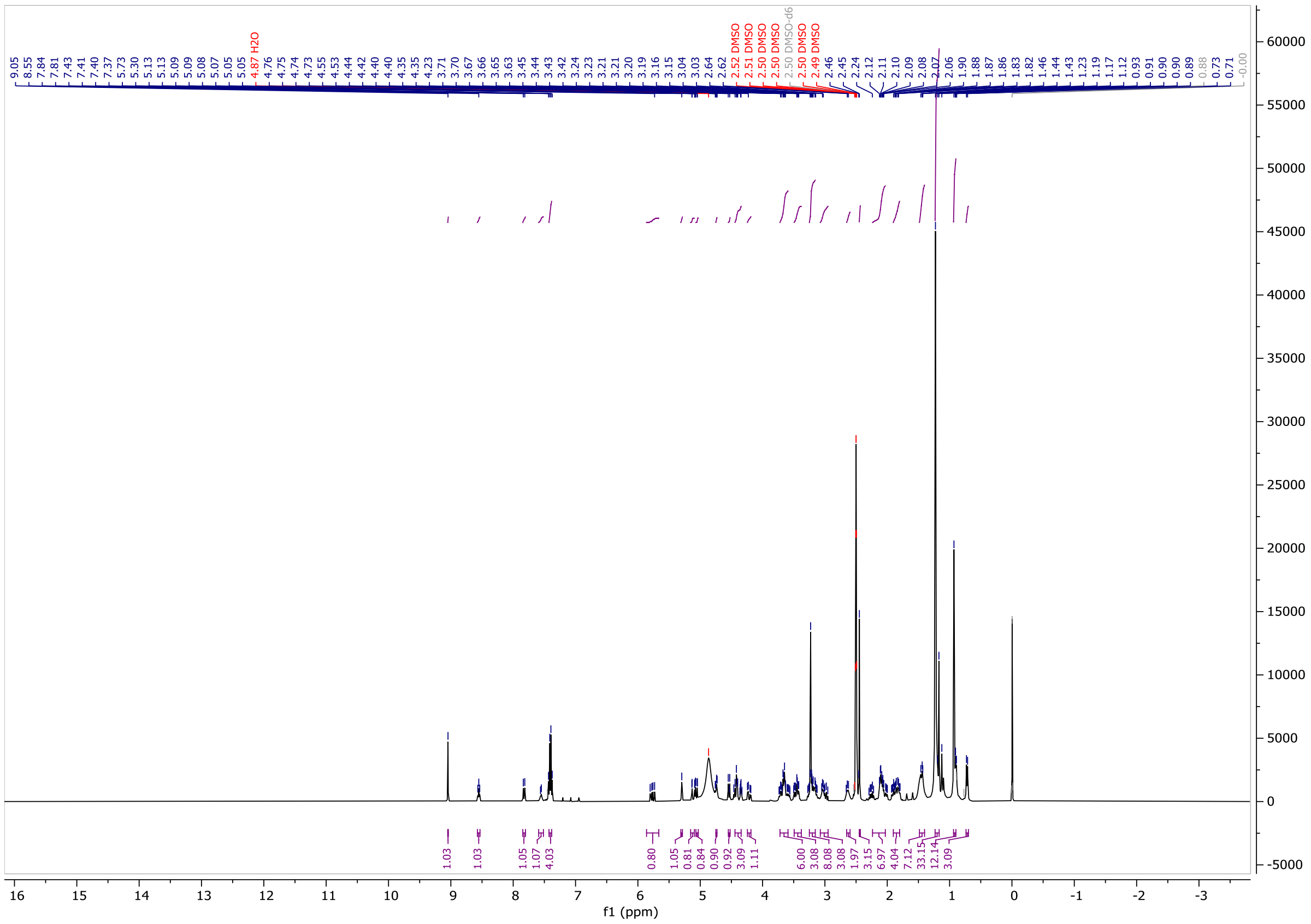

^{13}C NMR spectrum of **CV3a**

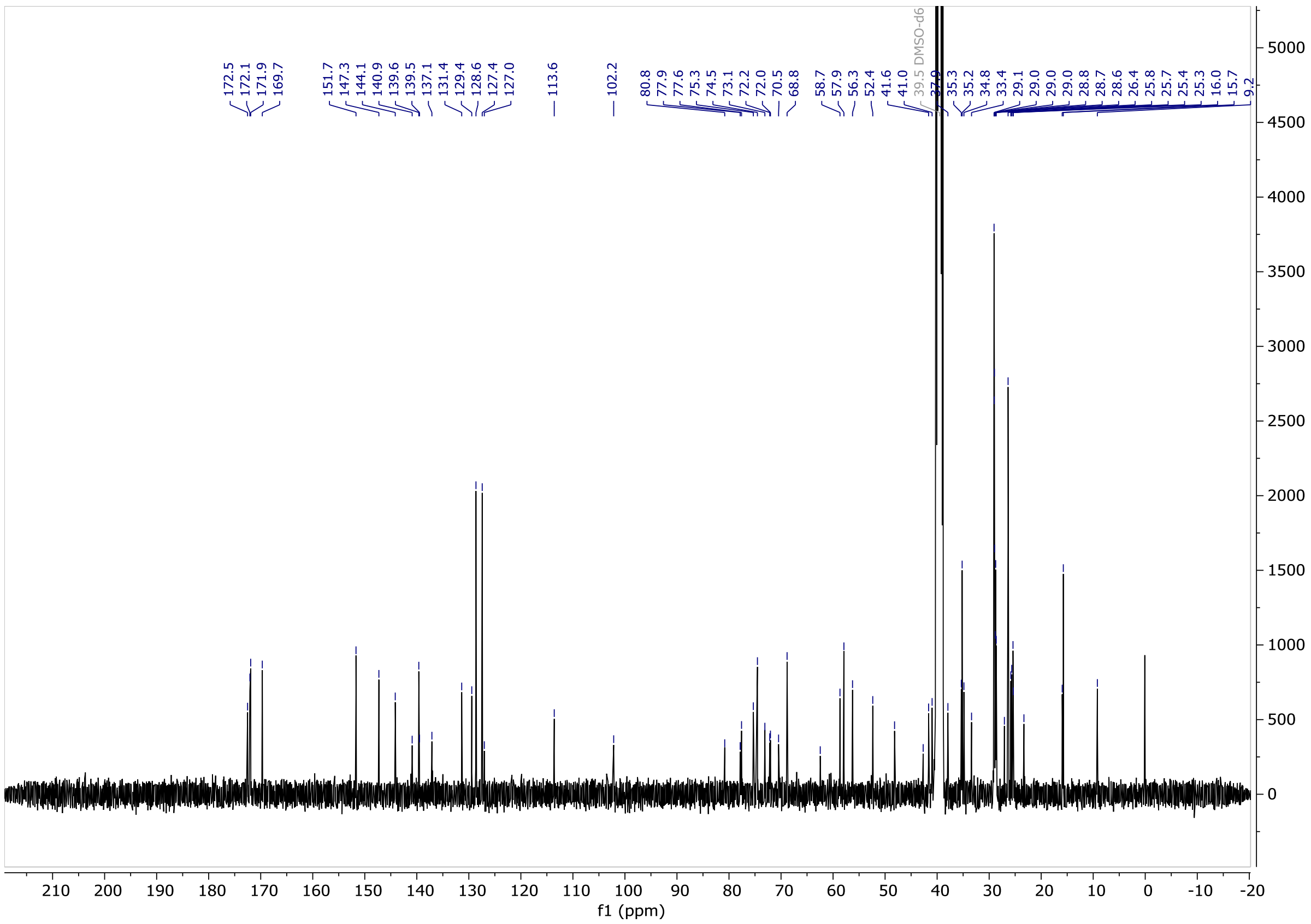

^{1}H NMR spectrum of **(*S*-OH)-CV2a**

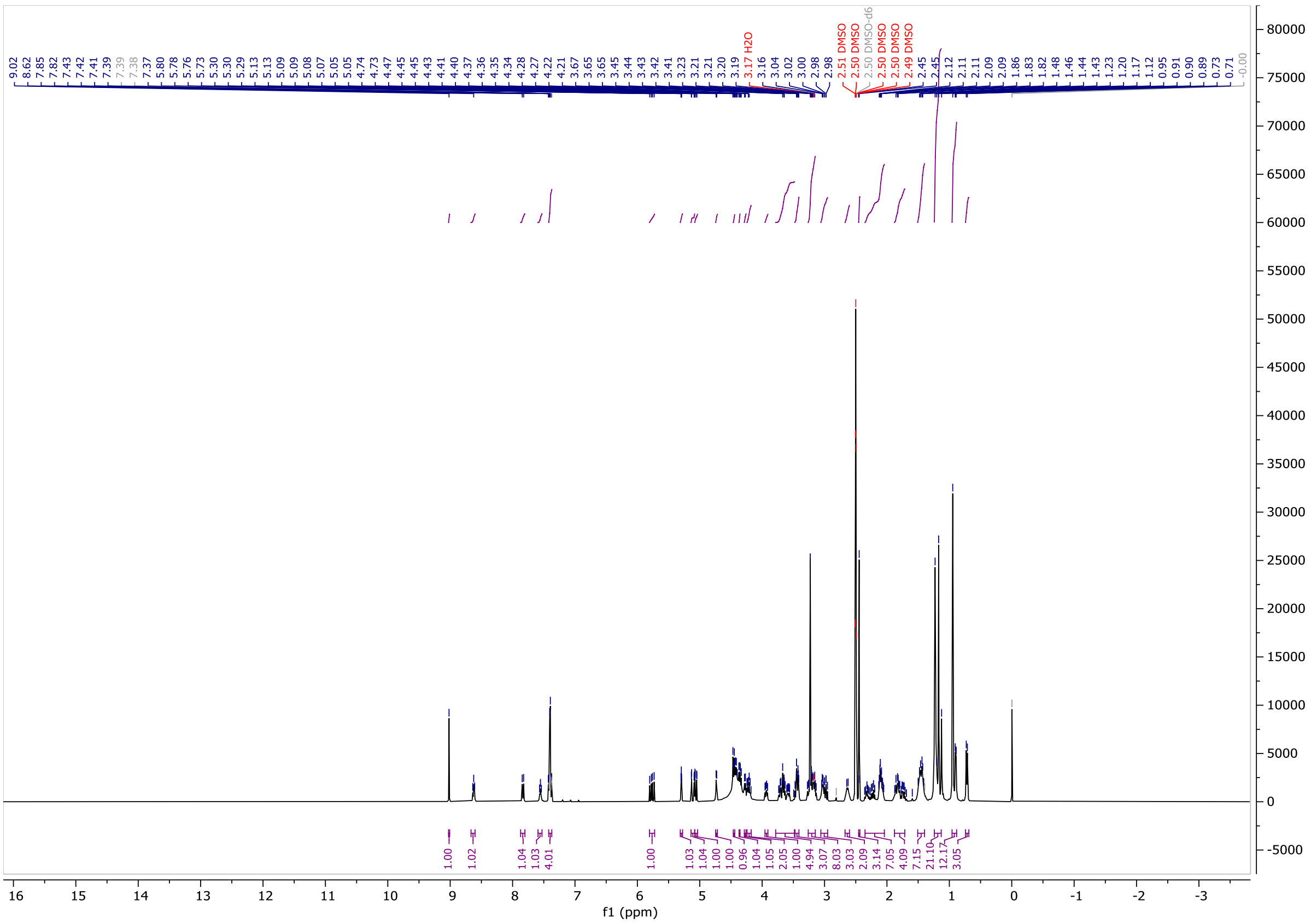

^{13}C NMR spectrum of **(*S*-OH)-CV2a**

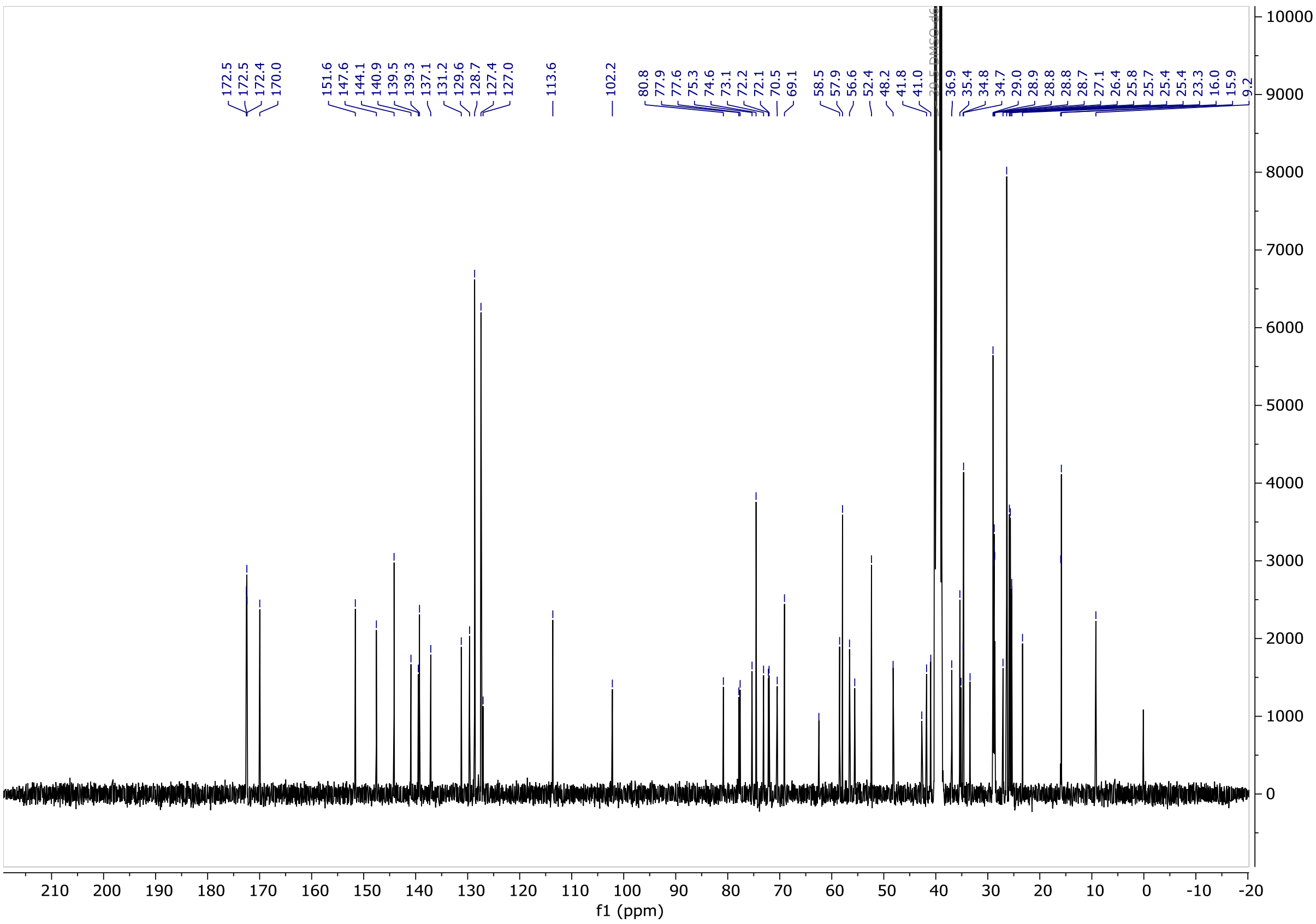

HPLC chromatograms

HPLC chromatogram of **FC-NH$_2$**

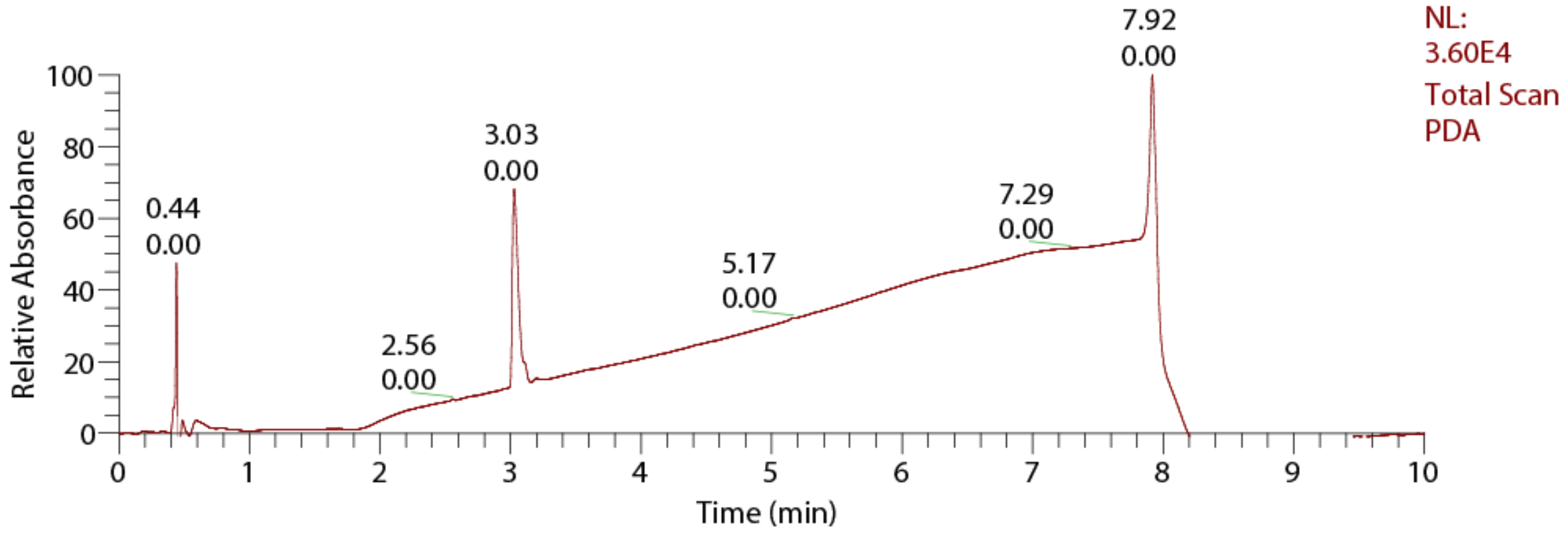

HPLC chromatogram of **FC-NAc**

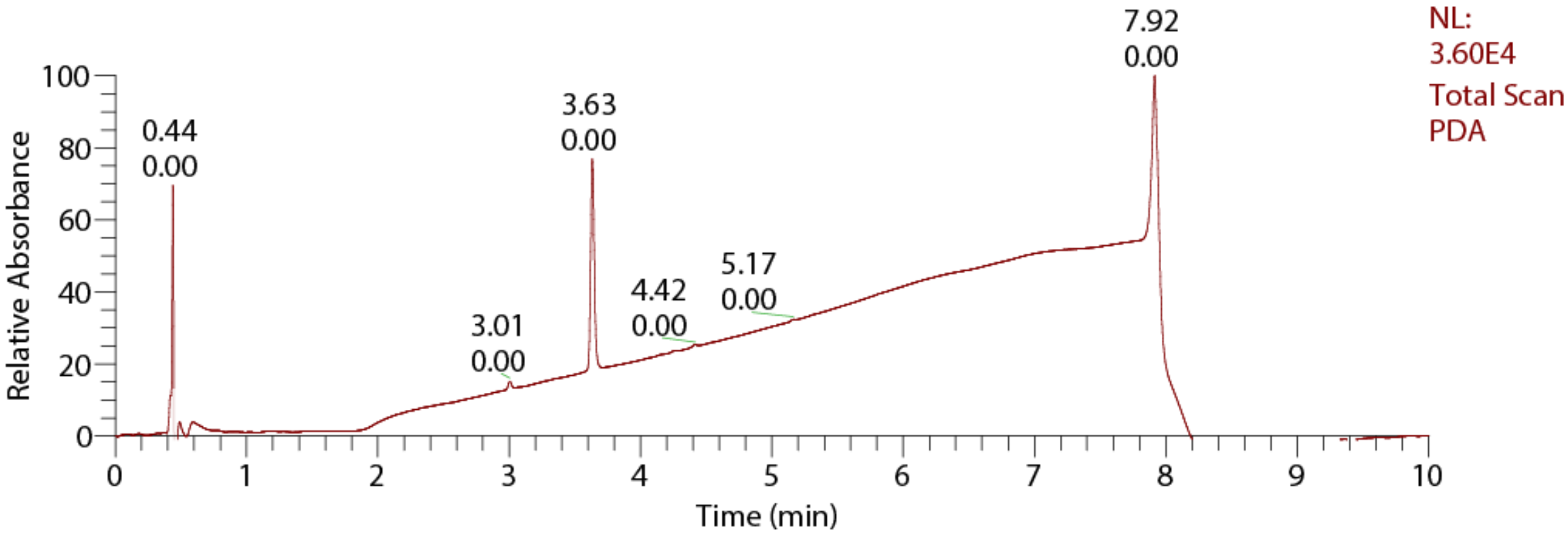

HPLC chromatogram of **VH032**

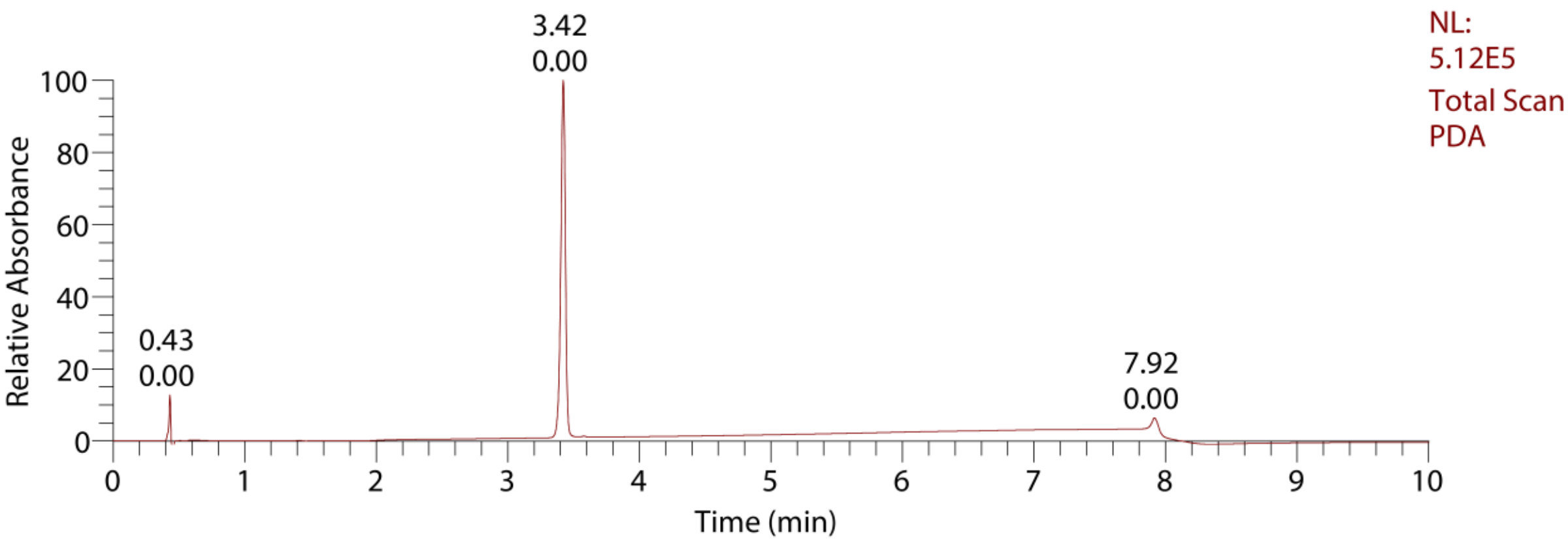

HPLC chromatogram of **2-methylbut-3-en-2-yl ether cleaved FC-NAc**

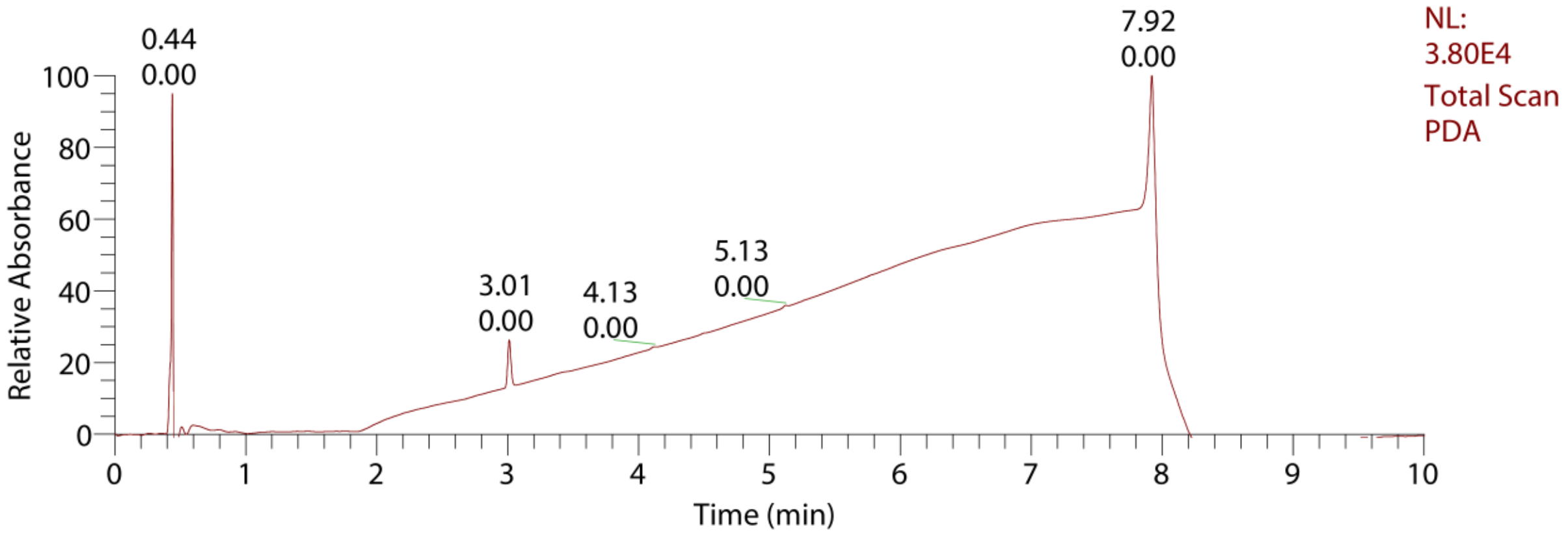

HPLC chromatogram of **CV1p**

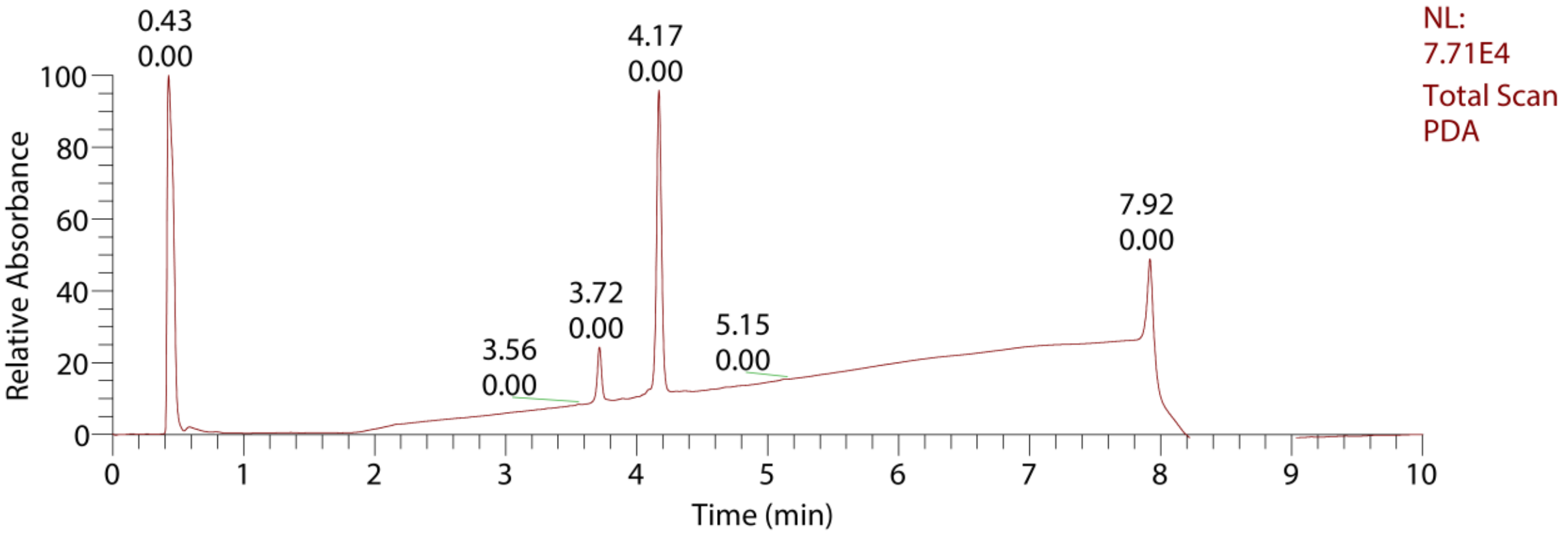

HPLC chromatogram of **CV2p**

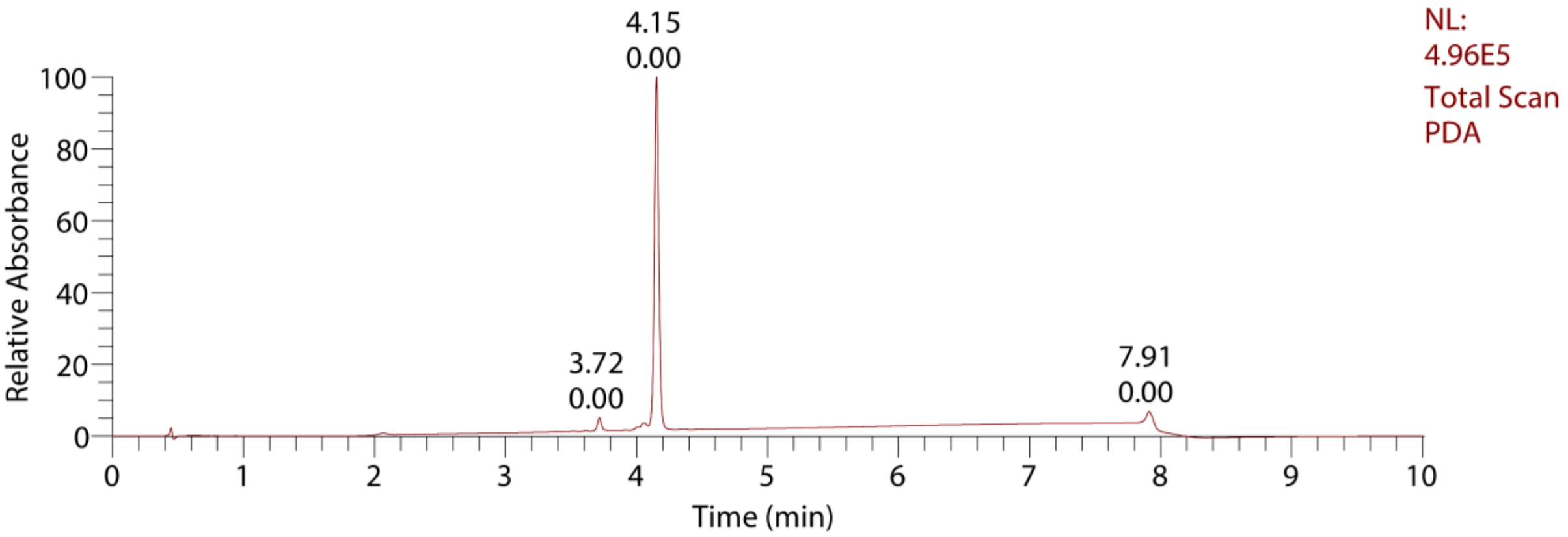

HPLC chromatogram of **CV3p**

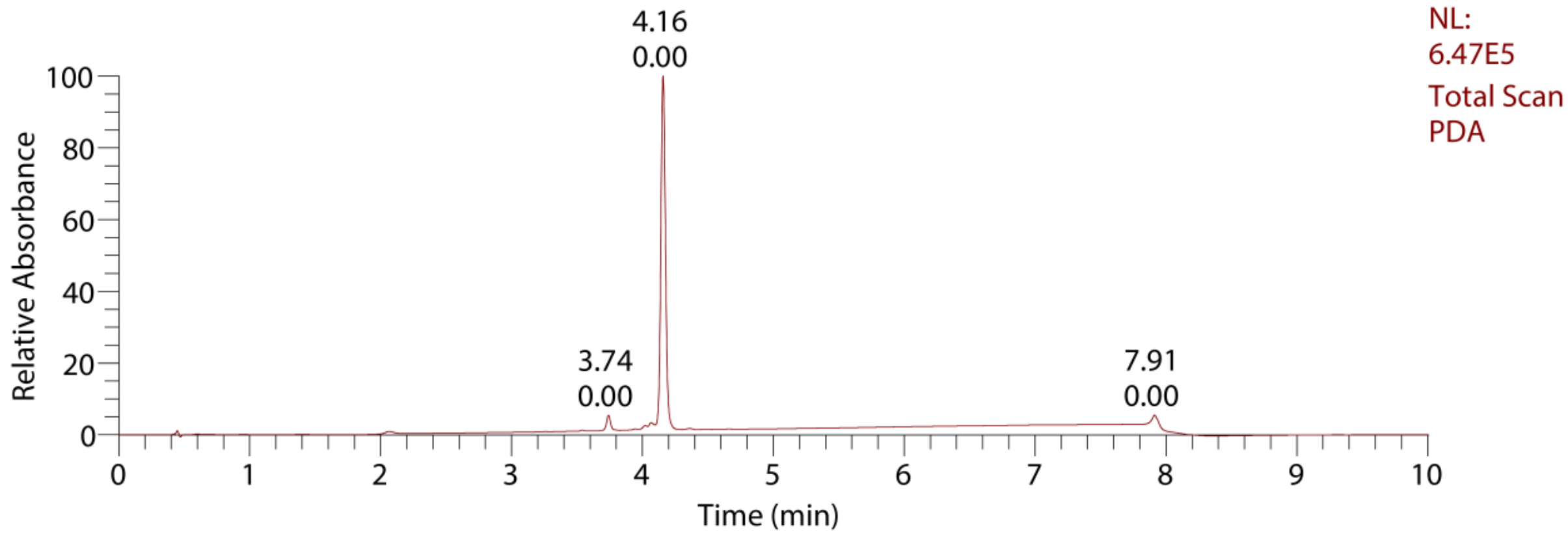

HPLC chromatogram of **CV4p**

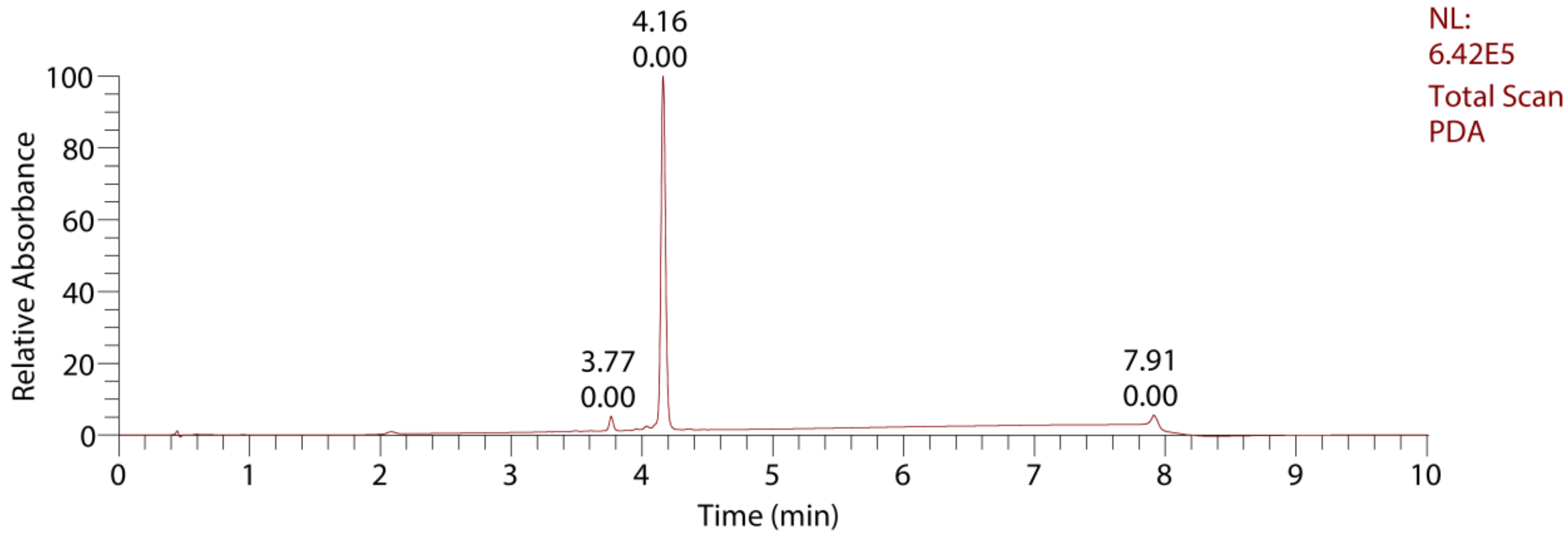

HPLC chromatogram of **CV0a**

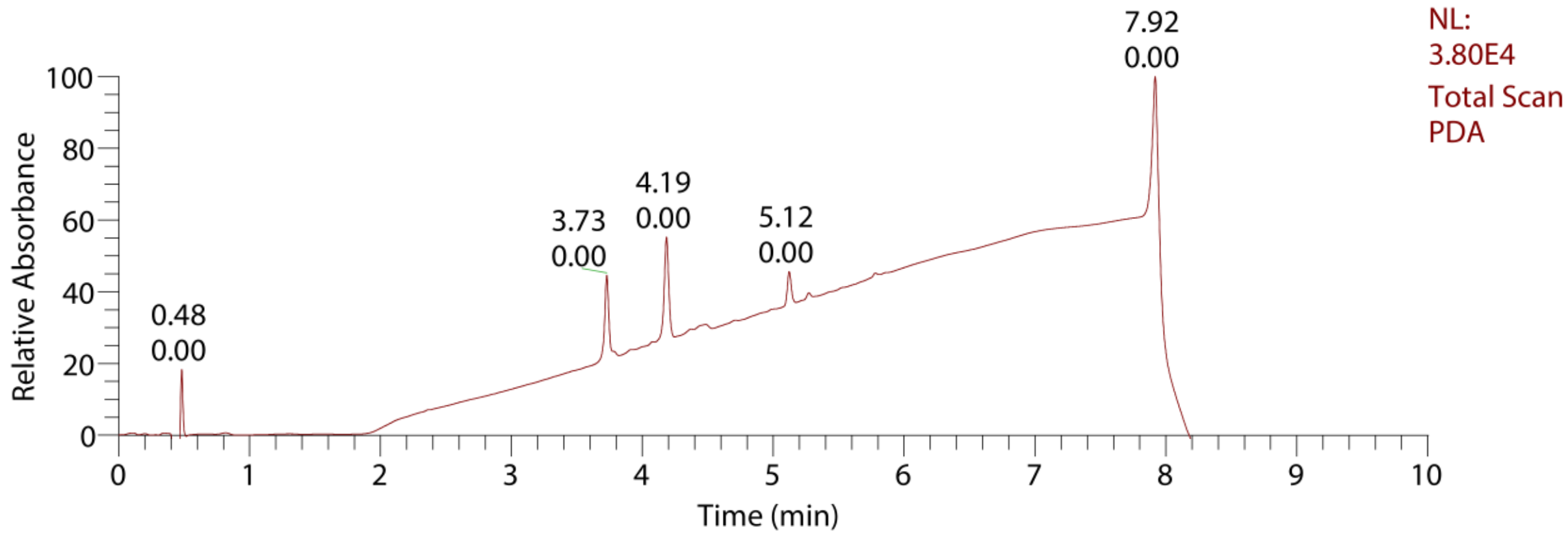

HPLC chromatogram of **CV1a**

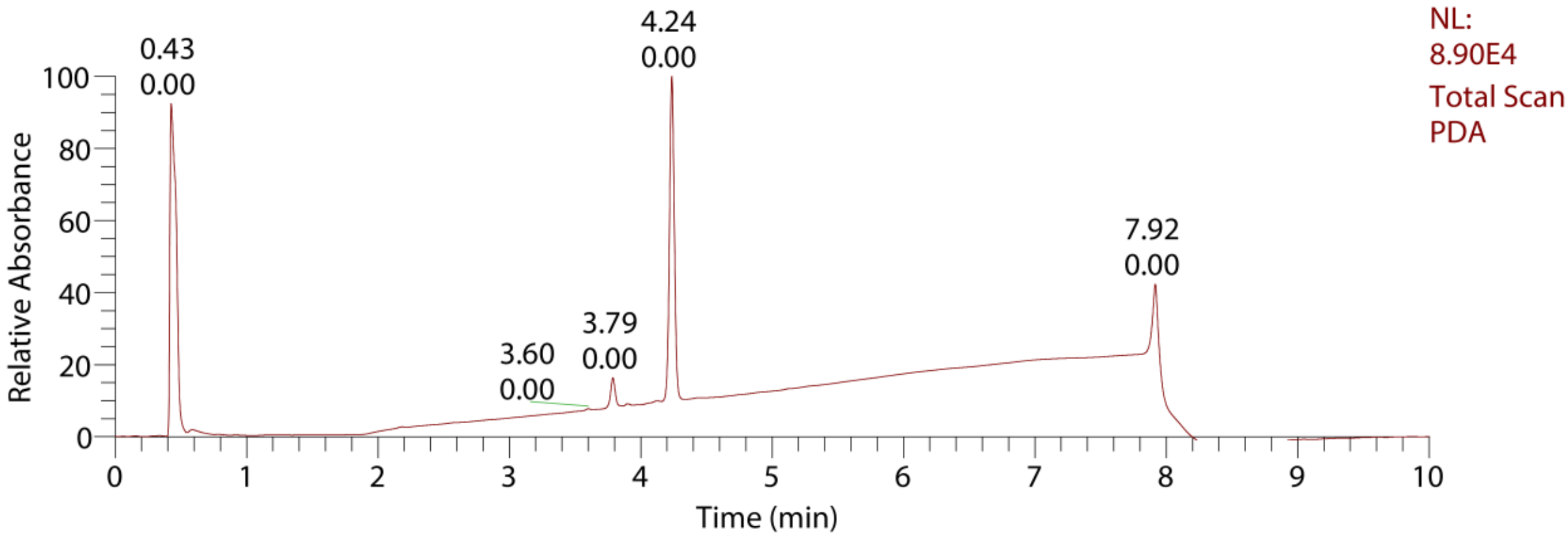

HPLC chromatogram of **CV2a**

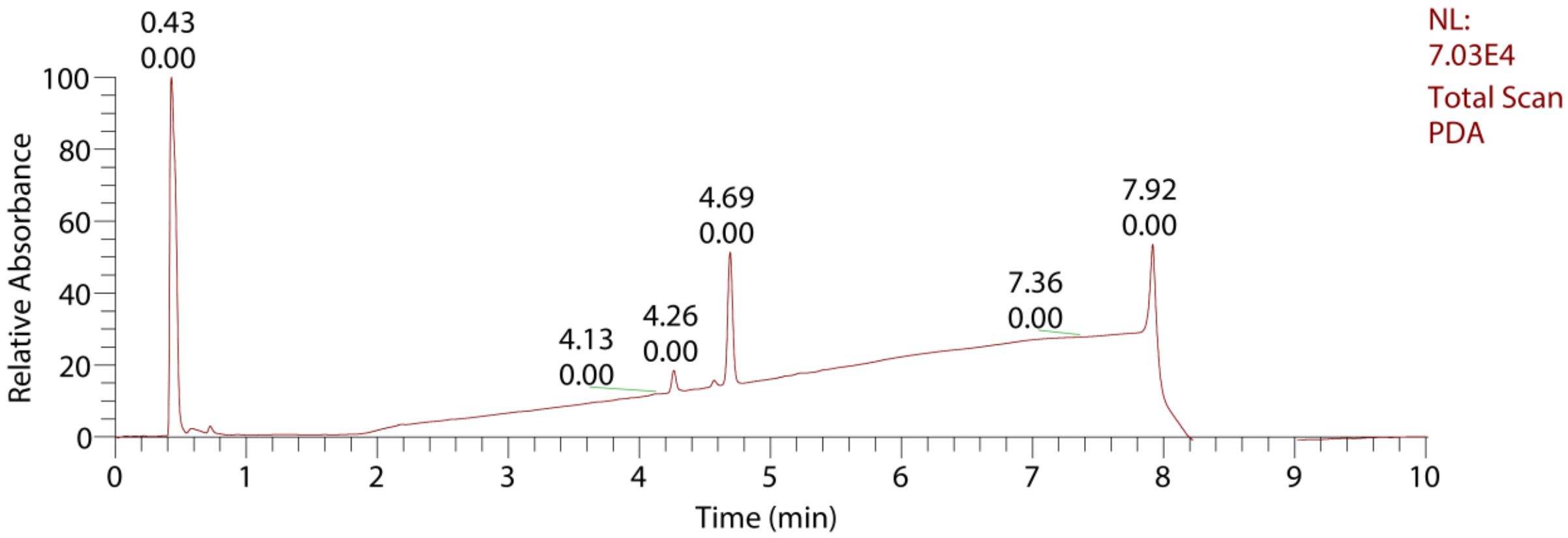

HPLC chromatogram of **CV3a**

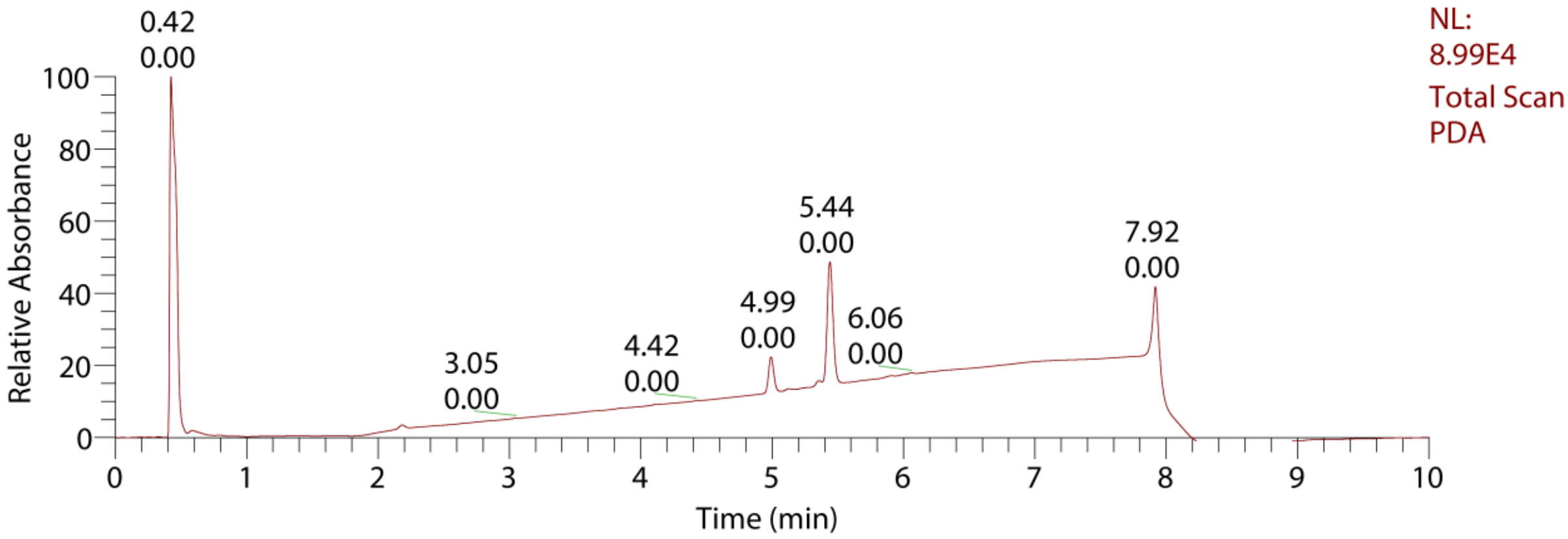

HPLC chromatogram of **(*S*-OH)-CV2a**

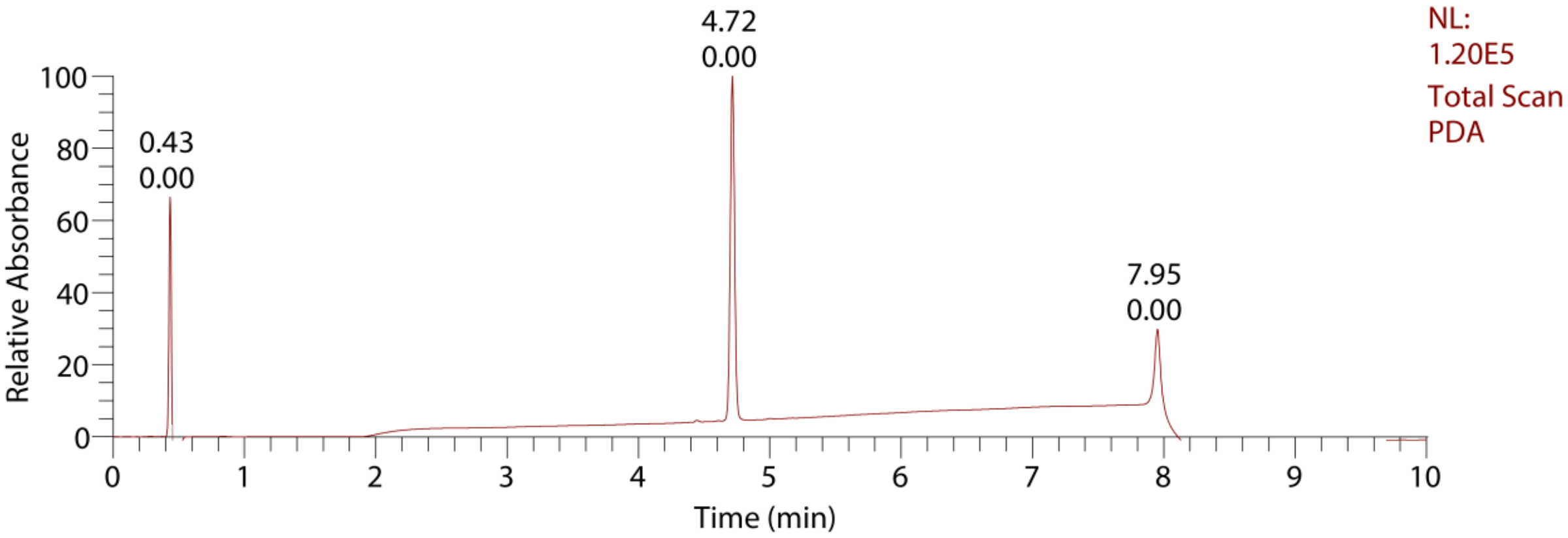

4. Supplementary References

1. Somsen, B. A. *et al.* Molecular basis and dual ligand regulation of tetrameric estrogen receptor α/14-3-3ζ protein complex. *J. Biol. Chem.* **299**, 104855 (2023).

2. Altschul, S. F., Gish, W., Miller, W., Myers, E. W. & Lipman, D. J. Basic local alignment search tool. *J. Mol. Biol.* **215**, 403–410 (1990).

3. Andrei, S. A. *et al.* Rationally Designed Semisynthetic Natural Product Analogues for Stabilization of 14-3-3 Protein–Protein Interactions. *Angew. Chem. Int. Ed.* **57**, 13470–13474 (2018).

4. Ohkanda, J. *et al.* Structural Effects of Fusicoccin upon Upregulation of 14-3-3-Phospholigand Interaction and Cytotoxic Activity. *Chem. – Eur. J.* **24**, 16066–16071 (2018).

5. Galdeano, C. *et al.* Structure-guided design and optimization of small molecules targeting the protein-protein interaction between the von Hippel-Lindau (VHL) E3 ubiquitin ligase and the hypoxia inducible factor (HIF) alpha subunit with in vitro nanomolar affinities. *J. Med. Chem.* **57**, 8657–8663 (2014).
