# Supplementary material for: Induced ubiquitination of the partially disordered estrogen receptor alpha via a 14-3-3 directed molecular glue-PROTAC
Source: Nat Commun. 2026 Jul 15;17:8696. doi: 10.1038/s41467-026-75333-w (PMC13490399; doi:10.1038/s41467-026-75333-w)
Supplement: Supplementary file 2 [file 41467_2026_75333_MOESM2_ESM.pdf]

Description of Additional Supplementary Files

File Name: Supplementary Movie 1

Description: 500 ns MD simulation of one of the three replicates. Simulation is of a single monomer of 14-3-3, CV2, and VHL.

File Name: Supplementary Movie 2

Description: Enlarged view of the 14-3-3/VHL protein interface, based on the 500 ns MD simulation of one of the three replicates. Simulation is of a single monomer of 14-3-3, CV2, and VHL.
