# Supplementary material for: Induced ubiquitination of the partially disordered estrogen receptor alpha via a 14-3-3 directed molecular glue-PROTAC
Source: Nat Commun. 2026 Jul 15;17:8696. doi: 10.1038/s41467-026-75333-w (PMC13490399; doi:10.1038/s41467-026-75333-w)
Supplement: Supplementary file 3 [file 41467_2026_75333_MOESM5_ESM.pdf]

nature portfolio

Corresponding author(s): Alessio Ciulli, Peter J. Cossar

Last updated by author(s): 30/11/2025

Reporting Summary

Nature Portfolio wishes to improve the reproducibility of the work that we publish. This form provides structure for consistency and transparency in reporting. For further information on Nature Portfolio policies, see our Editorial Policies and the Editorial Policy Checklist.

Statistics

For all statistical analyses, confirm that the following items are present in the figure legend, table legend, main text, or Methods section.

n/a	Confirmed	
☐	☒	The exact sample size (*n*) for each experimental group/condition, given as a discrete number and unit of measurement
☐	☒	A statement on whether measurements were taken from distinct samples or whether the same sample was measured repeatedly
☒	☐	The statistical test(s) used AND whether they are one- or two-sided<br>*Only common tests should be described solely by name; describe more complex techniques in the Methods section.*
☒	☐	A description of all covariates tested
☒	☐	A description of any assumptions or corrections, such as tests of normality and adjustment for multiple comparisons
☒	☐	A full description of the statistical parameters including central tendency (e.g. means) or other basic estimates (e.g. regression coefficient) AND variation (e.g. standard deviation) or associated estimates of uncertainty (e.g. confidence intervals)
☒	☐	For null hypothesis testing, the test statistic (e.g. *F*, *t*, *r*) with confidence intervals, effect sizes, degrees of freedom and *P* value noted<br>*Give P values as exact values whenever suitable.*
☒	☐	For Bayesian analysis, information on the choice of priors and Markov chain Monte Carlo settings
☒	☐	For hierarchical and complex designs, identification of the appropriate level for tests and full reporting of outcomes
☒	☐	Estimates of effect sizes (e.g. Cohen's *d*, Pearson's *r*), indicating how they were calculated

Our web collection on statistics for biologists contains articles on many of the points above.

Software and code

Policy information about availability of computer code

Data collection	Xcalibur (v4.4.16.14), Tecan Sparkcontrol (v3.1 SP1), EPU (v2.1)
Data analysis	Microsoft Excel (v2510), Origin 2020 (v9.7.0.188), MestReNova (v14.2.1-27684), cryoSPARC (v4.5), crYOLO (v1.9.9), Topaz (v0.2.5), deepEMhancer (v2.0), UCSF ChimeraX (v1.9), ISOLDE (v.1.10.1), Phenix (v.1.21), Schrodinger Maestro (v2023.1), ImageLab (v6.1), Proteome Discoverer (3.0.0.757)

For manuscripts utilizing custom algorithms or software that are central to the research but not yet described in published literature, software must be made available to editors and reviewers. We strongly encourage code deposition in a community repository (e.g. GitHub). See the Nature Portfolio guidelines for submitting code & software for further information.

Data

Policy information about availability of data

All manuscripts must include a data availability statement. This statement should provide the following information, where applicable:

- Accession codes, unique identifiers, or web links for publicly available datasets
- A description of any restrictions on data availability
- For clinical datasets or third party data, please ensure that the statement adheres to our policy

All data needed to evaluate the conclusions in the paper are present in the paper and/or the Supplementary Materials. Atomic models have been deposited to the PDB under ID 9SV3. Electron microscopy reconstructions have been deposited to the Electron Microscopy Databank (EMDB) under accession codes EMD-55233, EMD-55234, EMD-55235, EMD-55236 and EMD-55237. The mass spectrometry proteomics data have been deposited to the ProteomeXchange Consortium via the

PRIDE partner repository with the dataset identifier PXD071378 and PXD075638.

Research involving human participants, their data, or biological material

Policy information about studies with human participants or human data. See also policy information about sex, gender (identity/presentation), and sexual orientation and race, ethnicity and racism.

Reporting on sex and gender	n/a
Reporting on race, ethnicity, or other socially relevant groupings	n/a
Population characteristics	n/a
Recruitment	n/a
Ethics oversight	n/a

Note that full information on the approval of the study protocol must also be provided in the manuscript.

Field-specific reporting

Please select the one below that is the best fit for your research. If you are not sure, read the appropriate sections before making your selection.

[x] Life sciences [ ] Behavioural & social sciences [ ] Ecological, evolutionary & environmental sciences

For a reference copy of the document with all sections, see nature.com/documents/nr-reporting-summary-flat.pdf

Life sciences study design

All studies must disclose on these points even when the disclosure is negative.

Sample size	Quantitative biochemical assays (FA assays, ubiquitination quantification) were performed with a sample size of 3, providing sufficiently small standard deviations to support the conclusions. Qualitative assays (FA assay with 14-3-3 isoforms, time-course ubiquitination, and ubiquitination site mapping) were performed with a single representative experiment
Data exclusions	No data were excluded from the analyses.
Replication	Quantitative assays were independently replicated three times. Qualitative experiments were conducted once to illustrate representative outcomes.
Randomization	Samples were assigned to experimental conditions without bias; no further randomization was applied due to the nature of in vitro biochemical assays.
Blinding	Analyses were performed without blinding, as data interpretation was objective and based on quantitative measurements.

Reporting for specific materials, systems and methods

We require information from authors about some types of materials, experimental systems and methods used in many studies. Here, indicate whether each material, system or method listed is relevant to your study. If you are not sure if a list item applies to your research, read the appropriate section before selecting a response.

Materials & experimental systems

n/a	Involved in the study	
[ ]	[x]	Antibodies
[ ]	[x]	Eukaryotic cell lines
[x]	[ ]	Palaeontology and archaeology
[x]	[ ]	Animals and other organisms
[x]	[ ]	Clinical data
[x]	[ ]	Dual use research of concern
[x]	[ ]	Plants

Methods

n/a	Involved in the study	
[x]	[ ]	ChIP-seq
[x]	[ ]	Flow cytometry
[x]	[ ]	MRI-based neuroimaging

Antibodies

Antibodies used	ERα mAb (#8644, CST), pan 14-3-3 antibody (#8312, CST), anti-His mAb Tb-conjugate (61HISTLF, Revvity)
Validation	ERα mAb (#8644, CST), pan 14-3-3 antibody (#8312, CST), anti-His mAb Tb-conjugate (61HISTLF, Revvity) were commercially available and validated by the manufacturer. The phosporylated ERα antibody raised against the phosphorylated Thr594 of ERα was prepared by GL Biochem as described previously described (DOI: 10.1073/pnas.1220809110)

Eukaryotic cell lines

Policy information about cell lines and Sex and Gender in Research

Cell line source(s)	MCF7 cells were obtained from ATCC
Authentication	the MCF7 cell were authenticated using STR profiling (Cell Line Authentication Service of Eurofins Genomics, Ebersberg, Germany). Match was 98%
Mycoplasma contamination	All cell lines tested negative for mycoplasma contamination.
Commonly misidentified lines (See ICLAC register)	n/a

Plants

Seed stocks	n/a
Novel plant genotypes	n/a
Authentication	n/a
