# Supplementary material for: Induced ubiquitination of the partially disordered estrogen receptor alpha via a 14-3-3 directed molecular glue-PROTAC
Source: Nat Commun. 2026 Jul 15;17:8696. doi: 10.1038/s41467-026-75333-w (PMC13490399; doi:10.1038/s41467-026-75333-w)
Supplement: Supplementary file 4 [file 41467_2026_75333_MOESM6_ESM.pdf]

nature portfolio

Peer Review File

Induced ubiquitination of the partially disordered estrogen receptor alpha via a 14-3-3 directed molecular glue-PROTAC

Corresponding Author: Dr Peter Cossar

This file contains all reviewer reports in order by version, followed by all author rebuttals in order by version.

Version 0:

Reviewer comments:

Reviewer #1

(Remarks to the Author)
In the manuscript, which combines experiments and simulations, the authors developed a molecular glue-based PROTAC (MGPROTAC) approach to target and ubiquitinate the 14-3-3/Estrogen Receptor α (ERα) complex. This is the first time I read about a MGPROTAC, and I find the idea and work presented very original and interesting. Most of my comments are related to the computational part, since this is my background.

Major concerns

1- page 15: "As we could not detect measurable degradation of ERα in ERα-positive MCF7 cells
treated with CV2a, we turned our attention(...)"
There is something wrong with the MGPROTAC, if it does not induce degradation inside cells. This should be solved before publication.
The results mentioned should be shown at SI, they are relevant.

2- Methods - section "Molecular dynamics simulations"
a- Many details about the methods to perform molecular dynamics (MD) simulations are missing, preventing reproducibility of the work.
- Procedure for energy minimization and equilibration of the system (adjustment of pressure and temperature) before production MD simulations.
- Barostat and thermostat used.
- Treatment of long-range interactions.
- Force field used.
- Scheme to parametrize small molecules, especially partial atomic charges.
b- The standard in the field is to perform at least 3 replicas for every system, but it looks like only one replica was performed.
c- The methods section should clarify how the interactions presented in figures 3 and 4 were identified, and the criteria to consider that a hydrogen bond was formed.
d- It would be useful to show the root mean square fluctuation of the complex during MD simulations as SI.

3- page 13: "The MD simulation identified an extensive network of stabilizing inter-protein interactions. (...)"
I would not call it extensive network of inter-protein interactions, since figure 4 and the paragraph presents hydrogen bonds only.
Additionally, figure 4 provides only a static picture of hydrogen bonds. The presentation of such interactions would be improved if the authors showed, for instance, distance histograms for the heavy atoms involved in the interactions to show how stable such interactions are in MD simulations.

Minor concerns

- figure 1g: confusing. There is a mismatch between n and the number of linker atoms in the table and in the figure.

- figure 2b: the MGPROTACS with alkylic-linkers have a range of diverse behaviors, among themselves and in comparison to PEG linkers. This could be better explained / discussed in the manuscript (especially the change in anisotropy at very low

compound concentration).

- pages 12/13: "We therefore performed energy minimization and 500 ns molecular dynamics (MD) simulation to explore plausible conformations and interactions of the complex."
It is unclear if the authors used only the model from cryoEM or the full model (adding missing components) for MD simulations.

- page 13: "Together, our mass photometry, cryo-EM, and MD analyses reveal a network of favourable interprotein contacts between the VHL β-domain, CV2a, and 14-3-3ζ α8/α9 helices, rationalizing why CV2a displays the strongest cooperativity in the MGPROTAC series."
This paragraph has many overstatements, and needs rewriting.
In particular, only one complex is presented in cryoEM, and used to perform MD simulations. The authors do not present data that allows one to rationalize why CV2a displays the strongest cooperativity.

Reviewer #2

(Remarks to the Author)
Verhoef et al. Induced ubiquitination of the partially disordered Estrogen Receptor alpha protein via 14-3-3-directed molecular glue-based PROTAC design

The authors introduce a new concept of molecular glue-based PROTACs (^{MG}PROTACs), which is the development of a bridging molecule between the VHL E3 ligase and 14-3-3. A distinctive feature of this ^{MG}PROTAC strategy is that 14-3-3 recruits the partially disordered protein ERα, a well-known therapeutic target in breast cancer. Rather than directly linking an E3 ligase to a disease target, the described ^{MG}PROTAC stabilizes the interaction between 14-3-3 and ERα. Consequently, the phosphorylated C-terminal region of ERα binds more tightly to 14-3-3 in the presence of the compound. Notably, the fusicoccin scaffold contains a solvent-accessible moiety that allows attachment for a linker to generate a bi-functional PROTAC. The authors systematically tested various linkers to achieve efficient VHL-mediated ubiquitylation of ERα. Overall, the present data provide solid proof-of-concept validation. The underlying concept is robust and promising; however, this referee questions whether the ^{MG}PROTAC strategy described here may represent a rare and highly specific case limited to the particular molecular pairs examined.

Major concerns:

- The authors show, using TR-FRET-based affinity measurements, that recognition of 14-3-3 by the ^{MG}PROTAC alone appears to be weak, whereas the interaction is markedly enhanced in the presence of ERα. Cryo-EM structural data further support the idea that additional interactions between VHL and ERα contribute to this enhancement. However, this may not fully explain the observed ubiquitylation and subsequent degradation of ERα. According to the ubiquitylation and mass spectrometry analyses, VHL ubiquitylates not only ERα but also 14-3-3. Therefore, in a cellular context, 14-3-3 itself may be substantially depleted by the ^{MG}PROTAC. Moreover, the proposed/observed interaction between VHL and ERα from the cryo-EM structure should be interpreted with caution, as the resolution is insufficient for atomic-level detail. While it is appreciated that the authors likely made extensive efforts to improve the cryo-EM resolution, additional independent evidence supporting a direct interaction between VHL and ERα would greatly strengthen the proposed mechanistic explanation.

- The ^{MG}PROTAC binding site on 14-3-3 involves the α8 and α9 helices, which overlap with known binding sites for BRAF1, LRRK1, and HSPB6. It is therefore likely that the ^{MG}PROTAC binding could interfere with signaling pathways involving these proteins, potentially leading to unintended perturbations of physiologically important pathways. Have the authors considered these off-target or pathway-level effects in the context of future therapeutic development?

- The reported stoichiometry of the ternary complex is 14-3-3:ERα:VHL = 2:2:1. Dimeric 14-3-3 contains two binding sites for phosphorylated ERα, which itself forms a dimer. While the long, unstructured phosphorylated C-terminal tails of ERα could readily engage dimeric 14-3-3, it is difficult to rationalize why only a single VHL molecule associates with dimeric ERα. One possible explanation is a steric hindrance that prevents the accommodation of two VHL molecules within the ternary complex. Under this stoichiometry, VHL may bind asymmetrically, interacting predominantly with one ERα protomer or contacting both protomers in a non-equivalent manner. Have the authors observed any evidence for such asymmetric binding? If so, it would be valuable to illustrate this schematically in the figures (for example, by depicting the two ERα protomers in distinct colors).

Minor comments:

- In Fig. 3d, the cryo-EM map and model of the best resolved VCB/CV2a/14-3-3ζ/ERα species showed a 1:1:2:2 stoichiometry. While steric clash likely preclude binding two VCB molecules, it is unclear why only a single CV2a (^{MG}PROTAC) molecule is observed. The authors should provide an explanation for this observation.

- In Fig. 6f, high-molecular weight band (above the ERα(LBD_F)~Ub2) are detected upon blotting with an ERα antibody. What are the identities of these species? In particular, the strong band that disappears in the presence of CV2p and ATP.

- Although it is a common limitation at this stage, the ultimate goal of ^{MG}PROTAC development is therapeutic application in vivo. As noted by the authors, the current compound is not effective in the in cellular systems, despite robust in vitro biochemical activity. While inclusion of supporting cellular data would strengthen the manuscript, it may not be strictly mandatory. Nevertheless, the rationale for lack of cellular efficacy remains vague and should be discussed in greater depth.

- There is an error in reference 14; all subsequent references should be renumbered accordingly.

Reviewer #3

(Remarks to the Author)
The authors describe the development and characterization of MG-PROTACs targeting the protein-protein interaction between 14-3-3 and estrogen receptor alpha. The bifunctional compounds are based on the natural product analog FC-Nac, which stabilizes the disordered C-terminus of ERα when bound to 14-3-3, and in this work it is linked to VH-023, aiming to recruit the E3 ligase VHL and degrade the complex. The authors characterize eight MG-PROTAC in biophysical and cellular assays. Structurally, they were able to show the formation of a quaternary complex with cryo-EM. The overall approach is interesting, however, in the end, the MG-PROTACs did not lead to degradation. Nevertheless, this work could be of broad interest for the field of targeted protein degradation and molecular glues.

Comments to the authors:
1. Page 3, 2nd paragraph "Beyond degradation, induced proximity has expanded to encompass diverse heterobifunctional strategies that recruit other enzymatic machineries—including kinases, phosphatases, deubiquitinases, and lysosomal components—to alter protein function". This sentence is a nice summary of different approaches, but these strategies don't alter protein function. They alter protein fate, rewiring biological networks. I would recommend rephasing the last part of the sentence.
2. In the same paragraph, the citations for 14-3-3 glues are incomplete. There are more examples for 14-3-3/ERα (DOI: 10.1021/jacs.3c05161), 14-3-3/ChREBP (DOI: 10.1038/s41467-020-17741-0, DOI: 10.1002/anie.202521678), and 14-3-3/CFTR (DOI: 10.1038/s41467-022-31206-6) glues that should be included here.
3. The authors should elaborate more on the choice of VHL as the E3 ligase. Apart from the obvious connection with previous, successful examples from the Ciulli lab, is there a biological reason for recruiting only VHL? Is there any literature regarding specific E3 ligases that degrade 14-3-3? Additionally, given that Arvinas's ERα PROTAC Vepdegestrant (ARV-471) recruits CRBN, one might wonder if CRBN would be a better choice.
4. It would be nice to include in figure 1 the chemical structure of FC-Nac, instead of FC-A, since the analogs are based on FC-Nac. Looking at the crystal structure of FC-Nac in figure 1E, the choice of the attachment point for the linker (exit vector position) seems surprising. It appears to be buried/less accessible than the gem-dimethyl ether position on the sugar moiety, which can be easily modified chemically. This position seems more solvent-exposed in the crystal structure. Further explanations should be included, since the attachment position of the linker is known to have a substantial effect on the degradation potential of PROTACs.
5. The sentence "Notably, the final products contained a partial cleavage (5–43%) of the 2-methylbut-3-en-2-yl ether moiety on the fusicoccane ligand, however, this modification had negligible impact on 14-3-3/ERα stabilization" is not very clear. Does this imply partial cleavage during the synthesis or instability of the compounds in general? For which compounds did the authors notice 43% cleavage? This point seems significant and should be clarified.
6. The library size of 8 PROTACs seems very small. Maybe "library" is not the most appropriate term.
7. Page 6. "To assess MGPROTAC binding each compound was titrated to a 14-3-3/ ERα protein-peptide complex". The assay conditions (protein – peptide concentrations) should be mentioned. Additionally, 14-3-3 has seven isoforms with different client affinities. Which one was used in this assay? In the next sentence the authors state "All MGPROTAC, except for CV3a, displayed dose dependent binding (EC50 of 0.59—1.49 μM)", followed by the mention of the weakest PROTAC. It would be more useful to include which PROTAC was the most potent.
8. In the next paragraph, for protein titrations, it is common practice to report fold-stabilization in addition to apparent KD values.
9. For the FA HIF1α displacement assay it would be useful to include more details regarding the order of addition for the different proteins, pre-incubation times and ratios of the 14-3-3/ERα vs VHL/Elongin C/Elongin B (VCB). Some of these appear in the caption of figure 2 and the methods, but it would be better to include more details in the main text.
10. Similarly for the BRET VHL cellular assay, more experimental details should be included in the main text, including the EC50 values for the most potent PROTACs.
11. In page 8 "A VCB titration was performed at a fixed concentration of compound, and 14-3-3/ERα complex". What is the readout of this assay? Is it FP? What were the concentrations used and how were they chosen? More broadly, adding experimental details regarding optimization of assays for quaternary complexes would be of interest, preferably in the main text.
12. In page 9, where the authors investigate the effect of the different 14-3-3 isoforms on VCB recruitment, did they take into account the potential differences in Kd values of the binary 14-3-3/ERα complexes with the different isoforms? Adding a sentence or a table in a figure would be useful, including both Kd values and fold-stabilization.
13. In page 11, the first paragraph should include the IC50 values, as well, not only IC50 fold-change.
14. Can the authors elaborate on the following sentence? "This represents one of the first examples of controlled chemical-induced recruitment of an E3 ligase to a protein complex." Molecular glue degraders of CDK12/Cyclin K also recruit the CRL4-DDB1 ubiquitin ligase to a protein complex.
15. In figure 3d, the heterogeneity of the cryo-EM poses is perplexing. Although it is understandable that the authors focused

more on pose 1, could they add a short discussion for poses 2, 3 and the 14-3-3/ERα pose shown between those two? Since the percentages don't differ that much between poses 1, 2 and 3, does this imply that VHL interacts with 14-3-3 in the absence of ERα? This seems to contradict the biochemical data, at least by looking at the figure.
16. Page 15, "As we could not detect measurable degradation of ERα in ERα-positive MCF7 cells treated with CV2a…". The authors should explain which assay was used for degradation, as well as include experimental details. Was the lack of degradation based on Western blots in the presence and absence of MG132 for example? This is the first mention of a degradation assay.
17. Page 19 "By covalently linking a molecular glue stabilizer ligand…". I would suggest removing the word "covalently" as it could also imply a covalent electrophilic warhead, which is not the case for these PROTACs.
18. The next sentence "we show that tuning the linker length and composition allows precise control" seems like an over-statement for such a small library of compounds. Please consider rephrasing.
19. In the discussion, the authors mention "A possible explanation for this lack of degradation is the limited characterized phosphorylation state of ERα at Thr594, which mediates 14-3-3 binding". Have the authors considered phospho-proteomics or in vitro phosphorylation to ensure that ERα is indeed phosphorylated? This seems like a weak argument, since in the absence of phosphorylation, 14-3-3 and ERα would not interact in the BRET assay in MCF7 cells. That said, the reviewer doesn't necessarily suggest additional experiments here but at least reconsidering the accuracy of this statement. Other plausible explanations for the lack of degradation could be the sub-optimal quaternary complex geometry, the positioning of the linker or DUB activity after the initial ubiquitination.
20. Although the overall approach is interesting, from a chemical perspective turning a monovalent MG to a bifunctional MG-PROTAC seems to have certain disadvantages, including the substantially increased molecular weight, decreased ligand efficiency and, as the authors note, reduced cell permeability. These limitations might be worth mentioning in the discussion.

I would recommend publication after revision.

Version 1:

Reviewer comments:

Reviewer #1

(Remarks to the Author)
I thank the authors for addressing my comments.
I am satisfied with most of the answers and manuscript changes. I still have some minor concerns (the number scheme follows the same number scheme of the previous report).
- comment 2
"Prior to production MD, the system was relaxed using the default Desmond relaxation protocol in three sequential steps." The authors could provide more information about the relaxation protocol. Is it a short MD simulation? How long? Which parameters?

- comment 3
Figure S8: it would be useful to see the distances for the 3 replicas. It seems like distances for only one replica are shown. The figure description can be improved, it is not clear which atoms were used for the distance calculation.
The distance in panel B looks too high for a hydrogen bond (assuming this is a donor-acceptor distance).

Reviewer #2

(Remarks to the Author)
Verhoef et al. "Induced ubiquitination of the partially disordered estrogen receptor alpha protein via a 14-3-3-directed molecular glue-based PROTAC design"

I have reviewed the detailed response from the authors and found they have revised the manuscript in a highly professional manner. My concerns have been fully addressed, including comments I had initially expected to be incorporated into the Discussion. Now, I am satisfied with the revised manuscript.

Reviewer #3

(Remarks to the Author)
The authors have addressed most of my comments and added significant experimental details. Most of the overstatements have been rephrased and the conclusions are adequately supported by the experimental data, in a more balanced way. However, there are still some issues that need to be addressed.
1) The authors report two different EC50 values for FC-NAc. "Consistent with this rationale, FC-NAc binds the 14-3-3σ/ERα complex with high affinity (EC50 = 15 nM), substantially stronger than FC-A (EC50 = 421 nM)." In the next paragraph, the authors state "The monovalent molecular glue FCNAc served as a positive control (EC50= 0.34 μM)". This EC50 value is not substantially different than the one previously mentioned for FC-A.
2) An additional point that needs clarification is the use of different 14-3-3 isoforms in different assays. Initially, 14-3-3σ is used in the fluorescence anisotropy assays. 14-3-3ζ is used in the cryo-EM and the MD simulations, but after that for a TR-FRET assay, 14-3-3γ is used instead. If ζ and ε showed the strongest VCB stabilization, why did the authors use a different

isoform for the TR-FRET assay?
3) In the IP-MS experiment at 24h, ERα was not significantly increased, based on the statistics. In the next paragraph the first sentence mentions “Given cell target engagement assays and 14-3-3 IP-MS experiments showed significant in cell target engagement of VHL and 14-3-3/ERα…”. This seems contradictory to the results observed in the IP-MS experiment and should be re-written to accurately reflect the previous data. Additionally, the term “target engagement assays” is also confusing, since in some cases it indicates the quaternary complex with VHL, whereas in other cases it refers to the 14-3-3/ERα complex.
4) Since ubiquitination is observed both on ERα and 14-3-3ζ with both compounds CV2a and CV3a, it is not clear what the authors try to conclude with the sentence “Ubiquitination level is enhanced by cooperative quaternary complex formation and target preference is fine-tuned by the chemical linker of the MGPROTAC”. If the fine-tuning was successful or the different ubiquitination levels were significant, one would expect successful degradation of the complex. As this is not the case, this conclusion should be rewritten.
5) There are several mistakes in the references. For example, in the introduction, where 14-3-3 molecular glues are cited, as ref. 19-24, only ref. 20 seems to be correct. In the discussion, the sentence “For example, the synthetic 14-3-3/ERα molecular glue stabilizer 181...” also has the wrong reference (currently ref. 22). References 57 and 58 in the text are also incorrect – possibly referring to references 58 and 59 in the list of references.

Open Access This Peer Review File is licensed under a Creative Commons Attribution 4.0 International License, which permits use, sharing, adaptation, distribution and reproduction in any medium or format, as long as you give appropriate credit to the original author(s) and the source, provide a link to the Creative Commons license, and indicate if changes were made.
In cases where reviewers are anonymous, credit should be given to 'Anonymous Referee' and the source.
The images or other third party material in this Peer Review File are included in the article’s Creative Commons license, unless indicated otherwise in a credit line to the material. If material is not included in the article’s Creative Commons license and your intended use is not permitted by statutory regulation or exceeds the permitted use, you will need to obtain permission directly from the copyright holder.
To view a copy of this license, visit https://creativecommons.org/licenses/by/4.0/

Reviewer #1

General remark (GR):

In the manuscript, which combines experiments and simulations, the authors developed a molecular glue-based PROTAC (MGPROTAC) approach to target and ubiquitinate the 14-3-3/Estrogen Receptor α (ERα) complex. This is the first time I read about a MGPROTAC, and I find the idea and work presented very original and interesting. Most of my comments are related to the computational part, since this is my background.

GR Response 1.0: We thank Reviewer One for their constructive reviewer report. We appreciate the suggestions, particularly around the MD modelling, which has improved the robustness of our experimental methods. Their expertise has been invaluable in improving the quality of this manuscript.

Comment 1.1

1- page 15: "As we could not detect measurable degradation of ERα in ERα-positive MCF7 cells treated with CV2a, we turned our attention(...)" There is something wrong with the MGPROTAC, if it does not induce degradation inside cells. This should be solved before publication.The results mentioned should be shown at SI, they are relevant.

Response 1.1

Reviewer 1 raises an important point regarding the absence of measurable ERα degradation in MCF-7 cells treated with CV2a. We agree that this point requires careful clarification and additional transparency. Below we detail our experimental efforts at multiple levels on the mechanism of action of our molecule, and provide the relevant data in the Supporting Information (now Figures S13), as requested.

1. Western blot optimization for ERα degradation in MCF-7 cells

We undertook extensive optimization of the cellular degradation assay prior to concluding that CV2a does not induce robust ERα degradation under the tested conditions.

1.1 Antibody validation and band assignment

Initial experiments used a non–phospho-specific ERα antibody (MA5-14104, Thermo Fisher Scientific) to detect total ERα. Multiple bands were observed. To identify the correct ERα band, we titrated Fulvestrant (ICI 182,780; 10–0.1 μM), a well-established ERα degrader (Fig. s13a). This control experiment demonstrated: The ~65 kDa band corresponds to ERα and that the ERα signal was poorly resolved under standard growth conditions.

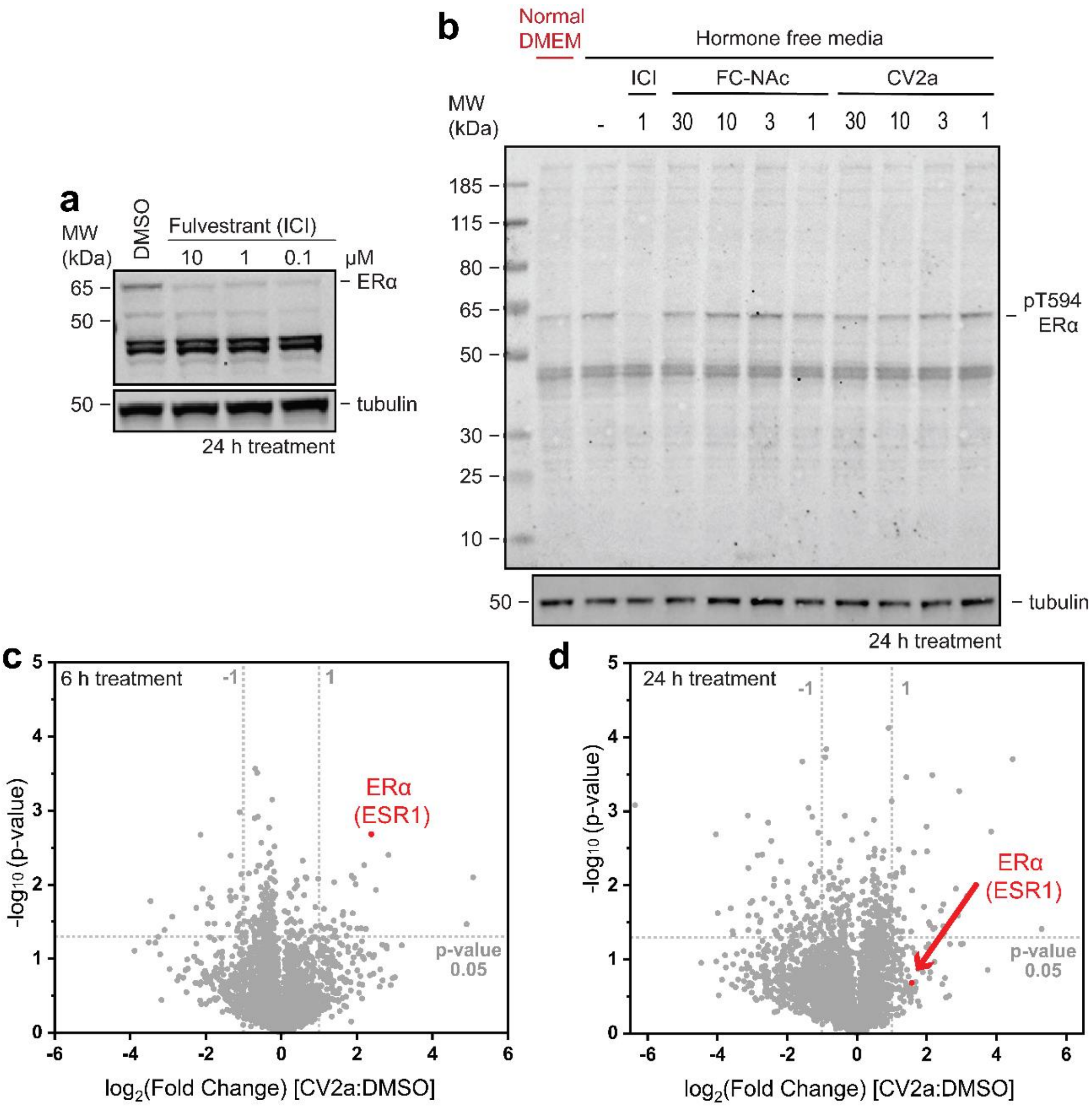

These data are now included in the SI **(Figure S13).**

1.2 Media optimization and use of phospho-specific antibody

To improve signal quality, cells were cultured in hormone-depleted media, which significantly enhanced ERα band resolution. Given specificity concerns with the total ERα antibody, we subsequently focused on a phospho-specific ERα antibody recognizing pT594 (Fig. S13b). This provided a cleaner signal. Cells were treated with DMSO, Fulvestrant (1 µM), FCNAc (30–1 µM), or CV1184 (30–1 µM). As shown in new SI Fig. S13; Fulvestrant (1 µM) induced clear degradation of the ~65 kDa band. As expected, FCNAc didn't not reduce pERα levels. However, in our hands CV2a also did not induce detectable degradation under these conditions.

2. Evidence for cellular target engagement

In our first submission, we had already evidenced that CV2a was cell-permeable using a VHL in-cell non-BRET target engagement assay in MCF-7 cells (Extended Figure 1). This supports

intracellular engagement of the E3 ligase component and argues against lack of permeability as the primary explanation.

To further investigate cellular effects, we performed 14-3-3 immunoprecipitation followed by mass spectrometry (IP–MS) at 6 h and 24 h after CV2a treatment (10 µM; Fig. S13d). At 6 h, ERα was enriched ~5-fold in the 14-3-3 pull-down upon CV2a treatment, with the strongest statistical significance among detected proteins. At 24 h, ERα enrichment decreased to ~2.5-fold and was no longer statistically significant ($p > 0.05$). This suggests that, while CV2a does not induce significant degradation, CV2a does engage and stabilize the 14-3-3/ERα complex in MCF-7 cells.

Attempts to monitor the pERα peptide by phosphoproteomics were unsuccessful. Specifically, we performed global phosphoproteomics on MCF7 cells treated with CV2a and DMSO to identify changes in the ER-pT594 phosphopeptide. This analysis identified 4,951 phosphopeptides corresponding to 1,948 proteins. However, we were unable to detect phosphopeptides from ERα, including pT594, likely due to limited phosphoproteome coverage and the known analytical challenges associated with negatively charged C-terminal phosphopeptides.

Together with the NanoBRET target engagement data, these results indicate that the MGPROTAC engages both components of the system (VHL and the 14-3-3/ERα complex) in cells.

3. Mechanistic considerations

A likely explanation for the absence of robust ERα degradation is the polypharmacology of FC-NAc. Previous proteomic studies using FC-A and related derivatives have shown that these molecules stabilize numerous 14-3-3 interactions (PMIDs: 32220335 and 37130519). Our 14-3-3 IPMS, further supports this showing CV2a, a derivative of FC-A, enriches several 14-3-3 complexes. Engagement of multiple 14-3-3 complexes could reduce the effective formation of a productive ternary complex with any single client protein such as ERα.

Alternatively, lifetime of the quaternary complex is too short under the cellular conditions to induce sufficient ubiquitination and require improved biophysical properties (cooperativity and/or affinity) or physical chemistry properties.

We respectfully submit that the central contribution of this work is the first conceptual demonstration that molecular glue stabilizers can be repurposed into bifunctional degraders targeting protein complexes. While CV2a does not induce robust ERα degradation in MCF-7 cells, it:

- Engages its intended components biophysically and cellularly
- Demonstrates complex-dependent recruitment and ubiquitination in vitro
- Establishes a design framework for future, more selective ^{MG}PROTACs.

We now explicitly clarify in the revised manuscript that CV2a should be regarded as a proof-of-concept molecule and that future work will focus on improving complex selectivity and degradation efficiency prior to advanced cellular studies.

All relevant optimization data have now been included in the Supporting Information, as requested. To clarify this to the readership, we have added additional text to the results & discussion and conclusion sections. Additionally, the 14-3-3 IPMS has been added to the methodology.

'Having established robust ^{MG}PROTAC-mediated recruitment of VHL to the 14-3-3/ERα complex, we next assessed degradation of the ERα complex in ERα-positive MCF-7 breast cancer cells. MCF-7 cells were cultured in hormone-free medium and treated for 24 h with vehicle (DMSO), fulvestrant (ICI, 1 μM)—a known ERα degrader—or increasing concentrations of FC-NAc or CV2a (0.3–30 μM). Levels of T594-phosphorylated ERα (pERα) were analysed using a pT594-specific antibody. As expected, DMSO and FC-NAc treatment showed no reduction in pERα level and ICI induced complete degradation of pERα (Fig. S13a-b).[37] However, CV2a failed to reduce pERα levels.

Given the absence of cellular degradation observed with CV2a, we performed 14-3-3 immunoprecipitation–mass spectrometry (IP–MS) experiments in MCF-7 cells to determine whether CV2a promotes enrichment of the 14-3-3/ERα complex in cells (Fig. S13c-d). Briefly, 14-3-3 IP–MS provides a snapshot of the cellular 14-3-3 interactome and enables assessment of how compound treatment influences 14-3-3 partner binding. As most 14-3-3 client proteins are phosphorylated, this approach primarily enables detection of phosphorylated proteins, such as phosphorylate ERα (pERα).

To assay CV2a enrichment of ERα, MCF-7 cells were cultured and treated with either DMSO (0.1% (v/v)) or 10 μM CV2a for 6 or 24 h. Endogenous 14-3-3 was then pulled-down using a pan-14-3-3 antibody coupled to magnetic beads. The resulting pull-down was then subject to washing, on-bead digestion, and LC-MS/MS analysis. IP–MS analysis following CV2a treatment revealed enrichment of 14-3-3/ERα interaction, and several other 14-3-3 interactions. At 6h, ERα was enriched ~5-fold in the 14-3-3 pull-down upon CV2a treatment, with the strongest statistical significance among detected proteins (Fig. S13c). At 24 h, ERα enrichment decreased to ~2.5-fold and was no longer statistically significant ($p > 0.05$; Fig. S13c). This finding indicates that whilst CV2a does not degrade ERα, it effectively engages and stabilizes the 14-3-3/ERα complex in MCF-7 cells, and several other 14-3-3 interactors. This observation is consistent with the previous proteomics work using FC-A and its semisynthetic derivatives, which demonstrated that FC-NAc induce polypharmacological perturbation of the 14-3-3 interactome.[47,48]'

And

'Despite robust biochemical ubiquitination of both 14-3-3ζ and ERα(LBD_F), as well as CV2a-mediated target engagement of VHL with the 14-3-3/ERα complex, no measurable reduction in ERα levels was detected in cells. Proteomic studies with FC-A and its semisynthetic analogues have been shown to elicit polypharmacological effects, specifically, stabilizing several 14-3-3 interactions,41,42 which our finding support. Such multi-complex engagement may dilute the formation of productive ternary complexes with ERα, thereby reducing the degradation efficiency of any individual client protein. Alternatively, the disconnect between biochemical and cellular ubiquitination may emerge from differences in cellular stoichiometries and requires improving the biophysical properties and physical chemistry properties of these proof-of-concept molecules. These challenges mirror early

limitations in PROTAC development, where issues such as permeability and molecular complexity were subsequently overcome through chemical optimization.55 '

And

'From a chemical perspective, advancing this strategy will require the development of more drug-like molecular glue scaffolds. Moving beyond semisynthetic FC-A derivatives toward fully synthetic, lower-molecular-weight compounds will be important. For example, the synthetic 14-3-3/ERα molecular glue stabilizer 181.22 In parallel, optimization of linker architecture and E3 ligase ligands—through reduction of hydrogen-bond donors, increased molecular rigidity, and minimization of polar surface area—should improve overall physical chemistry properties.'

Comment 1.2

2- Methods - section "Molecular dynamics simulations"

a- Many details about the methods to perform molecular dynamics (MD) simulations are missing, preventing reproducibility of the work.

- Procedure for energy minimization and equilibration of the system (adjustment of pressure and temperature) before production MD simulations.

- Barostat and thermostat used.

- Treatment of long-range interactions.

- Force field used.

- Scheme to parametrize small molecules, especially partial atomic charges.

b- The standard in the field is to perform at least 3 replicas for every system, but it looks like only one replica was performed.

c- The methods section should clarify how the interactions presented in figures 3 and 4 were identified, and the criteria to consider that a hydrogen bond was formed.

d- It would be useful to show the root mean square fluctuation of the complex during MD simulations as SI.

Response 1.2

We appreciate reviewer 1's comments and their input has significantly improved the quality of our MD data package. To address their comments, we have provided a point-by-point response bellow;

a- Many details about the methods to perform molecular dynamics (MD) simulations are missing, preventing reproducibility of the work.

The methods section has now been revised to include the request experimental details regarding the procedure for energy minimization and equilibration, Barostat and thermostat used, Treatment of long-range interactions, Force field used and the ligand parameters used, including partial atomic charges (see text below).

'Modelling of CV2a into the cryo-EM structure was performed using Maestro (Schrödinger), in triplicate. The docked structures of VH032 in complex with VHL and of fusicoccin A in complex with 14-3-3 were used for molecular dynamics simulations. Modifications to the chemical structures and modelling of the aliphatic linker region were performed using the

build tool in Maestro (Schrödinger). The resulting ternary VHL/CV2a/14-3-3 complex was pre-treated using the Protein Preparation Workflow (PPW) implemented in the Schrödinger Suite (Release 2024-3) prior to molecular dynamics (MD) simulations. Protein complex preparation was carried out at pH 7.5 by adding hydrogen atoms and building missing side-chain atoms. The hydrogen-bonding network was optimised by reorienting hydroxyl and thiol groups, water molecules, amide groups of asparagine (Asn) and glutamine (Gln), and the imidazole ring of histidine (His). Protonation states of histidine, aspartic acid (Asp), and glutamic acid (Glu), as well as tautomeric states of histidine, were assigned automatically by the PPW. MD simulations were performed using the Desmond Molecular Dynamics System[65] implemented within the Schrödinger Suite Release 2024-3 (Schrödinger, LLC). The prepared system was solvated in an orthorhombic box of explicit SPC water molecules, with a buffer distance of 12 Å between the solute and the box boundaries. The pH of the system was set to 7.5 and sodium and chloride ions were added to neutralise the system and to achieve a final salt concentration of 0.1 M. Ligand parameters, including partial atomic charges, were assigned using the OPLS4 force field as implemented in Schrödinger. Prior to production MD, the system was relaxed using the default Desmond relaxation protocol in three sequential steps. In the first step, positional restraints were applied to all atoms of the proteins and the ligand. In the second step, restraints were applied only to the Cα atoms of the proteins. In the final step, all positional restraints were removed, allowing full relaxation of the system while preserving the overall structural integrity. Production MD simulations were carried out in the NPT ensemble at 300 K and 1 bar using Desmond default settings, a Nosé–Hoover chain thermostat (relaxation time 1 ps), and a Martyna–Tobias–Klein barostat (relaxation time 2 ps). A multi-time-step RESPA integrator was employed, using a time step of 2 fs for bonded and short-range interactions and 6 fs for long-range interactions. Long-range electrostatic interactions were treated using the u-series decomposition with a real-space cut-off of 9 Å. 500 ns MD simulations were initiated from randomised initial velocities. Coordinates and energies were saved every 250 ps, resulting in 2,000 frames per trajectory.'

b- The standard in the field is to perform at least 3 replicas for every system, but it looks like only one replica was performed.

The MD simulation has now been performed in triplicate, and this has been highlighted in the Methods section.

c- The methods section should clarify how the interactions presented in figures 3 and 4 were identified, and the criteria to consider that a hydrogen bond was formed.

The following wording has now been added to the Methods: "Interactions that occurred more than 30% of the simulation time (0 to 500 ns) were shown. The geometric criteria for protein-ligand hydrogen-bonds was: a distance of 2.5 Å between the donor and acceptor atoms, a donor angle of ≥120° between the donor and hydrogen-acceptor atoms, and an acceptor angle of ≥90° between the hydrogen-acceptor-bonded atoms."

d- It would be useful to show the root mean square fluctuation of the complex during MD simulations as SI.

d – The root mean square fluctuation of the complex has now been shown in the Supplementary Information, Figure S7, and the following text add to the main manuscript;

'The protein residues involved in CV2a MGPROTAC binding remained relatively stable throughout all MD replicates, as evidenced by the RMSF profiles of the Ca carbons (Figure S7A–C, green bars).'

Comment 1.3

3- page 13: "The MD simulation identified an extensive network of stabilizing inter-protein interactions. (...)"

I would not call it extensive network of inter-protein interactions, since figure 4 and the paragraph presents hydrogen bonds only.

Additionally, figure 4 provides only a static picture of hydrogen bonds. The presentation of such interactions would be improved if the authors showed, for instance, distance histograms for the heavy atoms involved in the interactions to show how stable such interactions are in MD simulations.

Response 1.3

We agree that the initial language was excessive, and we have amended the language to simply 'a network'. We have also included time-dependent interaction diagrams in the Supplementary Information, Figure S7-8.

Comment 1.4

figure 1g: confusing. There is a mismatch between n and the number of linker atoms in the table and in the figure.

Response 1.4:

We thank Reviewer 1 for pointing out this source of confusion. In our original definition, the number of linker atoms corresponds to all consecutive heavy atoms between the two amide carbonyls (Figure 1g). Importantly, this definition includes the two methylene carbons adjacent to the FC-NAc amide group, as the ligand is connected to the PEG linker via an amide bond rather than directly through an ether linkage.

Accordingly, the total number of linker atoms is calculated as the sum of these two constant methylene carbons and the atoms contributed by the repeating PEG or alkylic units. For example, in CV2a this corresponds to 2 + (3 × 3) = 11 atoms between the two carbonyls. This approach was applied consistently across both linker series.

We recognize that the contribution of the two constant methylene carbons may not have been immediately apparent from the original figure, particularly for the alkylic linker series. To eliminate this ambiguity, we have revised the terminology from "linker atoms" to "total linker atoms" and updated Figure 1g to explicitly illustrate this definition.

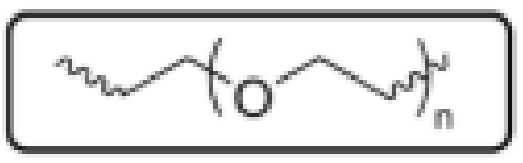

PROTAC	n	total linker atoms
CV1p	1	5
CV2p	3	11
CV3p	5	17
CV4p	7	23

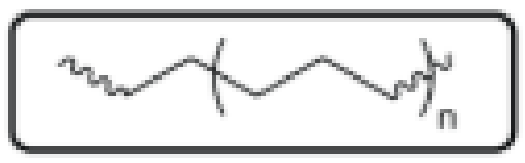

PROTAC	n	total linker atoms
CV0a	0	2
CV1a	1	5
CV2a	3	11
CV3a	5	17

Comment 1.5

figure 2b: the MGPROTACS with alkylic-linkers have a range of diverse behaviors, among themselves and in comparison to PEG linkers. This could be better explained / discussed in the manuscript (especially the change in anisotropy at very low compound concentration).

Response 1.5:

We agree and have rewritten the following discussion to better describe the structure-function relationship between the linker and VCB recruitment.

'Structure–activity relationship (SAR) analysis revealed distinct linker-dependent effects on ^{MG}PROTAC-induced VCB recruitment. PEG-based ^{MG}PROTACs displayed moderate positive recruitment across the series, yielding similar EC_{50} values (0.37–0.59 µM), with the shortest linker (CV1p) producing the smallest anisotropy shift. The minimal response observed for CV1p suggests that the short PEG-based linker, comprising five heavy atoms, is insufficient to effectively recruit VCB to the 14-3-3/ERα complex, consistent with steric constraints. Beyond a linker length of 11 heavy atoms (CV2p), PEG-based linkers exerted limited additional influence on VCB recruitment.

In contrast, alkyl-linked ^{MG}PROTACs exhibited greater diversity in cooperative behavior. The shortest alkyl-linked ^{MG}PROTAC (CV0a; two heavy atoms) disrupted 14-3-3/ERα complex formation, indicative of negative cooperativity, likely reflecting steric limitations imposed by insufficient linker length, similar to CV1p. Extension of the alkyl linker (CV1a–CV3a) restored cooperative recruitment, with CV2a and CV3a (11- and 17-heavy-atom linkers, respectively) displaying the largest anisotropy shifts across both linker series, albeit with a ~20-fold difference in EC_{50} values (0.10 and 1.95 µM, respectively; Fig. 2b). Notably, CV2a elicited increased anisotropy at very low VCB concentrations, below the intrinsic affinity of VH032 for VHL, indicative of highly cooperative complex formation relative to PEG-based and other alkyl-linked ^{MG}PROTACs. In contrast, recruitment of VCB by CV3a was only observed at high VCB concentrations, consistent with its reduced stabilization activity. This is likely due to the hydrophobic linker promoting non-productive conformations, thereby limiting effective engagement of the 14-3-3/ERα complex.'

Comment 1.6

pages 12/13: "We therefore performed energy minimization and 500 ns molecular dynamics

(MD) simulation to explore plausible conformations and interactions of the complex."
It is unclear if the authors used only the model from cryoEM or the full model (adding missing components) for MD simulations.

Response 1.6:

Thank you for highlighting this, we have added the following clarify sentence and updated the Methods section.

'Specifically, we excluded the poorly resolved ERα LBD, opting to model the VCB complex, a single 14-3-3 monomer, and CV2a.'

Comment 1.7

page 13: "Together, our mass photometry, cryo-EM, and MD analyses reveal a network of favourable interprotein contacts between the VHL β-domain, CV2a, and 14-3-3ζ α8/α9 helices, rationalizing why CV2a displays the strongest cooperativity in the MGPROTAC series."
This paragraph has many overstatements, and needs rewriting.
In particular, only one complex is presented in cryoEM, and used to perform MD simulations. The authors do not present data that allows one to rationalize why CV2a displays the strongest cooperativity.

Response 1.7:

We agree that the original statement overstated the conclusions. We have revised the text to more clearly reflect the structural context consistent with the observed biophysical trends, rather than a definitive explanation of CV2a cooperativity. The revised text now reads:

'Together, our cryo-EM and MD analyses provide structural context for the biophysical data, illustrating favourable interprotein contacts between the VHL β-domain and the 14-3-3ζ α8/α9 helices induced by the VHL-recruiting moiety of the ^{MG}PROTAC. In addition, an MD simulation of CV2a showed that its linker adopts an extended conformation compatible with simultaneous engagement of the VH032 and FC-NAc binding sites, whereas shorter linkers (e.g., CV0a and CV1a; 2 and 5 atoms, respectively) are less able to span this distance. These observations are consistent with the structure–activity relationships between linker length and cooperative complex formation.'

Reviewer #2

General remark 2.0:

Verhoef et al. Induced ubiquitination of the partially disordered Estrogen Receptor alpha protein via 14-3-3-directed molecular glue-based PROTAC design.

The authors introduce a new concept of molecular glue-based PROTACs (^{MG}PROTACs), which is the development of a bridging molecule between the VHL E3 ligase and 14-3-3. A distinctive feature of this ^{MG}PROTAC strategy is that 14-3-3 recruits the partially disordered protein ERα, a well-known therapeutic target in breast cancer. Rather than directly linking an E3 ligase to a disease target, the described ^{MG}PROTAC stabilizes the interaction between 14-3-3 and ERα. Consequently, the phosphorylated C-terminal region of ERα binds more tightly to 14-3-3 in the presence of the compound. Notably, the fusicoccin scaffold contains a solvent-accessible moiety that allows attachment for a linker to generate a bi-functional PROTAC. The authors systematically tested various linkers to achieve efficient VHL-mediated ubiquitylation of ERα. Overall, the present data provide solid proof-of-concept validation. The underlying concept is robust and promising; however, this referee questions whether the ^{MG}PROTAC strategy described here may represent a rare and highly specific case limited to the particular molecular pairs examined.

GR Response 2.0

We thank the reviewer for their constructive and thoughtful report. We particularly appreciate the comments regarding the broader conceptual implications of the MGPROTAC strategy.

With respect to the concern that the MGPROTAC approach described here may represent a rare or highly specific case limited to the particular molecular pair examined, we believe that this will likely not prove to be the case, though appreciate this will require further work in future. We however confidently posit that this strategy is likely broadly applicable as more molecular glue stabilizers emerge and the field expands. There are a number of observations and considerations that support our views:

First, within the 14-3-3 proteins interactome, multiple molecular glue stabilizers have already been reported that enhance interactions between 14-3-3 and diverse client proteins, including Raf kinases, p65, Pin1, ChREBP, and FOXO1. These examples demonstrate that 14-3-3 functions as a versatile hub protein capable of mediating stabilized protein–protein interactions across a wide biological spectrum. Notably, these synthetic molecular glue stabilizers are significantly more selective the FC-NAc. Our ^{MG}PROTAC strategy leverages this inherent plasticity. Second, beyond the 14-3-3 system, the broader field of molecular glue stabilization is rapidly expanding. For example, Revolution Medicines recently reported the first-in-class KRAS(G12D) molecular glue inhibitor RMC-9805, highlighting the clinical potential of glue-based strategies targeting challenging protein interfaces. Thirdly, small-molecule stabilizers have been identified that enhance interactions within the KRAS–SOS1 complex, further illustrating that induced proximity through interface stabilization is not an isolated phenomenon but part of a growing and accelerating area of drug discovery. Beyond, it is clear that molecular glue stabilisers of protein-protein complexes are perhaps a more pervasive mechanism than previously thought, and in almost all cases they involved interfacial binding that leaves plenty of scope for chemical functionalisation at surface-exposed regions of the molecules – suggesting our approach could be widely applicable.

Collectively, these advances suggest that the principles underlying MGPROTAC design—namely, stabilization of a protein–protein interaction to create a productive platform for E3 ligase recruitment—are generalizable. While our current work provides proof-of-concept validation in the context of 14-3-3 and ERα, we view this as a representative example of a broader strategy that can be extended to other hub proteins and glue-stabilized complexes.

Comment 2.1

The authors show, using TR-FRET-based affinity measurements, that recognition of 14-3-3 by the ^{MG}PROTAC alone appears to be weak, whereas the interaction is markedly enhanced in the presence of ERα. Cryo-EM structural data further support the idea that additional interactions between VHL and ERα contribute to this enhancement. However, this may not fully explain the observed ubiquitylation and subsequent degradation of ERα. According to the ubiquitylation and mass spectrometry analyses, VHL ubiquitylates not only ERα but also 14-3-3. Therefore, in a cellular context, 14-3-3 itself may be substantially depleted by the ^{MG}PROTAC. Moreover, the proposed/observed interaction between VHL and ERα from the cryo-EM structure should be interpreted with caution, as the resolution is insufficient for atomic-level detail. While it is appreciated that the authors likely made extensive efforts to improve the cryo-EM resolution, additional independent evidence supporting a direct interaction between VHL and ERα would greatly strengthen the proposed mechanistic explanation.

Response 2.1

We agree that VHL can ubiquitylate both ERα and 14-3-3, and that the proposed VHL–ERα interaction inferred from cryo-EM should be interpreted cautiously due to limited resolution. Importantly, 14-3-3 is highly abundant in cells, often overexpressed in breast cancer, providing buffering against MGPROTAC-induced depletion. Our data show that CV2a preferentially promotes ERα ubiquitylation over 14-3-3 (Fig. 6g–h and Extended Fig. 9), indicating that target selectivity can be tuned through linker optimization.

Obtaining independent evidence for direct VHL–ERα contacts is challenging. TR-FRET is not well suited because the 48-amino-acid ERα F-domain is highly proteolysis-prone without 14-3-3 (Ottmann et al., PMID: 37224961), necessitating co-expression to stabilize the F-domain. Short ERα peptides therefore cannot report on VHL–ERα contacts.

Instead, we used mass photometry to detect intact higher-order assemblies and provide orthogonal evidence for MGPROTAC-dependent quaternary complex formation. Mass spectrometry data are also consistent with VHL and ERα proximity, though direct contacts outside the MGPROTAC-induced complex cannot be conclusively determined. Together, these complementary approaches support our mechanistic model while acknowledging structural limitations.

Comment 2.2

The ^{MG}PROTAC binding site on 14-3-3 involves the α8 and α9 helices, which overlap with known binding sites for BRAF1, LRRK1, and HSPB6. It is therefore likely that the ^{MG}PROTAC binding could interfere with signaling pathways involving these proteins, potentially leading to unintended perturbations of physiologically important pathways. Have the authors considered these off-target or pathway-level effects in the context of future therapeutic

development?

Response 2.2

Review 2 raises an interesting question. Previous work from our group in Eindhoven (PMID: 36926879) examined the 14-3-3 interactome using a panel of nine 14-3-3 binding peptides combined with computational modeling and mass spectrometry experiments. This study showed that the "binding penalty" for stabilizing a specific protein-protein interaction is distributed across the entire network of 14-3-3 interactions.

Briefly, when a specific complex is stabilized in a competitive mixture of binding partners, the reduction in binding affinity is shared relatively equally across all peptide–protein interactions, independent of their individual binding strengths.

Extrapolating to the broader 14-3-3 network, which consists of several hundred interactors, these results suggest that stabilizing a single protein complex is unlikely to cause major perturbations of 14-3-3–mediated signaling pathways. While off-target effects cannot be completely excluded, the network-level buffering makes significant unintended pathway perturbations less probable, an important consideration for future therapeutic development.

Comment 2.3

The reported stoichiometry of the ternary complex is 14-3-3:ERα:VHL = 2:2:1. Dimeric 14-3-3 contains two binding sites for phosphorylated ERα, which itself forms a dimer. While the long, unstructured phosphorylated C-terminal tails of ERα could readily engage dimeric 14-3-3, it is difficult to rationalize why only a single VHL molecule associates with dimeric ERα. One possible explanation is a steric hindrance that prevents the accommodation of two VHL molecules within the ternary complex. Under this stoichiometry, VHL may bind asymmetrically, interacting predominantly with one ERα protomer or contacting both protomers in a non-equivalent manner. Have the authors observed any evidence for such asymmetric binding? If so, it would be valuable to illustrate this schematically in the figures (for example, by depicting the two ERα protomers in distinct colors).

Please refer to response 2.4 where we provide an integrated response to the related points.

Comment 2.4

In Fig. 3d, the cryo-EM map and model of the best resolved VCB/CV2a/14-3-3ζ/ERα species showed a 1:1:2:2 stoichiometry. While steric clash likely preclude binding two VCB molecules, it is unclear why only a single CV2a (^{MG}PROTAC) molecule is observed. The authors should provide an explanation for this observation.

Response 2.3 and 2.4:

Reviewer 2 raises insightful and related points regarding the observed stoichiometry of the ^{MG}PROTAC-induced complexes; we therefore address comments 2.3 and 2.4 together.

The cryo-EM and mass photometry samples were prepared using a CV2a concentration selected to favor formation of the productive quaternary complex while minimizing excess free ^{MG}PROTAC that could give rise to a hook effect (1.2 equivalents CV2a relative to 1.2 equivalents VCB and 1.1 equivalents 14-3-3ζ/ERα). Under these conditions, both CV2a and

VHL are limiting relative to the 14-3-3ζ/ERα dimer-of-dimers, corresponding to an approximate 1:2 ratio of ligand to available 14-3-3/ERα binding sites.

Importantly, CV2a binding is cooperative and preferentially stabilizes the quaternary VCB/CV2a/14-3-3ζ/ERα assembly, rather than forming a stable ternary CV2a/14-3-3ζ/ERα complex in the absence of VCB. As a consequence of the limiting ligand stoichiometry and cooperative assembly, only a single VHL–CV2a unit is incorporated per 14-3-3ζ/ERα dimer-of-dimers under the conditions used.

We do not observe clear structural evidence for intrinsic asymmetric binding of VHL to the ERα dimer. Rather, the observed 2:2:1 (14-3-3:ERα:VHL) stoichiometry and the presence of electron density for a single ^{MG}PROTAC are consistent with the experimental molar ratios employed and the cooperative nature of quaternary complex formation. Binding of a second VHL–CV2a unit is therefore disfavored under these conditions and is not detected in the cryo-EM map.

To clarify this point for the reader, we have added the following statements to the manuscript:

"This molar ratio was selected to minimize hook effects and corresponds to a 1:2 ratio of ligand to available 14-3-3/ERα binding sites."

And

"Notably, only electron density corresponding to a single MGPROTAC is observed, consistent with CV2a being present at approximately half the molar ratio relative to the available 14-3-3/ERα binding sites."

Comment 2.5

In Fig. 6f, high-molecular weight band (above the ERα(LBD_F)~Ub2) are detected upon blotting with an ERα antibody. What are the identities of these species? In particular, the strong band that disappears in the presence of CV2p and ATP.

Response 2.5:

We thank the reviewer for raising this important point.

In Fig. 6f, several higher-molecular-weight bands are detected with the ERα antibody, including a prominent species at ~60 kDa that disappears in the presence of CV2p and ATP. Based on multiple lines of evidence, we conclude that these bands do not represent defined ERα species but instead arise from artificates of the antibody or low-level impurities that are cross-recognized by the antibody.

First, re-analysis of the Ponceau stain shows clear bands corresponding to ubiquitin, UBE1, Cul2, 14-3-3, ERα, and ubiquitinated products. Notably, no distinct band is observed at ~60 kDa, indicating that this species is not a major component of the reaction mixture.

Second, we performed a control western blot using the purified 14-3-3/ERα(LBD_F) complex probed with the ERα antibody (new data shown below). While the ERα(LBD_F) band is clearly detected, two faint additional bands are also visible, including a species at approximately ~60 kDa, corresponding to the band noted by the reviewer. This demonstrates that the signal is already present in the purified complex prior to the ubiquitination reaction.

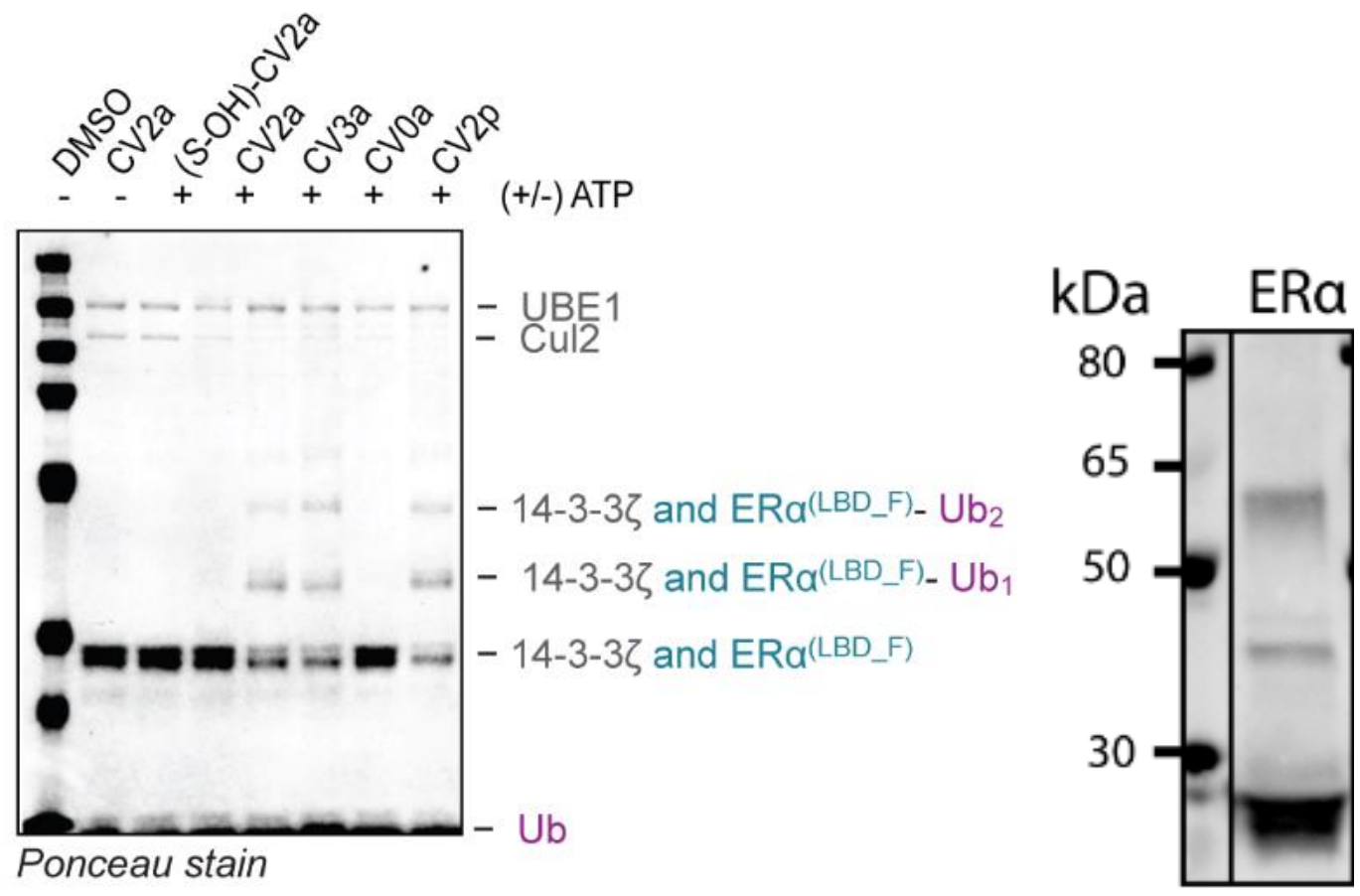

Third, intact mass spectrometry analysis of the purified complex confirms high sample purity. The chromatogram shows two dominant peaks, consistent with 14-3-3δ and ERα(LBD_F) co-expression . Deconvolution of mass-to-charge envelops aligns with the expected mass of the 14-3-3δ and ERα(LBD_F). No additional defined protein species were detected within the sensitivity limits of the method.

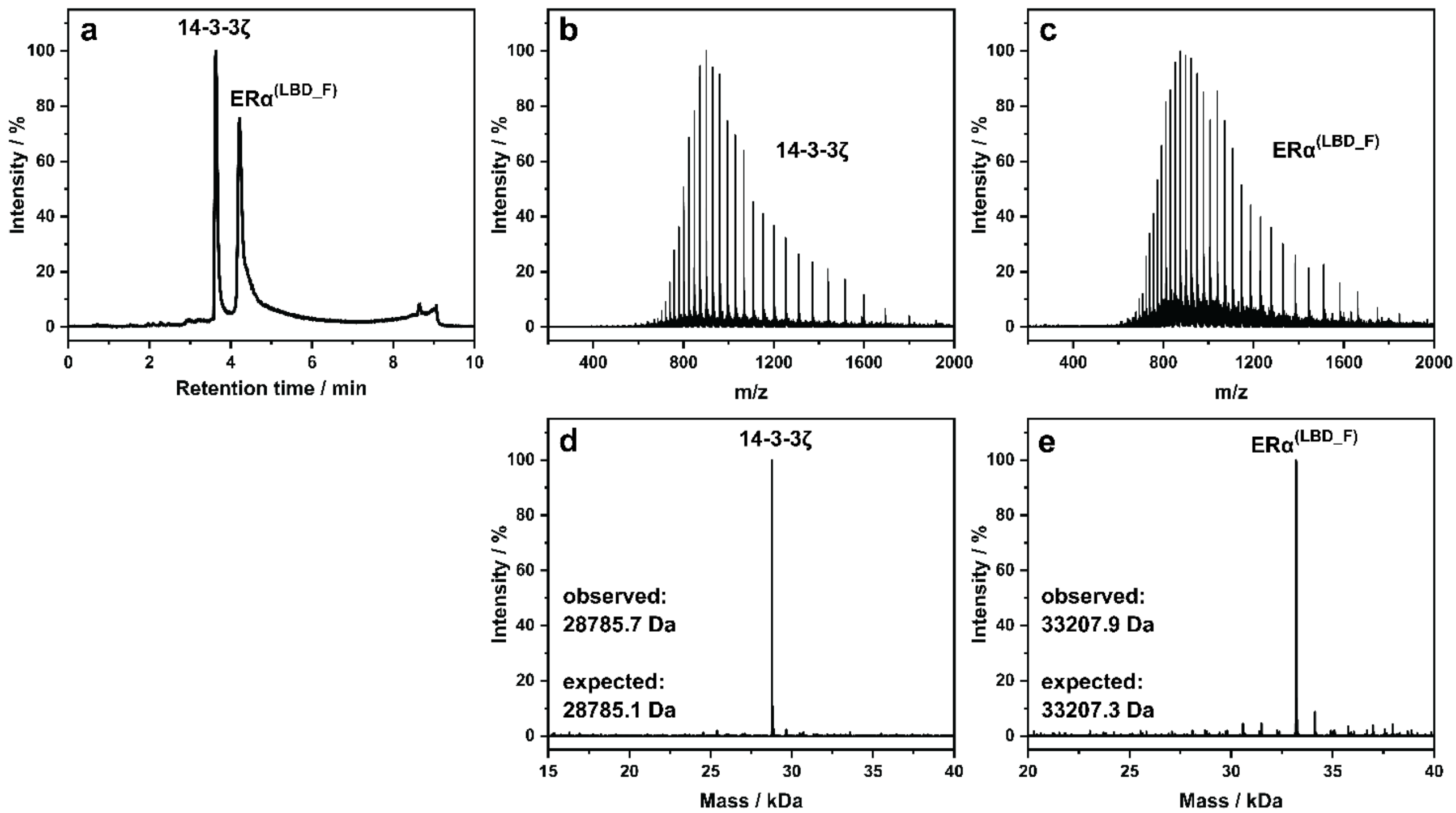

Taken together, these data indicate that the ~60 kDa band most likely reflects nonspecific recognition of a minor contaminant in the recombinant preparation. Trace bacterial proteins (for example, heat shock proteins in the ~60–70 kDa range) are known to co-purify at very low levels and can be detected by highly sensitive antibodies.

The disappearance of this band upon incubation with CV2p and ATP suggests that it is likely modified or consumed during the ubiquitination reaction. A plausible explanation is ubiquitination of a minor contaminant protein that is cross-recognized by the ERα antibody.

Bacterial heat shock proteins (e.g., DnaK/Hsp70 family members), which migrate around ~60–70 kDa, are well-known trace contaminants in bacterial recombinant protein preparations and can occasionally be detected by highly sensitive antibodies.

Comment 2.6

Although it is a common limitation at this stage, the ultimate goal of ^{MG}PROTAC development is therapeutic application in vivo. As noted by the authors, the current compound is not effective in the in cellular systems, despite robust in vitro biochemical activity. While inclusion of supporting cellular data would strengthen the manuscript, it may not be strictly mandatory. Nevertheless, the rationale for lack of cellular efficacy remains vague and should be discussed in greater depth.

Response 2.6

Reviewer 2 raises an important point. We have provided a detailed response to this point already (see Response 1.1 to Reviewer 1) – which we re-instate here for the Reviewer's benefit:

We agree that this point requires careful clarification and additional transparency. Below we detail our experimental efforts at multiple levels on the mechanism of action of our molecule, and provide the relevant data in the Supporting Information (now Figures S13), as requested.

1. Western blot optimization for ERα degradation in MCF-7 cells

We undertook extensive optimization of the cellular degradation assay prior to concluding that CV2a does not induce robust ERα degradation under the tested conditions.

1.1 Antibody validation and band assignment

Initial experiments used a non–phospho-specific ERα antibody (MA5-14104, Thermo Fisher Scientific) to detect total ERα. Multiple bands were observed. To identify the correct ERα band, we titrated Fulvestrant (ICI 182,780; 10–0.1 µM), a well-established ERα degrader (Fig. s13a). This control experiment demonstrated: The ~65 kDa band corresponds to ERα and that the ERα signal was poorly resolved under standard growth conditions.

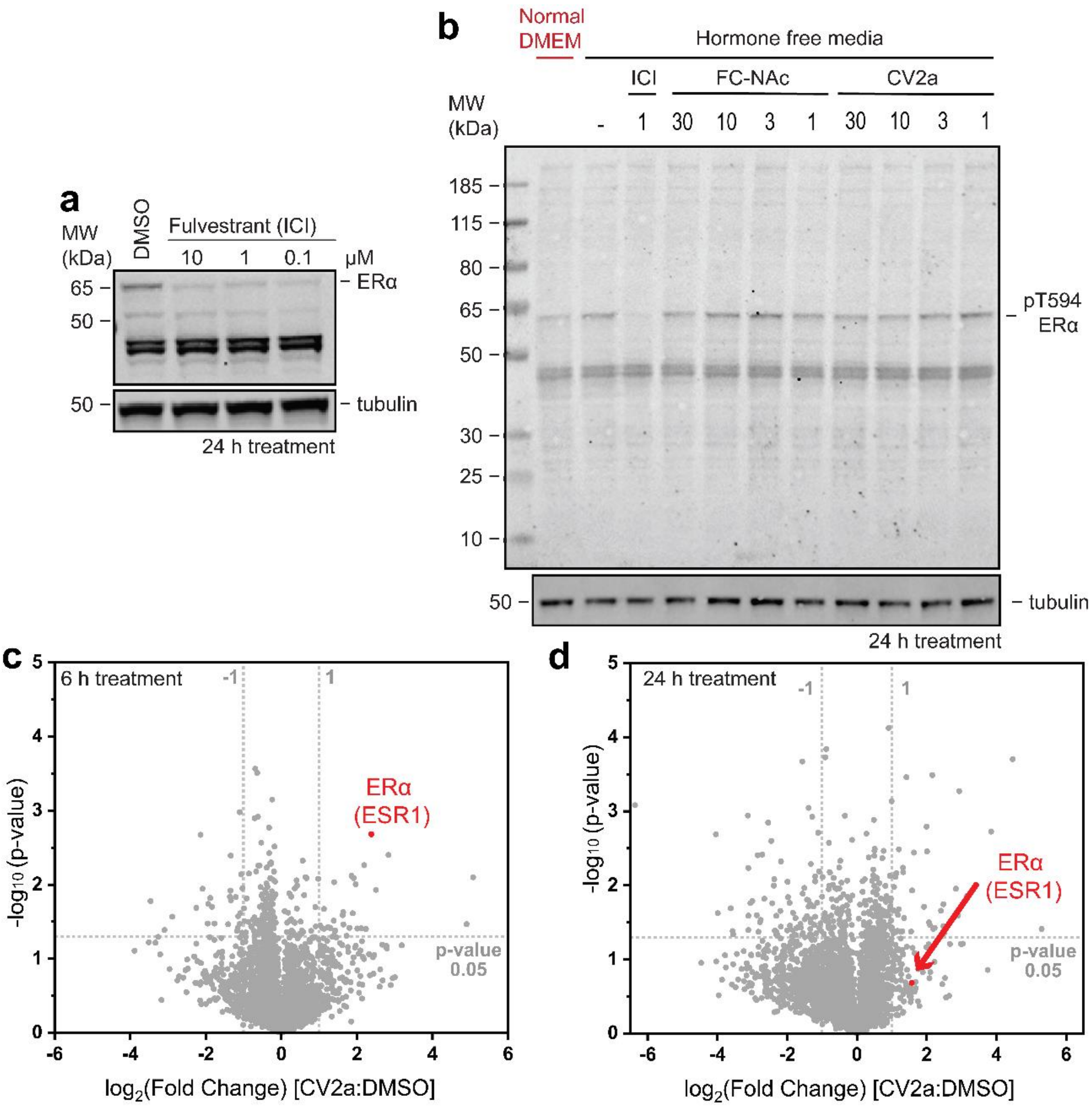

These data are now included in the SI **(Figure S13).**

1.2 Media optimization and use of phospho-specific antibody

To improve signal quality, cells were cultured in hormone-depleted media, which significantly enhanced ERα band resolution. Given specificity concerns with the total ERα antibody, we subsequently focused on a phospho-specific ERα antibody recognizing pT594 (Fig. S13b). This provided a cleaner signal. Cells were treated with DMSO, Fulvestrant (1 μM), FCNAc (30–1 μM), or CV1184 (30–1 μM). As shown in new SI Fig. S13; Fulvestrant (1 μM) induced clear degradation of the ~65 kDa band. As expected, FCNAc didn't not reduce pERα levels. However, in our hands CV2a also did not induce detectable degradation under these conditions.

2. Evidence for cellular target engagement

In our first submission, we had already evidenced that CV2a was cell-permeable using a VHL in-cell non-BRET target engagement assay in MCF-7 cells (Extended Figure 1). This supports

intracellular engagement of the E3 ligase component and argues against lack of permeability as the primary explanation.

To further investigate cellular effects, we performed 14-3-3 immunoprecipitation followed by mass spectrometry (IP–MS) at 6 h and 24 h after CV2a treatment (10 µM; Fig. S13d). At 6 h, ERα was enriched ~5-fold in the 14-3-3 pull-down upon CV2a treatment, with the strongest statistical significance among detected proteins. At 24 h, ERα enrichment decreased to ~2.5-fold and was no longer statistically significant ($p > 0.05$). This suggests that, while CV2a does not induce significant degradation, CV2a does engage and stabilize the 14-3-3/ERα complex in MCF-7 cells.

Attempts to monitor the pERα peptide by phosphoproteomics were unsuccessful. Specifically, we performed global phosphoproteomics on MCF7 cells treated with CV2a and DMSO to identify changes in the ER-pT594 phosphopeptide. This analysis identified 4,951 phosphopeptides corresponding to 1,948 proteins. However, we were unable to detect phosphopeptides from ERα, including pT594, likely due to limited phosphoproteome coverage and the known analytical challenges associated with negatively charged C-terminal phosphopeptides.

Together with the NanoBRET target engagement data, these results indicate that the MGPROTAC engages both components of the system (VHL and the 14-3-3/ERα complex) in cells.

3. Mechanistic considerations

A likely explanation for the absence of robust ERα degradation is the polypharmacology of FC-NAc. Previous proteomic studies using FC-A and related derivatives have shown that these molecules stabilize numerous 14-3-3 interactions (PMIDs: 32220335 and 37130519). Our 14-3-3 IPMS, further supports this showing CV2a, a derivative of FC-A, enriches several 14-3-3 complexes. Engagement of multiple 14-3-3 complexes could reduce the effective formation of a productive ternary complex with any single client protein such as ERα.

Alternatively, lifetime of the quaternary complex is too short under the cellular conditions to induce sufficient ubiquitination and require improved biophysical properties (cooperativity and/or affinity) or physical chemistry properties.

We respectfully submit that the central contribution of this work is the first conceptual demonstration that molecular glue stabilizers can be repurposed into bifunctional degraders targeting protein complexes. While CV2a does not induce robust ERα degradation in MCF-7 cells, it:

- Engages its intended components biophysically and cellularly
- Demonstrates complex-dependent recruitment and ubiquitination in vitro
- Establishes a design framework for future, more selective ^{MG}PROTACs.

We now explicitly clarify in the revised manuscript that CV2a should be regarded as a proof-of-concept molecule and that future work will focus on improving complex selectivity and degradation efficiency prior to advanced cellular studies.

All relevant optimization data have now been included in the Supporting Information, as requested. To clarify this to the readership, we have added additional text to the results & discussion and conclusion sections. Additionally, the 14-3-3 IPMS has been added to the methodology.

'Having established robust ^{MG}PROTAC-mediated recruitment of VHL to the 14-3-3/ERα complex, we next assessed degradation of the ERα complex in ERα-positive MCF-7 breast cancer cells. MCF-7 cells were cultured in hormone-free medium and treated for 24 h with vehicle (DMSO), fulvestrant (ICI, 1 μM)—a known ERα degrader—or increasing concentrations of FC-NAc or CV2a (0.3–30 μM). Levels of T594-phosphorylated ERα (pERα) were analysed using a pT594-specific antibody. As expected, DMSO and FC-NAc treatment showed no reduction in pERα level and ICI induced complete degradation of pERα (Fig. S13a-b).[37] However, CV2a failed to reduce pERα levels.

Given the absence of cellular degradation observed with CV2a, we performed 14-3-3 immunoprecipitation–mass spectrometry (IP–MS) experiments in MCF-7 cells to determine whether CV2a promotes enrichment of the 14-3-3/ERα complex in cells (Fig. S13c-d). Briefly, 14-3-3 IP–MS provides a snapshot of the cellular 14-3-3 interactome and enables assessment of how compound treatment influences 14-3-3 partner binding. As most 14-3-3 client proteins are phosphorylated, this approach primarily enables detection of phosphorylated proteins, such as phosphorylate ERα (pERα).

To assay CV2a enrichment of ERα, MCF-7 cells were cultured and treated with either DMSO (0.1% (v/v)) or 10 μM CV2a for 6 or 24 h. Endogenous 14-3-3 was then pulled-down using a pan-14-3-3 antibody coupled to magnetic beads. The resulting pull-down was then subject to washing, on-bead digestion, and LC-MS/MS analysis. IP–MS analysis following CV2a treatment revealed enrichment of 14-3-3/ERα interaction, and several other 14-3-3 interactions. At 6h, ERα was enriched ~5-fold in the 14-3-3 pull-down upon CV2a treatment, with the strongest statistical significance among detected proteins (Fig. S13c). At 24 h, ERα enrichment decreased to ~2.5-fold and was no longer statistically significant ($p > 0.05$; Fig. S13c). This finding indicates that whilst CV2a does not degrade ERα, it effectively engages and stabilizes the 14-3-3/ERα complex in MCF-7 cells, and several other 14-3-3 interactors. This observation is consistent with the previous proteomics work using FC-A and its semisynthetic derivatives, which demonstrated that FC-NAc induce polypharmacological perturbation of the 14-3-3 interactome.[47,48]'

And

'Despite robust biochemical ubiquitination of both 14-3-3ζ and ERα(LBD_F), as well as CV2a-mediated target engagement of VHL with the 14-3-3/ERα complex, no measurable reduction in ERα levels was detected in cells. Proteomic studies with FC-A and its semisynthetic analogues have been shown to elicit polypharmacological effects, specifically, stabilizing several 14-3-3 interactions,41,42 which our finding support. Such multi-complex engagement may dilute the formation of productive ternary complexes with ERα, thereby reducing the degradation efficiency of any individual client protein. Alternatively, the disconnect between biochemical and cellular ubiquitination may emerge from differences in cellular stoichiometries and requires improving the biophysical properties and physical chemistry properties of these proof-of-concept molecules. These challenges mirror early

limitations in PROTAC development, where issues such as permeability and molecular complexity were subsequently overcome through chemical optimization.55 '

And

'From a chemical perspective, advancing this strategy will require the development of more drug-like molecular glue scaffolds. Moving beyond semisynthetic FC-A derivatives toward fully synthetic, lower-molecular-weight compounds will be important. For example, the synthetic 14-3-3/ERα molecular glue stabilizer 181.22 In parallel, optimization of linker architecture and E3 ligase ligands—through reduction of hydrogen-bond donors, increased molecular rigidity, and minimization of polar surface area—should improve overall physical chemistry properties.'

Comment 2.7

There is an error in reference 14; all subsequent references should be renumbered accordingly.

Response 2.7

Thank you, this has been corrected, and all subsequent references have been renumbered accordingly.

Reviewer #3

General remark 3.0

The authors describe the development and characterization of MG-PROTACs targeting the protein-protein interaction between 14-3-3 and estrogen receptor alpha. The bifunctional compounds are based on the natural product analog FC-Nac, which stabilizes the disordered C-terminus of ERα when bound to 14-3-3, and in this work it is linked to VH-023, aiming to recruit the E3 ligase VHL and degrade the complex. The authors characterize eight MG-PROTAC in biophysical and cellular assays. Structurally, they were able to show the formation of a quaternary complex with cryo-EM. The overall approach is interesting, however, in the end, the MG-PROTACs did not lead to degradation. Nevertheless, this work could be of broad interest for the field of targeted protein degradation and molecular glues.

Response 3.0

We thank the reviewer for their supportive and constructive comments. We greatly appreciate their acknowledgment of the significance of our work on MG-PROTACs and the detailed feedback, which has helped us improve the clarity and presentation of the manuscript.

Comment 3.1

Page 3, 2nd paragraph "Beyond degradation, induced proximity has expanded to encompass diverse heterobifunctional strategies that recruit other enzymatic machineries—including kinases, phosphatases, deubiquitinases, and lysosomal components—to alter protein function". This sentence is a nice summary of different approaches, but these strategies don't alter protein function. They alter protein fate, rewiring biological networks. I would recommend rephasing the last part of the sentence.

Response 3.1

Reviewer 3 makes an insightful comment. We agree and have rephrased the sentence to:

'Beyond degradation, induced proximity strategies have expanded to include diverse heterobifunctional approaches that recruit alternative enzymatic machineries, such as kinases, phosphatases, deubiquitinases, and lysosomal components. Non-enzymatic heterobifunctional strategies have also emerged, including regulated induced proximity targeting chimeras (RIPTACs) and transcriptional chemical inducers of proximity (TCIPs). Together, these approaches modulate protein fate to rewire cellular networks .'

Comment 3.2

In the same paragraph, the citations for 14-3-3 glues are incomplete. There are more examples for 14-3-3/ERα (DOI: 10.1021/jacs.3c05161), 14-3-3/ChREBP (DOI: 10.1038/s41467-020-17741-0, DOI: 10.1002/anie.202521678), and 14-3-3/CFTR (DOI: 10.1038/s41467-022-31206-6) glues that should be included here.

Response 3.2

We agree that the exemplar references do not fully capture all relevant examples. As such we have inserted the suggested references.

Comment 3.3

The authors should elaborate more on the choice of VHL as the E3 ligase. Apart from the obvious connection with previous, successful examples from the Ciulli lab, is there a biological reason for recruiting only VHL? Is there any literature regarding specific E3 ligases that degrade 14-3-3? Additionally, given that Arvinas's ERα PROTAC Vepdegestrant (ARV-471) recruits CRBN, one might wonder if CRBN would be a better choice.

Response 3.3

Yes, we agree and have added additional discussion to both results and discussion sections of the manuscript.

'Further, we selected VHL as the target E3 ligase given its extensive use as E3 ligase ligand and tractability to biophysics and structural biology of ternary complexes, though recent advances in chemical warheads and structural enablement warrant future expansion to CRBN as well.'

And

'In parallel, exploring alternative E3 ligases, such as Cereblon or other Cullin RING ligases , represents a promising direction given the availability of high-quality ligands.55,56 CRBN ligands are an attractive alternative, with Arvinas's ERα PROTAC Vepdegestrant having entered phase three clinical trials for advanced breast cancer.57 Notably, constitutive

photomorphogenic 1 (COP1) has been reported to ubiquitinate and promote the degradation of 14-3-3σ58, however the lack of small molecule binders complicates ligand development. '

Comment 3.4

It would be nice to include in figure 1 the chemical structure of FC-Nac, instead of FC-A, since the analogs are based on FC-Nac. Looking at the crystal structure of FC-Nac in figure 1E, the choice of the attachment point for the linker (exit vector position) seems surprising. It appears to be buried/less accessible than the gem-dimethyl ether position on the sugar moiety, which can be easily modified chemically. This position seems more solvent-exposed in the crystal structure. Further explanations should be included, since the attachment position of the linker is known to have a substantial effect on the degradation potential of PROTACs.

Response 3.4

We thank reviewer 3 for highlighting this oversight. We have updated Figure 1.

While the gem-dimethyl ether on the sugar is also solvent-exposed, we chose FC-NAc for its stronger stabilizing effect on the 14-3-3/ERα complex compared to FC-A, and FCA-NAc has been demonstrated to be highly chemically tractable and tolerate several bulky modifications at this position. (PMID: 30025189 and 23192606. To further clarify this in the manuscript we have added the following.

'Consistent with this rationale, FC-NAc binds the 14-3-3σ/ERα complex with high affinity (EC_{50} = 15 nM), substantially stronger than FC-A (EC_{50} = 421 nM). Furthermore, FC-NAc is chemically tractable, and previous studies demonstrate its tolerance to bulky substitutions at this position.36,41,42 '

Comment 3.5

The sentence "Notably, the final products contained a partial cleavage (5–43%) of the 2-methylbut-3-en-2-yl ether moiety on the fusicoccane ligand, however, this modification had negligible impact on 14-3-3/ERα stabilization" is not very clear. Does this imply partial cleavage during the synthesis or instability of the compounds in general? For which compounds did the authors notice 43% cleavage? This point seems significant and should be clarified.

Response 3.5

The partial cleavage of the 2-methylbut-3-en-2-yl ether moiety occurred during HPLC purification of the ^{MG}PROTAC carried out in a water/acetonitrile mixture containing 0.1% formic acid. Specifically, during the concentration of the eluted fractions. Notably, reanalysis of the compound showed no further cleavage post purification. The following has been added to the manuscript.

Investigation of this gem-dimethyl ether cleavage revealed that the moiety is labile under the acidic conditions used during HPLC purification, with the highest extent of cleavage (43%) observed for CV0a. Importantly, this modification had a negligible impact on 14-3-3/ERα complex stabilization (Fig. S1).

Comment 3.6

The library size of 8 PROTACs seems very small. Maybe "library" is not the most appropriate term.

Response 3.6

Thank you for the suggestion. The word 'library' has been replaced with the word 'panel'

Comment 3.7

Page 6. "To assess MGPROTAC binding each compound was titrated to a 14-3-3/ ERα protein-peptide complex". The assay conditions (protein – peptide concentrations) should be mentioned. Additionally, 14-3-3 has seven isoforms with different client affinities. Which one was used in this assay? In the next sentence the authors state "All MGPROTAC, except for CV3a, displayed dose dependent binding (EC50 of 0.59—1.49 μM)", followed by the mention of the weakest PROTAC. It would be more useful to include which PROTAC was the most potent.

Response 3.7

We agree and have added the conditions to the main text referencing the binding experiment. Additionally, we have clarified the text around the binding affinity of the MGPROTAC panel, adding the following;

All MGPROTACs, with the exception of CV3a, displayed dose-dependent binding (EC_{50} = 0.59–1.49 μM; Fig. 2a), with CV2p exhibiting the highest apparent potency. In contrast, CV3a, which features a 17-atom aliphatic linker, showed markedly weaker binding (EC_{50} ≈ 40 μM).

Comment 3.8

In the next paragraph, for protein titrations, it is common practice to report fold-stabilization in addition to apparent KD values.

Response 3.8

We agree and have added the fold stabilization values alongside the apparent KD values.

Comment 3.9

For the FA HIF1α displacement assay it would be useful to include more details regarding the order of addition for the different proteins, pre-incubation times and ratios of the 14-3-3/ERα vs VHL/Elongin C/Elongin B (VCB). Some of these appear in the caption of figure 2 and the methods, but it would be better to include more details in the main text.

Response 3.9

Yes, to clarify FA-displacement assay is measured at equilibrium. Given that all components are in a dynamic equilibrium, the order of addition does not influence the measurement. Notably, the equilibrium of the system is reached in the 10s-100s of seconds.

We appreciate that the reviewer is an expert in the field and appreciate their interest. However, Nature Comms is a broad interest and non-technical journal, and a high-level of technical detail may make the manuscript less accessible to the non-expert. As such we have left technical details pertaining to concentrations to the captions and methods.

Comment 3.10

Similarly for the BRET VHL cellular assay, more experimental details should be included in the main text, including the EC50 values for the most potent PROTACs.

Response 3.10

We agree and have revised the text to read.

'All MGPROTACs except CV3a displaced the probe with potencies comparable to VH032, exhibiting IC_{50} values in the 0.3–1.3 μM range. In non-permeabilized MCF-7 cells, marked differences in cellular permeability were observed. MGPROTACs bearing alkylic linkers showed improved cell penetration relative to their PEG-linked counterparts, with IC_{50} values ranging from 1.0 to >10 μM versus >10 μM for PEG-linked analogues, respectively. Notable exceptions were CV0a and CV3a (Extended Fig. 1c). The poor engagement of CV0a (IC_{50} > 10 μM) is likely due to its short linker, which increases overall polarity and limits membrane diffusion. In contrast, the reduced activity of CV3a (IC_{50} = 1.4 μM) is hypothesized to arise from the hydrophobic nature of its long aliphatic linker, potentially compromising solubility and effective ligand engagement. Combined, the results of the FA assays and VHL target engagement experiments demonstrated that the MGPROTAC effectively engaged both 14-3-3/ERα and VHL complexes.'

Comment 3.11

In page 8 "A VCB titration was performed at a fixed concentration of compound, and 14-3-3/ERα complex". What is the readout of this assay? Is it FP? What were the concentrations used and how were they chosen? More broadly, adding experimental details regarding optimization of assays for quaternary complexes would be of interest, preferably in the main text.

Response 3.11

We agree that additional experimental detail are helpful. The readout of the VCB titration assay is fluorescence anisotropy (FA), which reports binding of the fluorescently labeled ERα peptide to 14-3-3 in the presence of VHL. To clarify the assay design, we have added the following text to the main manuscript:

'Recruitment of the VCB complex to the 14-3-3/ERα complex was investigated by titrating VCB to a fixed concentration of compound (1 μM), fluorescently labeled ERα peptide (10 nM), and 14-3-3 (500 nM). This 14-3-3 concentration corresponds to approximately 40% bound ERα peptide. Fluorescence anisotropy was used as the readout to monitor ERα binding.'

A partial-occupancy regime (~40% ERα bound) was selected to enable detection of both positive and negative cooperative effects upon VCB addition.

Comment 3.12

In page 9, where the authors investigate the effect of the different 14-3-3 isoforms on VCB recruitment, did they take into account the potential differences in Kd values of the binary 14-3-3/ERα complexes with the different isoforms? Adding a sentence or a table in a figure would be useful, including both Kd values and fold-stabilization.

Response 3.12

Thank you for your helpful observation. Yes, we accounted for differences in the binary affinities of the 14-3-3/ERα complexes across the different isoforms (K_D values ranging from 2.0 to 29 μM). To ensure comparison of VCB recruitment across isoforms, we quantified stabilization factors (fold stabilization) rather than apparent binding affinities. Each stabilization factor was calculated using the corresponding binary KD of the relevant 14-3-3 isoform. This normalization allows direct comparison of ternary complex stabilization independent of intrinsic isoform-dependent affinity differences.

To improve clarity for the reader, we have added a sentence to the main text highlighting this approach. In addition, we recognized that the previous cross-reference to Extended Figure 3 may have been confusing and have repositioned it accordingly.

' To account for isoform-dependent differences in ERα affinity (K_D ranging from 2 to 29 μM), we compared the extent of stabilization for different 14-3-3 isoforms following treatment with CV2a in the presence or absence of VCB.'

Comment 3.13

In page 11, the first paragraph should include the IC50 values, as well, not only IC50 fold-change.

Response 3.13

We agree and have revised the text to explicitly include the absolute IC_{50} values alongside the corresponding fold-changes, as requested

'Cotreatment with the 14-3-3/ERα$^{(LBD_F)}$ protein–protein complex and either CV2a or CV3a significantly enhanced displacement of HIF1α probe from VCB by the MGPROTACs compared to ^{MG}PROTAC alone. This resulted in a 2.8-fold reduction in the IC_{50} for HIF1α probe displacement (0.37 μM) for CV2a and a >34-fold reduction (2.9 μM) for CV3a (Fig. 3a and Extended Fig. 4a). In contrast, the negatively cooperative ^{MG}PROTAC CV0a reduced HIF1α probe displacement in the presence of the complex, increasing the IC_{50} by 4-fold to 5.4 μM. Together, these data are consistent with ^{MG}PROTAC engagement of the full-length 14-3-3/ERα protein complex.'

Comment 3.14

Can the authors elaborate on the following sentence? "This represents one of the first examples of controlled chemical-induced recruitment of an E3 ligase to a protein complex." Molecular glue degraders of CDK12/Cyclin K also recruit the CRL4-DDB1 ubiquitin ligase to a protein complex.

Response 3.14

We agree with the reviewer that molecular glue degraders of the CDK12/Cyclin K complex represent an important precedent in which an E3 ligase (CRL4–DDB1) is functionally linked to a protein complex. However, in this system, Cyclin K degradation occurs via a piggy-back mechanism, in which the small molecule induces direct recruitment of CDK12 to the ligase, and Cyclin K is co-degraded as an associated binding partner rather than through chemically induced recruitment to the E3 ligase itself.

In contrast, in our system the small molecule directly and controllably induces recruitment of the E3 ligase to a pre-formed protein complex, rather than recruiting a single protein that brings along its binding partner. It is this aspect of chemical control over E3 ligase recruitment to a protein complex that we refer to in the manuscript.

We note that our statement deliberately uses the wording "one of the first examples" and does not claim absolute precedence. We believe the distinction described above supports the accuracy of the statement as written.

Comment 3.15

In figure 3d, the heterogeneity of the cryo-EM poses is perplexing. Although it is understandable that the authors focused more on pose 1, could they add a short discussion for poses 2, 3 and the 14-3-3/ERα pose shown between those two? Since the percentages don't differ that much between poses 1, 2 and 3, does this imply that VHL interacts with 14-3-3 in the absence of ERα? This seems to contradict the biochemical data, at least by looking at the figure.

Response 3.15

We appreciate Reviewer 3's careful analysis of the heterogeneity observed in the cryo-EM reconstructions. While poses 2 and 3 are less well resolved than pose 1, we hypothesize that in these classes the ERα C-terminal tail remains bound within the 14-3-3 binding groove together with CV2a. Owing to the intrinsically disordered nature of the ERα C-terminus, the ligand-binding domain (LBD) is likely highly dynamic and samples multiple orientations in solution, resulting in poorer local resolution and increased apparent heterogeneity.

Importantly, although the relative particle populations of poses 1–3 are comparable, we do not interpret these data as evidence for VHL binding to 14-3-3 in the absence of ERα. This interpretation would indeed contradict our biochemical data. Consistent with this, TR-FRET experiments demonstrate that CV2a is unable to recruit VHL in the absence of ERα peptide, indicating that ERα is required for productive ternary complex formation.

We therefore interpret poses 2 and 3 as alternative conformational states of the same ERα-dependent ternary assembly rather than distinct binding modes lacking ERα. We have clarified this interpretation in the text to avoid potential confusion.

'Although multiple cryo-EM poses are observed, all classes are interpreted as ERα-dependent ternary assemblies, with heterogeneity arising from the conformational flexibility of the ERα C-terminal region and ligand-binding domain.'

Further, we have expanded our discussion of the TR-FRET experiments by adding the following;

'—consistent with the proposed mechanism in which 14-3-3 acts as a scaffold to mediate E3 ligase recruitment to ERα and validating our interpretation that all cryo-EM classes are ERα-dependent ternary assemblies'

Comment 3.16

Page 15, "As we could not detect measurable degradation of ERα in ERα-positive MCF7 cells treated with CV2a…". The authors should explain which assay was used for degradation, as well as include experimental details. Was the lack of degradation based on Western blots in the presence and absence of MG132 for example? This is the first mention of a degradation assay.

Response 3.16

We agree that the description of the degradation assay was not sufficiently clear in the original manuscript. We have now clarified the assay description and added the relevant experimental details to the revised manuscript. For a comprehensive discussion of the degradation of experiments and their interpretation, refer to **response 1.1.**

Comment 3.17

Page 19 "By covalently linking a molecular glue stabilizer ligand…". I would suggest removing the word "covalently" as it could also imply a covalent electrophilic warhead, which is not the case for these PROTACs.

Response 3.17

We agree that the term "covalently" could be misleading in this context and we have therefore removed it from the revised manuscript to avoid any potential confusion.

'By linking a molecular glue stabilizer ligand…'

Comment 3.18

The next sentence "we show that tuning the linker length and composition allows precise control" seems like an over-statement for such a small library of compounds. Please consider rephrasing.

Response 3.18

We agree and have revised the text to read.

‘Furthermore, we show that tuning the linker length and composition modulates MGPROTAC cooperativity, E3 ligase recruitment, and site-selective ubiquitination efficiency.’

Comment 3.19

In the discussion, the authors mention “A possible explanation for this lack of degradation is the limited characterized phosphorylation state of ERα at Thr594, which mediates 14-3-3 binding”. Have the authors considered phospho-proteomics or in vitro phosphorylation to ensure that ERα is indeed phosphorylated? This seems like a weak argument, since in the absence of phosphorylation, 14-3-3 and ERα would not interact in the BRET assay in MCF7 cells. That said, the reviewer doesn’t necessarily suggest additional experiments here but at least reconsidering the accuracy of this statement. Other plausible explanations for the lack of degradation could be the sub-optimal quaternary complex geometry, the positioning of the linker or DUB activity after the initial ubiquitination.

Response 3.19

We thank the reviewer for this thoughtful comment and agree that our original statement may have been overly speculative. For a broader discussion of the degradation experiments and their interpretation, we refer reviewer 3 to **response 1.1**, in this text we specifically discuss our proteomics efforts. Below we have provided an exert from the discussion specifically detailing our IP MS and phosphoproteomics efforts, for your convenience.

2. Evidence for cellular target engagement

In our first submission, we had already evidenced that CV2a was cell-permeable using a VHL in-cell non-BRET target engagement assay in MCF-7 cells (Extended Figure 1). This supports intracellular engagement of the E3 ligase component and argues against lack of permeability as the primary explanation.

To further investigate cellular effects, we performed 14-3-3 immunoprecipitation followed by mass spectrometry (IP–MS) at 6 h and 24 h after CV2a treatment (10 μM; Fig. S13d). At 6 h, ERα was enriched ~5-fold in the 14-3-3 pull-down upon CV2a treatment, with the strongest statistical significance among detected proteins. At 24 h, ERα enrichment decreased to ~2.5-fold and was no longer statistically significant ($p > 0.05$). This suggests that, while CV2a does not induce significant degradation, CV2a does engage and stabilize the 14-3-3/ERα complex in MCF-7 cells.

Attempts to monitor the pERα peptide by phosphoproteomics were unsuccessful. Specifically, we performed global phosphoproteomics on MCF7 cells treated with CV2a and DMSO to identify changes in the ER-pT594 phosphopeptide. This analysis identified 4,951 phosphopeptides corresponding to 1,948 proteins. However, we were unable to detect phosphopeptides from ERα, including pT594, likely due to limited phosphoproteome coverage and the known analytical challenges associated with negatively charged C-terminal phosphopeptides.

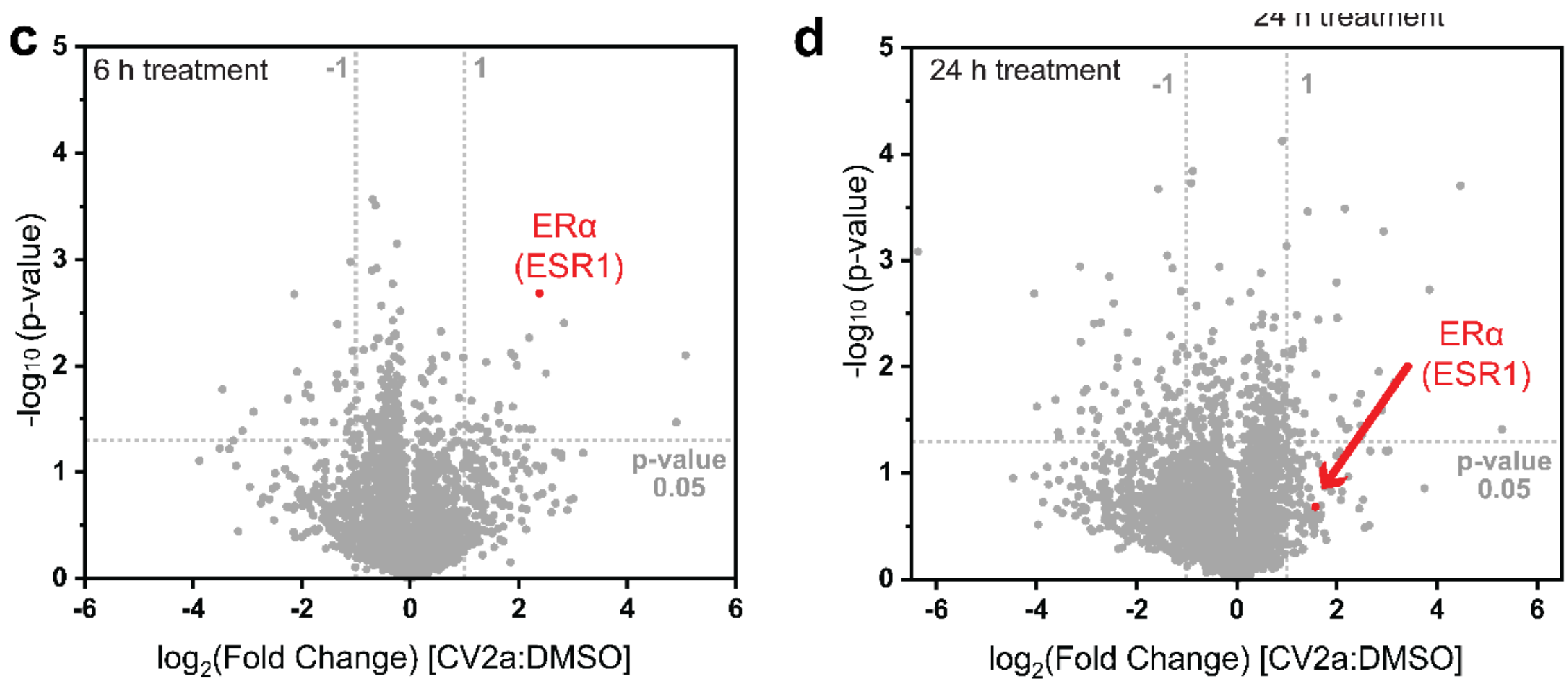

Together with the NanoBRET target engagement data, these results indicate that the MGPROTAC engages both components of the system (VHL and the 14-3-3/ERα complex) in cells.

Regarding the BRET assay, we would like to clarify that this assay primarily reports on ^{MG}PROTAC interaction with VHL and is used to assess cellular permeability and target engagement with the E3 ligase complex. Within this experimental setup, interaction with 14-3-3/ERα is not required, as the ^{MG}PROTAC binds VHL independent of the 14-3-3/ERα complex. The potential confusion may arise from the use of the MCF7 cell line, which expresses high levels of ERα. This cell line was selected to maintain consistency between the target engagement assay and the subsequent degradation studies.

Comment 3.20

Although the overall approach is interesting, from a chemical perspective turning a monovalent MG to a bifunctional MG-PROTAC seems to have certain disadvantages, including the substantially increased molecular weight, decreased ligand efficiency and, as the authors note, reduced cell permeability. These limitations might be worth mentioning in the discussion.

Response 3.20

We agree and have revised the conclusion to further discuss limitations.

'Future work should focus on translating this proof-of-concept strategy toward more drug-like molecules. To this end, moving beyond the semisynthetic natural product FCA-NAc toward fully synthetic, lower-molecular-weight molecular glue scaffolds will be essential. For example, the synthetic 14-3-3/ERα molecular glue stabilizer 181.[22] A notably challenging of this compound being that it is a covalent molecular glue stabilizer. In parallel, optimization of linker architecture and E3 ligase ligands—through reduction of hydrogen-bond donors, increased

molecular rigidity, and minimization of polar surface area—should improve overall physical chemistry properties and potential efflux liabilities.'

Reviewer #1

General remark (GR):

I thank the authors for addressing my comments.
I am satisfied with most of the answers and manuscript changes. I still have some minor concerns (the number scheme follows the same number scheme of the previous report).

GR Response 1.0: We thank review one for their in-depth feedback, we recognize their contribution to improving this work. We agree with all the reviewers' follow-up comments and below you will find a point-by-point response.

Comment 1.1

"Prior to production MD, the system was relaxed using the default Desmond relaxation protocol in three sequential steps."
The authors could provide more information about the relaxation protocol. Is it a short MD simulation? How long? Which parameters?

Response 1.1

The 'default Desmond relaxation protocol' consisted of a short MD simulation. Specifically, a 100 ps MD simulation was performed under NVT conditions at 10 K. This low-temperature MD simulation was used to remove unfavorable contacts and gently relax the system prior to production MD. To provide further clarity to the readership, in the Methods section we have added the following text:

"Prior to production MD, the system was relaxed using the default Desmond relaxation protocol consisting of a short MD simulation (100 ps) under NVT conditions at 10 K, in three sequential steps. In the first step, positional restraints were applied to all atoms of the proteins and the ligand. In the second step, restraints were applied only to the Cα atoms of the proteins. In the final step, all positional restraints were removed, allowing full relaxation of the system while preserving the overall structural integrity."

Comment 1.3

Figure S8: it would be useful to see the distances for the 3 replicas. It seems like distances for only one replica are shown. The figure description can be improved, it is not clear which atoms were used for the distance calculation. The distance in panel B looks too high for a hydrogen bond (assuming this is a donor-acceptor distance).

Response 1.3

We agree and have now provided time-dependent distance measurements for all three replicates for all heavy atoms relevant to the contact interactions between 14-3-3 and VHL identified from the cryo-EM structure. The data is now shown in Figure S15 and Figure S15 [continued]. To improve clarity, we have added titles to each panel specifying which pair of heavy atoms were used for the measurements, both within the figure and in the figure legend.

We have also included two supplementary movie files showing one of the three 500 ns molecular dynamics simulation replicates, which models the 14-3-3 monomer, CV2a, and VHL complex. Supplementary movie file 1 shows the full complex, while Supplementary

movie file 2 provides an enlarged view of the 14-3-3/VHL protein interface. Overall, the 14-3-3/VHL/CV2a interaction is globally stable over each of the 500 ns simulations.

Regarding the reviewer's question about the "the distance in panel B ", this referred to the paired-atom distances that were measured between Glu208^{14-3-3} and His110^{VHL}/Arg108^{VHL}. We have now carefully inspected these distances in all the 3 replicates of the new calculations. Visual inspection of the MD simulation shows that Arg108^{VHL} moves in and out of hydrogen-bonding distance with Glu208^{14-3-3}. When Arg108^{VHL} moves out of phase, Gln73^{VHL} moves in to fill the gap and forms a hydrogen bond with Glu208^{14-3-3}. This dynamic exchange is consistent with His110^{VHL} and Arg108^{VHL} being located on β-strands connected by flexible loops, while Glu208^{14-3-3} is in the loop region between helices α8 and α9.

By contrast, the interaction between Tyr211^{14-3-3} and Tyr112^{VHL} appears to function as the key hotspot interaction, given its stability around short pairwise distances of <5 Å over time. The additional interactions likely contribute to supporting and anchoring this interface. These structural insights will be valuable for future medicinal chemistry studies focused on optimizing the VHL ligand.

Based on this discussion, we have revised the main manuscript paragraph to read;

The MD simulation identified a network of stabilizing inter-protein interactions (Fig. S15). VHL Tyr112 hydrogen bonds to Tyr211 of 14-3-3ζ (α9 helix), which in turn hydrogen bonds with CV2a (Fig. 4a). This interaction proved to be highly stable throughout the 500 ns simulation (Figure S15c), suggesting this trivalent interaction functions as anchor point. On the same β-sheet, VHL Arg108, Asn72, and His110 form a dynamic hydrogen-bonding network with 14-3-3ζ Glu208 (Fig. 4b). Visual inspection of the MD simulation over time showed that VHL Arg108 moves in and out of hydrogen-bonding distance with 14-3-3ζ Glu208 (Supplementary Movie 2; Fig. S15b). When VHL Arg108 moves out of phase, VHL Gln73 moves in to form a hydrogen bond with 14-3-3ζ Glu208. Towards the CV2a alkylic exit point on the VHL-binding moiety, we observed VHL Asn67 and 14-3-3ζ Gln219 form a hydrogen bond (Fig. 4c). Further along the α9 helix, 14-3-3ζ Arg222 forms a hydrogen bond with the carboxylic acid group of Asp92 of VHL. Sequence alignment across the seven human 14-3-3 isoforms (β, ε, η, γ, σ, θ, ζ) showed high conservation of the previously described interface residues (Fig. S17). Notably, Asp204 of 14-3-3ζ, which hydrogen bonds with Pro99 of VHL, is substituted by His in 14-3-3σ—introducing potential electrostatic repulsion with nearby VHL Arg107 and providing a structural rationale for the reduced cooperativity observed for 14-3-3σ. Taken together, these analyses suggest that the hydrogen bond between VHL Tyr112 and 14-3-3ζ Tyr211 functions as the key hotspot interaction, given its stability over time around short pairwise distances of <5 Å, while the additional interactions support protein-protein contacts that are likely more transient in nature.

Reviewer #2

General remark (GR):

Verhoef et al. "Induced ubiquitination of the partially disordered estrogen receptor alpha protein via a 14-3-3-directed molecular glue-based PROTAC design"

I have reviewed the detailed response from the authors and found they have revised the manuscript in a highly professional manner. My concerns have been fully addressed, including comments I had initially expected to be incorporated into the Discussion. Now, I am satisfied with the revised manuscript.

GR Response 2.0:

We thank Reviewer 2 for their valuable comments, particularly regarding the in vitro ubiquitination experiments and cellular studies. This feedback has helped refine the manuscript, and all authors greatly appreciate these insights. We also appreciate their positive assessment of the work and hope that the revised manuscript will make a significant impact.

Reviewer #3

General remark (GR):

The authors have addressed most of my comments and added significant experimental details. Most of the overstatements have been rephrased and the conclusions are adequately supported by the experimental data, in a more balanced way. However, there are still some issues that need to be addressed.

Response 3

We thank review three for their in-depth feedback, we recognise their contribution to improving this work. We agree with the reviewers follow-up comments and below you will find a point-by-point response.

Comment 3.1. *The authors report two different EC50 values for FC-NAc. "Consistent with this rationale, FC-NAc binds the 14-3-3σ/ERα complex with high affinity (EC50 = 15 nM), substantially stronger than FC-A (EC50 = 421 nM)." In the next paragraph, the authors state "The monovalent molecular glue FCNAc served as a positive control (EC50= 0.34 μM)". This EC50 value is not substantially different than the one previously mentioned for FC-A.*

Response 3.1

We thank the reviewer for highlighting this inconsistency. We agree that the way the EC_{50} values were presented was confusing.

The EC_{50} value of 15 nM refers to a previously reported literature value for FC-NAc measured

with the 14-3-3σ/ERα complex (Visser et al., 2024). In contrast, the EC_{50} value of 0.34 μM corresponds to our experimentally determined value obtained using the same assay conditions (10 nM FAM-ERα and 1 μM 14-3-3σ).

To avoid confusion, we have now:

1. Explicitly cited the literature value at the point where it is mentioned.
2. Added the statement:
   "Notably, in our hands FC-NAc displayed lower potency than previously reported."

We note that such differences in EC_{50} values can arise from protein preparations, and measurement formats. The revised text now reflects this distinction clearly.

Comment 3.2. *An additional point that needs clarification is the use of different 14-3-3 isoforms in different assays. Initially, 14-3-3σ is used in the fluorescence anisotropy assays. 14-3-3ζ is used in the cryo-EM and the MD simulations, but after that for a TR-FRET assay, 14-3-3γ is used instead. If ζ and ε showed the strongest VCB stabilization, why did the authors use a different isoform for the TR-FRET assay?*

Response 3.2

We thank the reviewer for raising this point. Our choice of isoforms was guided by a combination of available protein reagents and established literature precedents.

Initial fluorescence anisotropy experiments were performed with 14-3-3σ as a well-characterized system for compound profiling fusicoccin analogues. For structural studies, we transitioned to 14-3-3ζ, as a robust co-expression system for the 14-3-3ζ/ERα complex had been previously established (e.g., Somsen et al. 2023; PMID: 37224961), which enabled reliable preparation of material for cryo-EM and resulting MD simulations. For the TR-FRET assay, we employed existing biotinylated 14-3-3γ, leveraging an established reagent from the Brunsveld lab, allowing us to implement the assay quickly and in a reproducible manner.

Importantly, 14-3-3 isoforms are highly conserved in both sequence and structure and are generally considered functionally interchangeable in client protein binding. To address potential isoform-dependent effects, we benchmarked compound activity across multiple 14-3-3 isoforms (Extended Data Fig. 3). To further, improve interpretation of the structure-function relationship we also have added Figure panel 2e. While absolute affinities may vary across isoforms the SAR trend remains consistent across treatments in presence of absence of VCB.

Comment 3.3. *In the IP-MS experiment at 24h, ERα was not significantly increased, based on the statistics. In the next paragraph the first sentence mentions "Given cell target engagement assays and 14-3-3 IP-MS experiments showed significant in cell target engagement of VHL and 14-3-3/ERα…". This seems contradictory to the results observed in the IP-MS experiment and should be re-written to accurately reflect the previous data. Additionally, the term "target engagement assays" is also confusing, since in some cases*

it indicates the quaternary complex with VHL, whereas in other cases it refers to the 14-3-3/ERα complex.

Response 3.3

We agree with both points of the reviewer. The sentence has been revised to improve clarity. Additionally, we have refined the wording to better distinguish between VHL target engagement and small molecule binding to the 14-3-3/ERα complex throughout the manuscript.

"As the in-cell VHL target engagement assay and 14-3-3 IP-MS at 6 h showed significant engagement of VHL and the 14-3-3/ERα complex, respectively, we next examined whether the 14-3-3ζ/ERα(LBD_F) complex undergoes ubiquitination in vitro as a preceding mechanistic step."

Comment 3.4. Since ubiquitination is observed both on ERα and 14-3-3ζ with both compounds CV2a and CV3a, it is not clear what the authors try to conclude with the sentence "Ubiquitination level is enhanced by cooperative quaternary complex formation and target preference is fine-tuned by the chemical linker of the MGPROTAC". If the fine-tuning was successful or the different ubiquitination levels were significant, one would expect successful degradation of the complex. As this is not the case, this conclusion should be rewritten.

Response 3.4

We agree, the sentence has been removed.

Comment 3.5. *There are several mistakes in the references. For example, in the introduction, where 14-3-3 molecular glues are cited, as ref. 19-24, only ref. 20 seems to be correct. In the discussion, the sentence "For example, the synthetic 14-3-3/ERα molecular glue stabilizer 181…" also has the wrong reference (currently ref. 22). References 57 and 58 in the text are also incorrect – possibly referring to references 58 and 59 in the list of references.*

Response 3.5

We thank the reviewer to bring this to our attention. This occurred during unlinking the references from the referencing software. The references have now been corrected.
